# Book of Posters

**DOI:** 10.1080/24740527.2023.2251871

**Published:** 2024-01-05

Poster Judging L’évaulation de l’affiche

**Regulation of mechanical hypersensitivity in a model of neuropathic pain by endogenous and exogenous amyloid-beta in the spinal dorsal horn**

Laura Bennett, Hantao Zhang and Robert Bonin

University of Toronto

**Introduction/Aim**: Synaptic plasticity that allows for memory in the brain has mechanistic and functional parallels to synaptic plasticity that occurs between neurons in the spinal dorsal horn. The small peptide, amyloid-beta (Ab), is associated with memory loss in Alzheimer’s disease but is present at endogenously low concentrations in brains of healthy individuals. We hypothesize that Ab contributes to synaptic plasticity and sensory processing in the spinal dorsal horn. Our overall aim is to modulate Ab in the spinal dorsal horn to improve hypersensitivity in pain models.

**Methods**: We used Enzyme Linked Immunosorbent Assay (ELISA) to quantify Ab in the spinal cord during central sensitization, induced via capsaicin, complete Freund’s adjuvant (CFA) and spared nerve injury (SNI). We increased Ab levels transiently via synthetic Ab and decreased Ab levels via gamma-secretase inhibitor DAPT. We used a knockout mouse model of the Ab precursor protein (APP KO) which does not produce Ab and an APP mouse model that over produces Ab (TgCRND8). Sensory sensitivity was tested using Von Frey and Hargreaves.

**Results**: Mechanical sensitivity of CFA-injected mice were not impacted by synthetic Ab or DAPT injection. Interestingly APP KO mice did not respond with a change in mechanical sensitivity after CFA whereas TgCRND8 male mice did show improved mechanical sensitivity. After SNI, both sexes showed improvement in mechanical sensitivity after DAPT injection and after Ab injection in females only.

**Discussion/Conclusions**: Taken together, our results thus far indicate modulation of Ab may play a role in the mechanical sensitivity attributed to a model of neuropathic pain.

**Medseek: R package for predicting therapies based on omics data**

Shahrzad Ghazisaeidi, Mahshad Kolahdouzan and Michael Salter

University of Toronto/Hospital for Sick Children

**Introduction/Aim**: In the past decade, with the recent advancement in sequencing technologies, gene expression profiling has increasingly gained attention. These technologies promise to reveal new molecular pathways and thereby provide the basis for novel treatments for disease. However, the majority of omics studies focus on describing a phenomenon or providing an atlas rather than finding a treatment.

**Methods**: Here, intending to close the knowledge gap, we designed a MedSeek, multi-targeting design R package that predicts potential treatments from already approved drugs by starting with data on of differentially expressed genes (DEGs). The MedSeek generates a protein-protein interactome from DEGs based on druggable nodes. MedSeek then interrogates the drug-gene interaction database (DGIb) for approved therapies and calculates the drug impact score based on the integrated value of influence (IVI) for each node.

**Results**: We tested this approach by inputting genes found to be differentially expressed in the spinal dorsal horn after peripheral nerve injury, a model of neuropathic pain, in two rodent species in both males and females. The top MedSeek hit was fostamatinib, an FDA-approved drug for Immune thrombocytopenic purpura. We found that administering R406, the active metabolite of fostamatinib, significantly reversed pain hypersensitivity in both sexes.

**Discussion/Conclusions**: Using this approach, we identified and showed the efficacy of an agent that could not have been previously predicted to have analgesic properties. Thus, we believe this approach can be useful for finding new indications for existing drugs.

**The impact of pain on task performance depends on task value**

Georgia Hadjis^a^, Amy Ying Lin^a^, Andrew Yu^a^, Ying Zhou^b^, David A. Seminowicz^c^, Dehan Kong^b^, Mary Pat McAndrews^d^ and Massieh Moayedi^a^

^a^University of Toronto Faculty of Dentistry; ^b^University of Toronto; ^c^Western University; ^d^University Health Network

**Introduction/Aim**: Pain affects cognitive processing in healthy adults. It is unknown whether its inherent salience interrupts ongoing tasks (i.e., distraction), or it competes with the ongoing task based on its inherent value, or both. If the former is true, any salient non-painful stimulus should exert the same effects as pain on tasks. If the latter is true, the value of pain can determine the extent of its interference, given that the priority of tasks depends on perceived value. We aimed to determine how pain impacts cognition using a value-based framework.

**Methods**: Forty healthy adults consented and performed procedures approved by the University of Toronto Human Research Ethics Board. They experienced two stimuli (one noxious chemical-heat and one non-painful electric control) while performing two competing tasks: a high value task ($1.00/correct response) and a low value task ($0.05/correct response). To control for salience, salience ratings of stimuli were matched using a Spearman correlation and paired samples t-test. Correct reaction times were analyzed using a linear mixed model.

**Results**: Salience ratings between stimuli were matched (r > 0.95, p < 0.05) and did not differ (p > 0.05). Compared to baseline, noxious heat selectively affected performance based on task value: it slowed reaction times on the low value (p < 0.05) but not high value task. The iso-salient electric stimulus affected performance on both tasks, regardless of value (p < 0.05).

**Discussion/Conclusions**: While a non-painful sensation impacted both tasks, the effect of pain depended on the value of each task. This suggests the impact of pain on cognition depends more on its value than its salience.

**Beclin 1 regulates neuropathic and inflammatory pain hypersensitivity in a sex-dependent manner**

Theresa Tam^a^, YuShan Tu^b^, Wenbo Zhang^b^, Sophia Farcas^b^ and Michael Salter^a^

^a^Hospital for Sick Children and the University of Toronto; ^b^Hospital for Sick Children

**Introduction/Aim**: Autophagy is a lysosome-mediated degradation pathway reported to decreases in the spinal dorsal horn after nerve injury. However, whether autophagy regulates pain hypersensitivity is unknown. Here, we addressed this by targeting a critical protein involved in autophagy initiation, beclin 1 (Becn1).

**Methods**: Mice underwent spared nerve injury (SNI) surgery as a model of neuropathic pain, or intraplantar injection of complete Freund’s adjuvant (CFA) to model inflammatory pain. Sensitivity to mechanical stimuli was assessed by von Frey assay.

**Results**: We found that intrathecal administration of a Becn1-activating peptide (BAP) reversed SNI-induced mechanical hypersensitivity in males (M) but had no effect in females (F) (BAP vs control; M: p < 0.001, n=7-10; F: p > 0.05, n=4-5). In mice with CFA, BAP reversed hypersensitivity in males, not in females (BAP vs control; M: p < 0.05, n=7-8; F: p > 0.05, n=7-8). Moreover, mice with monoallelic deletion of Becn1 (Becn1+/-) displayed greater CFA-induced hypersensitivity than wild-type, in males only (Becn1+/- vs Becn1+/+; M: p < 0.01, n=8; F: p > 0.05, n=8).

The neurotrophin BDNF is implicated in pain hypersensitivity in males by upregulating NMDA receptors in dorsal horn neurons. In males, co-administering BAP with BDNF prevented BDNF-induced hypersensitivity (BDNF+BAP vs BDNF; p < 0.01, n=6-7). Furthermore, the level of the GluN2B subunit of the NMDA receptor in the dorsal horn of Becn1+/- males is greater than that in wild-type (p < 0.05, n=7-10).

**Discussion/Conclusions**: We found that Becn1 regulates mechanical hypersensitivity in neuropathic and inflammatory pain in a sex-dependent manner. In males, activating Becn1 blocks BDNF-induced hypersensitivity and loss of Becn1 upregulates GluN2B.

**Effect of exercise on neuropathic pain: Spinal mechanisms and the role of microglia**

Holly A. Vogel^a^, Charlie H. T. Kwok^a^, Lauren Coulombe^a^, Leonardo A. Molina^a^, Yifei Dong^b^, Kyle A. Mayr^a^, Wee Yong^a^, Patrick J. Whelan^a^ and Tuan Trang^a^

^a^University of Calgary; ^b^University of Saskatchewan

**Introduction/Aim**: Disease or damage to the peripheral nerves can produce neuropathic pain, one of the most debilitating chronic pain conditions. Recent pre-clinical and clinical studies suggest that exercise reduces pain and improves motor recovery following nerve injury, but how this occurs is unclear. We test the hypothesis that exercise augments microglia function, immune cells that reside in the central nervous system, which alters pain responses.

**Methods**: Spared nerve injury surgery was performed on male and female C57BL6/J and CX3CR1creER; Rosa26tdTom mice. With an appropriate interval following tamoxifen induction, these mice enabled fate-mapped microglia to be delineated from monocyte-derived macrophages. Animals were trained on an in-house designed voluntary running wheel with an Arduino-powered base. Each animal was tagged with a unique radio-frequency identification tag to track running distances, duration, and speed. Allodynia following nerve injury was measured using von Frey filaments. Immunohistochemical changes in the lumbar spinal cord dorsal and ventral horns, both ipsilateral and contralateral to the injury, were analyzed to assess the effect of exercise on spinal circuitry and microglial profile.

**Results**: Our findings indicate that exercise before and after nerve injury impacts nerve injury-induced changes in sensory nerve innervation and motor circuitry. Specifically, nerve injury drives ventral horn microglia reactivity and increases peripheral immune cell infiltration. Exercise decreased this reactivity and was correlated with reduced nerve-injury-induced mechanical hypersensitivity.

**Discussion/Conclusions**: We demonstrate that exercise increases microglia reactivity and restores synaptic connectivity critical for sensory processing in the spinal cord. Elucidating specific cellular targets engaged by exercise presents opportunities for evidence-based treatment for neuropathic pain.

**Neuroimmune sex differences of chronic pain in multiple sclerosis**

Adam C. Wass, Ethan Chen, Julia E. R. Nickols, Bradley J. Kerr and Anna M. W. Taylor

University of Alberta

**Introduction/Aim**: Chronic pain is a common symptom in Multiple Sclerosis (MS), afflicting more than half of all patients. As MS is three times more common in women, and effective pain therapeutics are still lacking, there is a need to understand the dissociable mechanisms by which men and women process pain in MS. The transition to chronic pain involves a range of changes in pain processing regions of the brain. Previous work has shown that in an animal model of MS, Experimental Autoimmune Encephalomyelitis (EAE), microglia, the innate immune cells of the central nervous system, adopt an altered phenotype within the brain of EAE mice. We do not yet know whether there are differences in microglial response between males and females.

**Methods**: To investigate this, male and female mice were induced with EAE, and evoked pain behaviours were assessed every week until disease onset (tail paralysis). At onset, brains were dissected, and the morphology of central amygdala microglia were compared in male and female control and EAE mice. Additionally, 44 pro- and anti-inflammatory cytokines and chemokines within the limbic and sensory regions were assessed via ELISA and fluorescent in situ hybridization.

**Results**: Overall, females with EAE exhibited more of a “proinflammatory” phenotype within pain processing regions compared to males with EAE.

**Discussion/Conclusions**: This work suggests that the neuroimmune response leading to chronic pain in men and women with MS may be different, and that the development of chronic pain therapies for men and women with MS may require different approaches.

**Urinary TNF-α as a potential biomarker for the trajectory and recovery status of patients with chronic primary low back pain**

Carlos Gevers-Montoro^a^, Mariana Puente-Tobares^b^, Aléxiane Monréal^b^, Francisco Miguel Conesa-Buendía^c^, Mathieu Piché^a^, Arantxa Ortega de Mues^d^

^a^Université du Québec à Trois-Rivières; ^b^Real Centro Universitario María Cristina; ^c^Instituto de Investigación Sanitaria; Fundación Jiménez-Díaz; ^d^Fujitega Research Foundation

**Introduction/Aim**: The majority of individuals with low back pain may experience recurrent or persistent symptoms in the long term. Yet, current data cannot predict who will develop chronic low back pain and who will recover from an acute episode. Increased serum and urinary levels of the proinflammatory cytokine tumor necrosis factor-α (TNF-α) have been associated with poor recovery and persistent pain trajectory after an acute episode. The aim of this study was to measure urinary levels of TNF-α in a cohort of chronic primary low back pain (CPLBP) patients, before and after receiving manual therapy, and to assess whether these levels were associated with and predicted clinical outcomes.

**Methods**: Twenty-four patients with CPLBP were exposed to eight sessions of instrument-assisted spinal manipulation (two weekly visits for four weeks). Before and after treatment, pain intensity and disability were assessed, and urine samples of the first morning micturition collected to measure concentrations of TNF-α.

**Results**: This study confirmed previous data showing that TNF-α is elevated in urine samples of patients with CPLBP. Moreover, these values differed among patients based on pain trajectory. Thus, trajectories classified as ongoing showed greater levels of TNF-α, when compared to episodic CPLBP. Furthermore, baseline urinary TNF-α and its fluctuations predicted changes in pain and disability after receiving manual therapy.

**Discussion/Conclusions**: These findings warrant further research on the potential use of urinary TNF-α concentrations as a prognostic biomarker for poor recovery from low back pain episodes and the development of ongoing CPLBP trajectories.

**It’s all in (my) head: Do chronic pain patients view pain as more of a ‘mental experience’ than the general public?**

Madelaine Gravelle^a^, Daria Toptygina^a^, Richard Harrison^b^, Emma Borg^b^, Nat Hansen^b^ and Tim Salomons^a^

^a^Queen’s University; ^b^University of Reading

**Introduction/Aim**: Misalignment in how people think/talk about pain as either mind-centric or body-centric in clinical settings is a barrier to effective treatment. Therefore, measuring an individuals implicit beliefs about what pain “is” before initiating treatment may help improve patient outcomes. The current study aimed to a) develop a clinically useful questionnaire to measure mind/body centricity in individuals conceptualization of pain, and b) test whether chronic pain patients differ in their views of pain from the general public.

**Methods**: To assess the psychometric rigor of the Pain Priors Questionnaire (PPQ), one hundred and ninety-eight participants (Mage=36.26) were recruited via Amazon Mechanical Turk (MTurk) to electronically complete the PPQ. Of those 198, 150 individuals with no reported chronic pain condition (i.e., healthy controls) were then compared to a second sample of 344 participants with chronic pain (Mage=41.89). An independent-samples t-test was completed to look at group differences in underlying pain conceptualizations (i.e., more mind-centric or body-centric).

**Results**: A final unidimensional 18-item version of the PPQ was developed following psychometric testing (α=.9). Participants with chronic pain were found to view pain as more of a mental phenomenon rather than a product of the periphery (M=3.79, SE=0.05), in comparison to healthy controls (M=3.39, SE=0.08; t(249)=-4.25, p < .001, d=.4).

**Discussion/Conclusions**: Understanding how people conceptualize pain as either mind- or body-centric is expected to have useful clinical implications. Administering the PPQ prior to medical intervention could facilitate communication between chronic pain patients and practitioners, and/or indicate amenability towards resiliency-focused psychointervention.

**Circadian rhythmicity affects pain intensity and opioid consumption?**

Doriana Taccardi^a^, Hailey Gowdy^a^, Mitra Knezic^a^, Amanda Zacarias^a^, Etienne J. Bisson^a^, Marc Parisien^b^, Lesley Norris Singer^c^, Jennifer Daly-Cyr^c^, Daenis Camiré^a^, Rosemary Wilson^a^, Elizabeth Brown^d^, Zihang Lu^a^, Manon Choinière^e^, Luda Diatchenko^b^, M. Gabrielle Pagé^e^ and Nader Ghasemlou^a^

^a^Queen’s University; ^b^McGill University; ^c^Chronic Pain Network CircaPain Research Group; ^d^Kingston Health Sciences Centre/Chronic Pain Clinic; ^e^Université de Montréal

**Introduction/Aim**: Chronic low back pain (cLBP) contributes to most years lived with disability worldwide. Previous work suggests that chronic pain fluctuates throughout the day. Targeting circadian rhythmicity in gene expression and biopsychosocial variables is crucial to understand whether pain rhythmicity can predict functional outcomes and medication use.

**Methods**: Gene Ontology was used to identify whether circadian rhythms contribute to pain and biopsychosocial outcomes using the UK biobank (UKBB), particularly in opioid users vs non-users. Gene- and pathway-level summary results were obtained from SNP-level summary results using MAGMA. Concurrently, 63 participants with cLBP in our Kingston cohort study completed e-diary assessments tracking daily pain symptoms over 7 days at 3 times per day (8am/2pm/8pm). Blood was collected within a 12-h period (8am/8pm or 8pm/8am) to quantify changes in immune cell populations; RNA from PBMCs was used to assess changes in core circadian genes.

**Results**: Circadian pathways were significantly associated with opioid usage in UKBB participants with chronic back or musculoskeletal pain. In our Kingston cohort, only people with rhythmic or constant-low pain patterns displayed characteristic rhythms of core clock genes, which was not observed in those with constant high or mixed pain patterns. People with rhythmic pain patterns also reported reduced depressive symptoms and use of opioids compared to those with constant pain.

**Discussion/Conclusions**: These findings suggest that circadian rhythmicity, on a molecular and biopsychosocial level, influences pain intensity and opioid use. This may help better define treatment groups and future strategies tailored to circadian rhythmicity.

**Qualitative research methods in pediatric pain and gender diversity: Youth experiences and lessons learned**

Katelynn Boerner^a^, Danya Fox^b^, Levi Du^c^, Sheila Marshall^a^, Daniel Metzger^b^, Eva Moore^b^, Pam Narang^b^, Marie-Noelle Wharton^a^ and Tim Oberlander^b^

^a^University of British Columbia; ^b^University of British Columbia & BC Children’s Hospital; ^c^Lived Experience Consultant

**Introduction/Aim**: Gender-diverse youth experience increased risk for pain related to inadequate healthcare access, transphobic violence, minority stress, and pain from gender-affirming binding or surgery. While guidelines for research with gender-diverse populations exist (Adams et al., 2017; Bauer et al., 2019), gender-diverse youth are underrepresented in pain research. This project describes methods used to ensure safe and ethical data collection in a qualitative study on pain in gender-diverse youth, and the experiences of youth who took part.

**Methods**: 19 gender-diverse youth completed semi-structured virtual interviews about their chronic pain experiences. Methods were in place to promote autonomy, confidentiality, and comfort, such as partnership with a person with lived experience, assessment of youth capacity to consent, and flexible participation options (e.g., to turn off their camera, have a support person present). At the end of each interview, youth were asked about their participation experience.

**Results**: Youth reported positive experiences of participation, with research team interactions described as respectful, welcoming, safe, and professional. Some reported experiencing benefit from having space to tell their story. Many reflected on the need for research in this area, which motivated them to participate despite having to discuss challenging topics. The research team experienced challenges in balancing safety and flexibility, e.g., an identity verification step was added after potential incidents of fraudulent responding.

**Discussion/Conclusions**: There is an urgent need for research regarding the experience and care of chronic pain in gender-diverse youth. Findings suggest that steps to protect the safety and autonomy of gender-diverse youth facilitated their participation in pain research.

**“All of my pain was dismissed”: Narratives of chronic pain in emerging adult women**

Jenise Finlay, Añiela dela Cruz, Graham McCaffrey and Andrew Estefan

University of Calgary

**Introduction/Aim**: Emerging adult women are at higher risk for insufficient pain management and face unique challenges navigating chronic illness, dating, body image, college, careers, establishing independence from family, and bearing children at an age where youth is equated to being healthy. An increasing prevalence in chronic pain has been observed across all age groups in Canada, most notably among those aged 20 to 29 with no other health conditions, yet few qualitative studies examine chronic pain exclusively in women under 30. The purpose of this narrative inquiry was to understand how the experience of living with chronic pain affects the identity of emerging adult women aged 18 to 29.

**Methods**: Clandinin and Connelly’s form of narrative inquiry was used to explore the lived and told stories of two emerging adult women living with chronic pain, gaining a deeper understanding of how their experiences shape, and are shaped by, social, cultural, familial, and institutional narratives. Data were generated through composition of field texts that included in-depth conversational interviews, participant artwork, and the first author’s field journal writing. Transitioning from field texts to research texts, narrative accounts were then co-composed with research participants.

**Results**: Narrative threads that resonated across narrative accounts include: silenced, invisible, and locating self with pain; pain experiences storied through relationships; and resisting the singular stories of people living with chronic pain.

**Discussion/Conclusions**: Personal, practical, and social significance of this work are discussed with implications for nursing practice, health education, research, and policy before concluding with final reflections.

**A multimethod examination of inflammatory bowel disease impacts on patient’ sexual lives**

Katherine Fretz, Katherine Hunker and Dean Tripp

Queen’s University

**Introduction/Aim**: Inflammatory bowel disease (IBD) is a painful, chronic gastrointestinal condition with varied biopsychosocial sequelae. Despite sexual difficulties being elevated among IBD patients, there is little holistic research investigating the impacts of the disease on patients’ sexual lives. We used a multimethod approach to test a biopsychosocial model of sexual functioning (Objective 1) and elucidate the impacts of IBD on patients’ sexual and intimate lives (Objective 2).

**Methods**: An international sample of n=470 adults with IBD completed our online survey. Objective 1 data were collected through questionnaires and analyzed using multiple regression. Objective 2 data were collected via patients’ written responses and analyzed using reflexive thematic analysis.

**Results**: Regression results for Objective 1 suggested that greater IBD disease activity, body image dissatisfaction, and sexual script flexibility (i.e., adaptability in responses to sexual problems) predicted lower levels of IBD-related sexual dysfunction. For Objective 2, thematic analysis resulted in eight themes encompassing the ways in which IBD had impacted patients’ intimate and sexual lives. In sum, patients’ sexuality was negatively affected in numerous ways (i.e., sexual activity, dating and relationships, self-perception) through various means (i.e., disease-related factors, anxieties, and other mental health issues).

**Discussion/Conclusions**: These multimethod findings indicate that the sexual lives of IBD patients are impacted by disease related factors and psychosocial constructs. Clinicians should be mindful of the biopsychosocial impact of IBD on sexual functioning. Future research and knowledge translation should be aimed at informing holistic assessment and treatment recommendations for this important area of IBD patient well-being.

**“It’s a wheel that turns”: An interpretative phenomenological analysis of military personnel’s sense-making of comorbid PTSD and chronic pain**

Larah Maunder^a^, Joel Katz^b^ and Tim Salomons^a^

^a^Queen’s University; ^b^York University

**Introduction/Aim**: Chronic pain and posttraumatic stress disorder (PTSD) are debilitating conditions that co-occur at high rates. This has prompted investigators to propose theoretical models identifying mechanisms driving this high comorbidity. Unfortunately, these models were almost exclusively developed through the identification of similarities between PTSD and chronic pain when they occur separately, rather than through in-depth interviews of individuals with both conditions. Through detailed analysis of specific cases of individuals with comorbid PTSD and chronic pain, qualitative work may generate further insights into how the conditions may interact and maintain each other. Thus, we conducted a qualitative analysis to investigate how Canadian Armed Forces personnel with both conditions make sense of their experiences of chronic pain and PTSD.

**Methods**: Eight CAF personnel were interviewed about their experiences of PTSD, chronic pain, and the relationship between the two conditions. An Interpretative Phenomenological Analysis was conducted on the interview data.

**Results**: Three themes emerged from the analysis. “Ghost Pains” describes how participants view their current pain as a re-experiencing of the pain they felt during a traumatic event. “It’s a Wheel that Turns” details how PTSD symptoms trigger pain, and vice-versa. “Struggling Past the Traumatized Self” describes identity loss resulting from living with chronic pain and PTSD, and efforts to recover from that loss.

**Discussion/Conclusions**: This study elucidated mechanisms by which PTSD and chronic pain may maintain each other. For instance, pain may act as a trauma trigger for those with PTSD, while intrusion symptoms (e.g., nightmares) increase bodily tension and exacerbate pain.

**Chronic pain care delivered remotely from a community pain clinic: Providers and patients experience**

Brenda Lau^a^, Emmanuel Abreu^b^, Neha Singh^b^, Katherine Zhang^b^, Tameus Venkataraman^a^ and Milan Zivadinovic^b^

^a^CHANGEPain Clinic/University of British Columbia; ^b^CHANGEPain Clinic

**Introduction/Aim**: The COVID-19 pandemic forced healthcare centers to evolve into virtual modalities. New modalities to deliver care remotely are being implemented across Canada. The perspective of patients is pivotal during the conception of these practices. This study aimed to (1) Understand the sociodemographic characteristics of patients receiving care remotely at a community pain clinic (2) Gather information from providers’ experiences to develop a code of conduct that can be applied during online interventions.

**Methods**: Institutional Review Board approval from the University of British Columbia, 1721 patients that had participated in virtually delivered appointments (group medical visits or consultations) and 58 providers were surveyed. The study followed a mixed-method approach. A python script was developed to calculate patients’ travel times to the clinic. All quantitative data was processed using SPSS. Qualitative data from the in-depth responses was analyzed and the grounded theory approach was used.

**Results**: 304 responses were received from patients and 44 by providers. The average patient in the study lived 64.3 km from the facility. 64% of patients declared receiving financial assistance from the Canadian government in the last 12 months. 76% of patients report being satisfied with their telemedicine interactions at the clinic. 86% of providers felt comfortable delivering care virtually and aim to continue even when it is not mandatory. Qualitative answers showcased how the telehealth delivery was beneficial for patient’s condition.

**Discussion/Conclusions**: Virtually delivered pain care satisfies both patients and providers. More research is needed on the effectiveness of these practices compared to the standard of care.

**Where we live matters: Treatment of chronic pain in remote regions of Quebec, Canada**

Claudie Audet^a^, Meriem Zerriouh^a^, Hermine Lore Nguena Nguefack^a^, Nancy Julien^a^, Gabrielle Pagé^b^, Line Guénette^c^, Lucie Blais^d^ and Anaïs Lacasse^a^

^a^Université du Québec en Abitibi-Témiscamingue; ^b^Centre de recherche du Centre hospitalier de l’Université de Montréal (CRCHUM), Département d’anesthésiologie et de médecine de la douleur, Faculté de médecine, Université de Montréal; ^c^Faculté de pharmacie, Université Laval, Centre de recherche du CHU de Québec - Université Laval; ^d^Faculté de pharmacie, Université de Montréal

**Introduction/Aim**: Where a person lives is a recognized socioeconomic determinant of health and influences health care access. This study aimed to compare the pain treatment profile of people with chronic pain (CP) living in remote regions vs. near or in major urban centres.

**Methods**: A cross-sectional study was performed among people living with CP across Quebec. In a web-based questionnaire, participants were asked to report in which of the 17 administrative regions they were living (six are defined as “remote”). Pain treatment profile was drawn up using seven variables: use of prescribed pain medications, over-the-counter pain medications, non-pharmacological pain treatments, multimodal approach (pharmacological + non-pharmacological), access to a trusted health care professional for pain management, excessive polypharmacy (≥10 medications), and cannabis use for pain. Chi-square tests were performed.

**Results**: 1933 participants completed the questionnaire (women: 83.4%, mean age: 50 years, people living in remote regions: 23.8%). As compared to persons living in remote regions, those living near or in major centres were more likely to report using prescribed pain medications (83.8% vs. 67.4%, p=< 0.001), a multimodal approach (81.5% vs. 75.5%, p=0.017), experience excessive polypharmacy (28.1% vs. 19.1%, p=0.001), and report using cannabis for pain (33.1% vs. 20.7%, p=< 0.001). No statistically significant differences were found regarding the three other variables (p > .05).

**Discussion/Conclusions**: There are differences in treatment profiles of people with CP depending on where they live. Our results highlight the importance of considering remoteness, and not only rurality, when it comes to better understand the determinants of pain management.

**Comprendre le stress au quotidien, ses caractéristiques, et son impact sur la douleur et sa gestion**

Mael Gagnon Mailhot^a^, Karen Ghoussoub^a^, Élise Develay^b^, Mathieu Roy^c^, Étienne Vachon-Presseau^c^, Pierre Rainville^d^, Sonia Lupien^e^, Lise Dassieu^b^ and Gabrielle Pagé^a^

^a^Université de Montréal, Centre de recherche du Centre hospitalier de l’Université de Montréal; ^b^Centre de recherche du Centre hospitalier de l’Université de Montréal; ^c^Université McGill; ^d^Centre de recherche de l’Institut Universitaire de Gériatrie de Montréal; ^e^Université de Montréal

**Introduction/Aim**: L’objectif est d’explorer les perspectives des personnes vivant avec de la douleur chronique concernant les associations entre le stress et la perception de la douleur, en mettant l’accent sur les caractéristiques pouvant générer une réponse physiologique du stress (perception de contrôle faible, imprévisibilité, nouveauté, et égo menacé [CINÉ]).

**Methods**: Ce projet fait partie d’une étude longitudinale à devis mixte séquentiel auprès d’individus recrutés à partir d’une cohorte provinciale de douleur lombaire. Afin d’obtenir un échantillon hétérogène, ce volet qualitatif a recruté 29 participants en fonction du degré d’association entre les scores quotidiens de stress et douleur colligés à l’aide d’un journal électronique. Le verbatim d’entretiens virtuels semi-structurés a été analysé selon une approche thématique réflexive.

**Results**: Le stress et la douleur semblent s’influencer de façon complexe et bidirectionnelle. Le stress était décrit comme pouvant augmenter la perception de la douleur, mais plusieurs participants décrivaient la douleur comme une source de stress de par sa nature souvent imprévisible et incontrôlable. Pour certains, les techniques de gestion de la douleur utilisées visaient l’atténuation d’une ou plusieurs des caractéristiques CINÉ. Par exemple, l’activité physique permettant un meilleur contrôle sur le fonctionnement quotidien et la douleur. Dans certains cas, le stress avait un effet inhibiteur momentané ou prolongé sur la douleur lorsqu’il était utilisé comme catalyseur de motivation et d’énergie.

**Discussion/Conclusions**: Les résultats préliminaires de cette étude suggèrent qu’il y a plusieurs profils de stress et de douleur qui interagissent de manière distinctive. Ces résultats pourraient mener au développement de nouvelles approches de gestion de la douleur chronique.

**Prevalence of polypharmacy among adults living with chronic pain: A Quebec prescription claims database study**

Hermine Lore Nguena Nguefack, Ghita Zahlan, Gwenaëlle De Clifford-Faugère and Anaïs Lacasse

Université du Québec en Abitibi-Témiscamingue (UQAT)

**Introduction/Aim**: Polypharmacy, which is the simultaneous use of multiple medications, is common in persons living with chronic pain. However, prevalence estimates may vary depending on definitions and data sources. This study aimed to estimate the prevalence of polypharmacy (≥5 medications) and excessive polypharmacy (≥10 medications) using Quebec public prescription drug insurance data.

**Methods**: The TorSaDE Cohort (n=102,148), which links 2007 to 2016 cycles of the Canadian Community Health Survey with Quebec administrative databases (longitudinal health insurance data) was analyzed. Descriptive and bivariate analyses (frequencies, percentages, Chi-square tests) were used to determine the prevalence of polypharmacy and excessive polypharmacy among ≥18-year-old participants living with chronic pain (n=15,693) who were covered by the public drug insurance plan during the 30-day period before survey completion (n=9,526).

**Results**: Among participants, 64.2% were females and the mean age was 63.2 ± 15.7. Prevalence was 47.3% for polypharmacy and 16.2% for excessive polypharmacy. Prevalence of excessive polypharmacy was significantly higher among females (17.7%) than males (13.6%, p < .0001) and increased with participants’ age (18-24 years: 0.0%, 25-44 years: 2.4%, 45-64 years: 13.4%, ≥ 65 years: 21.4%, p < .0001). Results of a multivariable analysis of factors associated with excessive polypharmacy will also be presented.

**Discussion/Conclusions**: Prevalence estimates of polypharmacy found in the present study are lower than recent Quebec estimates based on patient self-report. Excessive polypharmacy, a risky clinical situation, is common (almost one in five people) and requires access to adequate clinical follow-up for all persons living with chronic pain in this situation.

**The efficacy of a multisession theta-burst repetitive transcranial magnetic stimulation (rTMS) protocol on pain and functional recovery in patients with an isolated upper limb fracture (IULF)**

Lea Proulx-Begin^a^, Marianne Jodoin^b^, Daphnée Brazeau^a^, Alberto Herrero Babiloni^c^, Catherine Provost^d^, Audrey Bellemare^a^, Dominique Rouleau^a^, Caroline Arbour^a^ and Louis De Beaumont^e^

^a^Université de Montréal; ^b^Hôpital du Sacré-Coeur de Montréal (CIUSSS du Nord de-l’Île-de-Montréal;^c^ McGill University; ^d^Hôpital du Sacré-Coeur de Montréal (CIUSSS du Nord de-l’Île-de-Montréal); ^e^Université de Montréal

**Introduction/Aim**: Pain is the most prevalent symptom following a fracture and affects functional recovery. Despite known deleterious side effects, pharmacological approach is often used for acute pain following a fracture. rTMS, a non-invasive brain stimulation technique with minimal side effects, is another approach to pain management. This study investigated the effectiveness of a multisession rTMS protocol on pain and functional recovery in IULF patients.

**Methods**: 83 patients were assigned to one of the 3 groups: 1)rTMS: n=27, 2)SHAM: n=23 or 3)Control: n=34. Groups 1-2 were recruited within 2 weeks post-fracture and underwent 12 laboratory visits (rTMS or SHAM) over 2 consecutive weeks, and a final visit at 3-month post-fracture. Reported pain (NRS) and functional disability (DASH) levels were collected at each time point. Group 3 was recruited over the phone at 3-month post-fracture and completed the same measures.

**Results**: A chi-square analysis indicated a significant between-group difference in the proportion of patients reporting chronic pain (NRS≥4) at 3-month post-fracture (p=0.015). While no patient from the rTMS group reported clinical pain levels, 8.7% from the SHAM and 27.3% from the CTL groups did. A between-group ANOVA also revealed a significant difference at 3-month post-fracture on DASH scores (F(2,79)=12.75, p < 0.0001). Patients in the rTMS group (M=8.30 ± 10.30) reported significantly less functional disabilities than SHAM patients (M=17.95 ± 16.48), whereas control patients (M=28.23 ± 17.28) showed poorest functional recovery.

**Discussion/Conclusions**: These results show the beneficial effects of an acute rTMS intervention on patients’ reported pain levels and functional recovery and underscore the pertinence of sustained patient care during the acute post-fracture phase.

**Who is experiencing excessive polypharmacy? A cluster analysis among persons living with chronic pain in Quebec**

Ghita Zahlan^a^, Hermine Lore Nguena Nguefack^b^, Gwenaelle De Clifford-Faugère^b^, M. Gabrielle Pagé^c^, Line Guénette^d^, Lucie Blais^e^, and Anaïs Lacasse^f^

^a^Université du Québec en Abitibi-Témiscamingue (UQAT); ^b^Université du Québec en Abitibi-Témiscamingue (UQAT); ^c^Centre de recherche du Centre hospitalier de l’Université de Montréal (CRCHUM), Faculté de médecine, Université de Montréal; ^d^Faculté de pharmacie, Université Laval, Québec, Québec, Canada, Centre de recherche du CHU de Québec - Université Laval, Québec, Québec, Canada; ^e^Faculté de pharmacie, Université de Montréal, Montréal, Québec, Canada; ^f^Université du Québec en Abitibi-Témiscamingue (UQAT)

**Introduction/Aim**: Persons living with chronic pain (CP) are at risk of excessive polypharmacy, which is the concomitant use of ≥10 medications. This study applied cluster analysis to identify distinct subgroups of persons living with CP experiencing excessive polypharmacy.

**Methods**: This cross-sectional observational study used data from 1342 persons living with CP. A multivariable logistic regression using excessive polypharmacy as the dependent variable and various clinically relevant independent variables was first conducted to identify the most discriminant input variables. A two-step cluster analysis was then performed with excessive polypharmacy, pain intensity, feeling that pain is terrible, and it’s never going to get better, use of prescribed or over-the-counter pain medications, access to a trusted health care professional for pain management, general health and physical functioning (SF-12), country of birth, and employment.

**Results**: The analysis revealed two distinct clusters (C1 and C2). In C1 (32% of the sample), none of the participants reported excessive polypharmacy. This group was characterized by a lower pain intensity and better general health and physical functioning. C2 (64% of the sample) regrouped all participants reporting excessive polypharmacy, which formed 38% of this group. The proportion of participants employed and born in Canada was higher in C2 (p < .05). They also reported more severe pain intensity, feeling that pain is terrible, and use of prescribed pain medications.

**Discussion/Conclusions**: Various pain, health and sociodemographic factors are discriminant to classify persons living with CP experiencing excessive polypharmacy and who could benefit from more personalized prevention/follow-up.

**Animal or Non-Human pain La douleur animale ou non humaine Tracing pain: Repetitive mild traumatic brain injury modifies nociception and orexinergic connectivity to the descending pain modulatory pathway**

Jennaya Christensen, Naomi MacPherson, Crystal Li, Glenn R. Yamakawa and Richelle Mychasiuk

Monash University

**Introduction/Aim**: Mild traumatic brain injury (mTBI) is a prevalent and debilitating condition, with particularly high incidence among males. There is a high co-occurrence rate between chronic pain and mTBI, with approximately 1/5 mTBI patients developing chronic pain. Additionally, mTBI and chronic pain have a bi-directional relationship with anxiety and sleep disturbances. Although the mechanisms driving this co-occurrence are unknown, it may relate to dysfunctional descending pain modulation (DPM). Given that orexin regulates the sleep-wake cycle and is a potent anti-nociceptive neuromodulator, altered orexinergic functioning may contribute to DPM dysfunction following mTBI. Furthermore, the orexinergic system receives excitatory innervation from the lateral parabrachial nucleus (lPBN), thus modulating orexinergic functioning. Therefore, we used neuronal tract-tracing to investigate how repetitive mTBI (RmTBI) affects orexinergic connectivity to the lPBN and periaqueductal gray (PAG), a key site in DPM.

**Methods**: Tract-tracing surgery targeting the lPBN or PAG was performed on 56 young adult male Sprague Dawley rats before they were then randomly assigned to RmTBIs or sham injuries followed by testing for anxiety-like behaviour and nociceptive sensitivity. Immunohistochemical analysis was used to identify distinct and overlapping orexin and tract-tracing neurons within the LH.

**Results**: The RmTBI group exhibited significantly altered nociceptive sensitivity and anxiety-like behaviour (p < .05). Furthermore, a significant (p < .05) loss of orexin cell bodies and reduced hypothalamic connectivity to the lPBN and PAG was identified post-injury.

**Discussion/Conclusions**: These structural losses and physiological changes in the orexinergic system following RmTBI improve our understanding of the acute mechanistic changes that occur post-injury and drive the chronification of pain.

**Repeated exposure to sucrose and pain induces alterations in pro/anti-inflammatory cytokine levels in hippocampus and blood in neonatal mice**

Fermin Hoq^a^, Rujun Kang^b^, Annie Ciernia^a^, Kiran Soma^a^ and Manon Ranger^c^

^a^University of British Columbia; ^b^BC Children’s Hospital Research Institute; ^c^University of British Columbia, BC Children’s Hospital Research Institute

**Introduction/Aim**: In the neonatal intensive care unit, preterm infants can experience 7-17 painful procedures daily. Oral sucrose is the standard treatment for procedural pain, but their combined short- and long-term cumulative effects on brain development remain unclear. Using a neonatal mouse model, we previously showed that repeated pain and/or sucrose exposure in the 1st week of life resulted in reduced hippocampal and white matter volumes and poorer short-term memory in adulthood.

This study aimed to determine whether repeated neonatal sucrose and/or pain exposure affect pro/anti-inflammatory markers in serum and hippocampal tissue of mouse pups.

**Methods**: Neonatal mice were randomly assigned to receive water or 24% oral sucrose prior to handling or being needle-pricked, 10X/day on postnatal day (P) 1-6. Blood and hippocampal tissue were collected at P8 and assayed for various cytokines (e.g. IL-1β, IL-10, IL-6, and TNF-α).

**Results**: While no sex effects were evident, a significant group effect was found for several pro/anti-inflammatory cytokines in serum and hippocampal tissue. Mouse pups exposed to sucrose and pain showed significantly lower serum anti-inflammatory IL-5 levels compared to all other groups, including controls. Furthermore, hippocampal anti-inflammatory IL-10 levels were reduced in almost all treatment/intervention groups compared to controls. Preliminary data investigating microglial density in the hippocampus suggests a trend that at P8, male mouse pups exposed to any treatment or intervention exhibit increased microglial density compared to controls.

**Discussion/Conclusions**: Our findings add to the understanding of possible underlying mechanisms that are driving the adverse effects of pain/sucrose on the developing brain.

**Investigating a rodent model of neonatal and postnatal trauma on dam health and pup nociception and anxiety**

Zoe Kodila, Marissa Sgro, Jennaya Christensen, Crystal Li, Sabrina Salberg, Sandy Shultz, Glenn Yamakawa and Richelle Mychasiuk

Monash University

**Introduction/Aim**: Early life factors such as prenatal trauma caused by intimate partner violence (IPV) or postnatal physical neglect have the potential to influence chronic pain development. Therefore, we aimed to develop a rodent model that combined neonatal and postnatal trauma and subsequently measured dam health and pup nociceptive and behavioural outcomes.

**Methods**: Dams were randomly assigned to IPV (mild traumatic brain injury and hypoxia) or sham injury, at gestational day 17. Following birth, offspring were separated from the dams for 4 hrs/day from postnatal day P2-12 or undisturbed. The pups were assessed for changes in nociception and anxiety-like behaviours.

**Results**: Experiencing the IPV during pregnancy did not significantly modify pregnancy-related outcomes including weight gain (p=0.733), litter size (p=0.850),) or number of pups reabsorbed (p=0.667). There were no differences in pup weight at P21 (p > 0.05), although IPV pups weighed less at P35 and P48 (p < 0.01). There were no significant differences in anxiety-like behaviour (p=0.411). IPV exposure in utero did significantly reduce time to respond on the hot plate (p=0.02), and on the cold plate for males (interaction, p=0.03). Pups born to IPV dams also exhibited increased sensitivity on the Von Frey test (p=0035).

**Discussion/Conclusions**: IPV during pregnancy did not significantly alter dam characteristics during gestation. Offspring weight was modified by IPV in adolescence but not at weaning. IPV also modified offspring nociception as detected by measures of thermal and mechanical sensitivity. Further investigation of these findings will allow us to understand the intergenerational transmission of risk for chronic pain.

**Sexually-dimorphic grey matter density and microbiome changes in a preclinical model of adolescent post-surgical pain following high-fat high-sugar diet administration**

Sabrina Salberg^a^, Angela Doshen^b^, Matthew Macowan^c^, Glenn R. Yamakawa^a^, Benjamin J. Marsland^c^, Luke Henderson^b^, Richelle Mychasiuk^a^

^a^Department of Neuroscience, Monash University, Melbourne, VIC, Australia; ^b^School of Medical Sciences (Neuroscience), Brain and Mind Centre, University of Sydney, NSW, Australia; ^c^Department of Immunology and Pathology, Monash University, Melbourne, VIC, Australia

**Introduction/Aim**: Although the etiology of adolescent post-surgical pain is poorly understood, several factors may contribute, such as female sex and poor diet. Therefore, we investigated the effect of sex and diet following a minor plantar incision (PI) surgical procedure on behaviour, neuroanatomy, and the microbiome.

**Methods**: Sprague Dawley rat dams were randomly assigned to a standard or high-fat high-sugar (HFHS) diet. Male and female pups (maintained on the same diet) were further randomized into sham or PI conditions (n=6M+6F/group). Chronically, the vonFrey and hot plate tests measured mechanical and thermal nociception respectively. This was followed by MRIs for regional grey matter density analysis, and fecal analyses of 16S rRNA. Three way-ANOVAs with sex, diet, and injury were run.

**Results**: The HFHS and PI groups had increased mechanical nociceptive sensitivity (p < .001). Females exhibited increased thermal nociceptive sensitivity compared to males (p < .001). Significant injury effects in regional grey matter density were identified in the cerebellum, amygdala, insula, caudate putamen, hippocampus, and nucleus accumbens (p’s < 0.05, voxel-by-voxel analysis), with females driving many of the effects. A significant effect of diet on the amygdala and entorhinal cortex was also observed (p < 0.05). Initial 16S rRNA analysis demonstrates a significant effect of diet on genera distribution and bacterial diversity, with correlational analysis between MRI and microbiome ongoing.

**Discussion/Conclusions**: We found persistent post-surgical pain following HFHS diet, with sexually-dimorphic changes in nociceptive sensitivity and regional grey matter density. We suggest that diet may be contributing to pain through neuroanatomical and microbiome changes in a sex-dependent manner.

**The gut microbiome differentially modifies nociceptive thresholds and peripheral circulating cytokines in adolescent and adult rats following repetitive mild traumatic brain injury**

Marissa Sgro, Zoe Kodila, Crystal Li, Glenn Yamakawa and Richelle Mychasiuk

Monash University, Central Clinical School

**Introduction/Aim**: Recent literature has demonstrated the influence of mild traumatic brain injury (mTBI) on the microbiota-brain-gut axis, with its alteration perpetuating neuroinflammation. However, there has been no investigation into the effects that the microbiome may have on RmTBI (Repetitive mTBI) induced inflammation and chronic pain development in adolescents and adults.

**Methods**: We compared the effects of microbiome depletion on pain following RmTBI in adolescence and adulthood. Specifically, half of the adolescent and adult rats (40F:40M, 44F:48M, respectively) were administered an antibiotic cocktail or placebo in their drinking water for 14 days. Rats were randomly assigned to receive 3 mild TBIs (RmTBI) or sham injuries. Pain sensitivity via hot/cold plate and anxiety testing via elevated plus maze (EPM) were conducted. A Milliplex Rat Cytokine Panel was run to determine circulating cytokine levels.

**Results**: Four-way ANOVAs with age (Adolescent/Adult), sex (Female/Male), treatment (Antibiotic/Placebo), and injury (RmTBI/Sham) as factors were run, followed by Bonferroni pairwise post-hoc analyses. For the hot plate, Antibiotic+Sham adolescents were less sensitive than adults (p < .01). Adult placebo+RmTBI rats were more sensitive than adult Placebo+Shams (p=.01). On the EPM, microbiome depletion increased anxiety-like behaviour and adolescents were more anxious than adults. Cytokine data demonstrated that adults had increased inflammation, compared to adolescents, suggesting that adolescents may not be mounting an appropriate inflammatory response.

**Discussion/Conclusions**: Microbiome recolonization differentially influenced RmTBI-induced pain sensitivity and peripheral inflammation in adolescents and adults, which may provide insight as to why adolescents are often more susceptible to poorer mTBI outcomes than adults.

**The waiting game: Investigating the neurobiological transition from acute to persistent pain in adolescent rats**

Glenn Yamakawa^a^, Sabrina Salberg^a^, Angela Doshen^b^, Jillian Vinall Miller^c^, Melanie Noel^c^, Luke Henderson^b^ and Richelle Mychasiuk^a^

^a^Monash University; ^b^University of Sydney; ^c^University of Calgary

**Introduction/Aim**: Persistent post-surgical pain affects 20% of youth undergoing a surgical procedure, with females exhibiting increased prevalence of chronic pain compared to males. This study sought to examine the sexually-dimorphic neurobiological changes underlying the transition from acute to persistent pain following surgery in adolescence.

**Methods**: Thirty-three male and female Sprague Dawley rats were allocated to a sham or injury (plantar-incision surgery) condition and assessed for pain sensitivity while also undergoing magnetic resonance imaging at both an acute and chronic timepoint within adolescence.

**Results**: Injury resulted in persistent pain in both sexes (p < .05), with females displaying greater nociceptive sensitivity. Significant differences in regional grey matter density, between sham and injured rats were determined at a voxel-by-voxel level by placing the grey matter density brain maps into a second level full factorial analysis with 3 factors and 2 levels in each factor; Injury (Sham:Injured), sex (male:female), Timepoint (Acute:Chronic) (p < 0.05, False Discovery Rate (FDR)-corrected, minimum cluster size 20 contiguous voxels). Total grey matter density was increased in the female injured group, and injury altered grey matter density in brain regions such as the cerebellum, with female driven changes in the amygdala and insula and male driven changes in the hippocampus and lateral orbital cortex. Differences between chronic and acute density in the cerebellum and hypothalamus were directly correlated with change in pain sensitivity across time.

**Discussion/Conclusions**: Our results indicate persistent behavioural and neurobiological changes following surgery in adolescence, with sexually-dimorphic and age-specific outcomes, highlighting the importance of studying both sexes and adolescents.

**Chronic pain mediated changes in the hedonic value of gentle touch in mice**

Maham Zain, Laura Bennett, Quinn Pauli, Hantao Zhang and Robert Bonin

University of Toronto

**Introduction/Aim**: Dysregulation of gentle touch processing is a hallmark of many disorders including chronic pain where gentle touch becomes painful and aversive. Sensory neurons expressing MrgprB4 detect gentle stroking in mice and their activation is known to be positively reinforcing. This project uses optogenetics and behavioral techniques to assess whether activation of channelrhodopsin (ChR2) expressing MrgprB4+ afferents signal positively valenced tactile information, whether this is altered in chronic pain and whether this is reflected in the downstream circuits recruited.

**Methods**: A ceramic ferrule was surgically implanted in the lumbar vertebrae of mice expressing ChR2 in MrgprB4+ afferents (MrgprB4-ChR2) to deliver blue light to the central projections of the primary afferents. We used a real-time place preference (RTPP) paradigm with optogenetics to assess the motivational properties of blue light stimulation in implanted male and female MrgprB4-ChR2 mice that had either undergone a spared nerve injury (SNI) or a sham surgery and assessed whether gabapentin administration affected the response. All mice underwent a final stimulation protocol after which the brains and spinal cords of the mice were dissected out for immunohistochemical analysis of c-fos expression.

**Results**: Light stimulation in one arm of the assay increased preference for that arm in the sham surgery mice but not the SNI animals. Preference for blue light could be restored in the SNI mice through treatment with gabapentin. Preliminary results from the immunohistochemistry experiments also show that stimulation successfully induced c-fos expression in the spinal dorsal horn of the stimulated animals and this expression was predominantly found in lamina II-III of the spinal cord, consistent with the innervation pattern of the MrgprB4+ afferents. Whole brain c-fos results also showed differential recruitment of brain regions following optogenetic activation of MrgprB4+ fibers in sham and nerve injured mice.

**Discussion/Conclusions**: In conclusion, the motivational value associated with gentle touch is plastic and can be abated in models of chronic pain. Future work will continue to elucidate the subtypes of spinal dorsal horn neurons recruited and the specific brain regions activated in the processing and perception of gentle touch both in chronic pain and in control conditions.

**Age related changes of systemic immune profile in nerve injured male and female mice**

Wen Bo Sam Zhou^a^, Xiang Qun Shi^a^, Magali Millecamps^b^, Jeffrey Mogil^a^, Younan Liu^c^, Simon Tran^c^ and Ji Zhang^a^

^a^McGill University, Alan Edwards Center for Research on Pain; ^b^ABC Platform, Alan Edwards Centre for Research on Pain, McGill University; ^c^McGill University

**Introduction/Aim**: Aging is associated with a higher prevalence of many chronic non-communicable diseases including chronic pain, and there is a higher prevalence of chronic pain in women than in men. Aging is also associated with low-grade systemic chronic inflammation (inflammaging). However, the relationships between inflammaging, chronic pain, and sex difference have not been fully understood.

**Methods**: We performed the spare nerve injury (SNI) and sham surgery on 3-month-old male and female mice, and longitudinally monitored them for 2 years. We monitored mechanical and cold sensitivities with von Frey and acetone tests, immune cell compartments with flow cytometry, and changes in serum cytokines and chemokines with the Luminex multiplex assay. We also treated 23-month-old SNI mice with mesenchymal stem cell extracts (MSCE), as our previous data showed its effectiveness in alleviating neuropathic pain in male young nerve-injured mice.

**Results**: Both male and female SNI mice exhibited persistent, stable mechanical and cold allodynia over the 2 years following surgery. Flow cytometry results showed age and sex dependent changes in circulating myeloid and lymphoid cell numbers, where the impact of nerve injury is minor. We found that in male mice with age, there is a significant increase of monocytes, CD8 T, and B cells, and a decrease of NK and CD4 T cells. We found that in female mice with age, while monocytes significantly increase, NK, CD4 T cells, CD8 T cells, and B cells all significantly decrease. Proportionally, monocyte and neutrophil compartments expand in aging female mice but remained stable in aging male mice. Both aging and injury affect the cytokine/chemokine patterns. Unexpectedly, MSCE treatment was not able to alleviate either mechanical or cold allodynia in both male and female aging SNI mice.

**Discussion/Conclusions**: We characterized age- and sex-dependent changes of systemic immune profile in mice, which is barely affected by nerve injury. Further investigation into inflammaging will help us to better understand the mechanisms of chronic pain in the aging population and to develop effective pain management strategies.

**Asymmetry of pain-induced facial grimacing**

Alicia S. Zumbusch, Paula Sanchez, Lilian Yoffe, Susansa Sotocinal and Jeffrey S. Mogil

McGill University

**Introduction/Aim**: Facial expressions are evolutionarily adaptive for visually communicating an organism’s state to others in its surroundings. Research shows that facial expressions relating to various experiences (e.g., fear, pleasure, etc.) in humans, non-human primates and other non-human animals are lateralized. That is, emotional expression is asymmetrical both in the observable facial output and in the neural structures and circuitry associated that govern their expression. To our knowledge, whether pain is similarly lateralized has never been assessed.

**Objective**: We sought to investigate whether facial expression of pain as measured by the Mouse Grimace Scale (MGS) is also lateralized and whether the pain model used alters the lateralization of grimacing. 

**Methods**: Mice were given intraperitoneal injections of acetic acid (AA) or a unilateral injection of complete Freund’s adjuvant (CFA) into the hind paw, and facial grimacing was recorded on video. Symmetrical still captures of the mouse face were sampled every 2 minutes and were mirrored about the y-axis to create left-left and right-right facial chimeras. We hypothesized that grimacing would be lateralized such that the left side of the face would yield higher MGS scores than the right side.  

**Results**: Mice experiencing non-localized reflexive pain induced by AA had higher grimace scores on the left hemiface than on the right. However, when the pain was localized to the left hind paw in the CFA model, grimacing was stronger on the ipsilateral (right) side of the face. 

**Discussion/Conclusions**: Future research will assess the neurobiological substrate of hemifacial asymmetry of pain-induced facial grimacing by unilaterally inhibiting areas known to be associated with pain expression such as the insula and amygdala.  

**Basic Science La science fondamentale Early-life microbiota colonization programs nociceptor excitability and pain sensitivity via mast cell-derived NGF**

Nasser S. Abdullah, Amyaouch Bradaia, Manon Defaye, Christina Ohland, Kristofer Svendsen, Ahmed Hassan, Mircea Iftinca, Kathy D. McCoy and Christophe Altier

University of Calgary

**Introduction/Aim**: The microbiota regulates the development of the nervous system, affecting sensory functions and host behavior. Specification of pain-sensing neurons (nociceptors) coincides with microbial colonization and early-life dysbiosis can lead to nociceptor hypersensitivity in adulthood. However, the mechanism by which the microbiota governs nociceptor development and pain sensitivity remains unknown. We investigated the impact of microbial colonization on nociceptors during early-life and on pain sensitivity in adulthood.

**Methods**: We generated germ-free (GF)-TRPV1GFP mice to gain insights into the transcriptional and functional changes of TRPV1+ nociceptors in the absence of a microbiota. GF-TRPV1GFP mice, GF-TRPV1GFP mice colonized with a complex microbiota at weaning (P24), and gnotobiotic-TRPV1GFP mice colonized from birth with a defined microbial consortia consisting of 12 bacterial species (OligoMM12) were analysed to investigate how microbes mediate neuronal modulation and to determine the critical developmental window of the phenotypic changes.

**Results**: GF mice show less pain responses to chemical irritants (capsaicin, formalin) and noxious heat than colonized mice. GF mice exhibited reduced nociceptor excitability and TRPV1 trafficking to the plasma membrane. Strikingly, colonization with a complex microbiota at weaning could not restore pain responses and nociceptor sensitivity. In contrast, colonization with OligoMM12 at birth was sufficient to restore neuronal perturbations. Mechanistically, the absence of microbiota reduced NGF in mast cells while colonization regulated NGF signaling on nociceptors.

**Discussion/Conclusions**: Bacterial composition during early-life regulates nociceptor activity and pain sensitivity in adulthood. Microbiota-mediated modulation of NGF release from mast cells at birth may shape nociceptor development, activity and ultimately pain sensitivity in adulthood.

**Biophysical characterization of a CaV3.1 channel mutation linked to trigeminal neuralgia**

Abdulaziz Alaklabi^a^, Eder Gambeta^b^ and Gerald Zamponi^b^

^a^Alberta Children’s Hospital Research Institute and Hotchkiss Brain Institute, Cumming School of Medicine, University of Calgary, College of Medicine, Alfaisal University, Riyadh, Saudi Arabia; ^b^Alberta Children’s Hospital Research Institute and Hotchkiss Brain Institute, Cumming School of Medicine, University of Calgary

**Introduction/Aim**: Trigeminal neuralgia is a debilitating disorder that affects one or more branches of the trigeminal nerve. This condition leads to severe pain attacks and a poor quality of life. Previous findings demonstrated that CaV3.1 calcium channels might play an important role in trigeminal pain. A recent study reported a new missense variant in the CACNA1G gene that encodes the pore forming α1 subunit of the T-type CaV3.1 calcium channel. The mutation is located in the I-II linker region of the channel and leads to a substitution of an Arginine (R) by a Glutamine (Q) at position 706.

**Methods**: We used whole-cell voltage-clamp recordings to assess the current-voltage (IV) relations, responses to action-potential waveforms, voltage-dependence of inactivation and recovery from inactivation of wild-type CaV3.1 and R706Q mutant channels expressed in tsA-201 cells.

**Results**: Our data reveal a gain-of-function in current density in the R706Q mutant, without changes in the voltage for half activation. Moreover, voltage clamp using an action potential waveform revealed an increase in the tail current in the R706Q mutant. No changes were observed in the steady-state inactivation curves. In addition, the R706Q mutant exhibited a faster recovery from inactivation.

**Discussion/Conclusions**: Taken together, the gain-of-function effects in the R706Q CaV3.1 mutant have the propensity to impact trigeminal pain transmission, consistent with a contribution to trigeminal neuralgia pathophysiology.

**The role of Slc7a5 (Lat1) in rodent and human nociceptors**

Sascha Alles^a^, Reza Ehsanian^a^, Aleyah Goins^a^, Kimberly Gomez^b^, Mark Shilling^a^, Ian Adams^a^, Elizabeth Solomon^a^, Mitra Afaghpour-Becklund^a^ and Rajesh Khanna^b^

^a^University of New Mexico School of Medicine; ^b^New York University

**Introduction/Aim**: Globally, 1 in 5 people suffer from high-impact chronic pain. More effective, non-addictive, non-opioid therapeutics are urgently needed. System L-neutral amino acid transporter (Slc7a5) also known as L-type amino acid transporter 1 (Lat1) is involved in several physiological processes related to inflammation. Transcriptomics studies in mice and humans have shown that Slc7a5 and its binding partner glycoprotein CD98 (Slc3a2) are expressed in neurons of the dorsal root ganglia (DRG) and spinal dorsal horn, which are critical to the initiation and maintenance of nociception and pathophysiology of chronic pain.

**Methods**: Behavioral studies were performed in rodents with the spared nerve injury (SNI) model of neuropathic pain. Human and rodent DRG and spinal cord tissues were analyzed using whole-cell electrophysiology, immunohistochemistry, qPCR and Western blot methods as described in our previous work.

**Results**: Our data show that blocking Slc7a5 with intrathecal administration of the Slc7a5 antagonist JPH203 alleviates allodynia in SNI rodents. Western blot studies show an increase in Slc7a5 protein levels in the spinal cord of SNI mice compared to controls. Whole-cell electrophysiology from cultured DRG neurons from mice and humans demonstrate that the excitability of these neurons was reduced following JPH203 treatment. JPH203 reduces peak tetrodotoxin-resistant (TTX-R) sodium current recorded from naïve rat DRG neurons.

**Discussion/Conclusions**: These results demonstrate that Slc7a5 is dysregulated in chronic neuropathic pain and can be targeted to provide relief of hypersensitivity. Future work will determine Slc7a5ʹs mechanism of action in different models of chronic pain using translationally-relevant human DRG and spinal cord tissues.

**Visuotactile integration in individuals with fibromyalgia**

Tania Augière, Martin Simoneau and Catherine Mercier

Center for Interdisciplinary Research in Rehabilitation and Social Integration (Cirris); Laval University

**Introduction/Aim**: The integration of various sources of sensory information improves the precision of our perception and is essential to generate a unified representation of our body. Individuals with fibromyalgia often experience somatosensory alterations and distortions of their body representation, suggesting that integration of somatosensory information may be altered. This study evaluates the capacity of individuals with fibromyalgia to integrate visuotactile information.

**Methods**: Pairs of stimulations were applied to the thumb and index (inter-stimuli intervals between 0 and 800 ms) and participants had to judge which finger was stimulated first (two-alternative forced choice). Stimuli were either tactile (non-painful electrical pulses), visual (diodes), or visuotactile (simultaneous tactile and visual stimuli). Two variables were derived from psychophysics curves: the standard deviation, indicating the precision of the perception, and the weight attributed to each sensory signal in the visuotactile percept.

**Results**: Ten participants with fibromyalgia and eight pain-free controls were recruited. Results showed an improvement of precision in the Visuotactile condition compared to the Visual or Tactile conditions, for both groups. Moreover, even though tactile information was more precise, it contributed slightly less to the visuotactile percept (i.e., less weight) than visual information; this difference was more pronounced in participants with fibromyalgia (35% vs 45% for the controls).

**Discussion/Conclusions**: Preliminary results suggest a perceptual benefit of having access to several sources of information, which is a sign of an optimal multisensory integration, in participants with fibromyalgia. However, the weighting of information seems more altered in individuals with fibromyalgia, possibly because somatosensory information is considered as less reliable.

**Characterization of the effect of carbamazepine in a model of trigeminal neuralgia in male and female rates**

Darciane Baggio, Fernanda da Luz, Julia Zortea, Vanessa Lejeune and Juliana Chichorro

UFPR - Federal University of Paraná

**Introduction/Aim**: Trigeminal neuralgia (TN) is a severe form of neuropathic pain characterized by unilateral and recurrent pain attacks in the area innervated by the trigeminal nerve. Carbamazepine (CBZ) is the drug of choice to control pain paroxysms, but its efficacy on other aspects of TN lacks characterization. Additionally, little is known about sex differences in the efficacy of CBZ in TN, which affects predominantly the female sex.

**Methods**: The chronic constriction injury of the infraorbital nerve (CCI-ION) was used as a TN model. The influence of CBZ (10 and 30 mg/kg) in male and female rats were tested on day 15 after CCI-ION in several parameters: facial mechanical allodynia (Von Frey filaments); spontaneous nociception (grooming time); affective/motivational pain component (conditioned place preference, CPP; emission of ultrasonic vocalization, USV), and anxiety-like behaviour (Elevated plus maze, EPM).

**Results**: CBZ reduced mechanical allodynia and grooming behaviour in female and male CCI-ION rats, but it induced CCP only in CCI-ION female rats. CCI-ION promoted a reduction in the emission of 50 kHz USV in male and female rats, but CBZ treatment restored it only in males. Anxiety-like behaviour was only detected in male rats, but CBZ had no anxiolytic effect. Plasma concentration of CBZ 1 hour after treatment was significantly higher in females than males.

**Discussion/Conclusions**: These results contribute to broadening the characterization of the CCI-ION model in male and female rats and provide additional evidence of the efficacy of CBZ in parameters that enhance the translational value of the model.

**Investigating the function of polymorphisms in APOE in chronic pain**

Nicole Brown, Shannon Tansley, Alba Ureña Guzmán, Marc Parisien, Luda Diatchenko and Arkady Khoutorsky

McGill University

**Introduction/Aim**: Activation of microglia in the spinal cord following peripheral nerve injury is critical for the development of long-lasting pain hypersensitivity. Single-cell RNA sequencing of isolated microglia revealed that Apolipoprotein E (Apoe) is the top upregulated gene in spinal cord microglia at chronic time points after peripheral nerve injury in mice. APOE is a lipoprotein that is essential for the regulation of neuroimmune functions, synaptic activity, and aging. In humans, there are 3 different isoforms of APOE: APOE-ε2, APOE-ε3 and APOE-ε4. APOE-ε4 is the strongest genetic risk factor for the development of Alzheimer’s disease. Previously we have shown that carriers with APOE-ε2 have significantly higher risk of developing chronic pain, whereas carriers of APOE-ε4 have lower risk.

**Methods**: To test the functional role of ApoE polymorphisms in chronic pain, we used humanized mice expressing APOE-ε2, APOE-ε3 and APOE-ε4, and implemented four models of chronic pain: spared nerve injury (SNI), Complete Freund’s Adjuvant (CFA), hyperalgesic priming, and chronic constriction injury (CCI). To determine pain behaviors, mice were tested in von Frey, radiant paw withdrawal, mouse grimace scale, hot plate, and cold plate tests.

**Results**: Behavioral testing conducted at baseline revealed an increase in cold sensitivity in APOE-ε2 mice. Following behavioral testing in mice with SNI, APOE-ε4 mice showed a decrease in nerve-injury induced cold hypersensitivity.

**Discussion/Conclusions**: Our results support epidemiological studies in humans as they show that APOE-ε2 promotes hypersensitivity whereas APOE-ε4 is protective against developing chronic pain. Altogether, these studies might facilitate better diagnosis and treatment of individuals living with different chronic pain conditions.

**Test stimulus intensity-dependent conditioned pain modulation**

Laila Chaudhry^a^, Isabel Aboud^a^, Mathilde Ferland^a^, Simon Carrier^b^, Marc O. Martel^a^ and Jeffrey Mogil^a^

^a^McGill University; ^b^Universite de Montreal

**Introduction/Aim**: Conditioned pain modulation (CPM) is a well-known phenomenon whereby pain in one location inhibits pain in another. Previous mouse/human data from our lab (Tansley et al., 2019; Chaudhry et al., unpublished data) demonstrates that test stimulus intensity affects CPM’s direction, with higher-intensity stimuli leading to hypoalgesia (i.e., CPM) and lower-intensity stimuli leading to hyperalgesia (i.e., “anti-CPM”). McPhee & Graven-Nielsen (2022) recently reported that positive affective manipulations increased CPM and negative affect decreased it. We aimed to see how affect valence and test stimulus intensity interact to affect CPM.

**Methods**: Participants (N=60) underwent an individual heat pain threshold (HPTh) assessment, followed by three CPM trials, at -1, +1, or +3°C below/above their HPTh, while viewing photos from the International Affective Picture System, administered in three counterbalanced affective valence groups: positive, negative, and neutral. Each CPM trial consisted of two baseline sub/suprathreshold heat pain stimulations, a 30-s cold pressor test (4°C; the conditioning stimulus in the CPM paradigm), and a final heat pain stimulation at the same temperature, with pain ratings (11-point NRS and 0–100 VAS) provided throughout.

**Results**: A two-way repeated measures ANOVA was conducted to assess differences in pain ratings from pre- to post-cold pressor in all test stimulus intensity and affect groups. Preliminary results indicate a trend in which negative valence produced hyperalgesia when compared to positive valence in the +3°C and -1°C conditions.

**Discussion/Conclusions**: Thus, negative affect may impair both CPM and anti-CPM, emphasizing the importance of both test stimulus intensity and affect in the conduct of CPM experiments.

Effect of various preprocessing techniques in developing different machine learning techniques to predict chronic pain.

**RONRICK DA-ANO, Matthew Fillingim, Azin Zare, Jax Norman, Gianluca Guglietti, Christophe**

Tanguay Sabourin and Etienne Vachon Presseau

McGill University

**Introduction/Aim**: The majority of functional magnetic resonance imaging (fMRI) investigations conducted to date have used the assumption that there is consistent functional connectivity (FC) between time series from different brain areas. Interestingly, there has recently been a rise in interest in quantifying potential dynamic changes in FC during fMRI investigations as it is believed that doing so may shed light on the basic operation of brain networks. However, a growing body of research in neuroimaging suggests that functional networks show dynamic changes in connection strength as well as variable phase difference (nonzero time-lag) between regions. Our goal is to compare various preprocessing techniques that addresses these problems and demonstrate their effect in developing and evaluating machine learning (ML) in predicting chronic pain.

**Methods**: A resting state functional MRI (rsfMRI) data with pain status were obtained from UK BioBank (UKBB) dataset. The brain was parcellated utilizing an adjusted Brainettome atlast of 279 regions of interest after various preprocessing techniques: i) Dynamic Conditional Correlation (DCC), ii) Dynamic Time Warping (DTW), iii) Tangent Correlation, iv) Partial Correlation and iv) Pearson Correlation. After splitting the data 67/33% in training and testing sets, models to predict chronic pain and sex were built using 6 ML pipelines: i) Logistic Regression (LR), ii) XGBoost (XGB), iii) LightGBM (GBM), iv) K-Nearest Neighbors (KNN), iv) Random Forest (RF) and iv) AdaBoost. All 6 pipelines used either the functional connectivity matrix only or with relevant covariates. They were compared using Area Under the Curve (AUC).

**Results**: In discriminating sex, Partial and Pearson correlations provided a consistent performance when using in 6 ML algorithm (AUC: 0.88, 0.85, 0.87, 0.87, 0.87, 0.87 and 0.93, 0.65, 0.88, 0.85, 0.80, 0.74 respectively). On the other hand, in predicting chronic pain, DCC and Pearson correlation provided a relatively better performance when using in 6 ML algorithm (AUC: 0.53, 0.53, 0.54, 0.54, 0.54, 0.52 and 0.55, 0.51, 0.53, 0.53, 0.55, 0.53 respectively).

**Discussion/Conclusions**: The 5 various preprocessing techniques affect the performance on whatever ML algorithms are performed in predicting either chronic pain or sex. However, the 6 different ML pipelines were consistent in the performance of predictive brain-based biomarker models.

**People with chronic pain who report greater pain interference and social invalidation are more likely to experience increased shame**

Nina Gregoire^a^, Alanna Coady^a^, Kimberley Kaseweter^b^, Susan Holtzman^a^

^a^University of British Columbia; ^b^UBC

**Introduction/Aim**: Chronic pain can significantly interfere with people’s ability to fully engage in daily activities. People with pain are also susceptible to stigmatizing social responses, particularly invalidation, which may take the form of discounting (i.e., disbelief/discrediting the legitimacy of pain) or lack of understanding (i.e., failing to respond empathetically to pain). People who struggle with pain, pain interference, and social stigma may internalize negative social judgments by negatively judging themselves. The present study investigated whether pain interference and pain severity, discounting, and lack of understanding were associated with an increased risk of pain-related shame.

**Methods**: Participants (N=305) were recruited from a pool of patients at a specialized pain clinic in British Columbia and asked to complete a cross-sectional survey online. The survey included the Brief Pain Inventory, the Illness Invalidation Inventory, and the Chronic Illness Related Shame Scale to measure pain interference and severity, discounting and lack of understanding, and pain-related shame respectively.

**Results**: Results found that pain interference (Beta=.33), lack of understanding (Beta=.21), and discounting (Beta=.34) were all significant and positive predictors of shame (all p’s < .001), while pain severity was not (Beta=-.11, p=.83). Together, the three variables explained 47% of the variance in shame.

**Discussion/Conclusions**: These findings suggest that the social stigma of pain can have an even more powerful impact on emotional functioning than the pain itself. Further, these findings highlight the vital importance of reducing stigmatizing attitudes toward chronic pain at the societal level. Moreover, focused interventions to reduce feelings of shame in response to pain-related challenges and interference may be needed.

**Differential regulation of Cav3.2 and Cav2.2 calcium channels by CB1 receptors and cannabidiol**

Erika K. Harding, Ivana A. Souza, Maria A. Gandini, Vinícius M. Gadotti, Md Yousof Ali, Sun Huang, Flavia T. T. Antunes, Tuan Trang and Gerald W. Zamponi

University of Calgary

**Introduction/Aim**: Cannabinoids represent a promising therapeutic avenue for chronic pain treatment, however mixed clinical and preclinical trial results indicate more work is needed to define the precise mechanism of action of cannabinoids in producing analgesia. Presynaptic terminals of nociceptive primary afferent neurons contain a unique complement of Cav2.2 and Cav3.2 voltage-gated calcium channels. Here, we determined whether two different cannabinoids, HU-210 and Cannabidiol (CBD) produced differential inhibition of these two channels, defining a novel mechanism through which CBD can produce analgesia in vivo.

**Methods**:Calcium currents were recorded from tsA-201 cells expressing Cav3.2 or Cav2.2 channels alone, or in combination with Cannabinoid Receptor 1 (CB1R). The analgesic effects of CBD in C57Bl/6 and Cav3.2 knockout mice were assessed in the formalin, Complete Freund’s Adjuvant (CFA), and Partial Sciatic Nerve Ligation (PSNL) models of inflammatory and neuropathic pain.

**Results**: HU-210 inhibited Cav2.2, but not Cav3.2 calcium currents through a CB1R dependent mechanism. Conversely, CBD had no effect on Cav2.2, but instead inhibited Cav3.2 calcium currents independent of CB1R. In all three pain models, intrathecal administration of CBD produced analgesia. In Cav3.2 knockout mice with PSNL, we found that the effect of CBD was significantly diminished.

**Discussion/Conclusions**: Altogether, our data indicate that while HU-210 produces inhibition of Cav2.2 through a canonical CB1R dependent mechanism, CBD produces inhibition of Cav3.2 through direct inhibition of the channel. Our results also indicate a dependence on Cav3.2 for CBD to produce analgesia. These findings advance our understanding of the potential utility of cannabinoids like CBD in treating chronic pain conditions.

**Psychosocial predictors of parent physiological response to their child in pain**

Sara Jasim^a^, Dan Flanders^b^, Eitan Weinberg^b^, Hartley Garfield^b^, Deena Savlov^b^ and Rebecca Pillai Riddell^c^

^a^York University; ^b^University of Toronto; ^c^York University, University of Toronto

**Introduction/Aim**: According to the polyvagal theory (Porges, 2007), autonomic functions, such as respiratory sinus arrythmia (RSA), are strongly associated with self-regulation, such that higher resting RSA is associated with adaptive emotion regulation. Further, previous research suggests that social factors (e.g., education; Singh & Shankar, 2013) and cultural stressors (e.g., acculturation; Antoniadou & Quinlan, 2020) differentially impact emotion regulation. This study aimed to examine the influence of social predictors on parental RSA when their child was undergoing an acute painful procedure (vaccination).

**Methods**: Parents were observed while holding their toddler during a routine vaccination in their pediatrician’s office (N=132 caregivers). Caregiver RSA measures were collected during baseline (pre-needle) and for three-minutes post needle. Caregiver education and acculturation data were collected via self-report prior to the appointment.

**Results**: Caregiver and spousal acculturation positively and negatively predicted baseline RSA, respectively. After controlling for baseline RSA, education and acculturation did not predict RSA levels post-needle.

**Discussion/Conclusions**: Caregiver regulation at baseline significantly related to acculturation and post-needle regulation while no other relationships between education and acculturation post-needle were found. These findings suggest that caregiver RSA prior to their child’s vaccination appointment sets the stage for how they will approach their child in pain post-needle. Previous research with parents of a child in acute pain (Waxman et al., 2020) noted that child pre-needle RSA significantly predicted post-needle RSA, suggesting the most influential time to support parents to support their children could be prior to the needle occurring. Generalizability is limited given the higher education level of the sample.

**Spinal cord meningeal macrophages exert an analgesic effect after skin incision in a sex-dependent manner**

Mahshad Kolahdouzan^a^, YuShan Tu^b^, Milind Muley^b^ and Michael Salter^a^

^a^The Hospital for Sick Children and University of Toronto; ^b^The Hospital for Sick Children

**Introduction/Aim**: Meningeal immune cells are increasingly implicated in health and disease. Whether meningeal immune cells play a role in pain is unclear. Here we investigated whether meningeal immune cells in the spinal cord regulate pain hypersensitivity.

**Methods**: In all experiments we used male and female Sprague-Dawley rats. Animals were anaesthetized with isoflurane and were randomly assigned to one of two groups: i) anaesthesia only controls or ii) animals receiving a skin incision (SI) on the lateral left thigh. Spinal meningeal macrophages were selectively depleted, where indicated, by intrathecal injection of an antibody to the macrophage cell-surface receptor CD206 (anti-CD206) conjugated to the toxin saporin. We evaluated mechanical sensitivity using von Frey assay on post-operative day 3 before injection (POD3b), and 4h and 24h after injection.

**Results**: In naïve rats, paw withdrawal threshold (PWT) did not change after intrathecal injection of anti-CD206, or vehicle control, in either males or females. Following SI, PWT decreased in male rats after injecting anti-CD206 (4h vs. POD3b, p < 0.05, n=23, and 24h vs. POD3b, p < 0.01, n=23). By contrast, following SI, PWT did not change in vehicle-injected male rats (4h vs. POD3b, p > 0.05, n=16). We observed no change in PWT in females after CD206-saporin injection (n=23) or vehicle (n=19).

**Discussion/Conclusions**: We interpret these findings as indicating that in males after SI, but not in naïve animals, depleting spinal meningeal macrophages revealed an underlying analgesic effect of these cells. In females, the lack of effect of meningeal macrophage depletion indicates that the analgesic effect after SI is sex-dependent.

**Changes to cerebellar gene expression following brain trauma, surgery, and early life adversity in rats: A pathway to pain?**

Crystal Li, Sabrina Salberg, Glenn Yamakawa and Richelle Mychasiuk

Monash University

**Introduction/Aim**: Post-traumatic and post-surgical chronic pain manifests frequently in adolescents. The mechanisms underlying the development of chronic pain are not well understood, but early life adversity (ELA) is a common risk factor. Given that cerebellar function may be modulated by ELA and pain, we explored pain outcomes and cerebellar gene expression related to neurotransmission following ELA, mild traumatic brain injury (mTBI), or plantar incision surgery (PIS).

**Methods**: Male and female Sprague Dawley pups underwent a maternal separation (MS) paradigm or were left undisturbed during development. At adolescence, rats were assigned to mTBI, PIS, or sham injury. Von Frey and hot-cold plate examined changes in mechanical and thermal pain sensitivity, respectively. Cerebellum tissue was processed for RT-qPCR analysis of the following genes: BDNF, GABRA1, MAOA, GR, MR, and IBA1.

**Results**: On the von Frey test, the hind paw withdrawal response was reduced following mTBI and PIS (p < 0.001), but did not differ between MS and control groups (p > 0.05). On the hot plate, PIS groups displayed longer latencies to react (p < 0.05). Cerebellar gene expression of GR, GABRA1, and MAOA were significantly different between injury groups (p’s < 0.05), while cerebellar MR expression was reduced in MS groups relative to controls (p < 0.05).

**Discussion/Conclusions**: mTBI and PIS are associated with increased sensitivity to mechanical stimuli and reduced sensitivity to thermal stimuli. Cerebellar changes to neurotransmission and stress reactivity occurred in response to mTBI, PIS, and ELA. These changes may contribute to an increased risk for chronic pain and future investigations should explore the specific cerebellar mechanisms involved.

**Circadian rhythm of chronic pain in a mouse model of multiple sclerosis**

Vina Li, Julia Segal, Mitra Knezic and Nader Ghasemlou

Queen’s University

**Introduction/Aim**: Multiple sclerosis (MS) is a chronic demyelinating disease of the central nervous system, impacting 1 in 400 Canadians and 2.8 million people worldwide. Over 50% of people living with MS (pwMS) experience chronic pain, with its intensity often following a rhythm throughout the day. However, the underlying mechanisms of these rhythms remain unknown. We sought to characterize the circadian rhythmicity of pain and neuroinflammation using an experimental autoimmune encephalomyelitis (EAE) model.

**Methods**: C57BL/6 female mice (7-12 weeks old) were induced with EAE or sham. Pain outcomes were assessed 4 times a day at 7, 10, 14, 21 and 28 days post-immunization (dpi) using the von Frey mechanical, Hargreaves radiant heat, and acetone cold tests. Flow cytometry and qRT-PCR were used to characterize neuroinflammation in the lumbar spinal cord. Clock gene expression patterns were also assessed in the spinal cord using qRT-PCR.

**Results**: We observed mechanical and cold hypersensitivity, but not thermal hypersensitivity in EAE mice. The greatest mechanical sensitivity was observed at ZT8 from 7dpi to the chronic phase of disease. At peak disease, pro-inflammatory cytokines exhibited a pronounced circadian pattern, with the trough observed at ZT8. Immune cell migration and clock gene expression patterns both deviated from their naïve rhythms.

**Discussion/Conclusions**: Our data shows, for the first time, that pain follows a circadian rhythm in EAE and that this rhythm may be regulated by neuroimmune responses and clock gene expressions. These results will help inform future research on the significance of circadian rhythms in MS pathophysiology and the potential development of chronotherapeutics.

**Spinal cell type-specific mRNA translational control mechanisms in the development of chronic pain**

Kevin Lister, Calvin Wong, Patricia Stecum, Jieyi Yang, Sonali Uttam, Mehdi Hooshmandi, Shannon Tansley, Noosha Yousefpour, Weihua Cai, Jeffrey Mogil, Arkady Khoutorsky

McGill University

**Introduction/Aim**: Sensitization of the pain pathway requires new gene expression to support structural and biochemical changes; however, the mechanisms controlling it are not fully identified. Recent studies suggest that gene expression in pain pathways following peripheral injury is regulated at the level of protein synthesis.

**Methods**: Characterization of protein synthesis in the spinal cord. Nascent protein synthesis in the spinal cord after peripheral nerve injury was assessed using Fluorescent Non–Canonical Amino acid Tagging (FUNCAT). In FUNCAT, non-canonical amino acid azidohomoalanine (AHA) is taken up by cells and charged onto methionine tRNAs. During translation, AHA is incorporated into newly synthesized proteins and is visualized using click chemistry. In combination with FUNCAT, we performed immunohistochemistry to identify translation in distinct cell types.

Upregulation of translation in specific cell types. To upregulate translation in specific cell types, we deleted translational repressor 4E-BP1 in excitatory and inhibitory neurons, and microglia.

**Results**: Visualizing de novo protein synthesis using FUNCAT in the spinal cord four days after peripheral nerve injury revealed a significant increase in general protein synthesis in inhibitory neurons and microglia. Deletion of the translational repressor, 4E-BP1, in microglia and inhibitory, but not excitatory neurons, caused mechanical but not thermal hypersensitivity.

**Discussion/Conclusions**: Our results show that protein synthesis is increased in inhibitory neurons and microglia following peripheral nerve injury. Additionally, ablation of 4E-BP1 is sufficient to induce modality-specific hypersensitivity. These results advance our understanding of protein synthesis in mediating sensitization of spinal nociceptive circuits and their contribution to the identification of novel therapeutic targets to alleviate pain.

**A transcriptomic approach to understanding pain signals in the brain and spinal cord**

Ryan Loke and John Kramer

University of British Columbia

**Introduction/Aim**: Chronic pain affects one in five Canadians and leads to over $40 billion in costs to the Canadian Healthcare System each year. Due to the variable and inadequate experiences in treating chronic pain with current medications, there is a need to develop better pain management solutions. One major barrier preventing the development of new analgesics is our lack of knowledge of the complex molecular mechanisms and organization of the pain sensory pathways. Quantitative gene expression analysis can help identify patterns in genes associated with pain sensory pathways and comparative analysis can compare novel gene lists against prior knowledge gene-set libraries to extrapolate functional association and predict biological processes. The goal of this study is to use a transcriptomic approach to understand the molecular structure and mechanisms of pain signaling in the central nervous system.

**Methods**: Gene enrichment analysis will be conducted using HPAanalyze, an R package for exploratory data analysis from the Human Protein Atlas. Looking specifically for upregulated genes in the dorsal horn, results can be filtered and compared using other databases like DisGeNET and Enrichr to determine functional, pathway, and expression associations.

**Results**: Preliminary results for gene analysis resulted in a list of dorsal horn enriched genes involved in somatosensory signaling. After comparative analysis, we anticipate a novel list of genes involved in pain signaling pathways from the spinal cord to regions of the brain.

**Discussion/Conclusions**: This quantitative gene expression analysis study alongside comparative analysis will provide insight into the molecular organization and mechanisms of nociceptive signaling in the nervous system.

**Visual exposure to green light ameliorates mechanical pain in a rat model of osteoarthritis**

Jason McDougall, Chris DeBow and Melissa O’Brien

Dalhousie University

**Introduction/Aim**: The use of non-pharmacological approaches to treat osteoarthritis (OA) pain offers a promising means of safely alleviating discomfort. Visual exposure to green ambient light has been shown to reduce chronic pain in migraine and fibromyalgia patients. This study investigated the effect of green light therapy (GLT) on joint pain and nociceptor activity in a rat model of OA. The role of the endocannabinoid system was also examined.

**Methods**: Male and female Wistar rats (200-320g) received an intra-articular injection of sodium monoiodoacetate (MIA; 3mg/50μl) to induce unilateral knee OA. Nine days following model induction, rats were exposed to ambient green light (wavelength: 455 nm; luminance: 20Lux) for 5 days (8hr/day). Control animals were exposed to white light. Tactile mechanosensitivity was assessed by von Frey hair algesiometry, and any changes in peripheral sensitization was determined by electrophysiologically recording from joint nociceptors. The role of the endocannabinoid system was investigated by treating GLT animals with the CB1 receptor antagonist, AM281 (500ug/kg i.p. in DMSO:cremaphor:saline, 1:1:8).

**Results**: Secondary mechanical allodynia was significantly reduced in both male and female rats treated with GLT compared to control white light (p < 0.0001, n=8, 2-way RMANOVA). Joint nociceptor activity was unaffected by GLT in either sex (p > 0.05, n=6-10, 1-way ANOVA). Blockade of CB1 receptors with AM281 significantly attenuated GLT-induced analgesia (p < 0.0001, n=8).

**Discussion/Conclusions**: Frequent exposure to GLT reduced experimental OA pain by activating the endocannabinoid system. These effects occurred centrally since joint nociceptor activity was unaffected by the treatment. Confirmation of these effects in humans warrants further investigation.

**Circadian rhythmicity of painful injury influences postoperative pain and recovery**

Eleri McEachern^a^, Nader Ghasemlou^a^, Jeffrey Mogil^a^

^a^McGill University and ^b^Queen’s University

**Introduction/Aim**: Postoperative pain is a major concern for patients worldwide which can last days, weeks, or even months following surgery. Circadian rhythms, controlled by molecular clocks, govern nearly every physiological function. Biological clocks are found throughout the body and reside in most cells including those of the immune system. Chronobiological approaches have emerged as a tool to study regulation of pain and inflammation. While time-of-day effects on pain levels of various pain conditions have been well studied, it remains poorly understood whether time of injury can alter subsequent pain behaviours.

**Methods**: The primary aim of this study is to assess postoperative pain in a mouse model of hind paw incision in a circadian-dependent manner. Incisions were made at four time points (03:00 h, 09:00 h, 13:00 h, 21:00 h). Evoked pain behaviours were measured using the von Frey mechanical sensitivity and Hargreaves radiant heat paw-withdrawal assays. Testing was performed 1, 3, 5, 7, and 10 days following incision, at the same time every day, then once weekly until pain resolution.

**Results**: Zeitgeber time (ZT) is used to standardize time based on individual laboratory light:dark cycles, where ZT0 corresponds to lights-on and ZT12 for lights-off. No statistically significant differences were observed for mechanical hypersensitivity between surgical groups (with the radiant heat test still ongoing). However, there appeared to be faster recovery in mice receiving surgery at ZT2/8 compared to ZT14/20, suggesting that pain from injuries occurring during the rest phase may resolve more rapidly than pain from injuries occurring during the active phase.

**Discussion/Conclusions**: Mice display robust circadian oscillation in clock-controlled gene expression, with Per1/2 and Reverb-a peaking in the dorsal root ganglion around the middle of the light phase, and Bmal1, Clock, and Npas2 in antiphase. Thus, ZT2 corresponds with low levels of Per1/2 and peak of Bmal1 and Clock expression. There is therefore likely a circadian effect regulating postoperative pain due to time of wounding, holding therapeutic potential for more biologically-informed operative and postoperative care.

**A predictive model for jaw pain evoked by a repeated clenching task based on masseter muscle tissue oxygen saturation**

Pedram Mouseli^a^, Suha Sagheer^a^, Darlene Reid^b^, Massieh Moayedi^a^ and Iacopo Cioffi^a^

^a^University of Toronto; ^b^University of Toronto, KITE - Toronto Rehab-University Health Network

**Introduction/Aim**: Oxygen tissue saturation (StO2) can assess abnormalities in skeletal muscle oxy/deoxygenation. During experimental jaw clenching exercises, StO2 monitoring can inform about potential impairment to blood flow regulation in the masticatory muscles. Here, we aimed to determine whether masseter StO2 predicts jaw muscle pain evoked by repetitive clenching.

**Methods**: 29 healthy participants (15F, 14M, 24.4 ± 8.5 years) consented to procedures approved by the University of Toronto Research Ethics Board. Participants performed fifteen 1min sustained jaw clenching trials, targeting ~30% of their maximum voluntary contraction (MVC), using visual EMG feedback. Next, participants clenched at their MVC until failure. Muscle pain ratings were collected every minute using visual analog scales. StO2 trajectories of the right masseter were collected using near-infrared spectroscopy and analyzed. A line was fitted to the difference of StO2 medians during clench and rest periods (15 trials). The slope of the fitted line and its sign (+/-), the sign of intercept, the number of times that the difference vector crosses zero, and the participants’ sex were used as features in a predictive model. A Lasso model with cross-validation was used to predict muscle pain ratings after the MVC session and the trend of reported pain from first to last trial.

**Results**: The proposed model including features of StO2 trajectories successfully predicted both post-MVC muscle pain (r=0.45, p=0.01) and pain trends (r=0.42, p=0.02).

**Discussion/Conclusions**: Pain evoked by repeated clenching can be predicted by features extracted from the masseter’s oxygenation patterns. It is feasible to implement this model in future studies evaluating predictors of temporomandibular disorders.

**Alterations in gastrointestinal motility as a driver of opioid-induced microbial dysbiosis**

Julia ER Nickols^a^, Andrew J. Forgie^a^, Elizabeth Sugiarto^a^, Ethan Chen^a^, Zöe Dworsky-Fried^a^, Adrienne Dang^b^, Ben P. Willing^a^, Catherine Cahill^b^ and Anna M. W. Taylor^a^

^a^University of Alberta; ^b^University of California Los Angeles

**Introduction/Aim**: Adverse side effects, including tolerance, hyperalgesia, and addiction, are serious clinical challenges for chronic pain management and opioid deprescribing. Opioid receptors are expressed throughout the gastrointestinal (GI) tract, and opioid use both influences gut motility and alters the gut microbiome. The gut microbiome and immune system are intimately connected via a gut-brain signalling axis. Given that neuroinflammation contributes to many of the adverse effects of opioids, I hypothesize that alterations in gut motility associated with opioid withdrawal drive persistent shifts in the taxonomic profile of the gut microbiome, leading to the development of opioid-induced hyperalgesia.

**Methods**: Male and female C57Bl6J mice were treated with escalating doses of morphine (10-40 mg/kg, i.p.) for 4 days. Pain behaviour, tissue collection, and 16S rRNA sequencing of microbiome samples was performed 12 hours- 4 weeks after morphine treatment to assess acute and protracted withdrawal syndromes. Pharmacological strategies to increase (loperamide) or decrease (tegaserod) GI motility was used to interrogate the role of motility on dysbiosis and pain.

**Results**: Microbiome sequencing revealed that chronic opioids altered microbial diversity for up to 1-week post-treatment and correlated with hyperalgesia and neuroinflammation. Fecal microbiota transfer from opioid-withdrawn to drug-naïve mice was sufficient to reproduce these results. Symptoms of opioid dependence could be attenuated by normalization of gut motility, while increasing gut motility in opioid naïve mice was sufficient to produce hyperalgesia and neuroinflammation.

**Discussion/Conclusions**: These results indicate that strategies that target the gut microbiome or GI motility may improve clinical outcomes in prescribing and deprescribing opioids.

**Dorsal horn parvalbumin-expressing interneurons express functional small conductance calcium-activated potassium channels implicated in mechanical allodynia**

Haoyi Qiu, Lois Miraucourt, Albena Davidova and Reza Sharif-Naeini

McGill University

**Introduction/Aim**: The nervous system processes sensory information through the precise coordination of neuronal networks and their firing patterns. In the spinal cord, disturbances to the tonic firing ability of parvalbumin (PV)-expressing inhibitory interneurons (PVINs) lead to abnormalities in the integration of touch information and may result in mechanical allodynia. PV is a calcium (Ca2+)-binding protein that buffers Ca2+ accumulation in neurons after trains of action potential to allow for tonic firing. Here, we examined whether the small conductance calcium-activated potassium (SK) channel is implicated in the changes to PVINs firing after nerve injury.

**Methods**: We used mice expressing tdTomato under the control of the parvalbumin gene (PV::Cre;tdTom) and performed visually guided whole-cell recordings in spinal cord slices of naïve and nerve-injured mice in the presence of various SK channel pharmacological blockers (Lei-Dab7, apamin) and activators (1-EBIO). We performed QPCR and fluorescence in situ hybridization to examine the expression of SK channels in PVINs.

**Results**: Our results show that PVINs transition from tonic to adaptive firing after nerve injury in both male and female mice. Further, we found that the SK channel subtype 2 (SK2) was most predominant in PVINs. In naïve mice, activation of SK channels led PVINs to develop frequency adaptation. Conversely, in nerve injured mice, blocking SK2 channels partially restored the normal tonic firing of PVINs.

**Discussion/Conclusions**: Our data identifies an important contribution of SK channels to the firing activity of PVINs after nerve injury. Blocking these channels in PVINs may restore their normal tonic firing and decrease mechanical allodynia after nerve injury.

**Assessing the effect of genetic restriction of mGluR5 to intracellular membranes on the late phase of the formalin test in mice**

Roseanna Rought^a^, Heidi Erb^a^, Karen O’Malley^b^ and Terence Coderre^a^

^a^McGill University; ^b^Washington University School of Medicine

**Introduction/Aim**: Previous pharmacological studies have implicated intracellular metabotropic glutamate receptor 5 (mGluR5) in persistent pain. Cell permeable but not impermeable antagonists effectively reduce neuropathic and inflammatory injury-induced pain hypersensitivity, as well as pain-induced enhancements of spinal cord dorsal horn signalling. To overcome limitations of pharmacological manipulation, we used mice in which mGluR5 was genetically targeted to intracellular membranes (mGluR5IM mice) to determine changes in several measures of pain behaviour and downstream nociceptive signalling.

**Methods**: mGluR5IM mice were generated by inserting an endoplasmic reticulum/ nuclear membrane targeting motif from the Lamin-B receptor immediately upstream of the mGluR5 C-terminal stop codon using CRISPR. Loss of cell surface mGluR5 was validated anatomically and functionally. Here, mGluR5IM and mGluR5wild-type (WT) mice of both sexes were injected with 20 µl of 5% formalin, intraplantar to the hind paw and the time spent licking, biting or flinching the injected hind paw was recorded for 60 minutes in 5-minute intervals.

**Results**: mGluR5IM mice spent significantly longer exhibiting pain behaviour during the late phase of the formalin response compared to mGluR5WT mice. Additionally, in the mGluR5IM group only, females demonstrated significantly more late phase pain formalin behaviour than males.

**Discussion/Conclusions**: This finding supports the importance of the intracellular localisation of mGluR5 in pain modulation and highlights a yet unreported sex difference in mGluR5 nociceptive signalling, which will be further investigated. Future work will assess mGluR5IM mice with a battery of behavioural tests and molecular techniques to establish differences in baseline and post neuropathic/ inflammatory injury behavioural responses and neuronal nociceptive signalling profiles.

**The role of oligodendrocytes in morphine exacerbated neuropathic pain**

Sierra Stokes-Heck, Julia Canet-Pons and Tuan Trang

Hotchkiss Brain Institute, University of Calgary

**Introduction/Aim**: Neuropathic pain resulting from peripheral nerve injury is among the most debilitating types of chronic pain conditions. Opioid medications are often used despite their poor efficacy in treating neuropathic pain symptoms and concerns about adverse effects. Notably, emerging evidence suggests that rather than alleviating neuropathic pain, opioids may worsen mechanical allodynia, wherein innocuous stimuli elicit pain. Morphine has been shown to exacerbate nerve injury-induced mechanical allodynia, but the cause is not understood. Both morphine and nerve injury have been implicated in myelin and oligodendrocytes perturbations. As myelin is primarily composed of lipids, here we determine whether lipid metabolism alterations are a potential mechanism underlying morphine exacerbated neuropathic pain.

**Methods**: Chronic constriction injury (CCI), consisting of three sutures around the sciatic nerve, was performed on eight-week-old male C57/B6J mice. Development of neuropathic pain and mechanical allodynia were followed for 10 weeks using the dynamic weight bearing and von Frey filament tests, respectively. RNA extracted from lumbar spinal cord tissue was profiled using lipid metabolism arrays.

**Results**: We found that morphine treatment of CCI prolonged mechanical allodynia and recovery from nerve injury. Morphine further increased the CCI-induced lipid metabolism changes.

**Discussion/Conclusions**: These results suggest that morphine delays recovery from CCI by dysregulating lipid metabolism at the spinal cord level. As oligodendrocytes are the myelinating cells of the CNS, we propose that alterations in lipid metabolism are linked to changes in this cell type. The potential findings could help mitigate adverse opioid effects and improve treatment for people suffering from neuropathic pain.

**Use it or lose it: The relationship between depth-specific subchondral bone mineral density and kinematic joint loading**

Lauren Straatman^a^, Elizabeth Norman^a^, Nikolas Knowles^b^, Nina Suh^c^, David Walton^a^ and Emily Lalone^a^

^a^Western University; ^b^University of Waterloo; ^c^Emory University

**Introduction/Aim**: According to Wolff’s law, bone tissue is influenced by its mechanical environment and adapts in response to the mechanical load that acts on it. This law has been used in the knee and hip joints to demonstrate bone changes may precede cartilage degeneration in painful diseases such as osteoarthritis, however this has not been studied in the wrist. Our primary objective is to evaluate the correlation between kinematic joint contact area (JCa) and subchondral volumetric bone mineral density (vBMD), as it relates to depth from the subchondral surface in healthy wrists. Our secondary objective is to determine the amount of variance in subchondral vBMD that can be explained by kinematic joint contact, sex, and age.

**Methods**: Twenty otherwise healthy participants (n=11 females and n=9 males) underwent static CT scans of their wrist accompanied by a calibration phantom with known densities, and kinematic CT scans while performing maximum wrist extension to flexion. Average subchondral vBMD was studied at three depths from the surface (0 – 2.5, 2.5 – 5, 5 – 7.5mm) and JCa was studied according to radial articular surface contact (radioscaphoid (RS) and radiolunate (RL)). Pearson product-moment correlations were calculated to determine the relationship between vBMD and JCa, and statistically significant correlations were assessed using a regression model.  

**Results**: The correlation analysis demonstrated significant positive correlations between vBMD and JCa in the deeper regions from the surface, while the regression analysis demonstrated that 38% and 33% of the variance in vBMD can be explained by degrees of motion in the RS and RL joints, respectively.  

**Discussion/Conclusions**: An increase in vBMD was significantly, positively correlated with an increase in JCa, notably in the deeper regions from the subchondral surface that are primarily composed of trabecular bone; a region that is adaptive to change and metabolically active. This relationship is paramount to better understanding joint remodeling following injury, early osteoarthritic signs in the joint separate from normal joint aging, and the influence of vBMD and JCa adaptations on pain. 

**TRIM32-mediated type I interferon signaling causes tactile allodynia in mice lacking translational repressor 4E-BP1 in nociceptors**

Calvin Wong^a^, Diana Tavares-Ferreira^b^, Carolina Thorn-Perez^a^, Behrang Sharif^a^, Sonali Uttam^a^, Kevin C. Lister^a^, Mehdi Hooshmandi^a^, Vivienne Nguyen^a^, Philippe Seguela^a^, Nahum Sonenberg^a^, Theodore J. Price^b^, Gary R. Lewin^c^, Christos G. Gkogkas^d^ and Arkady Khoutorsky^a^

^a^McGill University; ^b^University of Texas at Dallas; ^c^Max Delbrück Center for Molecular Medicine; ^d^Biomedical Research Institute, Foundation for Research and Technology-Hellas

**Introduction/Aim**: mTOR is a highly evolutionarily conserved serine/threonine kinase that regulates cell homeostasis through key cellular processes, including cell growth and proliferation, translation, autophagy, and cytoskeleton organization. mTOR is present in two structurally and functionally distinct multiprotein complexes: mTORC1 (mTOR Complex 1) and mTORC2. mTORC1 regulates the rate of eIF4E-dependent mRNA translation via inhibition of translational repressor 4E-BP1. It has been established that activation of the mTORC1 by general deletion of 4E-BP1 in mice selectively produces robust tactile allodynia.

**Methods**: To understand the underlying mechanism, we selectively ablated 4E-BP1 in Nav1.8-positive nociceptors (4E-BP1 cKO), neurons involved in the processing of tactile allodynia, and assessed behavioral and DRG neuron excitability phenotypes, as well as performed translating ribosome affinity purification (TRAP) to study differentially expressed genes (DEGs) in nociceptors.

**Results**: Our behavioral experiments demonstrated that 4E-BP1 cKO mice exhibit basal tactile allodynia but no thermal phenotypes. Profiling gene expression in nociceptors lacking 4E-BP1 (using TRAP approach) revealed changes in pathways involved in antiviral responses and mitochondrial activity. Follow up experiments showed TRIM32-mediated increase in type I interferon signaling. Blocking interferon receptors reversed the tactile allodynia and neuronal excitability in 4E-BP1 cKO mice.

**Discussion/Conclusions**: Our study demonstrates the central role of eIF4E-dependent translational control in nociceptors responsible for tactile allodynia. Moreover, our results indicate that ablation of 4E-BP1 in nociceptors increases the translation of TRIM32 which promotes interferon-mediated tactile allodynia.

**Cryotherapy delays the recovery from inflammatory pain in sedentary, but not physically active, mice**

Lucas Vasconcelos Lima^a^, Charlotte Pittman^a^, Boaz Laor^a^, Injy Fouda^a^, Luda Diatchenko^a,b^ and Jeffrey S. Mogil^a,c^

^a^Alan Edwards Centre for Research on Pain, McGill University, Montreal, QC H3A 2B4; ^b^Dept. of Anesthesia, Faculty of Dentistry, McGill University, Montreal, QC H3A 2B4; ^c^Depts. of Psychology and Anesthesia, Faculty of Dentistry, McGill University, Montreal, QC H3A 2B4

**Introduction/Aim**: We recently demonstrated that pharmacologically interfering with the acute inflammatory response leads to pain chronification (Parisien, Lima et al., Sci. Transl. Med., 2022). In this follow-up study we test whether cold therapy would produce similar effects and whether this effect is modulated by fitness level.

**Methods**: Paw inflammation was produced in mice via an injection of complete Freund’s adjuvant (CFA). Immediately afterwards, mice received either cryotherapy or control treatment: 3 sessions of 30 minutes per day for three days, in which both hind paws were submerged in 5°C (cryotherapy) or 20°C (control) water. Mechanical paw-withdrawal thresholds were measured at regular intervals over the next few weeks until they returned to baseline values. Further, we tested whether exercise implemented prior to injury would prevent cryotherapy-induced pain chronification. For this, mice were placed into individual cages containing a running wheel for 5 weeks prior to CFA (physical conditioning).

**Results**: Cryotherapy produced an acute anti-allodynic effect 1 h after treatment, but significantly delayed recovery. Exercise performed for 5 weeks prior to CFA injection prevented this pain-chronification effect.

**Discussion/Conclusions**: Our observations suggest that blocking the acute inflammatory response with cryotherapy, despite promoting immediate pain relief, might also lead to pain chronification in sedentary mice. Although this effect seems to be prevented in physically active mice, nevertheless these results add to our previous findings demonstrating long-term deleterious effects of interfering with the acute inflammatory response. If replicated in humans, our observations might lead to changes in long-term established acute pain management protocols.

**Assessment, diagnosis and measurement of pain L’évaluation, le diagnostic et la mesure de la douleur Pupil dilation predicts post-intubation opioid consumption in adults sustaining moderate-to-severe traumatic brain injury: Preliminary findings**

Caroline Arbour^a^, Céline Gélinas^b^, Sheila Alexander^c^, Raoul Daoust^d^, David Williamson^e^, Francis Bernard^d^

^a^Faculty of Nursing, Université de Montréal, Centre de recherche de l’Hôpital du Sacré-Cœur de Montréal, CIUSSS du Nord-de-l’Île-de-Montréal, Montreal, Quebec, Canada; ^b^Ingram School of Nursing, McGill University, Center for Nursing Research and Lady Davis Institute, Jewish General Hospital, CIUSSS West-Centralde l’Ouest-de-l’Île-de-Montreéal, Montreal, Quebec, Canada; ^c^School of Nursing, Department of Acute and Tertiary Care, University of Pittsburgh, PA, USA.; ^d^Faculty of Medicine, Université de Montréal, Centre de recherche de l’Hôpital du Sacré-Cœur de Montréal, CIUSSS du Nord-de-l’Île-de-Montréal, Montreal, Quebec, Canada; ^e^Faculty of Pharmacy, Université de Montréal, Centre de recherche de l’Hôpital du Sacré-Cœur de Montréal, CIUSSS du Nord-de-l’Île-de-Montréal, Montreal, Quebec, Canada.

**Introduction/Aim**: Opioids are used in critically ill patients with moderate-to-severe traumatic brain injury (TBI) to optimize comfort and mechanical ventilation. However, greater opioid use post-intubation puts TBI patients at risk of tolerance and hyperalgesia. Here, we explored the utility of pupil dilation (PD: a recognized marker of arousability), during mechanical ventilation to predict post-intubation opioid consumption in TBI patients.

**Methods**: A convenience sample of 30 moderate-to-severe TBI adults (18-64 years) under mechanical ventilation was recruited in the first 24h of intensive care unit (ICU) admission. PD measurements were performed on one eye once a day at fixed times during the first three days of ICU admission using automated infrared pupillometry (IDMed, France). PD was elicited using calibrated pressure algometry applied to the fingernails. PD amplitude was expressed in percentage using the formula: ([maximal diameter-minimal diameter] /minimal diameter). All opioids received within 7 days following intubation were converted into morphine mg equivalents (MME) and summed up.

**Results**: Pupillary measurements were averaged for each participant. Algometer application resulted in an average increase of 20 ± 14% in pupil diameter. Post-intubation opioid consumption was highly variable across participants ranging from 0.0-217.5 MME. Correcting for the severity of injury, higher PD amplitude was found to be a significant predictor of greater opioid consumption post-intubation (β=0.864, p < 0.001).

**Discussion/Conclusions**: Our preliminary results suggest that pupillometry within the first days of ICU admission can provide accurate insights into TBI patients’ opioid requirements post-intubation. While further validation is required, pupillometry could become a relevant tool for ICU clinicians implementing opioid-sparing preventive measures.

**An exploratory study of treatment goals in individuals undergoing assessment at the goodhope ehlers-danlos syndrome clinic and their relation to pain**

Kristina Axenova^a^, Joel Katz^a^, Maxwell Slepian^b^ and Molly McCarthy^b^

^a^York University; ^b^Toronto General Hospital

**Introduction/Aim**: Ehlers Danlos Syndrome (EDS) is a cluster of genetic disorders associated with connective tissue problems, with pain being a common symptom. This research aims to compare pain intensity, interference, and quality of life scores for individuals who reported pain-related vs. pain-unrelated treatment goals at the time of initial assessment for markers of connective tissue dysfunction at the GoodHope EDS clinic.

**Methods**: A random sample of 100 participants from the GoodHope EDS Clinic database (N=621) who listed three open-ended treatment goals were classified into three groups: Those providing 1) explicit pain-related goals (n=48), 2) ambiguous pain-related goals (n=19), and 3) only pain-unrelated goals (n=33). Responses on the Multi-Dimensional Health Assessment Questionnaire (MDHAQ) and Brief Pain Inventory (BPI) were compared among the 3 groups using a series of one-way ANOVAs.

**Results**: Worst pain over the past 24 hours (BPI), F(2, 91)=3.203, p=.045, was significantly greater in the pain-related (M=7.09; SD=1.8) than the pain-unrelated group (M=5.87; SD=2.5). Pain due to illness over the past week (MDHAQ), F(2, 43.2)=4.054, p=.024, was significantly greater in the pain-related (M=6.86, SD=1.8) than the pain-unrelated group (M=5.28, SD=2.6). No other group differences were statistically significant.

**Discussion/Conclusions**: Participants who identified pain-related treatment goals reported higher pain scores for illness over the past week and worst pain over the past 24 hours than those who listed pain-unrelated treatment goals. Screening individuals for pain-related goals at the time of initial assessment for EDS clinic services may be a valuable consideration for moving through the clinical intake process.

**Promising pain relief? Evaluating the PROMIS-29 in patients with chronic neuropathic pain undergoing a trial with spinal cord stimulation**

Himali Bergeron-Vitez^a,b^, John Kramer^c,d^, Jill Osborn^e,f^ and Vishal Varshney^g,h^

^a^International Collaboration on Repair Discoveries (ICORD), University of British Columbia, Vancouver, Canada; ^b^Department of Anesthesiology, Pharmacology and Therapeutics. Faculty of Medicine, University of British Columbia, Vancouver, Canada; ^c^International Collaboration on Repair Discoveries (ICORD), University of British Columbia, Vancouver, Canada; ^d^Department of Anesthesiology, Pharmacology and Therapeutics. Faculty of Medicine, University of British Columbia, Vancouver, Canada; ^e^Djavad Mowafaghian Centre for Brain Health, University of British Columbia, Vancouver, Canada; ^f^Department of Anesthesiology, Pharmacology and Therapeutics. Faculty of Medicine; ^g^University of British Columbia, Vancouver, Canada; ^h^Department of Anesthesia, Providence Healthcare, Vancouver, BC, Canada

**Introduction/Aim**: Spinal cord stimulation (SCS) success varies among individuals, prompting the need to better understand predictors of intervention success. The Patient-Reported Outcomes Measurement Information System (PROMIS) can be used to assess multiple health domains as it provides a comprehensive description of the patient’s health status. The purpose of this study was to investigate if the PROMIS-29 questionnaires could provide indications of success in patients with chronic neuropathic pain.

**Methods**: Following a retrospective chart review study design, PROMIS-29 version 2.0 questionnaires previously completed by chronic neuropathic pain who underwent an SCS implant trial were collected (n=51). Baseline scores were compared to a healthy population using a T-score metric. Changes in scores between failed and successful trials were compared using independent two-way t-tests. Unbiased recursive partitioning analyses were run to assess success thresholds using baseline scores and changes in scores between failed and successful trials.

**Results**: Sample T-scores were higher for symptoms and lower for functions relative to the healthy population. The greatest change between the successful and failed group was observed in pain interference (p=0.004) and pain intensity (p=0.01). A reduction in pain interference of 27.3% or more (p=0.004) combined with a reduction in pain intensity of 41.7% (p=0.04) or more was associated with a 100% success rate.

**Discussion/Conclusions**: PROMIS-29 is effective in evaluating the effects of SCS on some domains, especially those related to pain. Research in this area would benefit from controlled trials of overall health quality screening effects on long-term treatment outcomes and prospective studies comparing the predictive values of various factors.

**Pain is in the eye of the beholder: Race and gender disparities in observer gaze patterns and pain judgments for pediatric abdominal pain**

Kristina Bogdan^a^, Megan Miller^b^, Tracy Anastas^c^, Amy Williams^d^ and Adam Hirsh^a^

^a^Indiana University-Purdue University Indianapolis; ^b^Cincinnati Children’s Hospital Medical Center; ^c^University of Washington; ^d^Indiana University School of Medicine, Riley Hospital for Children

**Introduction/Aim**: Pain-related disparities across race and gender are prevalent. Pediatric pain adds complexity by including caregivers in the clinical process. We examined observer gaze patterns and pain assessments across child race and gender.

**Methods**: Participants’ gaze patterns were captured while watching four videos of virtual 12-year-olds (male/female, Black/White) with abdominal pain (e.g., grimacing, holding abdomen) and accompanied by their mother. Participants made pain decisions for each patient. We examined total gaze time (TGT) of four areas of interest (child face, child abdomen, mother, non-character space) and their associations with pain assessments.

**Results**: 57 participants completed the study (agemean=20.3, 68% female, 67% White). TGT for Black girl abdomens correlated positively with assessment ratings of secondary gain (r=.40, p < .05). This correlation was larger than and in opposite direction of that for Black boys (z=2.0, p < .05), White girls (z=1.89, p < .05), and White boys (z=2.96, p < .01). TGT for non-character space in Black girl vignettes correlated negatively with ratings of secondary gain (r=-.34, p < .05), and was in opposite direction of that for Black boys (z=-1.89 p < .05) and White girls (z=-1.77, p < .05). TGT for White boy faces correlated positively with ratings of child reaction to pain (r=.37, p < .01). This correlation was larger than that for Black boys (z=1.79, p < .05) and Black girls (z=1.77, p < .05), and was in opposite direction of that for White girls (z=2.56, p < .01).

**Discussion/Conclusions**: The link between observer gaze patterns and pain appraisals differs across child race and gender. Future research with clinicians is needed to determine whether these differences contribute to disparities in pediatric pain care.

**Assessing the relationship between pain experience and nutrition status in children with cerebral palsy**

Anna Reimann^a^, Maykala Owens^b^, Alyssa Merbler^c^, Chantel Burkitt^b^ and Frank Symons^c^

^a^Gillette Children’s Specialty Healthcare; ^b^Gillette Children’s; ^c^University of Minnesota

**Introduction/Aim**: Pain is common for individuals with cerebral palsy (CP). While we know children with CP may have challenges eating and drinking, little is known about how nutrition status may relate to pain in CP. Nutrition is gaining traction as an important component of chronic pain in other populations. We investigated the relationship between caregiver-reported eating and drinking problems and pain intensity, chronicity, and interference in children with CP.

**Methods**: Seventy-nine children (mean age=11.74 years, 50% ambulatory, 51% male) participated. Caregivers reported pain intensity and interference in the last week, chronic pain and chronic pain intensity, and the Eating and Drinking Ability Classification System (EDACS, scored 0-4). Wilcoxon signed rank tests were used to compare groups. Analyses were based on a) meeting criteria for nutrition referral (EDACS score ≥3) and b) any difficulty with nutrition (EDACS score > 0).

**Results**: Fifty-two (66%) children had pain in the last week and 66 (84%) had chronic pain. Fifteen (19%) children met criteria for nutrition referral and 43 (54%) had some difficulty with nutrition. There were no significant differences in pain scores based on nutrition referral (p > .05) and no significant differences in pain intensity or interference in the last week based on nutrition problems (p > .05). Chronic pain intensity was marginally greater in children with nutrition problems (Md=7, IQR=5) compared to those without (Md=5 IQR=5; p=.06).

**Discussion/Conclusions**: There may be a relationship between nutrition and chronic pain in children with CP. Further work powered on nutrition outcome variables and more detailed assessment of nutrition is warranted.

**Investigating sleep as a moderator of pain intensity and pain interference in cerebral palsy**

Alexis Friesen^a^, Alyssa Merbler^a^, Breanne Byiers^a^, Chantel Burkitt^b^ and Frank Symons^a^

^a^University of Minnesota; ^b^Gillette Children’s

**Introduction/Aim**: Both pain and sleep problems are commonly reported in cerebral palsy (CP), both impacting activities of daily living. Sleep problems are associated with chronic pain in typically-developing children, with evidence of a bidirectional relationship, but there is limited investigation in CP. This study aimed to identify if sleep problems moderated the relationship between maximum pain intensity and pain interference in individuals with CP.

**Methods**: We included 54 participants with CP (mean age=14.68 years [range 3-53], 89% GMFCS IV-V) whose caregivers completed questionnaires about their child’s pain intensity and interference in the last week and sleep problems in the last two weeks. The maximum pain intensity, total pain interference, and total sleep problems scores were used for analyses. The data were analyzed using Spearman correlations and a moderation analysis, with pain interference as the outcome.

**Results**: Pain interference was moderately positively correlated with both maximum pain intensity (ρ=0.467, p < 0.001, CI=0.220-0.658) and sleep problems (ρ=0.379, p < 0.005, CI=0.116-0.593). However, the relationship between maximum pain intensity and pain interference score was not moderated by sleep problems (t=-1.26, p=0.21, CI=-0.53 – 0.12).

**Discussion/Conclusions**: This research demonstrates that sleep and pain intensity are separately impacting the amount of interference pain has in the lives of individuals with CP without interaction. This presents two avenues of intervention to improve quality of life. Further work is needed with objective sleep measures and a larger sample, including more participants with mild CP and more individuals without sleep problems.

**“It was such a relief to hear that I wasn’t crazy. I was so in pain, I dreamed I cut off my own leg”: A qualitative analysis of chronic pain patients’ validation and invalidation experiences in Quebec healthcare system**

Catherine Côté and Pascale Devette

University of Montreal

**Introduction/Aim**: Pain invisibility requires healthcare professionals to rely on patient testimony for pain assessment and management. Healthcare practitioner’s reception of a testimony may be determinant for patient’s access to further care and services. This study aims at identifying factors leading to pain validation and invalidation by healthcare professionals, as well as their consequences.

**Methods**: Seventeen participants with chronic pain participated in a 1-hour narrative interview to explore different experiences they had with healthcare professionals in Quebec, in which they felt validated or invalidated in their pain experience. A thematic analysis was then conducted to identify factors and consequences.

**Results**: Patients highlighted the lack of resources in Quebec healthcare system leading to very little time with healthcare professionals, a context that does not foster empathy. The lack of communication between professionals was also mentioned, as well as the need for more collaboration and interdisciplinarity in chronic pain care, including mental health care. Other factors of pain invalidation include gender, healthcare practitioners’ fatphobia, and having a condition unknown to the practitioner or inexplicable. Consequences of validation and invalidation were observed at the individual and systemic levels.

**Discussion/Conclusions**: This research highlights how validation and invalidation of their chronic pain influence patients’ healthcare trajectory, resulting in systemic impacts. Chronic pain is not only the situation of a suffering body. It is lived through identities with different social recognitions. The caregiver must consider the recognition of chronic pain as embedded in a set of power relationships. To prevent prejudices and discrimination, a holistic vision of care is required.

**Comparison of tympanostomy tubes under local anesthesia versus general anesthesia for children**

Isabelle Fournier^a^, Camille Caron^a^, C M McMurtry^b^, Chantal Giguère^c^, Marie-Joëlle Doré-Bergeron^c^ and Mathieu Bergeron^c^

^a^Université de Montréal; ^b^University of Guelph; ^c^CHU Sainte-Justine, Université de Montréal

**Introduction/Aim**: Tympanostomy tube insertion (TTI) is typically accomplished under general anesthesia (GA) in the operating room. A recent statement from American Academy of Otolaryngology–Head and Neck Surgery supported TTI under local anesthesia (LA). We aimed to compare pain between GA and LA in surgically naïve children undergoing TTI. Secondary objectives examined patient’s quality of life (QoL) and parent’s satisfaction.

**Methods**: This prospective single-center study recruited consecutive children undergoing TTI under GA (n=50) or LA (n=50). Standardized observational pain scales (FLACC, CHEOPS) were completed pre-, during the first and second tympanostomy, post-procedure, as well as 1 week postoperatively. General health-related QoL (PedsQL) and QoL specific to otitis media (OM-6) were measured before insertion and 1 week postoperatively. Parental satisfaction was measured 1 week postoperatively. FLACC, CHEOPS, OM-6 and PedsQL were analyzed using repeated measures ANOVA. Parent perception data were analyzed with Chi-square test.

**Results**: Compared with the GA group, the LA group had higher pain levels pre- (7.3 vs 0), during the first and second tympanostomy (7.8 vs 0 and 7.7 vs 0, respectively), and post-procedure (6.9 vs 0) (all p < 0.01). No pain was noted 1 week after surgery in either group and QoL improved post-procedure in both groups. There was no between-group difference in QoL or parent satisfaction regarding anesthesia choice (p > 0.05). Minor complication rates were similar.

**Discussion/Conclusions**: Children experienced significantly less pain under GA than LA. If LA is to be used, pain and distress reducing strategies are critical.

**Psychometric properties of the tampa scale for kinesiophobia in Colombian adults with shoulder pain**

Adriana Angarita-Fonseca^a^, Maria Alejandra Camacho-Villa^b^, Jostin Fabian Diaz Niño^b^, Julian Fernando Duran Corzo^b^ and Dayana Rosa^c^

^a^Université du Québec en Abitibi-Témiscamingue, Rouyn Noranda, Quebec, Canada; Centre de recherche du Centre hospitalier de l’Université de Montréal, Montréal, Quebec, Canada; Universidad de Santander, Bucaramanga, Colombia; ^b^Laboratory of Exercise Physiology, Sports Science and Innovation Research Group (GICED), Unidades Tecnológicas de Santander (UTS); ^c^Department of Rehabilitation, Faculty of Medicine, Université Laval & Centre for Interdisciplinary Research in Rehabilitation and Social Integration (Cirris)

**Introduction/Aim**: Although the Tampa Scale for Kinesiophobia (TSK) is one of the most frequently employed measures for assessing pain-related fear in pain patients, a Spanish version of the TSK adapted to the Colombian population has yet to be evaluated. The study aimed to evaluate the TSK’s reliability and responsiveness for use in Colombian patients with shoulder pain.

**Methods**: Sixty-five individuals with shoulder pain for at least two months were included (30 females; 41 ± 16 years, 73.5 ± 14.7 kg). The TSK was applied three times between March and May 2022. The test-retest reliability was assessed by calculating the Intraclass Correlation Coefficient (ICC) between the first two measures at a 3-day interval (3.2 ± 1.2 days). The difference between the first and third assessments of the TSK (32.3 ± 1.7 days apart) and the Global Perceived Effect (≥3) were taken to determine the responsiveness. The responsiveness analysis was based on the area under the curve (AUC), and an AUC of 0.70 or greater were considered acceptable responsiveness.

**Results**: Principal component analysis favoured a three-factor structure using 11 items: somatic focus, activity avoidance, and pain avoidance (64% of explained variance). No floor or ceiling effects were identified for 11-items scale. The 11-item-TSK had excellent internal consistency (Cronbach’s α=0.84 to 0.86). The test–retest reliability for the total scale was substantial (ICC=0.87, (95% CI: 0.80, 0.92). The 11-item-TSK had acceptable responsiveness (AUC=0.76).

**Discussion/Conclusions**: The Colombian TSK has acceptable reliability and responsiveness for measuring pain-related fear among patients with shoulder pain.

**Healthcare provider beliefs about race differences in pain**

Alexis Grant^a^, Megan Miller^b^, Tracy Anastas^c^ and Adam Hirsh^a^

^a^Indiana University-Purdue University Indianapolis; ^b^Division of Behavioral Medicine & Clinical Psychology, Cincinnati Children’s Hospital Medical Center; ^c^University of Washington School of Medicine

**Introduction/Aim**: Previous studies in laypeople have documented broad endorsement of beliefs that Black people are less sensitive to pain and less likely to report their pain than White people. Recent studies have also found that White people tend to self-report that they themselves are less pain sensitive and less willing to report pain than “typical” White people and “typical” Black people. Little is known about provider beliefs in these domains.

**Methods**: In this secondary data analysis, we examined providers’ (physician residents/fellows; N=145) beliefs about race differences in pain sensitivity and willingness to report pain, as well as providers’ beliefs about their own pain sensitivity and willingness to report pain. We used paired-samples t-tests for these analyses.

**Results**: On average, providers rated the “typical” Black person as less pain sensitive and less willing to report pain than the “typical” White person (sensitivity: t=-3.31, p < .001; willingness: t=-5.48, p < .001). Interestingly, White providers (n=97) rated their own pain sensitivity and willingness to report pain as less than both the “typical” White person (sensitivity: t=-5.20, p < .001; willingness: t=-5.89, p < .001) and “typical” Black person (sensitivity: t=-3.82, p < .001; willingness: t=-2.59, p=.006).

**Discussion/Conclusions**: White providers’ beliefs about race differences in pain were similar to those found in prior studies in laypeople. These beliefs may impact clinical care and contribute to longstanding disparities in pain assessment and treatment. Moreover, the self-other comparisons made by White providers may be important to consider when developing and/or implementing interventions aimed at enhancing perspective-taking to facilitate equity in pain care.

**Comparing cerebral electrical and behavioural pain responses in preterm infants during noxious stimuli**

Connie Ku^a^, Manon Ranger^b^, Shahbaz Askari^a^, Guy Dumont^c^ and Liisa Holsti^b^

^a^University of British Columbia; ^b^University of British Columbia, British Columbia Women’s Hospital and Health Centre; ^c^University of British Columbia, British Columbia Children’s Hospital Research Institute

**Introduction/Aim**: Preterm infants are exposed up to 17 skin-breaking procedures daily in the NICU. Untreated, these painful interventions can result in negative long-term neurodevelopmental outcomes. Accurate pain assessment to treat pain effectively remains challenging in immature infants. Advances in multi-modal assessments, using EEG combined with valid behavioural pain measures, may provide more objective measures of acute pain.

We aimed to explore the relationship between cerebral electrical activity and behavioural responses during a skin-breaking procedure in preterm infants.

**Methods**: Twelve neonates born at 27-37 weeks were evaluated during a heel lance for blood procurement. Frontal brain activity was measured using Neolite, a novel EEG device designed for preterm infants which can capture unique ultra-low frequencies. Cerebral measures and videos were recorded synchronously during baseline, heel lance, and recovery phases of the procedure. Videos of the infants’ behavioural responses were coded using the Behavioral Indicators of Infant Pain (BIIP) scale.

**Results**: Preliminary data from a single case, a male born at 33 weeks gestational age, were examined. BIIP scores varied from no pain to moderate pain during the painful procedure. EEG power dramatically increased from baseline in the delta (0-4 Hz) and gamma (30-80 Hz) frequency bands and were especially notable in the ultra-low frequency band (0.01-1 Hz), which has rarely been reported in the literature.

**Discussion/Conclusions**: This preliminary, exploratory study shows promise to enhance our knowledge of the clinical applicability of the Neolite, combined with behavioural responses to assess pain, with the long-term goal of measuring pain accurately and the efficacy of neuroprotective interventions.

**Assessment of the autonomic nervous system reactivity to experimental pain using actimetric devices: A descriptive correlational cross-sectional study**

Jonathan Landry^a^, Martine Bordeleau^b^, Gwenaëlle Nivet^a^, Samuel Dallaire^a^, Hai-Oanh Bui-Nguyen^a^, Judith Paquin-Veillette^a^, Ming Zhao Du^a^ and Guillaume Léonard^b^

^a^Faculty of Medicine and Health Sciences, University of Sherbrooke; ^b^Centre intégré universitaire de santé et de services sociaux de l’Estrie - Centre hospitalier universitaire de Sherbrooke

**Introduction/Aim**: Pain detection may be inadequate among populations with verbal communication impairments such as individuals with neurocognitive disorders. A wrist-worn actimetric device measuring heart rate (HR) and electrodermal activity (EDA) could be an innovative device for assessing autonomic nervous system (ANS) reactivity and detecting pain. The aim of this study was to test this idea and determine whether it is possible to assess ANS reactivity to experimental pain with an actimetric device.

**Methods**: In this descriptive correlational study, 25 healthy participants immersed their arm into 10°C cold water (CPT) and lukewarm water (control), both for 2 minutes. In a follow-up session, participants underwent a second CPT. HR and EDA were measured with the actimetric Empatica E4TM device and were compared to the PowerLabTM system (gold standard). Pain scores were simultaneously assessed. Non-parametric tests were used to evaluate changes in HR and EDA across different time measurements.

**Results**: Significant changes in HR and EDA were recorded by both devices during the CPT (p < 0,001). The actimetric device recorded an increase in HR during the CPT followed by an HR decrease (p < 0,005), and an increase in EDA during the CPT that remained after the immersion (p < 0,001). Similar results were obtained with the gold standard device, but no correlation was found between pain ratings and physiological measures.

**Discussion/Conclusions**: Wrist-worn devices such as the Empatica E4TM appear to be promising for measuring ANS reactivity to pain and potentially detecting pain. Future studies in clinical settings are needed.

**Lasers, heat thermodes, and time-frequency analysis: How does stimulus modality influence pain related gamma band oscillations?**

Lukas Linde, Oscar Ortiz, Cassandra Choles and John Kramer

International Collaboration on Repair Discoveries (ICORD), University of British Columbia

**Introduction/Aim**: Painful contact heat and laser stimulation offer the ability to characterize nociceptive pathways involved in acute pain processing, by way of evoked potentials. Direct comparisons of laser and contact heat are limited, most notably regarding time-frequency responses to stimulation. This is important in light of recent evidence to suggest that gamma band oscillations (GBOs) in response to laser stimuli represent a surrogate and obligatory measure of pain. Our purpose was to investigate differences in GBOs generated in response to laser and contact heat stimulation of the non-dominant forearm.

**Methods**: Laser and contact heat stimuli were intensity matched to participant pain ratings using a double randomized staircase method. Following intensity matching, evoked electroencephalography (EEG) responses from both stimuli were examined in the time-frequency domain in the same participants (19 healthy adults), across two experimental sessions.

**Results**: At approximately 200ms after stimulation, both contact heat and laser resulted in significant, group-level event related synchronization (ERS) in the low gamma band (i.e., 30 to 60Hz) in central electrode locations (Cc, Cz, Ci). Laser stimulation also generated ERS in the 60 to 100Hz range (i.e., high gamma), at approximately 200ms, while contact heat led to a significant period of desynchronization in the high gamma range between 400 and 600ms.

**Discussion/Conclusions**: Based on our findings, and in light of previous studies, laser and contact heat stimulation generate characteristically different responses in the brain, with only the former leading to high frequency GBOs characteristic of painful stimuli.

**Bringing PoWR to the people: Development of the Perceptions of Work Relationships for Chronic Pain (PoWR-CP) scales**

Valentina Mihajlovic and Dean Tripp

Queen’s University

**Introduction/Aim**: Research has largely been piecemeal when investigating the associations of various aspects of work relationships and work-related outcomes for individuals living with chronic pain. However, when examining the research together, a unified message emerges: positive work relationships benefit physical and mental health for working individuals with chronic pain Therefore, to progress research forward in a unified manner, the present study aimed to develop a single measure of perceptions of work relationships specific to working individuals with chronic pain using Social Exchange Theory, a dominant theoretical framework for understanding the employee-organization relationship.

**Methods**: Following best practice recommendations for scale development, the present study included three steps: 1) item development, in which an item pool was generated, 2) scale development, in which an initial factor structure was explored, and the item pool was refined, and 3) scale evaluation, in which factor structure was confirmed and reliability was assessed. The proposed Perceptions of Work Relationships for Chronic Pain (PoWR-CP) Scale reflects Social Exchange Theory constructs with a pain focus for three types of work colleagues: supervisors, co-workers, and supervisees. As such, three identical 20-item PoWR-CP Scales were developed: PoWR-CP Supervisor Scale, PoWR-CP Co-Worker Scale, and PoWR-CP Supervisee Scale. All data collection occurred online through MTurk and Prolific. Data from 180, 179, and 171 participants for the PoWR-CP Supervisor, Co-Worker, and Supervisee Scales, respectively, was included in exploratory analyses (step 2). Data from 228, 231, and 177 participants for the PoWR-CP Supervisor, Co-Worker, and Supervisee Scales, respectively, was included in confirmatory analyses (step 3). Maximum likelihood exploratory and confirmatory factor analyses were conducted.

**Results**: Three factors were expected to emerge for each PoWR-CP Scale: Perceived Support, Absence of Perceived Stigma, and Perceived Friendship. A 3-factor model had the best fit with nine items loading onto the Perceived Support factor, seven items loading onto the Absence of Perceived Stigma factor, and four items loading onto the Perceived Support factor: PoWR-CP Supervisor Scale, RMSEA=.078, TLI=.931, SRMR=.047; PoWR-CP Co-Worker Scale, RMSEA=.060, TLI=.951, SRMR=.053; PoWR-CP Supervisee Scale, RMSEA=.067, TLI=.938, SRMR=.056. All loadings and factor correlations across the PoWR-CP Scales were significant (ps ≤ .001). All three PoWR-CP Scales were reliable (Cronbach’s alphas=.86-.96).

**Discussion/Conclusions**: The current study is a novel contribution to the literature on chronic pain at work for academics and practitioners. This research advances the current understanding of the impact of interpersonal relationships at work for individuals living with chronic pain as it provides a psychometrically comprehensive measurement of three constructs of Social Exchange Theory relevant to working individuals with chronic pain: perceived support, trust (i.e., absence of perceived stigma), and affective commitment (i.e., perceived friendship). Future research can confidently implement the PoWR-CP Scales to investigate how perceptions of work relationships impact other aspects of an individual’s work experience, such as occupational stress.

**Exploring pain phenotypes in people with early knee osteoarthritis**

Raghava Neelapala^a^, Tuhina Neogi ^b^, Cora E. Lewis^c^, Luciana Macedo^d^, Steven Hanna^e^, Dylan Kobsar ^f^, Deepak Kumar ^g^, Michael Nevitt^g^, Laura A Frey Law^i^, Tom Appleton^j^, Trevor Birmingham^k^, Lisa Carlesso^d^

^a^McMaster University; ^b^Professor, Department of Medicine, Boston University; ^c^Professor, Department of Medicine, University of Alabama at Birmingham; ^d^Associate Professor, School of Rehabilitation Science, McMaster University; ^e^Professor, Department of Health Research Methods, McMaster University; ^f^Assistant Professor, Department of Kinesiology, McMaster University; ^g^Assistant Professor, Boston University College of Health & Rehabilitation Sciences: Sargent College; ^h^Professor, Epidemiology & Biostatistics, University of California San Francisco; ^i^Professor of Physical therapy and Rehabilitation Science, Carver College of Medicine, University of Iowa Health Care; ^j^Department of Medicine and Physiology & Pharmacology, Western University, Canada; ^k^Professor, School of Physical Therapy, Western University, Canada

**Introduction/Aim**: Identification of early knee osteoarthritis (KOA) pain phenotypes will aid in supporting a move towards patient-centred management; however, the ideal components to assess phenotypes are not known.

**Aim**: To explore pain phenotype models using (i) pain-related variables (Model A) and (ii) pain-related and clinical variables (Model B) as indicators in early KOA.

**Methods**: @Early KOA individuals (n=759), defined as those with pain intensity < 3/10, Kellgren Lawrence grade ≤2, intermittent pain none to sometimes and no constant pain were selected from the Multicenter Osteoarthritis (MOST) Study. For Model A, pressure pain threshold (PPTs) (patella and forearm), temporal summation, conditioned pain modulation, pain catastrophizing, sleep quality, depression, and widespread pain (WSP) were included. For Model B, gait speed, step length, quadriceps power and comorbidities were considered along with pain-related variables. Latent Class Analysis identified the optimal phenotype in each model using fit statistics, ensuring each class has a minimum of 10% of the sample. Class profiles of participant characteristics were compared using ANOVA and Chi-Square tests.

**Results**: 759 individuals (62% females) and mean age (SD): 60.3 (9.4) were included. 3-class and 2-class solutions were favoured for Model A and Model B respectively. In Model A, all three classes were distinguished by forearm PPT and WSP. In model B, all variables were significantly different between the two classes. Differences in age, sex, KL grade, and BMI were identified between classes in both models.

**Discussion/Conclusions**: We identified different early KOA pain phenotypes dependent on factors included. These phenotypes require validation with clinically relevant endpoints.

**Pain experience of children with christianson syndrome**

Shajenth Premachandran, Don Daniel Ocay, Diana-Luk Ye, Lois Miraucourt, John Orlowski, Reza Sharif-Naeini and Catherine Ferland

McGill University

**Introduction/Aim**: Children with severe cognitive impairments express pain differently due to difficulties with communication and are unable to self-report pain intensities. This has led to the assumption that children with disabilities and verbal impairments have a higher pain tolerance threshold. To explore this possibility, we recruited fourteen young male participants with Christianson Syndrome (CS) for this study. This X-linked neurodevelopmental disorder is caused by a loss-of-function mutation in the SLC9A6 gene encoding the cation/proton exchanger NHE6. It is associated with autism-spectrum disorder-like symptoms, including mutism and hyposensitivity to pain.

**Methods**: Touch and thermal sensitivity were assessed in a mouse model of CS, to see if the behaviour observed in the mice could be translated to our patient cohort. Children with CS were subjected to a novel observational tool, the Pain Sensory and Painful Situations Questionnaire (PSQ), which takes multiple painful situations into account to broaden the description of pain expression.

**Results**: Using social expressive behaviours of pain, the PSQ documented on a “good day,” two of the participants likely experienced moderate to severe pain most of the time. Using a mouse model of CS, we observed an increased number of aversive responses to innocuous mechanical stimuli compared to control mice, and a similar result was also seen in our patient cohort. About 30-50% of these patients had an aversive response to normally innocuous stimulation like light touch.

**Discussion/Conclusions**: Despite that hyposensitivity to different painful situations was present and vocal expression of pain was less prominent in our sample of CS children, our work suggests they may experience chronic or recurrent pain which importantly calls for treatment.

**Identifying core outcome domains to guide pediatric population with chronic pain living in Alberta**

Magali Robert^a^, Jatin Patel^b^, Elena Lopatina^a^ and Sunita Vohra^c^

^a^University of Calgary; ^b^Alberta Health Services; ^c^University of Edmonton

**Introduction/Aim**: As part of Alberta Pain Strategy, the outcome measures committee’s mandate was to “Establish a provincial chronic pain measurement strategy encompassing patients, providers, and systems, with an emphasis on a succinct number of well-defined, relevant, and meaningful measures.” This include the identification of core outcomes for the pediatric chronic pain population.

**Methods**: In parallel with identifying adult oucomes, a modified Delphi process was undertaken to identify the most important measurement domains in the pediatric poulations.

Using the PedIMMPACT1 domains as a starting point, an online survey was done targeting Alberta stakeholders. The second survey to initial respondents included additional domains identified in round 1. The working group then convened online to review the results. Open discussion ensued until consensus was achieved.

**Results**: The first round sent February 21, 2021, 41 respondents [27 professionals, 13 people with lived experiences (PWLE) or family member, 1 blank] voted all domains to remain and the addition of: treatment options, healthcare optimization, barriers to care, cognition domains. Round two, sent Nov 15, 2021, resulted in no domains being removed (14 respondents; 5 professionals, 8 PWLE or family members, 1 blank). Consensus of the working group members (26 members) meeting January 19, 2022, identified functional outcomes, emotional outcomes and impression of change as core outcomes.

**Discussion/Conclusions**: The core outcome domains selected from this study will be used to identify validated outcome measurement instruments for the pediatric population with chronic pain living in Alberta. Acknowledge: Outcome Working Group Members of the Alberta Pain Strategy.

**Correlation of intake measures in a tertiary care interdisciplinary pain clinic**

Magali Robert^a^, Andrea Bezuidenhout^b^ and Andrew Walker^b^

^a^University of Calgary; ^b^Alberta Health Services

**Introduction/Aim**: Information is gathered at intake to programs to guide practice or identify individuals whose outcomes may be improved. All measures have advantages and limitations. Often, this may result in undue burden on patients who fill these measures with possible duplication of results. This study looks at the correlation between intake measures at the Calgary Chronic Pain Clinic to identify those with high correlation that may be removed generating more streamlined and efficient intake questionnaires.

**Methods**: All patients entering the Calgary Chronic Pain Program (a tertiary level care program with three streams: NMSK pain, pelvic pain or post-concussion headache) are sent a battery of questionnaires to fill online through RedCap. Restricting analysis to data captured at intake, correlation was explored between 13 intake measures, using Pearson’s correlation coefficient (r). A correlation of r > 0.7 was considered high.

**Results**: Data was available for 1125 patients at intake (855 NMSK, 158 pelvic and 112 post-concussion headache). Measures with r > 0.7 included PHQ depression and PHQ anxiety (0.72), PHQ severity with PHQ depression (0.93) and PHQ anxiety (0.92), EQ5D-5L with SMFA daily activity (0.77), SMFA bothersome and EQ5D-5L (0.70) and SMFA bothersome with SMFA daily activities (0.78), BPI interference and SWL bothersome (0.70).Measures with R < -0.7 included the SMF daily with PSEQ (-0.7) and SMF bothersome and PSEQ (-0.8).

**Discussion/Conclusions**: Several intake measures presented with high correlation. As patients progress through the program increasing available sample size, we will complete univariate analysis between correlated intake measures with our selected outcome variables to assess those that present with the strongest associations. This will allow us to produce more efficient, streamlined questionnaires without unnecessary intake measure duplication.

**Characterizing non-cancer breakthrough pain with ecological momentary assessments**

Margot Schmidt, Lukas Linde, Michael Berger and John Kramer

University of British Columbia

**Introduction/Aim**: Many individuals with chronic pain experience transient increases in pain intensity or changes in sensation, referred to as breakthrough pain (BTP). However, non-cancer breakthrough pain remains poorly characterized by existing literature. The current study aims to determine the prevalence, frequency, intensity, sensation, and impact on functioning, of non-cancer BTP using ecological momentary assessments (EMA) delivered over a 30-day period.

**Methods**: Participants aged 18-80 with non-cancer pain were recruited from a physiatry clinic. For 30 days, participants reported their BTP (daily) and baseline pain (weekly) through EMAs sent to their smartphone. These reports assessed BTP occurrences, and described the intensity, sensations, and impacts of these events. Baseline pain intensity and sensations were also assessed.

**Results**: In our preliminary results of n=5, all participants had neuropathic pain. The median frequency of BTP was 3.51 events per day (range=0.93-8.7), and median impact of BTP on functioning was 5.8/10 (range=2.1-8.5). BTP was more intense than baseline pain (BTP=median 7/10; range 5-8.8; baseline pain=mean 6/10, range 4-8.5). Additionally, BTP was more frequently sharp, whereas baseline pain was more frequently hot, dull, and sensitive. All individual sensation intensities were greater for BTP than baseline pain.

**Discussion/Conclusions**: These early findings suggest BTP is prevalent among individuals with non-cancer pain and has severe intensity that impacts daily functioning. Interestingly, the sensations that characterize BTP and baseline pain may differ. Further data collection will provide more nuanced data regarding BTP and increase the generalizability of the study findings across disease populations.

**Distinct chronic widespread pain trajectories in fibromyalgia**

Christophe Tanguay-Sabourin^a^, Azin Zare^b^, Pierre Rainville^a^ and Etienne Vachon-Presseau^c^

^a^University of Montreal/AECRP; ^b^McGill University/AECRP; ^c^McGill University

**Introduction/Aim**: Fibromyalgia syndrome (FS) is marked by widespread pain, but there has been little attention given to understanding how this pain spreads throughout the body. Furthermore, it is currently uncertain whether this spread occurs uniformly among patients. Here, we use an unsupervised machine learning approach to derive subtypes of fibromyalgia patients associated with distinct putative progressions of chronic widespread pain.

**Methods**: Fibromyalgia subtypes were derived from 955 fibromyalgia patients who completed the UK Biobank online pain questionnaires (8-13 years post-baseline visit). Subtypes trajectories were determined using Subtype and Stage Inference model, an unsupervised algorithm of disease progression using probabilistic cross-sectional spatiotemporal partitioning, on the pain ratings entered on 12 distinct body sites (no chronic pain to severe).

**Results**: We found two subtypes of fibromyalgia (Upper FS-Lower FS) associated with distinct trajectories of pain spread. While Upper FS was characterized by pain mostly in the upper body (Neck/Shoulder, Head, Chest, Arm; OR=1.6-5.1, *p* < 0.001), Lower FS was characterized by pain mostly in the lower body (Knee, Leg, Feet; OR=2.0-3.7, *p* < 0.001).

Both subtypes simultaneously captured the spatial (number of sites, Upper/Lower FS: R2=35/46%) and worsening trajectory of pain (worst pain intensity, Upper/Lower: R2=28/31%) as well as pain interference (BPI, Upper/Lower: 33/26%). Patients showing advanced widespread trajectories had longer pain duration (Upper/Lower: r=0.22/0.14, *p* < 0.005) and more neuropathy features (Upper/Lower: r=0.15/0.41, *p* < 0.005).

**Discussion/Conclusions**: Our data-driven model identifies the distinct spreading patterns within fibromyalgia which may inform the etiology and targets for its pain management.

**Towards healthcare equity: Pain assessment and pain management in Indigenous populations**

Kara Turcotte and Fiona Webster

University of Western Ontario

**Introduction/Aim**: Stemming from current and historical health inequities the Indigenous people of Canada experience significant health disparities compared to the general population. One in five Canadians experience chronic pain, but the incidence of chronic pain is highest in Indigenous individuals and households. The overarching aim of an ongoing research stream is to enhance the current understanding of the healthcare experiences of Indigenous peoples who experience acute as well as chronic pain.

**Methods**: Following initial consultations with Indigenous health directors in the Interior of British Columbia, semi-structured conversational-style interviews were held with four registered health care providers that worked with Indigenous patients. These interviews focused on topics such as pain communication, barriers to accessing treatment, and culturally appropriate methods for assessing and treating pain in Indigenous individuals.

**Results:** A qualitative thematic content analysis was conducted with interview transcripts. Initial themes included the importance of decolonizing pain assessment, accepting and encouraging narrative descriptions of pain, the need for relationship building to enhance trust, unique differences in stoicism in the expression of pain, and the need for increased acceptance of the holistic nature of health and well-being.

**Discussion/Conclusions**: While pain is a universal human experience it is becoming increasingly clear it is unique in how it is expressed. There is an urgent need to develop culturally safe approaches to improve equity in the healthcare system for the Indigenous populations of Canada.

**Epidemiology L’épidémiologie Musculoskeletal disorders among Quebec prosthetists/orthotists: Prevalence and financial burden**

Nabiha Benyamina Douma and Abir El-Haouly

Université du Québec en Abitibi-Témiscamingue

**Introduction/Aim**: The burden of musculoskeletal disorders (MSDs) is substantial for individuals and society. Effort to understand MSDs among health care professionals was mostly focused on nurses. MSDs in Quebec prosthetist/orthotists (OPs) are yet to be studied. The objective of this study was to describe the prevalence and the financial burden of the out-off pocket costs of MSDs in these workers.

**Methods**: Between October and November 2020, a web-based cross-sectional study was conducted among OPs working in the province of Quebec (Canada). The survey invitation was disseminated by the Association des orthésistes/prothésistes du Québec and on social media.

**Results**: A total of 168 OPs completed the questionnaire. The mean age of respondents was 37.0 ± 9.8 years and 75.5% were women. A majority reported at least one site of MSDs in the past year (93.6%). Among the MSDs group 87.9% perceived that their symptoms were totally/partially linked to their work. MSDs-related reduced work/home activities in the past year were reported by 60.5% of MSDs group and 15.9% reported absence from work in the past year because of MSDs. MSDs-related health visits and use of over-the-counter medications to relieve MSDs symptoms in the last three months were reported by 60.3% and 67.3% of MSDs group respectively. MSDs-related perceived financial burden was reported by 41.9% of OPs with MSDs.

**Discussion/Conclusions**: MSDs are frequent and burdensome condition among Quebec OPs even in a country with a universal health care system. Our results underline the importance to promote MSDs prevention and to implement workplace management programs.

**Trajectories of pain and depressive symptoms among people living with low back pain during COVID-19 pandemic**

Adriana Angarita-Fonseca^a^, Mathieu Roy^b^, Anaïs Lacasse^c^, Guillaume Léonard^d^, Pierre Rainville^e^, Marie-France Marin^f^, Erika Gentile^b^, M. Gabrielle Pagé^g^ and The Quebec Back Pain Consortium QPRN^h^

^a^Université du Québec en Abitibi-Témiscamingue, Rouyn Noranda, Quebec, Canada; Centre de recherche du Centre hospitalier de l’Université de Montréal, Montréal, Quebec, Canada; ^b^McGill University; ^c^Université du Québec en Abitibi-Témiscamingue; ^d^Research Center on Aging, CIUSSS de l’Estrie-CHUS; School of Rehabilitation, Faculty of Medicine and Health Sciences, Université de Sherbrooke; ^e^Faculty of Dentistry, Université de Montréal; ^f^Université de Montréal; ^g^Research Center of the Centre hospitalier de l’Université de Montréal, Université de Montréal; ^h^Université de Montréal, Université de Sherbrooke, Université Laval, UQAT, Concordia University, UQAC, UQTR

**Introduction/Aim**: The impacts of the COVID-19 pandemic on the pain and depressive symptoms’ trajectories of individuals living with low back pain (LBP) are unknown. We explored trajectories of pain intensity and depressive symptoms over the first 24 months of the pandemic in people with LBP and examined whether they varied as a function of different pre-COVID and early-pandemic characteristics.

**Methods**: This longitudinal study was embedded in the Quebec LBP Study (QLBPS). From April 2020 to November 2022, 291 participants completed an online survey every three months. Group-based trajectory modeling was used to identify patterns of pain intensity and depressive symptoms, and pre-COVID/early pandemic characteristics were put in relation with trajectory membership using multivariate logistic regression.

**Results**: The analysis revealed five trajectories of pain intensity and five trajectories of depressive symptoms. The pain trajectories were: 1) stable slight (n=17, 5.8%); 2) stable moderate (n=103, 35.4%); stable severe (n=81, 27.8%); U-Shape (n=24, 8.3) and inverted U-shape (n=66, 22.7). The trajectories of depressive symptoms were: 1) stable none (n=58, 19.9%); 2) stable slight (n=61, 21,0%); 3) stable mild (n=85, 29.2%); 4) stable moderate (n=59, 21.7%); stable severe (n=24, 8.3%). The severe/moderate pain intensity trajectories were associated with having pre-COVID pain daily or nearly every day, higher pre-COVID pain intensity, and widespread pre-COVID pain. Higher pre-COVID depression, early-COVID acute stress disorder, and early-COVID lockdown measures-related stressful scores were significant predictors of moderate/severe depressive symptoms group memberships.

**Discussion/Conclusions**: Our findings indicated relative stability of pain and depressive symptoms among participants with LBP during the COVID-19 pandemic.

**Perceived origins of chronic pain: A cross-sectional survey of Canadians living with chronic pain**

Andrew Jin^a^, Navroop Liddar^a^, Sepehr Bozorgi^a^, Maurice Zhang^b^, Anaïs Lacasse^b^, Manon Choinière^d^ and Jason Busse^a^

^a^McMaster University; ^b^Western University; ^c^Université du Québec en Abitibi-Témiscamingue; ^d^University of Montreal

**Introduction/Aim**: Chronic pain affects 1 in 5 adults in Canada, and accounts for over 10% of total health expenditures. The primary objective of our study was to identify self-perceived causes of chronic pain among Canadians living with this condition.

**Methods**: We developed an online survey consisting of 41 questions that was distributed nationally through partnerships with patient organizations and community groups.

**Results**: Interim analysis of 197 respondents (93% female, 81 % between 35-65 years of age), showed that most common perceived causes of chronic pain were idiopathic (59%) and degenerative changes (16%). On average individuals reported 3 different body parts affected. Insomnia (90%), physical impairment (61%), and mood disorders (57%) were common. Respondents were more likely to receive care in non-tertiary settings (71%), and receipt of care outside of a hospital-based pain clinic was associated with greater use of complementary and alternative medicine (46% vs 9%, *p* < 0.0001), physiotherapy (46% vs 21%, *p*=0.001), and psychotherapy (24% vs 9%, *p*=0.028). Interventional procedures were more commonly received in tertiary settings, but the difference was not significant (10% vs 19%, *p*=0.10). 1 in 4 respondents endorsed current use of opioids and/or cannabis. More than 50% reported dissatisfaction with their current regimen. Only 54% were currently engaged with a health care provider, and 26% had been without a healthcare provider for longer than 6 months.

**Discussion/Conclusions**: Recruitment for our survey is ongoing; however, preliminary results suggest that most Canadian living with chronic pain perceive no defined etiology to their symptoms.

**Chronic pain in the context of COVID-19: Greater psychological distress, functional limitations, & rates of long COVID**

Geoffrey Rachor^a^, Dalainey Drakes^b^, Michelle Paluszek^a^, Joanna Vint^a^, Steven Taylor^c^ and Gordon Asmundson^a^

^a^University of Regina; ^b^University of Ottawa; ^c^University of British Columbia

**Introduction/Aim**: Approximately 10-35% of individuals who have recovered from COVID-19 will develop Long-COVID, which include psychological and pain-related symptoms lasting for three or more weeks following recovery from infection. The current study was designed to describe and compare rates of COVID-19 infection, Long-COVID, and levels of anxiety and depressive symptoms and COVID-19-related stress and disability among individuals with and without chronic pain conditions.

**Methods**: A population representative sample of 5,812 Canadian and American adults with (n=1,452) and without (n=4,360) self-reported chronic pain completed online measures of COVID stress, anxiety and depressive symptoms, as well as COVID-19 diagnostic status and Long-COVID symptoms during the third wave of COVID-19 infection. Chi-squared tests and independent samples t-tests were used to compare rates of COVID-19 infection, Long-COVID, anxiety and depressive symptoms, and COVID stress and disability between individuals with or without chronic pain conditions.

**Results**: Individuals with chronic pain reported similar rates of COVID-19 infection in comparison to those without chronic pain; however, reported significantly greater rates of Long-COVID (22.0% and 40.9%, respectively; ps < .001). Individuals with chronic pain also reported significantly greater levels of COVID stress (p < .001), disability (25.7% and 48.9%, respectively; ps < .001), and anxiety and depressive symptoms (ps < .001).

**Discussion/Conclusions**: Results of the current study corroborate findings that physical and mental health impacts of the pandemic disproportionately impact individuals with chronic pain. Results further extend findings that individuals with chronic pain are more likely to develop symptoms of Long-COVID following infection. Research and clinical implications will be discussed.

**Exploring the association between bio-psycho-social predictors and chronic pain: An analysis of age and pain type**

Rayehehossadat Rezvaninejad^a^, Gianluca Guglietti^a^, Matt Fillingim^b^, Azin Zare^a^, Christophe Tanguay Sabourin^c^, Jax Norman^a^, Marc Olivier Martel^d^ and Etienne Vachon-Presseau^e^

^a^Faculty of Dental Medicine and Oral Health Sciences, McGill University, Montreal; ^b^Integrated Program in Neuroscience, McGill University, Montreal; ^c^Integrated Program in Neuroscience, McGill University, Montreal; ^d^Faculty of Dental Medicine and Oral Health Sciences, McGill University, Montreal; ^e^Faculty of Dental Medicine and Oral Health Sciences, McGill University

**Introduction/Aim**: The relationship between biopsychosocial (BPS) predictors and Chronic pain (CP) is complex and may vary depending on age and pain type. We aim to describe predictor models for CP types (widespread, neck-shoulder, hip, back, stomach-abdominal, knee, headaches, facial, and multi-site) based on the BPS predictors and age and evaluate the strength of the relationship between BPS pain predictors and CP across pain types and age groups.

**Methods**: We used information from 501,408 individuals aged 38-73 in the UK Biobank dataset. The data were normalized, divided into a train-test set; grouped into age windows using a sliding window analysis approach. For each age window and for each pain type, we analyzed 105 features using logistic regression to predict CP. These features were grouped into ten categories (mood, neuroticism, trauma, sleep, physiological, health, substance use, physical activity, socioeconomic, and occupational) using feature aggregation. The absolute mean coefficients of each category were used to determine the importance of categories per pain considering the age.

**Results**: The AUC scores ranged from 0.70 to 0.94 in different CPs and remained relatively constant through age groups. The highest AUC belongs to widespread pain. Neuroticism and Socioeconomic emerged as important predictors for most pain types and age groups. Socioeconomic predictors may become increasingly important in managing CP as people age. We also observed that trauma is a stronger predictor for chronic musculoskeletal in younger individuals.

**Discussion/Conclusions**: These findings highlight the relationship between BPS predictors and CP while considering age, with possible implications for pain prevention and management.

**Prescription opioid use is associated with co-morbid non-cancer illnesses among chronic non-cancer pain patients**

Azin Zare^a^, Christophe Tanguay-Sabourin^b^, Matt Fillingim^a^, Gianluca Guglietti^a^, Jax Norman^a^, Ronrick Daano^a^, Marc Martel^a^, Luda Diatchenko^a^ and Etienne Vachon-Presseau^a^

^a^McGill University; ^b^Universite de Montreal

**Introduction/Aim**: High rates of opioid prescriptions for chronic noncancer pain (CNCP) have raised questions about which factors influence physicians’ decisions when prescribing opioids. We aim to evaluate the contribution of pain-associated non-cancer illnesses (NCIs) to prescription opioid use in a large cohort of CNCP patients. Predictive modeling was used to understand the patients’ characteristics determining higher opioid use across NCIs.

**Methods**: We analyzed data from 195,808 CNCP participants from the UK-Biobank. Associations between opioid use and 11 major NCIs were described using odds ratios (ORs). Separate predictive models of opioid use were developed from 1) sociodemographic, lifestyle, mental health, and anthropometric measures (i.e., pain-agnostic model) and 2) the location of acute and chronic pain (i.e., pain model). Models’ diagnostic abilities were evaluated using Cohen’s-d effect sizes comparing each NCI group with the NCI-free group.

**Results**: Opioid use was associated with all NCIs, with ORs ranging from 2.2, [95%CI:2.14-2.27] for neurological and psychiatric illnesses and 1.29, [95%CI:1.24-1.35] for immunological comorbidities. The pain-agnostic model showed stronger discriminability between NCI groups and the NCI-free group compared to the pain model, with moderate effect sizes across all categories (average Cohen’s-d=0.40, *p*-value < 0.001).

**Discussion/Conclusions**: Our results suggest that opioids are more frequently prescribed to patients suffering from comorbid conditions, and that associations between chronic pain and opioid use may be confounded by pain-associated illnesses. CNCP patients diagnosed with an NCI had significantly higher pain-agnostic risk scores compared to the NCI-free group, indicating that opioids may be prescribed to co-treat the patients’ pain and overall poor functioning.

**Pain in specific populations (children, elderly, postoperative, cancer, etc.) La douleur dans les populations distinctes (enfants, personnes âgées, patient.e.s postopératoires ou atteints de cancer, etc.)Safety, tolerability, and feasibility of repetitive transcranial magnetic stimulation (rTMS) for youth with chronic pain: Preliminary results**

Elias Abou-Assaly, Jenna Jessa, Spencer Epp, Adam Kirton, Frank MacMaster, Nivez Rasic, Melanie Noel, Laura Rayner and Jillian Vinall Miller

University of Calgary

**Introduction/Aim**: Chronic pain is highly debilitating and prevalent. In 2014, the Alberta Children’s Hospital established an Intensive Interdisciplinary Pain Treatment (IIPT) to help youth with severe chronic pain. In 2020, repeated transcranial magnetic stimulation (rTMS) was added as an additional therapy.

**Methods**: 10 youth underwent IIPT with rTMS. MNI coordinates from the participant’s T1-weighted image were used to localize the dorsolateral prefrontal cortex (DLPFC) using a neuronavigation system. rTMS was applied to the DLPFC at 10Hz for 40 threshold pulses over 4s with an inter-train interval of 26s. Sessions lasted 37.5min every weekday for three weeks. At IIPT baseline and discharge, youth reported on pain, mental health, and ranked their top 3 out of 13 most and least helpful therapies received during IIPT. One-way ANOVAs were performed to compare symptomology at discharge between the historical (IIPT alone, n=35) and rTMS datasets (IIPT + rTMS).

**Results**: There were no significant differences between the historical and rTMS cohorts at discharge with respect to pain and mental health symptoms. Compared to the other therapies, 20% reported rTMS as the third least helpful, 20% reported rTMS as third most helpful, and 60% reported it as neutral.

**Discussion/Conclusions**: To date, no significant concerns regarding the safety, tolerability, and feasibility of adding rTMS to IIPT have been identified. On average, youth reported neutral feelings towards rTMS as compared to the other therapies. As some youth did not perceive the addition of rTMS as particularly helpful, it may be beneficial to offer rTMS as an optional addition to IIPT.

**Surgical Prehabilitation on Chronic Pain Patients in the community: A 20 week clinical trial based on 7 critical components of the SPOC strategy**

Brenda Lau^a^, Emmanuel Abreu^b^, Emma Harris^b^, Malcon Botteon^a^, Neha Singh^b^, Katherine Zhang^b^, Max Sun^b^, Priodarshi Roychoudhury^c^, Milan Zivadinovic^b^ and Rafaela Magalhaes^d^

^a^CHANGEPain Clinic / University of British Columbia; ^b^CHANGEPain Clinic; ^c^CHANGEpain Clinic / University of British Columbia; ^d^CHANGEpain Clinic

**Introduction/Aim**: The COVID-19 Pandemic forced a systematic national shutdown of surgical elective interventions. Many patients with chronic pain struggle with correct surgical preparedness caused my mobility limitations. The aims of this study were to (1) Understand barriers and effectiveness of perisurgical optimization (2) Gather information related to patient’s adherence to ‘change ideas’.

**Methods**: Prior Institutional Review Board approval from the University of British Columbia, 62 patients were screened and 40 enrolled into the 20-week, mixed-method study. The protocol included both medical and non-medical interventions and procedures. Patients were assessed by at least 3 independent physicians during the study. Remotely delivered motivational interviewing and attendance of group medical visits (education) was strongly encouraged. All data was collected on the EMR and transferred to an EDC secure server. Plots and tables were created on SPSS.

**Results**: 80% of patients were on an orthopedic surgery waitlist. 61% identified as female. 65% of patients had been diagnosed with chronic pain for more than 5 years. Adherence was substantially higher for ‘on-site’ delivered measures. Self-referred measures had an average compliance rate of 35%. 48% of patients experienced an 11-20% decrease in their BPI score. 25% of patients experienced a > 20% decrease in BPI score.

**Discussion/Conclusions**: Better communicational infrastructure is a considerable need between community and surgical sites. Motivational interviewing was, by far, the most praised intervention by patients. Next stages of this research should be focused on the appearance of adverse events related to the procedure when compared to the standard of care.

**Nitrous oxide use for children experiencing painful procedures: A survey of Canadian pediatric emergency physicians’ knowledge, attitudes, and practices**

Samina Ali^a^, Summer Hudson^a^, Martin Osmond^b^, Evelyne Trottier^c^, Naveen Poonai^d^ and Raagini Jain^b^

^a^University of Alberta; ^b^University of Ottawa; ^c^CHU Sainte Justine; ^d^Western University

**Introduction/Aim**: Nitrous oxide (N2O) is an inhaled analgesic/anxiolytic gas with ample evidence supporting its safety and efficacy in children. We aimed to characterize pediatric emergency physicians’ perceptions and site-specific procedures surrounding N2O use.

**Methods**: An electronic survey was distributed to all physician members of Pediatric Emergency Research Canada from February-April 2021. Survey items pertained to N2O availability, comfort with N2O use, and perceived barriers/facilitators to its use.

**Results**: Response rate was 67.8% (156/230), with 53.2% (83/156) women; mean clinical experience was 14.7 years (SD 8.6). 40.0% (6/15) of sites had N2O available in the emergency department, and 83.3% (5/6) of these had written policies/procedures in place. Overall, 48.7% (76/156) of physicians reported using N2O in their clinical practice. The most common indications for use were fracture/dislocation reduction (69.7%, 53/76), wound closure (60.5%, 46/76), and incision & drainage (59.2%, 45/76). The most common perceived barriers to N2O included concerns about ventilation/scavenging systems (71.2%, 57/80) and unfamiliarity with equipment (52.5%, 42/80). The most common perceived facilitators were N2O availability (73.0%, 114/156) and clinical experience with N2O (71.7%, 112/156). Of the 51.3% (80/156) physicians who did not use N2O, 93.7% (75/80) did not have access available at their site; notably, 77.3% (58/75) indicated a desire to have access to N2O.

**Discussion/Conclusions**: Despite evidence to support its use, only half of Canadian pediatric emergency physicians currently use N2O as a tool for treating children’s procedure-related pain and distress. Increasing availability of equipment, protocols, and clinical training surrounding N2O use may standardize clinicians’ abilities to better manage children’s acute pain and distress.

**Headaches and sleep difficulties in the early stage of mild traumatic brain injury**

Caroline Arbour, Danny Hjeij, Charles Gervais, Gilles Lavigne and Catherine Gervais

Hôpital du Sacré-Coeur de Montréal

**Introduction/Aim**: Sleep plays a vital role in brain health and brain injury recovery. Difficulties initiating or maintaining sleep are frequently reported in the first weeks after mild traumatic brain injury (mTBI) and often persist for more than 3 months, but the reason(s) remain elusive. This study examined sleep/wake activity following mTBI and its association with headache, a comorbidity often associated with insomnia in other patient groups.

**Methods**: Wrist actigraphy recording was performed for 5 ± 2 consecutive days/nights in 51 individuals at 1-month post-mTBI (57% female; 40 ± 13years) and repeated in 43 individuals at 3-month post-mTBI (58% female; 40 ± 13years). Headache frequency and pain intensity were assessed with items from the Head and Neck Disability Scale (HNDS).

**Results**: Participants were sleeping on average 7 ± 1 hours per night at 1-month and 6 ± 1 hours per night at 3-month, the latter being below the minimal 7–9 hours requirements made by the National Sleep Foundations recommendations for adults. While no significant differences in sleep/wake patterns were found between mTBI participants at both time points, higher frequency of headaches (46% vs. 32%, *p*=0.019) and moderate-to-severe pain intensity (39% vs. 14%, *p* < 0.01) were found at 1-month, compared to 3-month. Correcting for age and depressive symptoms, pain intensity was found to be a significant predictor of wake time after sleep onset at 1-month (β=6.704; *p*=0.034), but not at 3-month post-mTBI.

**Discussion/Conclusions**: Headache intensity could be associated with fragmented sleep during early mTBI recovery. Unalleviated pain should be screened for in all mTBI reporting new-onset or worsening of sleep disturbances.

**Associations between postural orthostatic tachycardia syndrome and pain among individuals assessed for generalized joint hypermobility spectrum disorder and ehlers danlos syndromes**

Andrea Aternali^a^, Maxwell Slepian^b^, Molly McCarthy^b^, Rachel Siegal^b^, Nimish Mittal^b^, Laura McGillis^b^, Joel Katz^a^ and Hance Clarke^b^

^a^York University; ^b^Toronto General Hospital

**Introduction/Aim**: Generalized Hypermobility Spectrum Disorder (G-HSD) and Ehlers-Danlos Syndromes (EDS) are connective tissue disorders characterized by joint hypermobility and chronic pain. Individuals with G-HSD/EDS often face multisystemic issues including postural orthostatic tachycardia syndrome (POTS). Previous research suggests a relationship between POTS and other forms of chronic pain. The current study investigates the association between POTS and pain severity/interference while considering connective tissue dysfunction.

**Methods**: Participants included adult patients referred to the Toronto General Hospital to assess for a potential diagnosis of G-HSD/EDS (N=811). Individuals completed self-report measures of pain severity and pain interference and underwent a three-minute standing test to screen for POTS. Connective tissue dysfunction was assessed via Beighton score, a nine-point scale of joint hypermobility. Independent-samples t-tests were used to evaluate the difference in pain severity, pain interference, and joint hypermobility based on POTS status. Multivariate regression analyses were used to examine if joint hypermobility moderated the relationship between POTS and pain severity or pain interference.

**Results**: There were no significant differences in pain severity, t(809)=1.263, *p*=585, pain interference, t(809)=.588, *p*=315, or joint hypermobility, t(809)=-2.691, *p*=.176, based on POTS status. Controlling for age and sex, the interaction between POTS status and joint hypermobility did not significantly predict pain severity, R2=0.02, or pain interference, R2=0.01.

**Discussion/Conclusions**: The current results do not suggest a unique relationship among POTS, pain, and connective tissue dysfunction. Further experimental research is needed to characterize this relationship in G-HSD/EDS.

**Greater trauma symptoms contribute to poorer health-related quality of life in LGBTQ2S+ youth**

Kelsey Barrie^a^, Adeline Eldred^b^, Tarannum Rahnuma^b^, Neta Bar Am^b^, Nivez Rasic^b^, Melanie Noel^b^, Daniel Kopala-Sibley^b^ and Jillian Vinall Miller^b^

^a^Alberta Children’s Hospital; ^b^University of Calgary

**Introduction/Aim**: Chronic pain (pain > 3 months) often emerges in adolescence, and affects approximately 25% of youth. Exposure to adverse childhood experiences (ACEs) and subsequent post-traumatic stress symptoms (PTSS) are associated with the development of pain problems in youth. Individuals identifying as LGBTQ2S+ are exposed to greater ACEs, as well as systemic injustices/barriers that could be a source of toxic stress, thereby exacerbating risk of negative outcomes as compared to heterosexual/cisgender peers. Therefore, we hypothesized that LGBTQ2S+ youth would report greater pain interference and poorer health-related quality of life (HRQOL) as compared to heterosexual/cisgender peers.

**Methods**: 50 youth from the community, aged 14-18 years, were invited to complete online surveys two times, three months apart. Youth reported on their gender and sexuality, as well as completed reliable and validated questionnaires on PTSS, HRQOL, pain interference and anxiety. Linear mixed models were used to examine the relationships between sexuality, ACEs, PTSS, pain interference and HRQOL, controlling for gender and anxiety.

**Results**: 13/50 (26%) of youth identified as LGBTQ2S+. Youth identifying as LGBTQ2S+ reported greater ACEs and PTSS relative to heterosexual/cisgender peers (p < 0.05). Sexuality moderated the relationship between PTSS and HRQOL, such that greater PTSS were associated with lower HRQOL over time, however this effect was greater for individuals identifying as LGBTQ2S+ (p < 0.05).

**Discussion/Conclusions**: LGBTQ2S+ youth are at greater risk for experiencing ACEs, PTSS, and poorer HRQOL. Assessment for PTSS and implementation of trauma-informed care may prevent negative long-term effects on the pain, physical and mental health of LGBTQ2S+ youth.

**Behavioural and psychological correlates of parents’ physiological responses during toddler vaccination**

Shaylea Badovinac^a^, Hartley Garfield^b^, Deena Savlov^b^, Eitan Weinberg^b^, Dan Flanders^b^ and Rebecca Pillai Riddell^a^

^a^York University; ^b^University of Toronto

**Introduction/Aim**: Parents play a central role in supporting their children’s acute pain-related distress during infancy and early childhood. Although parents’ responses to child pain are hypothesized to reflect an interplay of biological and psychological factors, few studies have investigated the mechanisms underlying caregiving responses in an acute pain context. The current study explored how parents’ physiological responses to toddler vaccination (i.e., high-frequency heart-rate variability [HF-HRV]) relate to parents’ behavioural and psychological responses.

**Methods**: Video footage and electrocardiograph (ECG) data were collected from parent-toddler dyads (n=224) during toddlers’ 12-month, 18-month, or 24-month routine vaccinations. Parents’ physiological responses (HF-HRV) were measured from ECG data, parents’ behaviour was coded from video footage, and parents self-reported their state anxiety and general stress levels (i.e., parenting stress and psychological stress). Growth curve modeling was used to examine how parents’ behavioural and psychological responses relate to parents’ HF-HRV responses across the vaccination procedure.

**Results**: Parents’ HF-HRV levels showed a significant linear decrease over time across the three minutes following the vaccination needle. After controlling for parents’ baseline HF-HRV levels, parents’ initial HF-HRV reactivity and rate of decline in HF-HRV over time were significantly associated with their use of soothing behaviours (i.e., distraction and rocking) and their self-reported parenting stress.

**Discussion/Conclusions**: Consistent with theory, results suggest that parents’ responses to their child’s pain are impacted by factors that are both proximal and distal to the acute pain context. These findings highlight the importance of educating and supporting parents as a strategy for supporting young children in pain.

**A longitudinal examination of pain as a risk factor for the first onset of depression in adolescence**

Emily Bernier^a^, Sabine Soltani^a^, Melanie Noel^b^ and Daniel C. Kopala-Sibley^b^

^a^University of Calgary; ^b^University of Calgary, Alberta Children’s Hospital Research Institute, Behaviour & the Developing Brain Theme, Hotchkiss Brain Institute, Mathison Centre for Mental Health Research & Education

**Introduction/Aim**: Pediatric chronic pain is a risk factor for mental health issues (e.g., depression, anxiety) well into adulthood. The vast majority of research to date has examined internalizing mental health issues among youth who already live with chronic pain. Less is known about whether pain (even before it become chronic) confers risk for first onset of mental health issues during the period of adolescence. This longitudinal investigation follows a cohort of youth who have a parental history of mood and/or anxiety disorders. The examination of symptoms of pain (intensity, interference, as well as pain catastrophizing, predicted first lifetime onset of depressive, but not anxiety disorders.

**Methods**: The study included 145 youth (M=13.74 years; 64% female) who completed structured diagnostic interviews to assess for depressive and anxiety disorders at baseline and follow-up time points (9 and 18 month). Participants also completed self-report baseline questionnaires which measured pain intensity, interference, catastrophizing, and depressive and anxiety symptoms.

**Results**: Approximately half of participants developed a depressive disorder (47.3%), while 15.7% developed an anxiety disorder. Around ten percent (10.3%) developed both. An increase in pain intensity, interference, and catastrophizing predicted a 1.7 to 2.6 fold increase in likelihood of developing first onset depressive disorder but not anxiety disorder, at follow-up.

**Discussion/Conclusions**: Increased pain intensity, interference, and pain catastrophizing presents a premorbid risk factor for the onset of depression in adolescent youth. These findings underscore the importance of early intervention in pain, as mental health (and especially depression) are one of the biggest global burdens of disease to society.

**Identifying risk and protective factors in the intergenerational transmission of chronic pain: A prospective cohort study**

Jaimie Beveridge, Melanie Noel, Sheri Madigan, Serena Orr, Sheila McDonald, Suzanne Tough and Kathryn Birnie

University of Calgary

**Introduction/Aim**: It is well-established that children of parents with (versus without) chronic pain have increased risk for poor outcomes (e.g., worse physical and socio-emotional functioning). Less is known about variability among children of parents with chronic pain, including factors that break the intergenerational transmission of risk. This study examined factors associated with increased or reduced risk for mental health problems and chronic pain in children of mothers with chronic pain.

**Methods**: Data from a subsample of mothers with chronic pain (n=307; mean age=36.87) and their children (52% boys) were analyzed from a larger, prospective pregnancy cohort study (N=1991). At child age 5, mothers self-reported their chronic pain. At child age 8, mothers completed surveys that assessed potential risk (ineffective/hostile parenting, maternal anxiety and depression, maternal stress) and protective (positive parent-child interactions, maternal social support, happiness in relationship, household income) factors, as well as child internalizing problems, externalizing problems, and chronic pain. Separate multivariable logistic regressions were conducted for risk and protective factors while controlling for child sex.

**Results**: Ineffective/hostile parenting (aOR=1.15, 95%CI=1.06-1.25), positive parent-child interactions (aOR=0.87, 95%CI=0.78-0.98), and maternal social support (aOR=0.89, 95%CI=0.83-0.96) were significantly associated with child mental health problems. No risk or protective factors were significantly associated with child chronic pain.

**Discussion/Conclusions**: Among children of mothers with chronic pain, factors that significantly increased or reduced risk were identified for mental health problems, but not chronic pain. These pathways to risk or resilience will be re-examined when the children enter adolescence (age 12-13, when the incidence of chronic pain increases).

**Pain in juvenile idiopathic arthritis: A systematic review of psychosocial factors**

Yvonne Brandelli^a^, Christine Chambers^a^, Sean Mackinnon^b^, Emily Wildeboer^a^, Jennifer Parker^c^, Adam Huber^a^, Jennifer Stinson^d^, Jennifer Wilson^e^ and Olivia Piccolo^c^

^a^Dalhousie University & IWK Health Centre; ^b^Dalhousie University; ^c^IWK Health Centre; ^d^University of Toronto & The Hospital for Sick Children; ^e^Cassie + Friends

**Introduction/Aim**: Youth with Juvenile Idiopathic Arthritis (JIA) report pain as a common experience; however, pain management continues to be a challenge. As pain is a multidimensional experience that is influenced by biological, psychological, and social factors, the key to effective management lies in understanding these complex relationships. This study systematically reviewed the literature on psychosocial factors associated with and predictive of JIA pain intensity, frequency, and sensitivity in youth 0-17 years of age.

**Methods**: Using the Joanna Briggs Institute methodology, terms related to pain and JIA were searched in English without date restrictions across various databases (CINAHL, PubMed, PsycINFO, Embase, Scopus, and the Cochrane database). Two reviewers independently identified, extracted data from, and critically appraised the included studies. Conflicts were resolved via consensus.

**Results**: Of the 9,929 unique studies identified, 61 studies reporting on 516 factors were included. Results were heterogeneous due to moderate study quality and methodological differences. Significant associations were generally identified between pain and child and parent internalizing symptoms, child and parent cognitions (e.g., pain beliefs, self-efficacy), worse social functioning, and lower well-being. Internalizing symptoms and lower well-being were associated with worse pain over time, whereas cognitions (i.e., fewer beliefs of harm, disability, and lack of control) and psychological intervention participation were associated with improved pain over time (1-60 months later).

**Discussion/Conclusions**: Clinically, findings support an interdisciplinary approach to pain management and provide information to better optimize psychosocial interventions. Future research should incorporate larger sample sizes, child pain reports, and more complex study designs and analyses.

**An investigation of the predictive and concurrent relations between caregiver heart rate and infant pain behaviours during vaccinations**

Oana Bucsea^a^, Cheryl Chow^a^, Ilana Shiff^a^, Hartley Garfield^b^, Dan Flanders^c^, Eitan Weinberg^c^, Deena Savlov^c^ and Rebecca Pillai Riddell^d^

^a^York University; ^b^University of Toronto, The Hospital for Sick Kids; ^c^University of Toronto; ^d^York University, University of Toronto, The Hospital for Sick Kids

**Introduction/Aim**: Although past research has recognized the critical role of caregivers in shaping infants’ responses following a painful medical procedure, the relations between caregivers’ cardiac stress and infants’ pain behaviours have remained largely understudied. The aim of the current study was to examine the predictive and concurrent associations between caregivers’ cardiac responses and infants’ pain-related behaviours during a vaccination appointment.

**Methods**: The relationships between caregivers’ cardiac stress (heart rate, [HR]) and infants’ pain behaviours (Face, Legs, Activity, Cry, Consolability coding system, [FLACC]) were measured during infants’ 12-months vaccination (n=166). Both infant FLACC and caregiver HR were measured at four different epochs: immediately before the needle (60 seconds pre-needle), immediately post-needle (0-30 seconds post-needle), 1-minute post-needle (60-90 seconds post-needle), and 2-minutes post-needle (120-150 seconds post-needle). A cross-lagged path model using a full-information maximum likelihood estimator to incorporate incomplete cases was employed to investigate the proposed relationships.

**Results**: Significant predictive within-measure relations were uncovered for both caregiver HR and infant FLACC. Infant FLACC immediately post-needle positively predicted caregiver HR 1-minute post-needle (b=.07, p=.01). Finally, infant FLACC and caregiver HR showed a significant positive concurrent association 1-minute post-needle (b=.15, p < .05).

**Discussion/Conclusions**: Overall, it appears that the more pain behaviours infants exhibited immediately following a needle, the more cardiac stress caregivers subsequently experienced 1-minute post-needle. Furthermore, at 1-minute post-needle, infants of caregivers experiencing higher cardiac stress levels demonstrated more pain behaviours, underscoring the importance of healthcare providers supporting both infants and their caregivers during stressful medical procedures.

**Are there age differences in pain stoicism? Results from equivalence tests**

Louise Castillo, Delaine Shackleton, Amy Hampton, Andrei Volodin, Thomas Hadjistavropoulos

University of Regina

**Introduction/Aim**: According to a widely cited assertion, compared to younger individuals, older adults are less likely to express pain complaints. Age-related differences in pain responses have been discussed in the literature despite a paucity of research involving direct comparisons of younger and older adults’ pain reactions. We previously identified a lack of age effect in various verbal and nonverbal pain response domains (e.g., stoicism, pain intensity, facial pain expressions) between younger and older adults. As such, our goal was to determine whether the lack of age effect represented equivalence in pain responses among younger and older adults.

**Methods**: We measured pain-related stoicism as well as multiple responses to experimentally induced pain. Two one-sided tests were conducted against defined equivalence bounds. The smallest meaningful group difference was determined to represent one standard deviation difference between the groups for each variable.

**Results**: Equivalence testing indicated that older and younger adults displayed similar verbal and non-verbal pain responses (e.g., pain stoicism, pain intensity/unpleasantness, facial pain expression). Our results suggest that older adults are no more stoic about their pain than younger persons.

**Discussion/Conclusions**: Our findings suggest that views in the field regarding pain stoicism among older adults may need to shift.

**The association between caregivers’ cardiac and behavioral responses during routine vaccinations in toddlerhood**

Cheryl Chow^a^, Oana Bucsea^a^, Ilana Shiff^a^, Miranda DiLorenzo-Klas^a^, Hartley Garfield^b^, Dan Flanders^c^, Eitan Weinberg^c^, Deena Savlov^c^ and Rebecca Pillai Riddell^a^

^a^York University; ^b^The Hospital for Sick Children; University of Toronto; ^c^University of Toronto

**Introduction/Aim**: In over 25 papers over the past decade, research from the original OUCH Cohort has shown the power of caregivers’ behaviours in shaping healthy infant pain responses over the first year of life. Despite the hypothesized influence of caregivers’ cardiac activity on their ability to engage in pain management behaviours, there has been a paucity of studies examining the role of caregiver physiology and behaviour in paediatric pain contexts.

**Methods**: Using a series of autoregressive cross-lagged path models, data from a longitudinal cohort of caregivers (n=222) during routine vaccinations in toddlerhood will be presented to demonstrate the associations between caregivers’ cardiac and behavioral indicators (i.e., distraction, verbal reassurance and proximal soothing) across time. Within each model, both concurrent and predictive relations will be examined.

**Results**: Preliminary results revealed that within-measure relations across indicators were positive, with each postneedle cardiac or behavioral response positively predicting the subsequent cardiac or behavioral response (*p* < .05). In addition, positive predictive relation was found between heart rate and distraction immediately postneedle (*p* < .05). Concurrent relation was also found between heart rate and verbal reassurance 3 minutes postneedle (*p* > .05).

**Discussion/Conclusions**: To the best of our knowledge, this is the first longitudinal study of typically developing toddlers to examine the relations of caregivers’ cardiac and behavioural indicators during toddlerhood vaccinations. These findings suggest that caregivers’ and cardiac responses have high stability within the acute pain context in toddlerhood. Interestingly, we also found predictive relations between caregivers’ physiology and behaviours during both the reactivity and regulation phases.

**Chronic struggle: A new framework for understanding chronic pain and marginalization**

Fiona Webster^a^, Laura Connoy^b^, Abhimanyu Sud^c^, Kathleen Rice^d^, Joel Katz^e^, Andrew Pinto^c^, Ross Upshur^c^ and Craig Dale^c^

^a^University of Western Ontario; ^b^Western University; ^c^University of Toronto; ^d^McGill University; ^e^York University

**Introduction/Aim**: In 2021, The Lancet published a special issue focusing on the need to re-think chronic pain. This was in part due to the growing awareness that social inequities impact chronic pain. While important, most research in this field continues to draw on clinical and biomedical approaches that fail to adequately take into account critical theoretical understandings of the social. While pain models do attempt to take the social into account, they still often position the social as: a separate domain external to people’s health; a binary of the medical/psychological; or, conflate the social with the subjective (i.e. individual). A meaningful rethinking of chronic pain must be anchored within the social lives of people living with chronic pain and social inequities.

**Methods**: Our team undertook a sociological approach known as institutional ethnography (IE) to examine the time and effort (also referred to as work) involved in managing lives beset by chronic pain and marginalization. Using a generous conception of work, we go outside the frame of paid employment to explore what people do (and how they know how to do it) in particular places with finite resources. Work thus defined is rarely addressed in descriptions of people who live with chronic pain, let alone chronic pain and marginalization. Upon obtaining approval from our Research Ethics Board (REB), we purposively recruited people living in Canada, over the age of 18 years, spoke fluent English, who self-identified as living with chronic pain, and also considered themselves as having difficulty making ends meet. While IE makes use of several types of data and methods of collection, this study relied on telephone interviews due to pandemic restrictions. Data was transcribed and indexed into people’s insights about the health work they performed daily. We also paid attention to authoritative texts and discourses embedded in the activities participants described. Our team met multiple times via Zoom to review transcripts, refine codes, and identify themes. An overview of our findings and analysis was shared with a Patient and Community Advisory Committee comprised of both people with lived experience and members of community organizations.

**Results**: We organize our findings first around four aspects of work that are featured in navigating peoples’ health and social lives. These include the work of: 1) managing chronic pain alongside poverty and subsistence, 2) legitimizing their struggles when dealing with health and social institutions, 3) adhering to biomedical models, and 4) navigating multiple, often ill-fitting, diagnoses. We then combine them to explicate how chronic pain and the experience of marginalization overlap in people’s everyday experiences through the concept of chronic struggle.

**Discussion/Conclusions**: We offer a new concept of “chronic struggle” which provides the conceptual space to explicate the work involved in managing lives beset by chronic pain and marginalization within de-legitimizing and stigmatizing systems. The struggles faced by those living with chronic pain and marginalization are often ignored by biomedical discourses that emphasize the individual. In this regard, chronic struggle captures how pain and inequity constitute a knot of experience that people are obliged to simplify to fit existing logics of medicine. Through this concept, we can meaningfully attend to how and in what ways people experience their encounters with social conditions alongside their chronic pain. Chronic struggle will enable primary care providers and policymakers to better recognize the ways in which the connections between personal experience and larger social systems and structures influence experiences of chronic pain, and work to improve the lives of those entangled in knots.

**A double-edged sword: The pandemic from the standpoint of people experiencing chronic pain and marginalization**

Laura Connoy^a^, Abhimanyu Sud^b^, Joel Katz^c^, Craig Dale^b^, Kathleen Rice^d^, Andrew Pinto^b^, Ross Upshur^b^ and Fiona Webster^e^

^a^Western University; ^b^University of Toronto; ^c^York University; ^d^McGill University; ^e^University of Western Ontario

**Introduction/Aim**: Canada and the world faced unprecedented challenges with the COVID-19 pandemic. Indeed, these challenges persist today. Governments and health care systems are seeking out ways to minimize the impacts of the pandemic on its citizens. Nevertheless, within Canada, as in many other countries, systemically and structurally marginalized populations are disproportionately susceptible to COVID-19 infection, particularly those living with chronic pain. Pandemic response measures create complex barriers to essential health and social services for this segment of the population. However, studies have yet to adequately include the voices of people living with chronic pain and marginalization. To gain a more wholistic understanding of the experiences of chronic pain during the pandemic, researchers must also take into consideration the complex and multi-faceted nature of the social context of peoples’ lives. Doing so will call critical attention to the various impacts of policies and public health recommendations, and responses to them.

**Methods**: We drew on a sociological approach known as institutional ethnography (IE) for our study. IE uses people’s everyday experiences as the starting point for an exploration of how these experiences are coordinated. Given the pandemic restrictions, participants were recruited in online spaces (e.g. Twitter and Kijiji) and through the existing networks of our multi-disciplinary study team and interviewed by telephone. Interviews were approximately 60-90 minutes in length. Our study focused on people living in Canada over the age of 18, who speak fluent English and who self-identified as both living with chronic pain, and struggling to make ends meet. We interviewed participants about what they do as they go about their everyday lives. Data was transcribed and indexed (grouped into inter-related themes that focused on social organization rather than individual experiences), and was analyzed in larger interdisciplinary team meetings

**Results**: Our preliminary findings include: 1) Interchangeable experiences pre- and peri-pandemic; 2) Heightened (risk of) food insecurity and its implications on chronic pain; 3) Public transportation obstacles; 4) Levelling the playing field; and 5) Primed for pandemic-related restrictions.

**Discussion/Conclusions**: While the pandemic is normatively understood as collective trauma or struggle (we are “all in the same boat”) that brought about immediate drastic changes to everyday life, people struggling with chronic pain and marginalization discussed the pandemic as but one additional struggle that compounded already existing health and social struggles. In some ways however, it simultaneously alleviated struggles for some. In this regard, the pandemic operated more as a double-edged sword. Through this more nuanced understanding of the pandemic, that begins in the standpoint (i.e., specific location of people within existing social systems) of people living with chronic pain and marginalization, we can begin to acknowledge their expertise and narrow in on targeted interventions to address structural inequities. For example, providing support to bolster people’s incomes should remain a priority. This knowledge is critical to help better (re)design and strengthen our health and social systems. Furthermore, our study highlights the contributions of qualitative sociological research in identifying often ignored complex social relations and inequalities that shape experiences of chronic pain.

**Pain in popular adolescent media**

Allison Cormier^a^, Samantha Noyek^a^, Katya Dittrich^b^ and Melanie Noel^a^

^a^University of Calgary; ^b^St. Mary’s University

**Introduction/Aim**: Pain is common in adolescent media, with an average of 10.24 pain instances per hour of media. Critically, media consumption can play a vital role in the socialization of pain. Findings from our previous research examining pain depictions in popular adolescent media have shown a representation of low empathy levels, increased violence, and an underrepresentation of gender and racial diversity. This research aims to explore how adolescents internalize the messages they see within pain depictions in the media.

**Methods**: This study will include thirty-two youth between 16 and 18 years of age, both with and without chronic pain. The sample aims to involve diverse individuals who are underrepresented in adolescent media (e.g., Black, Indigenous, People of Colour, and LGBTQ2S+). Online focus groups (approximately 1-2 hours in length) are being conducted wherein youth watch and discuss clips that depict pain in the media.

**Results**: Data collection is ongoing. Four adolescents (M=18.1 years, SD=0.37; 75% male) with and without chronic pain have participated in a focus group. Qualitative analyses are in progress with preliminary themes to come. Initial coding has brought forth concepts related to i) lack of expression in sufferer ii) media as a tool to harness empathy to action iii) gendered and racialized representation of pain.

**Discussion/Conclusions**: The findings suggest media norms regarding the roles of sufferers, observers, and diverse individuals. This study will build an understanding of how youth learn and respond to these norms and other pain messages.

**Toward a better understanding of pain severity, pain interference and symptoms of dysphoria in Ehlers Danlos Syndrome**

Charly Daly^a^, Kristina Axenova^a^, Joel Katz^a^, Hance Clarke^b^, P Maxwell Slepian^b^, Molly McCarthy^c^, Nimish Mittal^d^,and Laura McGillis^c^

^a^York University; ^b^Toronto General Hospital, University Health Network; ^c^Toronto General Hospital GoodHope Ehlers-Danlos Syndrome Clinic; ^d^Toronto General Hospital, University Health Network, GoodHope Ehlers Danlos Syndrome, Program

**Introduction/Aim**: Introduction: Generalized Hypermobility Spectrum Disorders (G-HSD) and hypermobile Ehlers-Danlos Syndrome (hEDS) are connective tissue disorders characterised by chronic pain, joint instability, and a range of organ dysfunction. The two diagnoses are often conflated, used interchangeably, or grouped together in clinical studies. However, comparisons between these two groups have been limited due the rarity of the disorder and small sample sizes.

**Aims**: To compare pain intensity, pain interference, and dysphoria symptoms in a large sample of individuals diagnosed with G-HSD vs. hEDS.

**Methods**: 419 participants with diagnoses of G-HSD (n=345) and hEDS (n=74) were examined from a retrospective database at the Toronto General Hospital GoodHope EDS Clinic. Responses on the Brief Pain Inventory (BPI) and the Inventory of Depression and Anxiety Symptoms - Dysphoria (IDAS-D) were compared using independent t-tests. The BPI average and worst pain scores were compared using Mann-Whitney U Tests.

**Results**: Dysphoria symptoms (IDAS-D) did not differ significantly between patients with hEDS (M=26.1, SD=9.1) and G-HSD (M=27.3, SD=9.5), t(417)=-0.981, p=0.33. Similarly, pain interference scores did not differ between patients with hEDS (M=5.5, SD=2.4) and G-HSD (M=5.6, SD=2.5), t(673)=-0.228, p=0.82. Finally, significant differences were not found in average pain (BPI5), U=36.0, Z=-0.544, p=0.59 and worst pain (BPI3), U=36550.50, Z=-0.481, p=0.63, between the two groups.

**Discussion/Conclusions**: Individuals with hEDS and G-HSD did not significantly differ on average pain, worst pain within 24 hours, pain interference, or dysphoria symptoms. Future research should examine whether these two patient groups differ on other symptoms.

**Factors that influence parental decision-making regarding analgesia for their children with musculoskeletal injury-related pain: a qualitative study**

Zoë Dworsky-Fried^a^, Mackenzie Moir^a^, Manisha Bharadia^a^, Manasi Rajagopal^a^, Serge Gouin^b^, Scott Sawyer^c^, Stephanie Pellerin^b^, Lise Bourrier^c^, Naveen Poonai^d^, Antonia Stang^e^, Michael van Manen^a^, Samina Ali^a^ and on behalf of the KidsCAN PERC Innovative Pediatric Clinical Trials No OUCH Study Team

^a^University of Alberta; ^b^CHU Sainte-Justine; ^c^University of Manitoba; ^d^Western University; ^e^University of Calgary

**Introduction/Aim**: Parents/caregivers are often the gatekeepers to the pharmacologic management of their children’s pain. Concerns and preferences regarding medications, including opioids, influence their analgesic decisions. Our primary objective was to explore and understand caregiver decision-making as it relates to acute pain management for their children presenting to the emergency department.

**Methods**: This study employed one-on-one semi-structured interviews. Parents of children with acute musculoskeletal injuries were recruited from three Canadian pediatric emergency departments (Edmonton, Montréal, Winnipeg). Interviews were conducted via telephone from June 2019 to March 2021. Verbatim transcription and thematic analyses occurred concurrently with data collection, supporting data saturation and theory development considerations.

**Results**: Twenty-seven interviews were completed. Five major themes regarding pain assessment and treatment emerged: a) My child’s comfort is a priority; b) Every situation is unique; c) Opioids only if necessary; d) Considerations when choosing opioids; and e) Pain research is important. Overall, parents were highly comfortable with their assessment of their child’s pain. Participants’ willingness to use opioid analgesia for their children was primarily dependent on perceptions of injury and pain severity. Opioid-averse and opioid-accepting families had similar considerations when making analgesic decisions but weighed risks and benefits differently.

**Discussion/Conclusions**: Parents assess and manage their children’s pain and distress as a global entity, with comfort being prioritized. For most parents, the desire to relieve their children’s pain outweighed concerns of addiction, misuse, and adverse events when making decisions about opioid analgesia. These results can inform evidence-based family-centered approaches to shared decision-making of analgesic plans for children with acute pain.

**Chronic post-surgical pain in women with breast cancer: A concept analysis**

Jasleen Farwaha, Monakshi Sawhney, Kevin Woo

Queen’s University

**Introduction/Aim**: Breast cancer is a commonly diagnosed malignancy in developed countries. Women who receive surgical treatment for breast cancer often experience acute and chronic pain. Moderate to severe acute pain has been reported in 68% of women after breast cancer surgery, and 57% of these women developed persistent post-surgical (PPS) pain. The pain experience is unique; therefore, the purpose of this concept analysis is to explore the antecedents, core attributes, and consequences of post-surgical pain in women with breast cancer. The objective is to identify potential care strategies to mitigate post-surgical chronic pain.

**Methods**: This analysis followed the Walker and Avant (2011) method. Online databases that were searched included the following terms: “breast cancer,” “pain,” “chronic,” “post,” “surgical,” “women,” “nursing,” “causes,” “persistent pain,” “acute pain,” “cancer pain, “risk,” “factors,” “post-operative” between the years of 2012 and 2022.

**Results**: Persistent post-surgical breast cancer pain is classified as traumatic nerve injury and inflammation. Pain is the result of pre-surgical pain, stress, anxiety, and socioeconomic risk factors (younger age, women of colour, being less educated, and having a lower annual income). Consequences include decreased quality of life, less enjoyment of life, depression, and reduced income.

**Discussion/Conclusions**: Healthcare providers play an essential role in alleviating post-surgical pain by addressing stress and anxiety and paying attention to vulnerable populations.

**Living with an ostomy: A needs assessment in pediatric inflammatory bowel disease**

Meghan Ford^a^, Dean Tripp^a^, Ashley Cruden^b^, Inez Martincevic^b^, Thomas Walters^b^ and Sara Ahola Kohut^b^

^a^Queen’s University; ^b^Hospital for Sick Children

**Introduction/Aim**: Inflammatory bowel disease (IBD) is a chronic, immune-mediated inflammatory condition of the digestive tract associated with pain. Treatment is often focused on medications but may also include surgical approaches (e.g., intestinal ostomy). Scientific literature regarding the psychosocial implications of ostomy surgeries is scarce, and even less is known about young people’s experiences which may differ markedly from adult experiences. The purpose of this study was to explore, from the perspectives of youth and their respective caregivers, what their needs and preferences are when they have undergone, are anticipating, or have anticipated the possibility of undergoing ostomy surgery.

**Methods**: A purposive sample of IBD patients between the age of 10 and 18 years old and their caregivers were recruited from the Hospital for Sick Children and completed a background questionnaire and semi-structured interview. Data analysis followed an inductive qualitative content analysis approach.

**Results**: Twelve patients (M=15y7m, SD=3y7m) and thirteen caregivers (M=47y5m, SD=2y5m) participated in the study. Qualitative results identified five themes: 1) feelings about the ostomy (e.g., fear of surgery, body image), 2) living with an ostomy (e.g., taking care of an ostomy, gap in care), 3) relationship to IBD team (e.g., shared decision making, limited time to make decision), 4) considering social effects (e.g., representation, openness and disclosure), and 5) recommended action items (e.g., resources, communication).

**Discussion/Conclusions**: Findings highlight several important clinical implications for IBD youth including the importance of communication with patients and their families.

**Supporting youth in school with chronic pain: A scoping review**

Aishwarya Gannamani^a^, Amanjot Kaur^a^, Kathryn Birnie^b^ and Krista Baerg^a^

^a^University of Saskatchewan; ^b^University of Calgary

**Introduction/Aim**: Approximately 15-20% of children have chronic pain. Youth with chronic pain face challenges in school resulting in higher rates of arriving late to, leaving early from, or entirely missing school. The purpose of this scoping review is to identify classroom accommodations or strategies that support youth in school with chronic pain.

**Methods**: Arksey & O’Malley’s five-step framework for scoping reviews and the PRISMA extension for scoping reviews (PRISMA-ScR) were used to guide our study. Literature searches in MEDLINE, EMBASE, CINAHL, PsycINFO, and ERIC were used to identify relevant studies published before June 2021. Articles were included if they discussed strategies, accommodations, or supports in the school setting for children with chronic pain aged 5-17 years old enrolled in kindergarten to grade 12. Primary outcomes identified were school attendance, school functioning, and academic achievement. Data extracted included study aim, study population, intervention used, outcome measures, and key findings.

**Results**: 6850 publications were identified in our database search; after removal of duplicates 4628 publications were screened for inclusion in our project. We identified 12 papers eligible for inclusion in our study with 6 studies reporting academic achievement outcomes, 8 studies reporting school functioning outcomes, and 9 studies reporting school attendance outcomes.

**Discussion/Conclusions**: Despite the large body of evidence supporting multidisciplinary chronic pain management, there is little that directly addresses how to support youth functioning in school. School attendance, school functioning, and academic achievement are essential components of youth development and evidence-based strategies to accommodate and support youth with chronic pain in school are needed.

**Virtual reality (VR) as an adjunctive pain management therapy in palliative cancer patients with chronic pain**

Bernie Garrett, Tarnia Tarverner, Gordon Tao, Crystal Sun

University of British Columbia

**Introduction/Aim**: Research exploring Virtual Reality (VR) for pain therapy demonstrates growing work examining acute pain applications, but with little work to date in the field of chronic pain and palliative care.

**Methods**: A randomized controlled trial (RCT) was implemented. Cancer patients with a diagnosis of ongoing chronic pain (*N*=100) were recruited. Participants were split into two blinded groups and undertook either a VR experience of 30 minutes daily for 6 days a week in their own homes using a VR head-mounted display, or the same experiences on a laptop. Participants completed pre, during and post pain scores using the numerical rating scale (NRS). Additionally, each week of the study each participant completed: a) the McGill Pain Questionnaire, b) an overall health survey, and c) a quality of sleep score at the end of each week.

**Results**: The RCT is currently concluding and results will be presented. Quantitative ANOVA and logistic regression for group performance over time were also undertaken as an interim analysis at the midpoint (n=52) and a qualitative data analysis of patient experience (focus group: n=7). This demonstrated a positive impact of adjunctive VR therapy in many participants as a powerful distraction therapy, but with no significant ongoing effects demonstrated, or difference between screen-based and VR implementations. However, participants reported a distinct benefit for their pain management approaches including the significant impact of being pain-free during the VR experience for many.

**Discussion/Conclusions**: This work highlights the early potential for targeted VR adjunctive therapies to help in the ongoing management of cancer pain for palliative care in hospital, clinic and home settings.

**Developmental trends of physiological and behavioural responses to acute pain over the second year of life**

Lojain Hamwi^a^, Dan Flanders^b^, Deena Savlov^b^, Eitan Weinberg^b^, Hartley Garfield^c^ and Rebecca Pillai Riddell^a^

^a^York University; ^b^Kindercare Pediatrics; ^c^Garfield Hartley A Dr

**Introduction/Aim**: Toddlers’ behavioral and cardiac indicators of distress can provide valuable insight to the appraisal of child pain. Longitudinal examination of how pain responses change over time can bring insight as to nuances within developmental stages, such as toddlerhood. The current study aimed to examine the development of toddlers’ behavioural and physiological pain responses reflected by pain-related facial actions and cardiac indicators respectively in toddlerhood.

**Methods**: 12- (*N*=136), 18- (*N*=114) and 24-month-old (*N*=47) infants had video recordings and physiological data collected and synchronized during their routine immunization appointments. Toddlers’ pain-related facial expressions were coded using NFCS as present or absent on a second-by-second basis for the first 10-second epochs at pre-needle, immediately after needle, 1-minute post-needle and 2-minutes post-needle. HR and respiratory sinus arrhythmia (RSA; RSA reflects the heart rate in relation to respiration) will be examined over 30 second epochs overlapping the same four epochs.

**Results**: Descriptive analyses of developmental trends revealed differences in behavioural distress responding and cardiac reactivity during immunization. Toddlers at all ages experienced drop in NFCS scores between the 10 seconds post-needle and one-minute post-needle. Facial expression scores were highest for 12-month-olds immediately post-needle. 12-month-olds also had the highest HR at all three time points post-needle, with little variability between 18- and 24-month-olds. RSA was consistent across age groups and across vaccination epochs.

**Discussion/Conclusions**: Findings from this study explore the developmental differences in behavioral and cardiac indicators of distress regulation across the second year of life. This research will contribute to enhancing clinical pain assessment tools and interventions for pediatric pain management.

**Patient expectations and understanding of diagnosis entering an interdisciplinary chronic pelvic pain program: A cross-sectional study**

Caitlin Anne Jago^a^, Caroline Lachance^b^, Vishal Varshny^c^, Maryam Nasr-Esfahani^a^ and Magali Robert^a^

^a^University of Calgary, Calgary Chronic Pain Centre; ^b^Centre Hospitalier de l’Université de Montréal (CHUM); ^c^Providence Healthcare

**Introduction/Aim**: Few studies have looked at patient expectations and beliefs around diagnosis and management when entering a chronic pain program. This can lead to a disparity between provider care and patient expectations, leaving patients frustrated. To date, patient expectations and understanding of diagnosis prior to engaging in an interdisciplinary pelvic pain program have not been explored.

**Methods**: Cross-sectional study of women enrolled in the Calgary Chronic Pain Centre pelvic pain program up to and including May 2019. Data was extracted from intake questionnaires and analyzed using descriptive statistics. Expectations and diagnoses were clustered in themes, expressed as proportions, ranked in order, and dichotomized as biological only or biopsychosocial approach. Student’s t-test compared biological and biopsychosocial approach in respect to PDI and EQ-5D results, and compared self-reported diagnoses with treatment expectations between groups.

**Results**: For perceived diagnosis: 74% reported a gynecologic cause for their pain, 25.7% reported musculoskeletal causes, and 21.9% reported other health conditions. For treatment expectations: 46.6% believed they required rehabilitation, 30.8% responded “I don’t know,” and 21.2% reported perceived need for medication. The most reported co-morbidity was mental health (39%). Average perceived required reduction in pain to “do things that are most important” was 72%.

**Discussion/Conclusions**: Most patients can identify a perceived cause of pain, but there is uncertainty about treatment. Understanding patient perspectives and expectations helps to establish a patient-provider relationship, which can validate patient beliefs, provide education about the biopsychosocial approach to management, and ensure patients feel respected and engaged in their care.

**Cortisol as an indicator of pain and distress following acute musculoskeletal trauma**

Joshua A. Jesin and David M. Walton

Western University

**Introduction/Aim**: A growing number of prognostic screening tools for chronic pain development following MSK trauma have been published that broadly focus on metrics of cognition (i.e. beliefs/expectations) about the trauma and resulting symptoms. One such tool is the Traumatic Injuries Distress Scale (TIDS) that measures pain, negative affectivity and hyperarousal. Here we explore whether cortisol levels can help explain the physiologic mechanism by which cognitions successfully predict pain outcomes.

**Methods**: Data for these analyses were drawn from a clinical trial in London, Ontario that recruited 130 participants presenting to hospital with pain related to a recent non-catastrophic MSK trauma. Cortisol was measured from participant saliva, hair, and blood samples. In addition to validated pain and distress questionnaires, metadata such as age, sex, BMI, adverse childhood events, pre-trauma stress levels and pre-existing physical/psychological comorbidities were collected.

**Results**: We found no significant associations between cortisol levels and TIDS in isolation. However when stratified by metadata, the hair cortisol and TIDS bivariate association became evident in young age and low pre-trauma stress subgroups. Through hierarchical regression analysis we found addition of ‘cortisol X metadata’ interaction terms significantly improved TIDS prediction (Age: ∆R2=15.1%; Pre-trauma stress: ∆R2=9.1%).

**Discussion/Conclusions**: Our findings are consistent with preliminary evidence of a curvilinear relationship between hair cortisol and the Perceived Stress Scale, where positive linearity is present in low-stressed individuals. In this exploratory study we identified several metadata variables with expected relevance to analysis or confounder characterization in subsequent longitudinal studies of the physiological basis of baseline pain and distress in chronic pain development.

**Understanding the role of resiliency in the relationship between disease activity, pain catastrophizing, and negative affect in individuals with inflammatory bowel disease**

Krista Jones^a^, Aida Fernandes^b^, Lesley Graff^c^, Sara Ahola Kohut^d^, Kim Daley^e^, Paul Moayyedi^f^, Deborah A. Marshall^g^, Valerie Taylor^h^, Sandra Zelinsky^e^, Gail Bellissimo^i^, Mark Swain^g^, Luciano Minuzzi^f^ and Dean Tripp^a^

^a^Queen’s University; ^b^McMaster University, IMAGINE Network; ^c^University of Manitoba; ^d^The Hospital for Sick Children; ^e^University of Calgary, IMAGINE Network; ^f^McMaster University; ^g^University of Calgary; ^h^University of Calgary, Alberta Health Services; ^i^IMAGINE Network

**Introduction/Aim**: Inflammatory bowel disease (IBD) is a chronic and often painful condition that is associated with increased levels of depressive symptoms. The aim of the current study was to understand the role of resiliency in the associations between disease activity, pain catastrophizing, anxiety, and depressive symptoms, and whether these relationships differ by IBD subtype.

**Methods**: Adult participants enrolled in the IMAGINE Network’s Mind and Gut Interactions Cohort (MAGIC) study were included in the current study if they had a diagnosis of Crohn’s disease (*n*=1267), ulcerative colitis (*n*=876), or unclassified IBD (*n*=32). Multiple groups analysis was first conducted in lavaan. The model was rerun using the PROCESS syntax for R Model 92 to get the conditional or simple effects.

**Results**: The path coefficients for the three groups (Crohn’s disease, ulcerative colitis, unclassified IBD) did not differ significantly, *p*=.108. For pain catastrophizing, the disease activity X resilience interaction was not significant, b=.005, t(2171)=0.707, *p*=.479. Conversely, for anxiety, both the disease activity X resilience, b=-0.028, t(2169)=3.411, *p* < .001, and the pain catastrophizing X resilience, b=-0.078, t(2169)=3.050, *p*=.002, interactions were significant. For depression, anxiety X resilience was the only significant interaction, b=-0.047, t(2167)=2.263, *p*=.024.

**Discussion/Conclusions**: Mechanisms examined in the current study did not differ based on IBD subtype. The effects of pain catastrophizing and anxiety on the association between disease activity and depressive symptoms are tempered by resiliency.

**A qualitative study of the pain experiences of children and their parents at a children’s hospital**

Elise Kammerer^a^, Joshua Eszczuk^a^, Katie Caldwell^a^, Jacob Dunn^a^, Sharon Appelman-Eszczuk^b^, Jennifer Dunn^b^ and Samina Ali^a^

^a^University of Alberta; ^b^Alberta Health Services

**Introduction/Aim**: Children often experience pain in the hospital. Pain care is best provided when children and their families are actively involved in their care. We need to learn more about families’ experiences in being involved in children’s pain management.

**Methods**: Twelve child-parent dyads were recruited from the Stollery Children’s Hospital (Edmonton, AB) to participate in the study by scanning a QR code on a poster that was posted throughout the hospital from January to August 2021. Children and their parents chose whether to be interviewed together or separately. Transcripts were analyzed using inductive, data-driven codes. Codes and themes were developed by three coders using a codebook and member-checking.

**Results**: We identified three main themes: a. Painful experiences can have a significant positive or negative effect on children’s and parents’ lives and healthcare trajectories; b. There can be a mismatch between children’s and parents’ expectations of pain management and how they perceive the pain was managed; and c. Children and their families feel that they must advocate for better pain care, but often feel too intimidated to do so, or worry that their concerns will be dismissed by healthcare professionals.

**Discussion/Conclusions**: Families want healthcare professionals to proactively manage their children’s pain, to support the shaping of early positive memories of a child’s healthcare interactions. Healthcare providers must further recognize that untreated or under-treated pain can significantly impact children’s and families’ lives, and should both seek and be receptive to child and family input for better pain care.

**A standardized methodology for social media-based recruitment within a pediatric chronic pain sample**

Rachel Kelly^a^, Vina Mohabir^a^, Fareha Nishat^a^, Tieghan Killackey^a^, Chitra Lalloo^b^, Melanie Noel^c^, Kathryn A. Birnie^d^ and Jennifer Stinson^e^

^a^The Hospital for Sick Children, Peter Gilgan Centre for Research and Learning; ^b^The Hospital for Sick Children, Peter Gilgan Centre for Research, Institute of Health Policy, Management & Evaluation; ^c^Alberta Children’s Hospital, Alberta Children’s Hospital Research Institute, University of Calgary; ^d^University of Calgary; ^e^The Hospital for Sick Children, University of Toronto

**Introduction/Aim**: The use of social media to recruit youth for research has become increasingly common. Social media provides a unique platform to potentially reach a diverse sample of participants. However, this approach can be subject to ineligible individuals seeking study reimbursement. The goal of this research is to utilize a standardized tool for identifying and excluding sham participants (i.e., individuals misrepresenting eligibility or software automated enrollment) in an efficient, cost-effective manner to ensure study validity.

**Methods**: An exclusion hierarchy was developed in the recruit for the Power over Pain study, which includes four stages of criteria: (1) Expression of Interest; (2) Information Verification; (3) Demographics and Pain Verification; and (4) Screening Call. Each stage consists of various questions designed to verify whether participants are truly eligible before enrollment into the study.

**Results**: Within the first three weeks of recruitment, 826 individuals indicated interest in the study. With the use of our hierarchy, 779 individuals were excluded in stages 1 and 2, and an additional 28 participants were excluded in stages 3 and 4. Of these fraudulent cases, 88% [717/807] were bots (programmed repetitive responses), 7% [58/807] provided invalid information, and 2% [15/807] demonstrated inconsistent/suspicious responses. Following this exclusion hierarchy, we were able to confidently include 12 of 826 respondents (1.4%).

**Discussion/Conclusions**: The exclusion hierarchy served as an accurate and effective tool to identify a significant number of sham responses in a timely manner. Lessons learned from implementing this tool suggest that there may be potential for implementing standardized methodologies for social media-based recruitment.

**Clinical impact of two different doses of oral melatonin on preoperative anxiety and postoperative pain relief -a randomized trial**

SHASHI KIRAN^a^, Sreevani Narala^b^ and Mamta Jain^c^

^a^Pt B D Sharma,Post graduate sciences of medical sciences; ^b^ESI Medical college; ^c^Pt BD Sharma post graduate institute of medical sciences

**Introduction/Aim**: Melatonin, a naturally occurring hormone, maintains the circadian rhythm, has anxiolytic, sedative and analgesic effects without significant side effects. Hence, we planned to study clinical impact of oral melatonin on preoperative anxiety and postoperative pain relief.

**Methods**: Sixty three adult patients undergoing lower limb orthopedic surgeries under spinal anaesthesia were enrolled and randomly allocated into three groups. Group M3 received melatonin 3mg, Group M6 melatonin 6mg and Group P received placebo orally night before and 1hr before surgery. Anxiety, pain and insomnia were assessed preoperatively and upto 72hrs postoperatively. The time to first analgesic request, 72hrs analgesic consumption, sedation and nausea scores were also noted.

**Results**: Lower anxiety scores were observed in group M6 both preoperatively and postoperatively upto 36hrs as compared to group M3 and P. Similarly, time to 1st and 2nd rescue analgesic request was longer and visual analogue scale scores for pain were lower in group M6 as compared to group M3 and P. However, the differences were statistically insignificant (p > 0.05). We also observed that slight reduction in analgesic consumption in group M6 as compared to group M3 or P. Also, patients in melatonin groups had slightly higher sedation scores compared to placebo but no effect on perioperative insomnia was observed.

**Discussion/Conclusions**: Premedication with 6mg oral melatonin seems to be more effective for preoperative anxiety and postoperative pain relief as compared to 3mg melatonin and placebo. Also, further studies with higher and safe doses of melatonin are warranted to obtain the optimal dose of melatonin for premedication.

**The sociocultural context of adolescent pain: Gender and racial/ethnic identity in popular adolescent media’s portrayal of pain**

Queenie Li^a^, Allison Cormier^a^, Kendra Mueri^a^, Maria Pavlova^a^, Abbie Jordan^b^, Idia Thurston^c^ and Melanie Noel^a^

^a^University of Calgary; ^b^University of Bath; ^c^Texas A&M

**Introduction/Aim**: Media consumption increases significantly during adolescence and is a powerful agent of socialization. Inaccurate representation of identity groups and phenomena are shown to have deleterious effects on how adolescents understand and enact social norms. Past research exploring popular media targeted towards children aged 4–6 revealed narrow and maladaptive portrayals of pain, as well as stereotypical pain depictions among girl and boy characters. These worrisome trends are thought to contribute to pain-related stigmas and reinforce gendered expectations of how pain is experienced and responded to in childhood. Pain problems typically emerge in adolescence and disproportionately affect marginalized individuals; however, it remains unclear how pain is portrayed as a function of gender and racial/ethnic identity in media consumed by adolescents.

**Methods**: A cross-section of adolescent media was chosen based on popularity. Selections included 10 movies and the first season of six streaming television shows. Pain portrayals were captured using two established behavioural coding schemes assessing sufferer pain characteristics and observer responses (e.g., empathy, prosocial responding, ignoring). Descriptive statistics were used to characterize pain depictions, and differences in pain experiences and responses were analyzed based on character gender and racial/ethnic identity.

**Results**: Across 60 hours of media, 616 pain instances were observed. Most sufferers showed no response to pain events (69%) and prosocial responses from observers were exceedingly rare (13%). Male characters experienced the majority of pain instances (77%), were hurt more severely, and were more likely to be mocked for their pain than female characters. Female observers displayed more empathy than male observers, exhibiting more concern for male than female sufferers. Likewise, male observers demonstrated more concerned for female sufferers, who were more often helped than male sufferers. Representation of gender identities outside the binary was absent. The racial/ethnic analyses showed that BIPOC sufferers were far less represented than white sufferers (22% vs. 78%). BIPOC sufferers were more likely than white sufferers to experience pain via victimization. Moreover, white observers attempted to understand the distress of white sufferers to a greater degree than that of BIPOC sufferers.

**Discussion/Conclusions**: Our findings highlight that the pain of historically marginalized groups is underrepresented in popular adolescent media. Given that research shows underrepresentation suggests lower social value, marginalized individuals may be internalizing messages that their pain is infrequent and less real. Additionally, current pain portrayals perpetuate heteronormative and patriarchal stereotypes of the heroic, stoic male and helpless, emotional female sufferers.

**Examining self-regulation factors in early childhood and future chronic pain using a longitudinal population-based birth cohort study (All our families)**

Tatiana Lund, Jaimie K Beveridge, Susan Graham, Sheri Madigan, Kathryn A. Birnie, Sheila McDonald, Melanie E. Noel and Suzanne C. Tough

The University of Calgary

**Introduction/Aim**: Due to its high prevalence and often lifelong and devastating impact, efforts to understand how chronic pain can be prevented before it begins are essential. There is evidence to suggest self-regulation factors (i.e., attention, emotional self-control and executive functioning) are implicated in the maintenance of pediatric and adult chronic pain. In the absence of prospective data, it remains unknown whether these early developmental factors increase risk for children’s later development of chronic pain. Longitudinal birth cohort studies are ideally suited for predictive modelling to elucidate these factors.

**Methods**: All Our Families, a longitudinal, population-based birth cohort study comprised the sample (N=1990, 48% girls). When children were 5 years old, mothers completed measures of an overall construct of Child Self-Regulation used in previous research, composed of the Emotional Self-Control, Executive Functioning and Attention Problems Subscales of the Behavioral Assessment System for Children, Second Edition (BASC-2) Parent Rating Scale. These factors were entered into a multivariable Logistic Regression model as predictors of the odds of children’s chronic pain (e.g., chronic stomach aches) at age 8 years.

**Results**: 15% of mothers reported chronic pain in their children at age 8. None of the Self-Regulation constructs were significant predictors of child chronic pain in the model.

**Discussion/Conclusions**: Analyses will be re-run in early 2023 on follow-up gold standard chronic pain outcome measures administered to children at age 12-13, which corresponds to when rates of chronic pain tend to increase in early adolescence. If found to be significant, these factors may represent key targets for prevention efforts.

**Immunizing children with confidence: The power of partnerships to improve vaccine uptake through reduced needle fear and pain**

Megan MacNeil^a^, Paula Robeson^b^, Rachel Martens^c^, Christine Chambers^d^, Emily Gruenwoldt^b^ and Kathryn Birnie^a^

^a^University of Calgary / Solutions for Kids in Pain; ^b^Children’s Healthcare Canada / Solutions for Kids in Pain; ^c^CanChild Centre for Childhood Disability Research; ^d^Dalhousie University / Solutions for Kids in Pain

**Introduction/Aim**: Vaccine hesitancy contributes to poor vaccine uptake and fear of pain and discomfort related to needles is a common contributor. Up to 60% of children and 24% of caregivers report needle fear. The impact of unmanaged needle pain and fear can result in the avoidance of future healthcare. Equity-seeking groups, including children with medical vulnerability, are at higher risk for poor pain management and face greater barriers to vaccine uptake.

**Methods**: Solutions for Kids in Pain (SKIP) partnered with Children’s Healthcare Canada through the Immunizing Children with Confidence project, funded by the Public Health Agency of Canada (PHAC), to develop evidence-based resources to address needle fear and pain in children.

**Results**: Our engagement with partners led to the development of two resource summaries, ten webinars and Q&As, two articles, two conferences, videos with PHAC’s Chief Medical Officer of Health, one rapid review report, one toolkit, and an online resource hub for families, health professionals and decision-makers. The updated Children’s Procedural Pain Toolkit includes information and resources on vaccine related needle fear and pain to support conversations with families. A rapid scoping review identified factors contributing to vaccine uptake in children with medical vulnerability and actionable recommendations for health professionals, decision-makers and families.

**Discussion/Conclusions**: These resources include SKIP’s most downloaded tools and reached more than half a million people in Canada this past year. Our activities showcase the power of partnerships and knowledge mobilization for evidence-based and equitable pain and distress management for vaccination in children.

**Income Inequity and the Financial and Healthcare Burdens of Pediatric Chronic Pain**

Mica Gabrielle Marbil, Jaimie Beveridge, Sabine Soltani, Jillian Miller, Melanie Noel and Kathryn Birnie

University of Calgary

**Introduction/Aim**: Research has shown that lower socioeconomic status poses a risk factor for chronic pain. However, it is unknown whether pediatric chronic pain differentially impacts families with lower income. Addressing inequity in pain is a goal identified by the Canadian Pain Task Force in 2021. We examined the characteristics of families of youth with chronic pain with varying income, and investigated whether medical expenses and healthcare utilization differ by income group.

**Methods**: Caregivers (N=295) of youth attending a Canadian outpatient pediatric pain clinic reported on self and youth demographic characteristics, annual household income, and youth pain-related medical expenses and healthcare utilization (i.e., number of visits to general practitioners, specialists, non-physician care providers, emergency departments, inpatient admissions) in the past three months. Chi-squares and t-tests analyzed group differences.

**Results**: Proportionately more caregivers with income < $90,000 identified as non-White, χ2(1)=10.24, *p*=.001, were not in a relationship, χ2(1)=53.60, *p* < .001, and reported chronic pain themselves, χ2(1)=4.54, *p*=.033, compared to caregivers with income > $90,000. Although proportionately more caregivers with income > $90,000 paid for youth pain-related medical expenses, χ2(1)=4.74, *p*=.029, costs did not differ by income group, t(99)=-1.32, *p*=.189. No significant differences in healthcare utilization were found.

**Discussion/Conclusions**: Caregivers differing in income reflect other social characteristics that can impact pain and its treatment (e.g., family constellation, minority status). Importantly, the similarity in pain-related expenses and resources between income groups shows a lack of equitable financial and systemic support for pain, potentially resulting in greater burden for families of youth with chronic pain and of lower income.

**Drawing from the evidence: The first step towards a decision aid for in-person vs virtual care for pediatric chronic pain**

Mica Gabrielle Marbil^a^, Nicole MacKenzie^b^, Sabine Soltani^a^, Diane Lorenzetti^a^ and Kathryn Birnie^a^

^a^University of Calgary; ^b^Dalhousie University

**Introduction/Aim**: Virtual healthcare is common for chronic pain (CP), but might be neither preferable nor accessible to all patients, particularly for marginalized groups. Shared decision-making (SDM) allows patients and health professionals to collaborate on treatment decisions based on evidence and patient preferences, potentially improving patient outcomes. As phase 1 of a project to develop a decision aid for in-person versus virtual care, this systematic review examined evidence on in-person versus virtual interventions for pediatric CP, with a secondary aim focusing on marginalized groups.

**Methods**: We searched CINAHL, EMBASE, MEDLINE, APA PsycINFO, and Web of Science for randomized controlled trials (RCTs) published since 2012 that directly compared single and/or multimodal interventions delivered in-person versus virtually for youth (< 18 years) with CP. Comparisons of virtual interventions to non-equivalent treatments (e.g., standard care) were excluded.

**Results**: Title and abstract screening were completed for 3630 articles, and 24 full-text articles were screened. Only 2 studies met inclusion criteria. Both RCTs compared psychological interventions (i.e., mindfulness, cognitive behavioural therapy) delivered in-person versus virtually (i.e., telephone and virtual), and included mostly white participants (72-84%). No difference in efficacy was found.

**Discussion/Conclusions**: Currently, minimal evidence compares in-person versus virtual care for pediatric CP, and marginalized populations are inadequately represented. Research that directly evaluates in-person versus virtual care and engages diverse groups is essential to inform equitable evidence-based treatment and guide SDM for pediatric CP. The project’s next steps include surveying pediatric pain clinics on decision-making resources and interviewing patients and health professionals to develop the decision aid.

**Impact of dexmedetomidine in conjunction with a weaning protocol on postoperative opioid use in a neonatal intensive care unit**

Deonne Dersch-Mills^a^, Alixe Howlett^a^, Jan Lind1^a^ Allison Marchuk^b^ and Khorshid Mohammad^a^

^a^Alberta Children’s Hospital; ^b^Mount Royal University

**Introduction/Aim**: To describe the impact of a protocol using dexmedetomidine (and clonidine) on opioid exposure in neonates postoperatively.

**Methods**: A retrospective chart review of surgical patients receiving opioids and alpha-2-agonists (dexmedetomidine and/or clonidine) postoperatively was performed over 2 time periods: one with and one without weaning protocols in place. Comparisons of opioid duration/exposure, NICU outcomes, and protocol process measures were made.

**Results**: Clinically, there were reductions in opioid wean duration (240 vs 227 h, *p*=0.82), total opioid duration (604 vs 435 h, *p*=0.23), and total opioid exposure (91 vs 51 mg ME/kg, *p*=0.13), although the improvements did not achieve statistical significance. A limited impact on NICU outcomes or pain/withdrawal scores with use of the protocol was noted, while there was an increase in the use of medications in alignment with the protocol (ie. scheduled acetaminophen).

**Discussion/Conclusions**: In previous work, we were unable to demonstrate a statistically significant reduction in opioid exposure with use of alpha-2 agonists alone in neonates postoperatively. The addition of a weaning protocol has shown some clinically, but not statistically, significant benefits. One limitation of this study is the number of post-protocol patients. The inclusion of additional patients may have resulted in statistically significant results. At this time, dexmedetomidine and clonidine should not replace other approaches such as standardized protocols and scheduled acetaminophen postoperatively as further study is needed.

**Brain volume changes following the addition of brain stimulation to intensive interdisciplinary pain treatment in youth with chronic pain**

Meghan McNaughton^a^, Spencer Epp^a^, Adam Kirton^a^, Nivez Rasic^a^, Catherine Lebel^a^, Frank MacMaster^b^, Melanie Noel ^a^, Laura Rayner^c^, Joanne Vallely^c^ and Jillian Vinall Miller^a^

^a^University of Calgary; ^b^Dalhousie University; ^c^Alberta Children’s Hospital

**Introduction/Aim**: Intensive interdisciplinary pain treatment (IIPT) was developed to help youth with chronic pain (pain > 3 months) and moderate to severe functional disability manage their pain and return to normal daily activities. IIPT programs typically involved psychology, physiotherapy, and anesthesia therapeutic teams. In 2020, we added repetitive transcranial magnetic stimulation (rTMS) to IIPT. Here we investigate brain volume changes pre- to post-IIPT, before and after adding rTMS to IIPT.

**Methods**: 24 youth underwent IIPT alone, and 11 youth underwent IIPT with rTMS. All youth underwent MRI scanning, including T1-weighted imaging, at baseline and discharge from IIPT. T1-weighted scans were parcellated into total grey, subcortical grey and white matter volumes. For the rTMS group, the coil was applied to the DLPFC at 10Hz for 40 threshold pulses over 4s with an inter-train of 26s. rTMS sessions lasted 37.5 min for 5d/wk for 3 wks. Paired t-tests were used to compare total grey, subcortical grey and white matter volumes between IIPT baseline and discharge for IIPT alone, and IIPT with rTMS groups.

**Results**: Total grey matter volume significantly increased between baseline and discharge in the IIPT with rTMS group (*p*=0.002; effect size=1.23), but not the IIPT alone group (*p*=0.30).

**Discussion/Conclusions**: Preliminary results revealed an increase in total grey matter volume in youth who underwent rTMS in addition to IIPT, which was not observed in youth who underwent IIPT without rTMS. It remains to be determined whether this volume change correlates with patient outcomes.

**Using pre-surgical sensory profiles to predict post-surgical pain-intensity outcome: Advances and challenges**

Alyssa Merbler^a^, Chantel Burkitt^b^ and Frank Symons^a^

^a^University of Minnesota; ^b^Gillette Children’s

**Introduction/Aim**: Our previous pilot study demonstrated behavioral reactivity (BR) profiles during a modified quantitative sensory test (mQST) prior to a surgery predicted post-surgical pain for participants with cerebral palsy (CP). These patterns did not hold when attempting replication with a larger sample. The purpose of this poster is to present the data from both studies and present advances and challenges in pain assessment in CP.

**Methods**: The same procedure was used for both studies. Pain response status post-surgery (e.g. decrease, increase, or no change) was determined by changes in caregiver-reported maximum pain intensity in the last week (0-10). We score observable BR (e.g. grimace, flinch) during calibrated sensory stimuli applications of the mQST (e.g. pin, cool) pre-surgery. We created z-scores of BR to each stimulus for both response groups, with participants who reported no pain pre-surgery as the reference group.

**Results**: In the pilot, the “responder” subgroup had pre-surgical sensory profiles characterized by increased BR to pin and cool (outside 95%CI of the reference group). The pre-surgical sensory profile for the “non-responder” subgroup had reduced BR for cool and repeated von Frey. The replication sample did not have significantly different sensory profiles.

**Discussion/Conclusions**: These results demonstrate the ability to compare sensory reactivity in CP. Participant variability within subgroups, the “right” comparison group (e.g. no pain, responders), and the appropriate pain outcome (e.g. intensity, chronic pain) present challenges to replication and the study of pain/sensory function in CP. These considerations must be addressed to understand sensory function and pain in CP.

**The intergenerational transmission of chronic pain in Canadian armed forces serving members/veterans and their young adult children**

Kendra Mueri^a^, Samantha Noyek^a^, Maria Pavlova^a^, Tatiana Lund^a^, Tom Hoppe^b^, Robert O’Connor^b^, Christine O’Connor^c^, Jessica Clemens^b^, Gordon Asmundson^d^, Richelle Mychasiuk^e^, Jennifer Stinson^f^ and Melanie Noel^a^

^a^University of Calgary; ^b^Chronic Pain Centre of Excellence; ^c^Chronic Pain Centre Of Excellence; ^d^University of Regina; ^e^Monash University; ^f^University of Toronto, The Hospital for Sick Children

**Introduction/Aim**: Chronic pain and mental health issues are alarmingly prevalent in veterans, possibly placing their children at risk for the development of pain problems. It is imperative to establish the prevalence and nature of pain in children of Canadian veterans and understand mechanisms underlying the transmission to chronic pain. This study aims to examine the pain experiences and mental health of Canadian Armed Forces (CAF) serving members/veterans and their children.

**Methods**: CAF members/veterans enrolled in any of the military elements after 1975 and their child aged 10 to 40 years were eligible to participate. Eighty-eight CAF members/veterans (M=52.3 years, SD=7.4; 73% male) and 51 of their children (M=17.1 years, SD=5.8) completed measures assessing pain and mental health symptoms (e.g., PTSD).

**Results**: Preliminary results indicate that 99% of members/veterans and 45% of their children reported chronic pain (pain ≥ 3 months). Members/veterans reported an average pain severity of 6.3/10, whereas children’s average pain severity was 4.2/10. More severe PTSD symptoms reported by members/veterans were significantly associated with higher levels of pain intensity, r=.28 p=.010, pain unpleasantness, r=.43, *p*=.001, and pain interference, r=.45, p < .001. Further, parent PTSD symptoms were significantly associated with youth pain unpleasantness, r=-.33, *p*=.027.

**Discussion/Conclusions**: These findings provide an opportunity to better understand the experiences of chronic pain within veteran families, which can help to minimize challenges faced by families and break the intergenerational cycle of pain and mental health concerns.

**A longitudinal examination of optimism and pain self-efficacy in youth with inflammatory bowel disease**

A. Natisha Nabbijohn^a^, Alain Stintzi^b^, David Mack^c^, Kieran O’Doherty^a^ and C. Meghan McMurtry^a^

^a^University of Guelph; ^b^University of Ottawa; ^c^Children’s Hospital of Eastern Ontario

**Introduction/Aim**: Pain is a common symptom of inflammatory bowel disease (IBD). Longitudinal work using a resiliency lens is important for intervention and improved health among those with IBD, contributing to lower healthcare costs. The current objectives are to: (1) describe resiliency factors of optimism and pain self-efficacy, as well as average pain, in youth with IBD over the first 12 months post-diagnosis and (2) examine if optimism at the time of diagnosis predicts pain self-efficacy at 4- and 12-months post-diagnosis.

**Methods**: Within a larger study aiming to examine psychosocial and biomedical variables within an observational, multi-time point longitudinal design, 77 newly diagnosed patients with IBD aged 8-16 were included. Participants completed questionnaires at the time of diagnosis (baseline) and at 4- and 12-month follow-ups. The Youth Life Orientation Test and the Child Self-Efficacy Scale were used. Average pain was rated using a numeric rating scale from 0-10.

**Results**: Using one-way repeated measure ANOVAs, optimism, pain self-efficacy, and average pain were each stable across time points (all *p* > .05). Correlations reveal positive associations between average pain and pain self-efficacy at all time points (r=-.24-.48, *p*=< .05). Multiple regression analysis controlling for sex, age, and average pain revealed that optimism at baseline significantly predicted self-efficacy at 4 and 12 months (F(3, 67)=10.5, *p* < .001).

**Discussion/Conclusions**: Our work indicates that early interventions for optimism may promote more resilient trajectories in youth with IBD and thereby offer a new pre-emptive approach to their care strategy for pain management.

**Youth perceptions of opioids for pain management - Intersecting stigmas & ambivalence**

Stephanie Nairn^a^, Patricia Conrod^b^

^a^McGill University; ^b^University of Montreal

**Introduction/Aim**: Despite the on-going consequences of the opioid overdose and health crises in Canada, there is surprisingly little qualitative research concerning youth perceptions of and experiences with prescription opioids for pain management. This gap is problematic because early use of prescription opioids is a risk factor for future opioid use and potential addiction later in the life course. Our research sought to understand youth perceptions of prescription opioids, youth needs with regards to opioid-related treatments, the gaps in opioid-related treatments for young people ages 15-25, and to develop resources for youth and their guardians with regards to prescription opioid use. We outline the results of our qualitative research with youth who were prescribed opioids for pain and display examples of resources for youth that have been co-developed by the Department of Surgery and the Department of Psychiatry at Sainte-Justine Children’s hospital.

**Methods**: We performed 3 focus groups (*N*=9), 5 interviews (*N*=5), and a youth summit with *N*=11 youth from Quebec who had been prescribed opioids for pain management and were in recovery from surgery for scoliosis, were in remission from cancer, or who had suffered acute injuries. We analyzed the results of the focus groups, interviews, and youth summit using an inductive thematic analysis approach. Collaborative development of resources for young people and their guardians involved on-line searches of grey literature and identification of the gaps in these resources as identified through feedback from youth.

**Results**: We found that there was ambivalence articulated by youth with regards to their perceptions of opioids for pain management. Negative experiences with opioids were articulated alongside positive experiences and ideas about prescription opioids. We also found that youth experienced imbricated stigmas – Youth often felt discriminated or judged not only with regards to their illnesses or conditions, but also simultaneously regarding their prescription opioid use. We found that youth receive very little holistic supports or even basic information about opioids during and throughout their recoveries.

**Discussion/Conclusions**: There are evidently large gaps in supports for youth throughout their recovery journeys and opportunities for the provision of psychosocial/educational interventions for both youth and their guardians. The development of resources for young people should entail a focus on the provision of non-stigmatizing and non-judgmental engagement that clarifies both the risks and benefits of opioid use in the context(s) of youth experiences and provision of information about psychosocial resources for young people as they navigate their recovery journeys.

**The characterization of pain in children and youth with cerebral palsy: A micro-longitudinal analysis**

Cara Nania^a^, Melanie Noel^a^, Laura Brunton^b^, Elizabeth Condliffe^a^, Daniel Kopala-Sibley^a^, Sandra Mish^c^, Brianne Redquest^a^ and Carly McMorris^a^

^a^University of Calgary; ^b^Western University; ^c^Alberta Children’s Hospital

**Introduction/Aim**: Pain is present in up to 75% of children and youth with cerebral palsy (CP), making it the most prevalent secondary condition. Yet, little is known about the multiple dimensions of pain, the role of mental health in the experience of pain, and the impact of pain on quality of life in youth with CP. Further, no study has investigated how pain interferes with daily functioning using an Ecological Momentary Assessment approach.

**Methods**: Forty-three youth with CP (61.2% male; Mage=11.66; SD=2.77) completed cross-sectional measures of pain, mental health, and quality of life. Following, youth completed 7 days of daily surveys measuring daily pain and functioning (i.e., “How much did pain interfere with your day?”).

**Results**: Preliminary Results. Youth reported a mean pain intensity of 3.70/10 and an average pain interference T-score of 52.25. Daily pain was endorsed by 23.3% of youth, and 46.3% reported pain location in their legs. Additionally, 51.2% of youth reported that they experience chronic pain. Pain interference and pain intensity were significantly correlated with anxiety, depression, and quality of life. Higher pain interference and pain intensity scores were associated with higher anxiety, higher depression, and a worse quality of life (p’s < .05).

**Discussion/Conclusions**: This is the first study to characterize the pain experience in youth with CP. Findings will directly inform treatment-tailoring approaches to improve outcomes for this vulnerable population, and offset a trajectory of increased mental health issues, reduced quality of life, and poor functioning and disability into adulthood.

**Photos sculpt youth stories of their major surgery journeys**

Samantha Noyek^a^, Gillian Newman^b^, Kathryn Birnie^a^ and Melanie Noel^a^

^a^University of Calgary; ^b^SPRINT Senior Care

**Introduction/Aim**: Chronic pain, defined as pain occurring for 3 months or more, affects an estimated 20% of Canadian youth, and can lead to mental health and pain problems into adulthood. One contributor to pain is memory. Children who develop negatively-biased pain memories following surgery are at risk for later developing chronic pain. This research expands upon the role of memory by exploring the journeys of youth who have undergone spinal fusion surgery, gaining a broader understanding of their lived experiences.

**Methods**: Photovoice offers a unique and novel approach, empowering participants to share what is important to them through photographs. Youth captured photos/videos in daily life and collected previously taken photos/videos from before, during, and/or after surgery. Individual qualitative interviews were conducted to expand upon photos/videos and a pain questionnaire was completed.

**Results**: Twenty youth 12-19 years old participated (Mage=16.7 years), including 19 females and 1 male. Fourteen participants (70%) reported having a current chronic pain problem (present for at least three months). One-hundred ninety-two photos and 24 videos were collected (5-25 photos collected per person). Preliminary interview themes include: i) impact of social support, ii) journey adjusting to a new body, iii) unexpected emotional turmoil, iv) seeking escape post-surgery, v) reflection on progress and strength.

**Discussion/Conclusions**: This study provides an in-depth understanding of the experiences of youth who have undergone spinal fusion surgery from their own perspective. Youth insights can improve healthcare pain and mental health management pre/post-surgery and enable youth to advocate with their voice within their major surgery journey.

**Retrospective chart review of outcomes at discharge or transition from pediatric chronic pain care**

Monica Ouellet, Amanjot Kaur and Krista Baerg

University of Saskatchewan

**Introduction/Aim**: Many people experiencing chronic pain as children often continue to have pain in adulthood and thus must make the transition from pediatric care to adult care. However, few studies have described the clinical features and outcomes of children at discharge from a tertiary care pediatric complex pain clinic or identified the risk factors for continued functional impairment at time of transition. The aim of this chart review was to describe the clinical features and referral patterns of children at discharge from a pediatric complex pain clinic.

**Methods**: Saskatchewan Pediatric Chronic Pain Registry participants aged 8-18 that completed their treatment course with the Interdisciplinary Pediatric Complex Pain Clinic in Saskatoon were identified. Pain scores (11-point numeric rating scale, NRS-11), 60-point Functional Disability Inventory (FDI) raw score, and PROMIS Pain Interference (PI) T-score were extracted from Registry data collected at clinic intake. Pain diagnosis and disposition at discharge was extracted from medical charts and linked to registry data. Descriptive analysis was completed.

**Results**: Thirty-five patients (78% female) were included in the analysis. Musculoskeletal pain was the primary source of pain (35%). The mean NRS-11 score was in the moderate range (4.8, 1-10). The mean FDI raw score was in the moderate range (25.4, 6-47). The mean PROMIS PI score was in the severe range (63.5, 40.6-78). The majority of patients (83%) returned to primary care at clinic discharge.

**Discussion/Conclusions**: Despite high rates of pain interference and functional disability at clinic intake, the majority of patients were assessed as suitable to return to primary care.

**Caregiver anticipatory worry prior to toddler vaccinations: Child and caregiver longitudinal predictors**

Ilana Shiff^a^, Dan Flanders ^b^, Eitan Weinberg^b^, Hartley Garfield^b^, Deena Savlov^b^ and Rebecca Pillai Riddell^a^

^a^York University; ^b^University of Toronto

**Introduction/Aim**: Caregiver pre-needle distress has been identified as a key contributor to children’s anticipatory distress, which is associated with patterns of high child distress that are difficult to disrupt. The aim of the present study was to examine how much of caregiver pre-needle worry in late toddlerhood (18-24 months) can be predicted by earlier (i.e., at the 12-month vaccination appointment) child behavioural and physiological pain-related distress and caregiver worry.

**Methods**: The study included parent-child dyads (*n*=112) who participated in at least two vaccinations over the 2nd year of life (toddlerhood). Dyads were a part of an ongoing longitudinal study (The OUCH Cardio Cohort) that naturalistically observed parents and toddlers during vaccinations at 12-, 18- and 24-months of age. Caregivers were asked to rate their pre- and post-needle worry on a scale from 0 to 10 at every appointment. Child behaviour on the FLACC scale and Heart Rate (parent and child; averaged over 30-second epochs) were analyzed for 2 minutes post-needle.

**Results**: Hierarchical multiple linear regression results revealed that the full model accounted for 35% of the variance in worry ratings, but the child behaviour variables did not significantly improve the model over the caregiver worry variables. Child heart-rate immediately following the needle (β=0.25, *p* < 0.05) as well as caregiver pre-needle worry (β=0.348 *p* < 0.001) at 12-months were unique predictors of caregivers’ pre-needle worry in later toddlerhood.

**Discussion/Conclusions**: Our findings highlight the importance of managing children’s distress for minimizing future caregiver worry.

**Characterizing pediatric pain patients with hypermobile Ehlers-Danlos Syndrome**

Olivia Sokol, Ardin Berger, Laura Simons and Rashmi Bhandari

Stanford Medicine School of Medicine

**Introduction/Aim**: The study aimed to characterize pediatric pain patients with Hypermobile Ehlers-Danlos Syndrome (hEDS), an understudied population.

**Methods**: Participants were 98 youth (Mage=14.76 years, SD=2.31) diagnosed with hEDS at a tertiary pediatric pain clinic and primary caregivers. Data were collected retrospectively from medical chart and PROMIS measures prior to interdisciplinary evaluation. Youth were predominantly Non-Hispanic Caucasian (57%) of female legal sex (86%); 18% identified as gender-diverse.

**Results**: Pain and functioning characteristics included: pain duration (M=43.81 months, SD=48.92), pain intensity (M=6, [0=no pain, 10=worst pain]), schooldays missed per month (M=5.26, SD=7.53), doctors seen for pain in past year (M=4). Common primary referral reasons were: EDS (26%), Other (18%), Fibromyalgia or pain amplification syndrome (10%), and Musculoskeletal pain (9%).

Co-existing symptoms/diagnoses were mast cell activation (49%), postural orthostatic tachycardia syndrome (39%), and both (24%). Common psychiatric diagnoses were: anxiety disorders (71%), psychological factors affecting medical conditions (54%), and depressive disorders (42%).

Clinical elevations on PROMIS scores for 8–17-year-olds (n=89) included: fatigue (58%), pain interference (30%), depression (24%), anxiety (18%), sleep disturbance (54%) and sleep-related impairment (43%). Majority had elevated fear of pain (FOPQ; 77%), pain-related distress (PCS-C; 70%), and at-risk school functioning (PedsQL School; 82%).

**Discussion/Conclusions**: Youth with hEDS come to tertiary clinics having struggled with pain for a long time, with significant autonomic and mental health comorbidities, elevated levels of fatigue, pain-related distress, school absences, and fear of pain.

**Pain as a risk factor for first lifetime onset of suicidality in adolescence**

Sabine Soltani, Melanie Noel, Emily Bernier, Daniel Kopala-Sibley

University of Calgary

**Introduction/Aim**: Chronic pain and mental health problems (e.g., anxiety, depression, suicidality) have both been identified as public health emergencies and co-occur at high rates in adolescence. Theoretical models have been proposed to explain the co-occurrence and interplay of these symptoms in youth; however, there is a paucity of prospective research examining the directional influence of pain in the development of first lifetime onset of mental health issues. Temporal associations between chronic pain and suicidality in particular remain remarkably understudied in youth, despite robust associations demonstrated in adults. This prospective, longitudinal investigation examined whether chronic pain status, pain-related symptoms (intensity, interference), pain catastrophizing, and insomnia severity predicted first lifetime onset of suicidality in a cohort of youth with parental history of mood and/or anxiety disorders.

**Methods**: Participants included 147 youth (Mage=13.74 years; 64% female) who completed structured diagnostic interviews at baseline and at 9- and 18-month follow-up to assess mental health symptoms, including suicidality. Participants also completed questionnaires assessing depressive and anxiety symptoms, pain symptoms and characteristics, pain interference, pain catastrophizing, and insomnia severity.

**Results**: Approximately 25% of youth reported having chronic pain at baseline, and 34% endorsed experiencing suicidality at follow-up. Chronic pain at baseline was associated with increased likelihood of onset of suicidality at follow-up, above and beyond sex and baseline depressive symptoms. Increased pain intensity and interference at baseline predicted increased severity of suicidality at follow-up.

**Discussion/Conclusions**: The presence of chronic pain and elevated pain-related symptoms are premorbid risk factors for the development of suicidality in youth.

**PTSD hyperarousal symptoms significantly differ between veterans with and without concurrent pain**

Martine Southall, Pamela Holens, Juanita Garcia, Spenser Martin, Deborah Zacharias, Jeremiah Buhler, Luigi Imbrogno

University of Manitoba

**Introduction/Aim**: Although the relationship between PTSD and pain has been well established, there is insufficient research examining this relationship using DSM-5 symptom clusters, and at the multidimensional symptom cluster level, in military populations.

**Methods**: A retrospective chart analysis of 121 Canadian Armed Forces veterans with a diagnosis of full or subclinical PSTD was conducted with the goal of examining the interaction between self-reported pain and PTSD symptom clusters. Data on to PTSD and pain symptomatology were extracted from standard intake forms at an outpatient clinic treating this population. A MANOVA was performed to determine if PTSD symptom clusters differed between veterans with pain and those without pain. Discriminant function analysis was subsequently performed to examine which symptom clusters differed between the two groups.

**Results**: The results indicated that hyperarousal was elevated in veterans with PTSD and concurrent pain compared to veterans with PTSD but no pain, suggesting this symptom cluster of PTSD has an important relationship to pain in veterans.

**Discussion/Conclusions**: Clinical implications include supporting screening for pain conditions in veterans where PTSD symptoms may be subclinical but hyperarousal is elevated. Treatments that aim to target both pain and PTSD simultaneously may see greater improvement in both conditions if hyperarousal symptoms are a prioritized treatment target.

**User-centered design of an AI-enhanced social robot in the pediatric emergency department: Child & caregiver perspectives**

Fareha Nishat^a^, Summer Hudson^b^, Alaa Khalaf^a^, Prabdeep Panesar^a^, Samina Ali^b^, Sasha Litwin^a^, Patricia Candelaria^b^, Mary Ellen Foster^c^ and Jennifer Stinson^a^

^a^The Hospital for Sick Children; ^b^University of Alberta; ^c^University of Glasgow

**Introduction/Aim**: Socially assistive robots (SARs) are a promising tool to manage pain and distress related to medical procedures [e.g. intravenous insertion (IVI)] in pediatric settings, however, current options lack autonomous (AI-enhanced) adaptability. The aim of this study was to understand children and caregivers’ perceptions toward using an AI-enhanced SAR as a distraction tool in the pediatric emergency department (ED) to facilitate IVI, thereby informing the development of such a system.

**Methods**: This study presents a descriptive qualitative needs assessment of children and caregivers. Data was collected via both semi-structured individual interviews and focus groups. Participants were recruited from two Canadian pediatric EDs between April 2021 to January 2022.

**Results**: Eleven caregivers and nineteen children were included. Three main themes were identified: (1) Experience in the ED, (2) Acceptance and concern surrounding SARs, and (3) SARs features to support child engagement. Most participants expressed comfort with robot technology, however, concerns were raised about sharing personal information, photo/videotaping, and the possibility of technical failure. Suggestions for feature enhancements included increasing movement to engage a child’s attention and tailoring the language to a child’s developmental age. To enhance the overall experience in the ED, participants also identified a role for the SAR in the waiting room.

**Discussion/Conclusions**: AI-enhanced SARs were perceived by children and caregivers as a promising tool to distract children during IVIs and to enhance the overall experience of visiting the ED. Next steps include development and usability testing of the SAR, subsequent evaluation in the pediatric ED via an RCT, and clinical implementation.

**Retrospective review of referrals to specialized chronic pain in pregnancy program**

Zaid Sweidan^a^, Elaine Teh^b^, Lucy Doan^b^, Rebecca Titman^c^, David Flamer^c^ and Matthew Sheppard^c^

^a^University of Toronto; ^b^Mount Sinai Hospital Wasser Pain Management Centre; ^c^Mount Sinai Hospital Wasser Pain Management Centre, University of Toronto

**Introduction/Aim**: Pregnant patients with underlying chronic pain disorders can often have their symptoms overlooked or inadequately addressed. This may be the result of a lack of referrals to specialized pain clinics and pregnancy related care. We sought to evaluate factors associated with referral to specialized pain care and assessed two different streams of specialized care.

**Methods**: A retrospective review of patients referred to a chronic pain in pregnancy program at a university-affiliated tertiary care center from January 2021 to June 2022 was performed. Patients were grouped based on whether they were referred to the collaborative care stream (nurse practitioner with physician support) or the PM&R stream. Patient characteristics, reasons for referral and source of referral were compared between groups.

**Results**: Overall, 116 patients were included. Between January 2022 and June 2022, 35 patients went to the NP stream and 27 to the PM&R stream. The most frequent reason for referral was MSK pain (n=31) followed by abdominal pain and medication/opioid management. Most referrals were sent from outpatient obstetrics (41%) followed by inpatient medicine/obstetrics (29%). In total, over 70% required opioid management.

**Discussion/Conclusions**: Patients referred to PM&R were much more likely to have MSK related pain whereas, patients seen in the collaborative stream tended to have more medication related issues. Overall, the majority of referrals were from outpatient obstetrics with few referrals from TAPMI and other programs . Most patients with chronic pain require adjustment of their opioids during pregnancy.

**Investigating age-related patterns in sensation detection and pain sensitivity following taxane-based chemotherapy within a biopsychosocial framework**

Frédérique Therrien^a^, Thaliane Côté-Cazes^a^, Lucia Gagliese^b^, Jennifer Gewandter^c^, Robert H Dworkin^d^, Maud Bouffard^a^, Sarah Béland^a^, Julie Lemieux^a^, Gaétan Daigle^a^, Josée Savard^a^, Michèle Aubin^a^, Sophie Lauzier^a^, Anne Dionne^a^, Pierre Gagnon^a^, Bruno Gagnon^a^, Philip L Jackson^a^ and Lynn R Gauthier^a^

^a^Université Laval; ^b^York University; ^c^University of Rochester; ^d^Rochester University

**Introduction/Aim**: Older adults may be at risk of chemotherapy-induced peripheral neuropathy (CIPN), but studies are equivocal, suffer from measurement limitations, and do not examine age-related risk within a biopsychosocial framework. This study explored the role of age, biopsychosocial factors, and their interactions on sensation detection (SD) and pain sensitivity (PS) before (T0), after (T1), and 3-months after (T2) taxane-based breast cancer treatment.

**Methods**: 94 younger (47.4 ± 9.1 years old) and 30 older (65.4 ± 4.4 years old) women underwent thermal- and vibration-SD, and thermal-PS testing in the foot and hand, and completed pain, sleep, and psychosocial measures. Medical charts provided clinical data. Stepwise multiple regression was used, and non-parametric mixed regression tested Beta-coefficient equalities when age group was significant (P≤0.05) at ≥1 assessment at the same test site.

**Results**: Older age was associated with poorer T0-, T1-, and T2-SD (P ≤ 0.01). While age was not associated with T0-PS (P > 0.05), older age was associated with greater T1- and T2-PS (P ≤ 0.05). Betas were consistent (P > 0.05). Lower T0-pain quality was associated with poorer T1-warm-SD among older (P=0.002) but not younger adults (P=0.11). Greater T1-negative pain appraisals were associated with greater T2-cold-PS among younger (P=0.007), but not older adults (P=0.75). Regardless of age, poorer post-treatment SD was associated with greater paclitaxel dose, adjuvant-treatment, poorer post-treatment functional status, and greater neuropathic pain; greater post-treatment PS was associated with adjuvant-treatment, T0-alcohol-consumption, poorer post-treatment sleep, and greater depression.

**Discussion/Conclusions**: Older adults may be at risk of thermal hyperalgesia following taxane-based treatment, highlighting necessary CIPN measure refinements. Age-related and age-independent biopsychosocial risk factors could inform treatments.

**Adversity & resilience among older adults living with chronic pain**

Natasha Gallan and Venezya Thorsteinson

University of Regina

**Introduction/Aim**: The aim of this study was to investigate adversity and resilience among older adults currently living with chronic pain. Seeing the limited literature on adversity and resilience among older adults and both chronic disease and chronic pain are most common among older adults, we chose to study the effects of adversity and resilience among this population.

**Methods**: The study was conducted according to a cross-sectional observational study design. Recruitment of participants involved both advertisement of the study’s purpose and inclusion criteria. Participants were asked to follow a link to complete a set of online questionnaires on pain, adversity, and resilience. Participants were asked to answer questions about their chronic pain, including diagnosis, pain intensity, pain-related distress, pain interference, and temporal characteristics of their pain. Furthermore, participants were asked to provide demographic information. Eligibility criteria included older adults (i.e., at least 65 years of age) living with chronic pain from across Canada. A series of stepwise regression models were conducted to test the influence of pain characteristics and adverse childhood experiences on resilience and pain-specific resilience.

**Results**: Pain characteristics (including severity, distress, interference, and temporal characteristics) was not significantly predictive of resilience or pain-specific resilience. While adverse childhood experiences were predictive of pain-specific resilience, they were not predictive of resilience. More specifically, emotional neglect was predictive of cognitive/affective positivity and physical neglect was predictive of behavioural perseverance.

**Discussion/Conclusions**: It is anticipated that the results will give insight towards adverse childhood experiences in relation to chronic pain, and the relationship between resilience or adversity relating to chronic pain in older adults.

**The impact of post-traumatic stress symptoms on cerebral perfusion and the development of pain symptomology in youth**

Linda Mai Tran^a^, Nils Daniel Forkert^a^, Nivez Rasic^a^, Neta Bar Am^a^, Catherine Lebel^a^, Daniel Kopala-Sibley^a^, Richelle Mychasiuk^b^, Melanie Noel^a^ and Jillian Vinall Miller^a^

^a^University of Calgary; ^b^Monash University

**Introduction/Aim**: Post-traumatic stress symptoms (PTSS) are symptom-level responses to trauma. Youth are more vulnerable to developing PTSS following adverse childhood experiences (ACEs). Chronic pain (pain > 3 months) is highly comorbid with PTSS; however, the mechanisms underlying this relationship are not well understood. Arterial spin labeling (ASL) quantitatively measures cerebral blood flow (CBF). Adults with chronic pain have reduced blood flow in pain-related brain regions. The extent that PTSS-related alterations in CBF are associated with increased pain symptomology in youth is unknown.

**Methods**: Participants consisted of 50 healthy youth aged 14-18 years in Alberta. Youth underwent an MRI, two times, three months apart. ASL data was also collected, and regional CBF values were obtained using an atlas-based approach. At both MRI visits, youth answered questionnaires regarding demographics, PTSS, anxiety symptoms, ACEs, and health-related quality of life (HRQOL). Paired t-tests were performed to compare mean changes in CBF between baseline and follow-up scans. Generalized estimating equations were used to determine relationships between CBF, PTSS, and HRQOL, accounting for age, sex, gender, anxiety, ACEs, and chronic pain status.

**Results**: Preliminary findings revealed that CBF increased in the left cerebral white matter from baseline to follow-up. Increased PTSS was associated with decreased blood flow in the left cerebral white matter. Decreased blood flow in the left cerebral white matter was associated with poorer HRQOL.

**Discussion/Conclusions**: Decreased CBF in the left cerebral white matter might suggest that PTSS decreases neuronal conductivity, leading to reduced HRQOL due to impaired physical, social, and school functioning.

**Caregiver ratings of toddler pain: The role of caregiver psychological predictors**

Jessica Zaffino^a^, Ilana Shiff^b^, Amy Stern^b^, Dan Flanders^a^, Eitan Weinberg^a^, Hartley Garfield^c^, Deena Savlov^a^ and Rebecca Pillai Riddell^b^

^a^University of Toronto; ^b^York University; ^c^SickKids

**Introduction/Aim**:Caregivers’ pain schemas trigger their reactions to their child’s pain-related distress, informing how they assess their child’s pain. Considering children’s limited ability to self-report pain, understanding the factors that influence pain ratings is critical.

**Methods**: One hundred and fifty-six parent-child dyads had pediatric vaccinations between 18 and 24 months of age recorded to capture pain responses before, during, and after the needle. Child pain behaviours were coded using the Face, Legs, Activity, Cry, Consolability Scale (FLACC; Merkel et al., 2002) and the Neonatal Facial Coding System (NFCS; Grunau & Craig, 1987). Caregivers rated their child’s pain immediately after the needle and reported on psychological stress during the appointment (i.e., worry levels before and after needle) and within 2 weeks of the appointment [Brief Symptom Inventory (Derogatis, 1977); Parenting Stress Index (Abidin, 1983)]. Multiple regression analyses were performed to determine predictors of pain ratings.

**Results**: The regression models for pain behaviour and parent psychoemotional behaviours were significant, with R-squared values of 0.17 and 0.19 respectively. While the overall child facial behaviour model was not significant, immediate post-needle facial behaviour was a significant predictor. When significant predictors from the first three models were entered into the final model (R-square=0. 25), caregiver psychoemotional variables, namely pre- and post-needle worry, were the only predictors that remained significant.

**Discussion/Conclusions**: In addition to the influence of child pain behaviours on pain ratings, caregivers are also heavily influenced by their own worries and stressors. Future research should continue exploring factors that may influence caregivers’ perception of child pain.

**Parent sleep and youth chronic pain: The role of parent pain-related cognitions**

Merek Zimmerman, Sabine Soltani and Melanie Noel

University of Calgary

**Introduction/Aim**: Chronic pain is highly prevalent (affecting 25% of Canadian youth), costly (19 billion USD/year), and debilitating for families. Thus, understanding predictors of long-term outcomes in youth with chronic pain is critical. Parent mental health has been found to be a significant driver of youth chronic pain; however, the role of parental sleep (a powerful influencer of mental health and cognitions) in this relationship has not yet been examined. This longitudinal study tested if parental sleep was linked to youth pain outcomes, and whether this relationship was mediated by parent pain-related cognitions that have been shown to drive fears and avoidance of pain.

**Methods**: Participants consisted of 105 parent-youth dyads recruited through tertiary chronic pain programs across Canada. Parental insomnia severity and youth 3-month pain interference were analyzed correlationally, with parental intolerance of uncertainty (IU) and pain catastrophizing (PC) acting as mediators.

**Results**: Results revealed that parent IU and PC did not mediate the relationship between parent insomnia and youth pain interference. However, parent insomnia was significantly correlated with parent IU.

**Discussion/Conclusions**: These findings indicate that though parent insomnia was not directly or indirectly linked to youth pain interference, it is tied to parental mental health through IU (a known risk-factor of poor youth chronic pain outcomes). With evidence showing the powerful influence of parental health on their child’s long-term chronic pain outcomes, it is critical to further examine how parental sleep may play a role in these relationships, and can be modified.

**Evidence, systematic reviews, guidelines, implementation science Les données probantes, les revues systématiques, les recommandations, la science de la mise en œuvreThe efficacy of psychological interventions on youth postoperative pain outcomes: A systematic review**

Rayna Anderson^a^, Brittany N Rosenbloom ^b^, Jill Chorney^c^, Samantha Noyek^a^, Rachel Kelly^b^, Estreya Cohen^b^, Jennifer Stinson^d^ and Kathryn A Birnie^a^

^a^University of Calgary; ^b^The Hospital for Sick Children; ^c^Dalhousie University; ^d^The Hospital for Sick Children, University of Toronto

**Introduction/Aim**: Approximately 20% of youth ages 6-18 undergoing surgery will develop chronic post-surgical pain (CPSP), which is associated with poorer overall quality of life. Psychological factors (e.g., child anxiety) have been found to increase the risk of CPSP. A 2016 systematic review by Davidson et al., evaluated the effectiveness of psychological interventions on post-operative pain outcomes in youth, but is now more than 5 years old. This systematic review sough to update the previous review and determine the extent of new research in the field.

**Methods**: Medline, Embase, CENTRAL, PsycINFO, and CINAHL were searched from 2016 – May 5th, 2022. The search strategy was developed through consultation with a medical librarian. Abstracts and full text articles were screened by two reviewers. Eligible studies were peer-reviewed randomized controlled trials published in English evaluating psychological interventions for acute and chronic postsurgical pain in youth aged 6-18 years old.

**Results**: The search yielded 3820 unique abstracts of which 18 full-text articles met our inclusion criteria. Fourteen of 20 additional articles were included from the original Davidson et al. review. Thus, a total of 32 articles are included. Studies include youth with a mean age of 6-15, a variety of surgeries (e.g., tonsillectomy, circumcision, spinal fusion), and a wide range of psychological interventions including distraction (e.g., music, puppet shows), preparation/education, and other cognitive-behavioural strategies.

**Discussion/Conclusions**: The efficacy of psychological interventions for pediatric postoperative pain varied with the type of intervention and pain outcome. Future research will determine factors that moderate the efficacy of various psychological interventions on pain outcomes.

**Meta-ethnography of barriers and facilitators of adherence to therapeutic exercise and health-related physical activity in individuals with musculoskeletal conditions**

Folarin Babatunde^a^, Joy MacDermid^a^, Ruby Grewal^a^, Luciana Macedo^b^ and Mike Szekeres^a^

^a^Western University; ^b^McMaster University

**Introduction/Aim**: Adherence is a complex phenomenon. The benefits of exercise interventions depend on adherence. Unfortunately, non-adherence is a persistent problem that acts as a barrier to achieving optimal health outcomes. This study sought to gain an in-depth understanding of factors associated with adherence to therapeutic exercise and health-related physical activity.

**Methods**: We conducted this meta-synthesis in three stages: literature search of five databases from and quality appraisal; data synthesis using Noblit and Hare’s framework with translation of key themes from eligible studies against the World Health Organization (WHO) model of adherence. A line-of argument was used to synthesize and interpret key concepts associated with barriers and facilitators of adherence to therapeutic exercise.

**Results**: Thirty-seven articles met the inclusion criteria with most originating from the UK, investigating low back pain using grounded theory, phenomenology and thematic analysis. Facilitators and barriers were identified across patient and healthcare professional perspectives and synthesized into five themes: (1) personal and lifestyle characteristics, (2) nature and structure of the exercise programs, (3) real and perceived impact of the condition, (4) social and environmental resources, and (5) care provider style, skills, and supportive roles.

**Discussion/Conclusions**: Patient-related and healthcare system-related factors account for most of the enablers and barriers to adherence in this meta-ethnography. We propose a model that shows the interplay within and across the five factors influencing adherence as proposed by the WHO. Furthermore, The model also conceptualizes barriers and facilitators of adherence as being on a continuum that reflects the extent of positive or negative influence of the factors on adherence.

**Building solutions for kids in pain: Development of the first national health standard for pediatric pain management in Canada**

Kathryn Birnie^a^, Laura Gibson^b^, Paula Robeson^c^, Justina Marianayagam^d^, Natasha Murji^e^, Stephanie Paravan^f^, Samina Ali^g^, Randi Dovland Andersen^h^, Sandy Baggott^i^, Fiona Campbell^j^, G. Allen Finley^b^, Renee Manworren^k^, Tim Oberlander^d^, Kelly Thorstad-Cullen^l^, Susan Tupper^m^

^a^University of Calgary; Solutions for Kids in Pain; ^b^IWK Health; Solutions for Kids in Pain; ^c^Children’s Healthcare Canada; Solutions for Kids in Pain; ^d^BC Children’s Hospital; University of British Columbia; ^e^Patient, Health Standards Organization; ^f^Family Partner; ^g^University of Alberta; Solutions for Kids in Pain; ^h^University of Oslo; ^i^Alberta Children’s Hospital; ^j^SickKids; Solutions for Kids in Pain; ^k^Ann & Robert H. Lurie Children’s Hospital of Chicago; Northwestern University; ^l^Shriners Hospital for Children; ^m^Saskatchewan Health Authority; SaskPain

**Introduction/Aim**: The Government of Canada’s 2021 “Action Plan for Pain in Canada” identified a need for national standards to ensure equitable and consistent pain care across jurisdictions. Solutions for Kids in Pain (SKIP) partnered with the Health Standards Organization to develop the first national health standard for pediatric pain management (PedPM).

**Methods**: The PedPM standard followed an accredited 7-step development process: identify need; form expert committee; review literature; draft standard; public review; refine final standard; publish. The standard was guided by 15 experts, including people with lived experience, knowledge mobilization advisors, health professionals (psychology, medicine, nursing, child life, physiotherapy), and hospital administrators. Five experts in equity, diversity, and inclusion were additionally consulted.

**Results**: The PedPM standard applies to all settings providing inpatient, procedural, and/or outpatient services to children (birth to 18 years), including children’s, community/regional, and rehabilitation hospitals. The standard is for organizational leaders and teams in these settings with 35 criteria to: (1) Make Pain Matter: Establishing a Pediatric Pain Management Framework; (2) Make Pain Understood: Professional Development to Create a Knowledgeable and Confident Workforce; (3) Make Pain Visible: Comprehensive Pain Assessment and Reassessment; (4) Make Pain Better: Co-developing an Individualized Care Plan; (5) Make Pain Better: Multimodal Pain Management Strategies; and (6) Make Pain Matter: Continuous Quality Improvement for Pediatric Pain Management.

**Discussion/Conclusions**: The new PedPM standard guides equitable, quality, person-centered pain management for all children. It operationalizes the 2021 Lancet Child & Adolescent Health Commission on pediatric pain and the Action Plan for Pain in Canada.

**The power over pain portal: Implementation pilot in an Ontario tertiary pain clinic**

Etienne J. Bisson^a^, Amin Zahrai^a^, Yaadwinder Shergill^a^, Lynn Cooper^a^, Natalie Zur Nedden^a^, Sarah Fitzgerald^a^, Rachael Bosma^b^, Josh Rash^c^ and Patricia Poulin^a^

^a^The Ottawa Hospital Research Institute; ^b^University of Toronto; ^c^Memorial University

**Introduction/Aim**: The Power Over Pain Portal provides self-assessments and a range of resources including education, self-directed courses, peer support, interactive workshops, and access to one on one counselling through Wellness Together Canada. We integrated the Power Over Pain Portal into the referral pathway of a tertiary care pain clinic and evaluated acceptability among patients referred.

**Methods**: Administrative staff referred patients to the Power Over Pain team during their initial booking call. Patients attended a one-on-one portal orientation delivered by a member of the Power Over Pain team. Referrals and completed orientations were tracked, including number of patients who intend to use the Power Over Pain Portal in the following 4 weeks. We are conducting 4-week follow-ups to assess engagement with the portal, and perceived benefit/usefulness.

**Results**: Of the first 30 patient referrals (60% female; Mean age (SD)=54.3 (15.9) years), 24 (80.0%) completed the orientation. All intended to use the Power Over Pain Portal indicating high acceptability and agreed to a follow-up interview. Of the remaining 6, 2 (6.7%) were not interested and 4 (13.3%) requested their orientation be postponed. Those who completed the orientation were older than those who did not (Mean age (SD)=56.8 (15.3) years vs. 44.3 (15.6) years). We will present updated implementation results including 1-month follow-up data on portal engagement and perceived usefulness.

**Discussion/Conclusions**: Most patients referred to our tertiary care pain centre find the Power Over Pain Portal to be acceptable to meet some of their pain management needs.

**A systematic review on the effectiveness and safety of sucrose for heel lance procedures in neonates**

Janet Yamada^a^, Mariana Bueno^b^, Lucia Santos^c^, Sarah Haliburton^b^, Marsha Campbell-Yeo^d^ and Bonnie Stevens^e^

^a^Toronto Metropolitan University; ^b^The Hospital for Sick Children; ^c^University of Toronto; ^d^Dalhousie University & IWK Health Centre; ^e^The Hospital for Sick Children & University of Toronto

**Introduction/Aim**: Oral sucrose is the most frequently studied intervention for procedural pain in neonates. We aimed to determine the efficacy of sucrose for decreasing pain intensity of heel lance in neonates.

**Methods**: Systematic Cochrane Collaboration review standards were implemented. Electronic searches were conducted from database inception until February 2022. Randomised controlled trials (RCTs) with term and/or preterm neonates receiving sucrose for heel lancing were included. Comparison groups included no treatment, water, glucose, breast or formula milk, breastfeeding, local anaesthetic, non-nutritive sucking (NNS), and facilitated tucking. The primary outcome, pain intensity, was assessed by a validated pain measure; secondary outcomes included behavioral and neurophysiological parameters.

**Results**: 55 RCTs (6,273 neonates) were included. Heel lance occurred in 50 trials, the remaining investigated other minor painful procedures in addition to heel lance. Significantly lower pain intensity scores 30s after heel lance were observed for sucrose (20-30%, 0.1-2ml) compared to water, placebo, or no intervention: MD -1.74 (95% CI -2.11 to -1.37); I2=62%; 7 studies, 547 term and preterm infants. At 60s, there were significantly lower pain intensity scores with sucrose compared to water: MD -2.14 (95% CI -3.34 to -0.94); I2=0%; 2 studies, 164 term and preterm neonates. Significantly shorter cry duration was observed for sucrose (12-24%, 0.33-2ml) compared to water, placebo, or no intervention: MD -30.74 (95% CI -40.73 to -20.74); I2=96%; 4 studies, 222 infants. Minor adverse events required no intervention.

**Discussion/Conclusions**: Sucrose is effective for reducing procedural pain for single heel lances in preterm and term neonates.

**Do the benefits outweigh the harms? A systematic review of opioid deprescribing in older adults**

Feng Chang^a^, Wade Thompson^b^, Kevin Pottie^c^, Nyasha Gondora^d^, Daniel Stuckless^a^, Breanna Quan^a^, Jackie Stapleton^a^, Sarah Versteeg^a^, Qutaiba Al-Khames Aga^a^, Anchanah Jeyamohan^a^

^a^University of Waterloo; ^b^University of British Columbia; ^c^Western University; ^d^Canadian Institute of Health Research

**Introduction/Aim**: Patients, their caregivers, and prescribers may be willing to pursue stopping or reduction (deprescribing) of opioids, but evidence is limited to guide this decision among older adults. This study examined the benefits and harms associated with deprescribing opioids in older adults.

**Methods**: Using PRISMA’s systematic review (SR) framework, Medline, Embase and International Pharmaceutical Abstracts were searched (1995 onwards). Randomized controlled trials (RCTs), SRs, and guidelines that evaluated the reduction, tapering or discontinuation of opioids among participants ≥ 40yrs were included.

**Results**: Our search produced 2,249 results, and 20 records were eligible: 12 (60%) RCTs and 4 secondary analyses of RCT data, 2 were guidelines, and 2 were SRs. The most common interventions evaluated included behaviour change interventions aimed at reducing opioids (e.g., counselling, education, cognitive behavioural therapy, motivational interviewing, compassionate mind training etc., *n*=10), discontinuation of opioids (*n*=3) and tapering (*n*=2). The impact on opioid dose was frequently reported (10 RCTs and 2 SRs) with statistically significant improvements captured in 7 records. Opioid discontinuation was evaluated in 5 records (1 SR and 4 RCTs) with mixed results. Impact on opioid misuse was evaluated in 4 RCTs with 50% reporting a reduction in risk. No serious adverse events were reported among included interventions.

**Discussion/Conclusions**: In terms of healthcare outcomes, opioid deprescribing may be associated with more benefits than potential harms. Patient and provider-based education that utilizes behaviour change frameworks and ongoing support seem to be effective enablers to opioid dose reduction.

**The exclusion of people with comorbid depression from chronic pain clinical trials: A secondary data analysis**

Darren K. Cheng^a^, Maarij Hannan Ullah^b^, Henry Gage^c^, Rahim Moineddin^d^, Golale Modarresi^a^ and Abhimanyu Sud^d^

^a^Lunenfeld-Tanenbaum Research Institute, Sinai Health; ^b^University of Western Ontario; ^c^McMaster University; ^d^University of Toronto

**Introduction/Aim**: Comorbid chronic pain (CP) and depression is very common, but guidance for management is limited. It is unknown how often CP trials include people with depression - previous research has demonstrated that people with comorbid mental illness are often excluded from trials. The objectives of this study were to investigate the proportion of CP clinical trials with depression outcomes that included participants with depression at baseline, and examine variations in inclusion proportion by subgroups.

**Methods**: A secondary analysis was conducted of randomized controlled trials (RCTs) captured by systematic reviews in an umbrella review. RCTs with at least 50% adult participants and which used validated depression scales were eligible for analysis. RCTs with populations that met commonly cited minimum thresholds at baseline were considered to have included participants with depression.

**Results**: From 67 systematic reviews, 437 studies were identified, of which 346 RCTs were analysed. Overall, 142 (41%) RCTs included participants with depression. RCTs investigating fibromyalgia and mixed CP had the highest inclusion proportions (57% and 61%, respectively), whereas arthritis and axial pain studies had amongst the lowest at 19% and 27%, respectively. US RCTs had a significantly lower inclusion proportion (32%) compared to non-US studies (47%), especially for arthritis studies (10%).

**Discussion/Conclusions**: CP trials, particularly for common pain types such as axial and arthritis pain, often exclude participants with depression, thereby limiting their applicability to real-world populations. Future research must intentionally include individuals with comorbid depression in trials of common CP conditions to better inform clinical practice.

**Using the Adverse Childhood Experiences (ACE) score in deciding to prescribe opioids: Barriers to care and further marginalization**

Leigha Comer

Western University

**Introduction/Aim**: New guidelines around opioid prescribing for chronic pain have resulted in shifts in physicians’ opioid prescribing practices. So too have biopsychosocial approaches to chronic pain resulted in changes in diagnosis and management. Of note has been an increased use of screening instruments to account for psychosocial determinants of health. The Adverse Childhood Experiences (ACE) Score is one such screening instrument originally developed to predict risk of disease on a population level based on experiences of childhood trauma. This study investigates the use of the ACE Score by physicians when prescribing opioids for chronic pain.

**Methods**: This study is part of a research project that uses institutional ethnography to examine the organization of opioid use for chronic pain in Ontario. Semi-structured interviews were conducted with 20 people with chronic pain and 21 health care practitioners.

**Results**: Many physicians used the ACE Score to determine whether they could prescribe opioids to a patient with minimal risk that their licensing college, the provincial government, or its agencies would find fault with their prescribing practices. The ACE Score was perceived as objective, quantitative ‘proof’ that opioids could be safely prescribed. In contrast, physicians were highly reticent to prescribe opioids to people with medium or high ACE Scores.

**Discussion/Conclusions**: For people with chronic pain who are marginalized and who are more likely to have higher ACE Scores (e.g., women, Indigenous people) use of the ACE Score can bar them from accessing opioids for pain management. Use of screening instruments in this manner represents a significant risk of further marginalizing people with chronic pain.

**Development of a tool for navigating institutional barriers to patient partner compensation in pediatric pain research**

Brittany Cormier, Jennifer Parker, Isabel Jordan, Christine Chambers, Kimberly Strain, Yvonne Brandelli, Nicole MacKenzie and Emily Wildeboer

Dalhousie University

**Introduction/Aim**: Compensation for patient partners’ contributions to pediatric pain research is necessary within patient engagement (PE), with literature guiding best practices (Richards et al., 2018). Unfortunately, institutional policies often present barriers to implementing best practices (Richards et al., 2022). One barrier is a lack of knowledge around implementation, as institutional policies are not always tailored to the individuals involved. The aim was to develop an implementation tool to navigate institutional policies and address barriers to patient partner compensation.

**Methods**: The research team (staff, patient partners, and researchers/trainees) identified barriers to compensation. Staff from affiliated institutions were engaged in dialogue about institutional policies and best practices in PE. Co-development strategies and problem solving were used to address identified barriers and implement best practices.

**Results**: Staff, patient partners, and researchers/trainees were found to have unique informational needs: staff required a tool containing comprehensive information on institutional policies; patient partners required a tool focused on clarity around compensation details (when, where, and how much); and researchers/trainees required a communication tool to help bridge the gap between the two. Thus, three role-specific tools were co-developed and tailored to those who will use them. The tools can be used to guide other research teams navigating similar barriers. Preliminary data on the acceptance and appropriateness of use of the tools will be presented.

**Discussion/Conclusions**: Recommendations that patient partners be compensated and identification of institutional barriers are only the first step. Tools that address role-specific barriers to navigate the implementation of patient partner compensation within projects, teams, and institutions are needed.

**Loneliness and chronic pain: A scoping review**

Natasha Gallant, Briana De Roo, Amara Kohlert

University of Regina

**Introduction/Aim**: Chronic pain is often associated with adverse psychological outcomes such as anxiety, depression, and stress. Another psychological outcome that is underresearched among chronic pain populations is loneliness.

**Methods**: A scoping review using CINAHL (Ebsco), PsycINFO (Ovid), and Web of Science (Clarivate) was carried out to map out the body of literature examining the overlap between loneliness and chronic pain. The following inclusion criteria were used: (1) peer-reviewed journal articles with original quantitative research, (2) studies including at least one chronic pain sample and one non-chronic pain sample, and (3) studies including at least one measure of loneliness (e.g., UCLA Loneliness Scale).

**Results**: This search identified 94 studies that were screened and assessed for eligibility. After deduplication (*N*=22) and exclusion of articles following titles/abstracts screening (*N*=46) and full text reviewing (*N*=18), a total of 8 articles were included in this scoping review. Eligible studies included adolescents (*N*=3), adults (*N*=2), and older adults (*N*=3) with and without chronic pain. Adult chronic pain samples reported significantly more loneliness compared to adult non-chronic pain samples, but findings comparing adolescent and older adult chronic and non-chronic pain samples were mixed.

**Discussion/Conclusions**: Based on the findings of this scoping review, the association between loneliness and chronic pain may be influenced by age. The importance of bringing a lifespan perspective to the study of chronic pain and related psychosocial variables such as loneliness will be discussed.

**The prevalence of persistent post-traumatic headache in adult civilian traumatic brain injury: A systematic review and meta-analysis**

Alberto Herrero Babiloni^a^, Yasmine Bouferguene^b^, Fernando Exposto^c^, Marc O Martel^a^, Gilles Lavigne^d^, Estephan Moana-Filho^e^ and Caroline Arbour^d^

^a^McGill University; ^b^Hôpital du Sacré-Coeur de Montréal (CIUSSS du Nord de-l’Île-de-Montréal); ^c^Aarhus University; ^d^University of Montreal; ^e^University of Minnesota

**Introduction/Aim**: The most recent prevalence estimate of PTH after traumatic brain injury (TBI) in veterans and civilians dates back to 2008. The prevalence was found to be 57.8%, with surprising higher rates (75.3%) in mild TBI when compared to moderate/severe TBI (32.1%). However, the revision of mild TBI diagnostic criteria, and an historic peak of TBI in the elderly attributed to the ageing population, may lead to different results. Thus, we conducted a systematic review and meta-analysis to assess the updated prevalence of PTH during the last 14 years only in civilians.

**Methods**: A literature search was conducted following PRISMA guidelines and guided by a trained librarian. Screening, full text assessment, data extraction, and risk of bias assessment was done blindly by two raters. Meta-analysis of proportions using the Freeman & Tukey double arcsine methods of transformation was conducted. Heterogeneity, sensitivity analysis, and meta-regressions were performed with the predictors: year of publication, mean age, sex, TBI severity, and study design.

**Results**: 16 studies were selected for the qualitative analysis, and 10 for the meta-analysis. The overall prevalence estimate of PTH was 47.1%, (CI=34.6, 59.8, PI=10.8, 85.4), being similar at different time points (3, 6, 12, and 36+ months). Heterogeneity was high, and none of the meta-regressions were significant.

**Discussion/Conclusions**: The overall prevalence of PTH after TBI over the last 14 years remains high even if assessed only in civilians. However, the prevalence attributed to mild and moderate/severe TBI were similar, differing significantly from previous reports. Efforts are needed to improve outcomes after TBI.

**Acute cannabis consumption and motor vehicle collision: A systematic review and meta-analysis of observational studies**

Andrew Jin^a^, Andrea Darzi^a^, Amne Dargham^b^, Navroop Liddar^a^, Sepehr Bozorgi^a^, Shamim Sohrevardi^c^, Maurice Zhang^c^, Rachel Couban^a^, Behnam Sadeghirad^a^ and Jason Busse^a^

^a^McMaster University; ^b^LAU Gilbert and Rose-Marie Chagoury School of Medicine; ^c^Western University

**Introduction/Aim**: After legalization of non-medicinal cannabis in Canada in 2018, the prevalence of use in the past 12 months increased from 15% in 2017 to 25% in 2021. We conducted a systematic review to explore the association between cannabis use and motor vehicle collision (MVC).

**Methods**: We searched MEDLINE, EMBASE, CINAHL, Cocharne library, SCOPUS, PsycInfo, Web of Science, TRID, and the grey literature, from inception to December 2022, for observational studies investigating the association between MVC and acute cannabis consumption confirmed by detection of THC. We used random-effects meta-analysis to pool relative measures of association as odds ratios (ORs) and the GRADE approach to certainty of evidence.

**Results**: We included 29 studies with 307,176 individuals. We found low certainty evidence of an increased association between acute cannabis consumption and increased risk of fatal MVC (13 studies, OR 1.67, 1.41-1.97, *P*=0.0001, I2=50%), injury from MVC (11 studies, OR 2.24, 1.52-3.30, *P*=0.0001, I2=79%), and unsafe driving action (UDA)/culpability (15 studies, OR 1.40, 1.24-1.58, *P*=0.0001, I2=77%). We found no credible subgroup effects based on analysis method, location, date, and study type.

**Discussion/Conclusions**: Low certainty evidence suggests that acute cannabis consumption is associated with increased risk of MVC injuries, fatality, and UDA/culpability. Our findings highlight the importance of increasing public awareness regarding the dangers of cannabis-related impaired driving.

**Expertise from experience - Building capacity in trainees to implement patient partnerships in pain research**

Isabel Jordan^a^, Christine T. Chambers^a^ and Jennifer A. Parker^b^

^a^Dalhousie University; ^b^IWK Health

**Introduction/Aim**: Despite many methods of collaborating with patient partners (PP) in pain research, lack of knowledge regarding implementation of PE remains a barrier to its uptake by pain trainees. Recommendations on facilitating PE include improving the availability and accessibility of training resources, and ensuring that supervisors, departments, and institutions support and encourage PE. The aim of this posters is to describe our experiences embedding a PP in a staff role in a research lab to build capacity in for POR.

**Methods**: A part-time staff position, ‘Strategic Lead, Patient Partnerships’ was co-developed between the researcher and PP with responsibilities that included, training on PE best practices, development of team resources to support consistent and innovative PE methods, and mentorship to trainees through feedback at lab meetings and 1:1 coaching opportunities.

**Results**: The Strategic Lead, Patient Partnerships, in collaboration with the research team, developed a priority list of resources to support a standardized approach to patient partnership. Among other outcomes, PP compensation tools have been co-developed with lab members and PPs. Trainees are mentored in PE through 1:1 coaching opportunities as well as through feedback during regular lab meetings. This poster will provide a summary of key learnings from our first 2 years with this role.

**Discussion/Conclusions**: Patient partnership in POR has expanded in the past several years. The co-development of a paid role on a research team, staffed by a PP with PE expertise, can bridge the gap between the aspiration to partner with patients and the capacity to do so with knowledge and confidence.

**Race-based reporting and participation of Black individuals in registered pain clinical trials, United States 2000-2019**

Anh Khoa Vo^a^, Jessica Cerdeña^b^, Jonathan Loree^a^, Brian Cairns^a^, Annalijn Conklin^a^, Kimberley Kasewater^a^, Lerato Chondoma^a^, Jacquelyn Cragg^a^ and John Kramer^a^

^a^University of British Columbia; ^b^Yale University

**Introduction/Aim**: Despite guidelines, recommendations, and initiatives, the representation of Black, Indigenous, and other People of Colour (BIPOC) remains low in clinical trials across many different fields. There is a knowledge gap about racial diversity in pain clinical trials. The purpose of our study was to investigate race reporting and Black participation in registered pain interventional clinical trials in the United States.

**Methods**: We identified registered pain interventional studies from ClinicalTrials.gov. We included only studies that had reported final results (either in the ClinicalTrials.gov results database or had published results in a peer-reviewed journal), and extracted demographic data. Across all included studies, we computed the proportion that reported any race demographics and the proportion that comprehensively reported race demographics (i.e., multiple race categories reported). Of the studies that comprehensively reported race demographics, we computed the proportion of Black participants. We examined factors associated with race reporting and Black participation using logistic and linear regression.

**Results**: 1,200 trials met our inclusion criteria; 482 (40.2%) reported participant race. More recent, publicly funded, and larger trials were more likely to report race. Of 82,468 participants included in pain clinical trials that reported race, 15,101 were Black (18.3%). Participation of Black individuals was significantly associated with pain type (ß=+27% in cardiovascular compared to acute pain, p < 0.05), study population (ß=+33% and +7% in pain in minoritized populations and women, respectively, compared to the general population, *p* < 0.05), pain intervention (ß=+7.5% for trials of opioid interventions compared to non-opioid interventions, *p* < 0.05), and a diverse team of investigators (ß=+8.0% for studies incorporating a visible non-White investigator compared to those that did not, *p* < 0.05).

**Discussion/Conclusions**: Our results indicate that the representation of Black participants in pain clinical trials generally aligns with national demographics in the United States. Increased representation corresponds with health conditions more prevalent among Black individuals (e.g., cardiovascular disease), and with a diverse study team composition. Despite these encouraging results, less than half of pain trials reported race, which introduces potential publication bias and limits external validity.

**Spinal cord stimulation for neurogenic claudication in patients without prior spinal surgery: A systematic review**

Aaron Kirschner^a^, Akash Goel^a^ and Jasmine Kang^b^

^a^University of Toronto; ^b^Michael G. DeGroote School of Medicine

**Introduction/Aim**: Spinal cord stimulation (SCS) is often utilized for patients with persistent pain after spinal surgery, including those treated for neurogenic claudication (NC). However, patients may not be surgical candidates or may decline surgery. SCS is a potential alternative to spinal decompression in these and other patients.

We conducted a systematic review assessing the efficacy of spinal cord stimulation for neurogenic claudication in patients without prior spinal surgery.

**Methods**: The following databases were searched to a final date of November 14, 2022: Medline, PubMed, Embase, and the Cochrane Library. The authors screened abstracts and full-text papers for relevance according to pre-specified criteria. Data were extracted and compiled for comparison.

**Results**: 6 studies were identified and analyzed. 4 were retrospective series and 2 were prospective series. 1 study directly compared lumbar decompression to SCS, though the method of patient allocation was unclear. A total of 384 patients underwent an SCS trial or implantation.

Outcome measurements were heterogeneous across studies. All studies reported positive responses to SCS in outcome variables including pain at rest, pain while standing or walking, standing time, walking distance, walking restrictions, ADL completion, medication requirement, and Oswestry Disability Index. The proportion of responders varied. Both conventional and high-frequency stimulation were used. Adverse events were rare and included early and late lead dislocation and infection.

**Discussion/Conclusions**: SCS may present an effective alternative to lumbar decompression for patients with neurogenic claudication from lumbar spinal stenosis. It remains to be determined which patients would benefit most from SCS vs. spinal surgery.

Additional high-quality research is needed, including randomized controlled trials, to remove potential bias and more accurately assess SCS as a potential treatment modality.

**Rehabilitation interventions for complex regional pain syndrome; An overview of systematic reviews**

Joy Macdermid^a^, Erfan Shafiee^a^, Tara Packham^b^, David Walton^a^, Ruby Grewal^a^, Maryam Farzad^a^ and Pavlos Bobos^a^

^a^Western University; ^b^McMaster University

**Introduction/Aim**: Complex Regional Pain Syndrome (CRPS) is a painful and disabling condition. ‎ A Systematic reviews have evaluated conservative ‎management of CRPS.To summarize and critically appraise the sysmatic reviews on conservative management of the CRPS.

**Methods**: We conducted a literature search from inception to January 2023 in ‎the following databases: Embase, Medline, CINAHL, Google Scholar, Cochrane ‎library, and Physiotherapy Evidence Database (PEDro). Two reviewers conducted ‎study screening, data extraction, and methodological quality assessment (using ‎AMSTAR-2). Qualitative synthesis was performed; the corrected covered area (CCA) index described overlap in primary studies.

**Results**: We identified 214 articles and a total of 9 systematic reviews of RCTs eligible for inclusion. Pain and disability were the most ‎common outcomes. There were six (6/9;66%) high quality, two (2/9;22%) moderate-quality, and one ‎critically low-quality systematic reviews (1/9;11%); the trials ranged from very low to high quality. There was a large overlap across primary studies (CCA=23%). There was a large effect size for the effectiveness of mirror therapy on pain and disability (SMD:1.88 (95%CI: 0.73 to 3.02) and 1.30 (95%CI: 0.11 to 2.49), respectively) and the effectiveness of graded motor imagery on pain and disability (SMD: 1.36 (95%CI: 0.75 to 1.96) and 1.64 (95%CI: 0.53 to 2.74), respectively).

**Discussion/Conclusions**: Systematic reviews support the use of movement representation techniques such as mirror therapy and graded motor imagery programs in patients with CRPS. The evidence is not comprehensive nor of sufficient quality to make definitive recommendations.

**Factors perceived as supporting successful knowledge mobilization in pediatric pain: A qualitative matrix analysis of implementation partner perspectives**

Nicole E. MacKenzie^a^, Christine T. Chambers^b^, Christine E. Cassidy^c^, Penny V. Corkum^c^, Meghan E. McGrady^d^, Jennifer A. Parker^e^ and Kathryn A. Birnie^f^

^a^Dalhousie University, IWK Health; ^b^Dalhousie University, IWK Health, Solutions for Kids in Pain; ^c^Dalhousie University; ^d^University of Cincinnati; ^e^IWK Health; ^f^University of Calgary, Dalhousie University, Solutions for Kids in Pain

**Introduction/Aim**: Knowledge mobilization (KM) activities (e.g., patient resources, clinical practice guidelines) contribute to high-quality pain management by supporting the implementation of evidence into practice. The engagement of researchers, health professionals, and patients/families in KM activities is growing; however, understanding of how each group engages in these activities is limited. This qualitative study identified factors that each partner group perceived as supportive of successful KM in pediatric pain.

**Methods**: Participants included ten individuals from three healthcare partner groups (researchers, health professionals, patients/families) who had experience leading, or contributing to, at least one pediatric pain KM activity participated in semi-structured interviews. The interview guide was informed by the Consolidated Framework for Implementation Research. Thematic analysis was used to generate themes within each group, and a matrix analysis was used to generate overarching themes and identify areas of convergence/divergence among the three groups.

**Results**: Analyses generated four overarching themes: 1) team dynamics (i.e., relationships and interaction quality within teams); 2) role of leadership (i.e., responsibilities of project leaders); 3) policy influence (i.e., priorities, resources); and 4) social influence (i.e., role of colleagues and practice communities on support for KM). While all groups agreed on what constituted supportive team dynamics, there were different perspectives on how policy, social, and leadership factors supported KM initiatives.

**Discussion/Conclusions**: Although partners identified common areas of support for KM activities, they shared unique perspectives on how each group can optimally be supported when it comes to external factors. These nuances should be considered when engaging different healthcare partners in KM initiatives for pain.

**The association of funding source and reporting quality with treatment effects among randomized trials on the management of pain associated with temporomandibular disorders: A meta-epidemiological study**

Sara Moradi^a^, Qi Wang^a^, Khotan Aflatounia^b^, Holly Crandon^c^, Liang Yao^a^, Ivan D. Flórez^d^, Jeffery Wells^e^, Jason W. Busse^a^ and Behnam Sadeghirad^a^

^a^Department of Health Research Methods, Evidence and Impact, McMaster University, Hamilton, Ontario, Canada; ^b^Department of Restorative Dentistry, Dental School, Shahed University of Medical Sciences, Tehran, Iran; ^c^The Michael G. DeGroote Institute for Pain Research and Care, McMaster University, Hamilton, Ontario, Canada; ^d^Department of Pediatrics, University of Antioquia, Medellín, Colombia; ^e^Cleveland Clinic, Head and Neck Institute Cleveland, OH, USA

**Introduction/Aim**: To assess the reporting quality of randomized trials evaluating treatment of chronic pain secondary to temporomandibular disorders (TMDs) using a modified version of the Cochrane risk of bias instrument, and to examine the association between sample size calculation, funding source, and other reporting quality with the likelihood of finding a statistically significant effect on pain relief.

**Methods**: We conducted a systematic search to identify randomized clinical trials (RCTs) evaluating the treatment of chronic pain secondary to TMDs up to May 2021. We extracted the following from all eligible trials: (1) industry vs. non-industry funding, (2) reporting a sample size calculation, and (3) risk of bias. We built a logistic regression model to evaluate the association between these three factors and finding a statistically significant effect.

**Results**: A total of 210 trials were eligible for review. %60.95 of included studies failed to adequately provide sample size calculations. Non-for-profit organizations provided financial support to %40.95 of included studies. Industry accounted for less than %3 of the funding. Approximately %40 of studies did not provide any statement on funding. In terms of risk of bias, %85 of the included studies were assessed to have a high risk of bias overall. The multivariate regression model showed that reporting the details of sample size calculation was significantly related to reporting a significant pain reduction outcome (OR:2.86, %95 CI (1.22- 6.7)) among trials. Funding source and risk of bias did not show a significant association with reporting significant pain reduction.

**Discussion/Conclusions**: Clinical trials on the treatment of TMJ disorders failed to adequately report the sample size calculations and funding source. The issue of not reporting accurate sample size calculation and running under power trials remains widespread in the TMD trials, which affects the treatment estimations.

**Mapping the scope of pain assessment in studies of youth with brain-based developmental disabilities: Identifying inequities for more holistic assessment**

Samantha Noyek^a^, Jenna Jessa^a^, Violeta Faulkner^a^, Cara Gail Nania^a^, Katelynn E. Boerner^b^, Tammie Dewan^a^, Dacey Doyle, Lara Genik^c^, Stacy Grainger-Schatz, Carly A. McMorris^a^, C. Meghan McMurtry^c^, Tim Oberlander^b^, Diane L. Lorenzetti^a^, Kailyn Turner^a^ and Kathryn A. Birnie^a^

^a^University of Calgary; ^b^University of British Columbia; ^c^University of Guelph

**Introduction/Aim**: Pain experiences of youth with brain-based developmental disabilities are often overlooked and misunderstood. A recent systematic review by our team revealed that a troubling number of pain assessments in youth with brain-based developmental disabilities focus exclusively on pain intensity (47.2%). While holistic pain assessment is the standard approach for neurotypical youth, it is largely absent in pain assessments of youth with brain-based developmental disabilities. In this project, we described a sub-set of articles with a primary focus on pain to identify outcomes beyond pain intensity.

**Methods**: A comprehensive search was conducted (August 2021) using six academic databases. Eligible studies for this analysis were English quantitative articles with a primary focus on assessing pain in youth 3-24 years-old with any brain-based developmental disability, where pain intensity measures met Cohen’s criteria (measure used at least twice by two different research groups). Data regarding additional outcomes and participant characteristics were extracted.

**Results**: 135 articles were eligible for inclusion. Outcomes assessed alongside pain intensity included: quality of life (*n*=21; 15.6%), pain interference (*n*=12; 8.9%), mental health (*n*=9; 6.7%), sleep (*n*=3; 2.2%), and social support (*n*=1; 0.1%). No articles assessed pain location, participation, family functioning, or sleep within the context of acute pain assessment. Articles rarely reported ethnicity (*n*=12, 8.8%) and frequently omitted information about youth’s motor ability (*n*=48; 35.6%) and gender (*n*=49; 35.9%).

**Discussion/Conclusions**: Limited scope of outcome assessment indicates that youth with brain-based developmental disabilities face inequities in their pain assessment as compared with neurotypical youth. Broadening key outcomes can improve holistic pain assessment in these youth and transparent reporting of individual participant characteristics is critical for future research.

**Neuroscientific implications for the development of virtual reality for patients with chronic pain**

Zahra Ofoghi and Diane Gromala

Simon Fraser University

**Introduction/Aim**: Virtual reality (VR) is a health-tech innovation that activates sensory and perceptual mechanisms in a controlled environment and can be used as a novel intervention to reduce pain in complex chronic conditions such as chronic pain (CP). The VR experience is a multi-sensory intervention which engages multiple aspects of the human sensory system, such as visual, auditory, tactile, vestibular, and proprioception. Therefore, it is critical to consider unique alterations of the sensory system in patients with chronic pain when one is developing and testing VR. Here, we summarize neuroscientific findings regarding the sensory sensitivities of patients with CP and provide practical considerations for the design and development of VR.

**Methods**: We performed a selective review of the literature regarding sensory system alterations in chronic pain and classified sensory sensitivities. Next, we will discuss design considerations for VR and present examples from our VR projects.

**Results**: The main sensitivities of the sensory system in patients with CP are mainly reported in visual, auditory, and tactile domains. Design recommendations for each sensory sensitivity will be discussed. In developing VR, we found that including a patient partner in each design step plays a critical role in confirming VR is adaptable to the unique characteristics of patients with CP.

**Discussion/Conclusions**: Sensitivity in the sensory processing of patients with chronic pain should be considered in the design and development of any new health-tech intervention. This could potentially optimize the effect of VR.

**Yoga for Veterans with Chronic Pain: a scoping review**

Neil Pearson

University of British Columbia

**Introduction/Aim**: This scoping review examines the state of research conducted on yoga for veterans with chronic pain. Chronic pain is a complex whole-human condition, with implications across the biopsychosocial spectrum. Yoga, with its inherent biopsychosocial approach, may have potential as a component of interdisciplinary pain care.

**Methods**: A literature review of CINAHL, Pubmed and Medline was completed from 2007 to 2015.

A literature search was completed, with specific objectives:

- Identifying available evidence

- Clarifying key concepts and definitions

- Evaluating how research is completed on yoga for civilians and veterans with chronic pain

- Identifying knowledge gaps

- Providing guidance for future research

**Results**: Thirteen quantitative reports and 3 qualitative reports were found.

Key findings include: positive effects of yoga are reported across biopsychosocial outcomes with few adverse effects, reductionistic perspectives are included in the reports related to people in pain, pain care and yoga, yoga interventions were not informed by veterans or by pain science, methodological transparency was lacking for the yoga interventions, and barriers exist to veterans benefiting from yoga as pain care.

**Discussion/Conclusions**: This review describes quantitative and qualitative studies about yoga and pain care as they relate to the unique experiences of veterans with chronic pain. The review identifies and analyzes gaps in the research, affirms the need for further study, and concludes with guidance to assist researchers in developing the most productive methodologies for ongoing study in this field. Future directions include: veteran-informed yoga interventions, methodological transparency, pain-informed yoga interventions, enhanced education of yoga teachers, maintaining yoga’s inherently integrated BPS approach, and studying individualized yoga therapy as pain care.

**The Alberta Back Care pathway: Implementation of a novel care pathway to improve low back pain management in primary care settings**

Brandyn Powelske^a^, Allyson Jones^a^, Alice Kongsted^b^ and Greg Kawchuk^a^

^a^University of Alberta; ^b^University of Southern Denmark

**Introduction/Aim**: In Canada, individuals experiencing low back pain (LBP) often seek care from family doctors because it is a no-cost healthcare option for patients. Unfortunately, primary care physicians often do not have access to evidence-based interventions resulting in inappropriate use of opioids, imaging and specialist referrals. To overcome this problem, the Alberta Back Care pathway (ABCp) was co-developed by patients, physicians and others to provide funded, best-practice interventions for presentations of acute, sub-acute, chronic, chronic non-responsive and radiculopathy LBP. The objective of this study was to evaluate ABCp implementation in Primary Care Networks (PCN).

**Methods**: In this hybrid effectiveness-implementation study, ABCp was implemented in a sub-population of currently 7 PCN clinics in Alberta commencing April 2021. Here, we compared results in the maintenance domain of the RE-AIM implementation framework for the two PCNs using the ABCp for the longest duration. Maintenance was used to answer the question, “Was the program sustained by the physician population twelve months post-implementation?”

**Results**: In the first targeted PCN, 40 out of 162 eligible physicians (25%) participated in ABCp. Of the 9 physicians that were enrolled for at least one year and referred patients, 6 (67%) continued to refer patients one year after implementation commenced. Comparatively, 11% (24/221) of eligible physicians participated in the second PCN. Among the 11 physicians who referred patients and were enrolled for at least one year, 91% (10/11) demonstrated sustained use of ABCp.

**Discussion/Conclusions**: One year after implementation, ABCp has been maintained by at least 65% of physician users at two large PCNs.

**The use of psychedelics for pain: A systematic review**

Yeshith Rai^a^, Shayan Sivadas^b^, Calvin Diep^a^, Karim Ladha^c^ and Akash Goel^c^

^a^University of Toronto; ^b^Queen’s University; ^c^St. Michael’s Hospital

**Introduction/Aim**: The mechanisms underlying pain are poorly understood. Conventional therapeutic agents for chronic pain such as opioids have been associated with adverse side-effects, issues with addiction and ineffective analgesia. Novel agents with a low side-effect burden and risk profile are needed to fill the therapeutic gap in chronic pain management. Psychedelics are thought to alter pain perception and processing through direct serotonin receptor (5HT2A) agonism, anti-inflammatory effects and synaptic remodeling.

**Methods**: This systematic review was conducted to identify human studies in which psychedelic agents were used for the treatment of pain. Two reviewers independently assessed titles, abstracts and full-text English articles using the Ovid Medline, Cochrane Database of Systematic Reviews, APA Psychinfo and EMBASE databases.

**Results**: Twenty-one studies that assessed the effects of psychedelics in treating various pain states were included. Lysergic acid diethylamide (LSD) and psilocybin were the most analyzed psychedelics for a variety of pain states. There was great variability in dosing and routes of administration. The most frequent diagnoses in this review were migraines and headaches. The remaining studies explored fibromyalgia, phantom-limb pain, malignant pain among other mixed pain states. The constellation of evidence is encouraging for psychedelic use in the management of pain, notably headaches, phantom-limb pain, and cancer pain.

**Discussion/Conclusions**: While mechanisms of their efficacy remain unclear, direct 5HT2A agonism and anti-inflammatory effects may play an important role. Future clinical trials should assess the effectiveness of various psychedelic agents in chronic pain management, especially considering the limited efficacy and complex side effects associated with existing treatment options.

**Identifying core outcome domains to guide adult albertans living with chronic pain**

Magali Robert^a^, Jatin Patel^b^, Elena Lopatina^a^ and Sunita Vohra^c^

^a^University of Calgary; ^b^AHS; ^c^University of Alberta

**Introduction/Aim**: The Alberta Pain Strategy tasked the Outcome Measures Working Group in defining the minimum core outcome measures that “will be shared openly and used uniformly across the province to guide healthcare.”

**Methods**: A modified Delphi process was undertaken to identify the most important measurement domains in adults.

Using the IMMPACT1 domains as a starting point, an online survey was done targeting Alberta stakeholders. The second survey to initial respondents included additional domains identified in round 1. The working group then convened online to review the results. Open discussion ensued until consensus was achieved.

**Results**: The first round sent February 21, 2021, 250 respondents [134 professionals, 109 people with lived experiences (PWLE), 7 blank] voted all domains to remain and the addition of: treatment options, healthcare optimization, barriers to care, cognition domains. Round two, sent Nov 15, 2021, resulted in no domains being removed (88 respondents; 47 professionals, 40 PWLE). Consensus of the working group members (26 members) meeting January 19, 2022, identified functional outcomes, emotional outcomes and impression of change as core outcomes.

**Discussion/Conclusions**: The core outcome domains selected from this study will be used to identify validated outcome measurement instruments for adults with chronic pain living in Alberta.

**Establishing consensus on key domains to evaluate pediatric transitional pain services**

Brittany N. Rosenbloom^a^, Isabel Jordan^b^, Dawn P. Richards^c^, Fiona Campbell^d^, Lisa Isaac^d^, Jennifer Tyrrell^e^, Jennifer Stinson^f^ and Kathryn A. Birnie^g^

^a^The Hospital for Sick Children; ^b^N/A - Patient Partner; ^c^Five02 Labs Inc; ^d^The Hospital for Sick Children and University of Toronto; ^e^The Hospital for Sick Children, University of Toronto; ^f^The Hospital for Sick Children, University of Toronto; ^g^University of Calgary

**Introduction/Aim**: “Transitional Pain Services” (TPS) have emerged in adult tertiary care as an innovative and effective health service model to identify and address risk factors for chronic post-surgical pain (CPSP), disability, mental health, and opioid use. With some pediatric TPS developing, it was our aim to identify developmentally appropriate outcomes to determine pain service effectiveness.

**Methods**: Multi-stakeholder participants completed a modified Delphi survey after two virtual design thinking workshops to co-design pediatric TPS. Survey 1 asked participants to identify important outcomes for evaluating the implementation, effectiveness, and success of pediatric TPS. All suggested outcomes from Survey 1 were included in Survey 2. Survey 2 asked participants to rate each outcome from 0 (“not at all important”) to 10 (“extremely important”). Consensus on an item was defined a priori and was achieved if at least 80% of participants agreed the outcome was “core” (i.e., a score of ≥8).

**Results**: Participants were youth with CPSP, parents, multidisciplinary health professionals in pediatric and/or surgical services, and decisionmakers (n=24/27 survey 1; n=18/21 survey 2). After two surveys, 11 domains were considered very important including both patient (emotional functioning/mental health, physical recovery, role functioning, sleep, reduced number of youth developing CPSP) and health service (adoption of evidence-based practice, appropriateness/perceived fit, feasibility, sustainability, accessibility, continuity of care) outcomes.

**Discussion/Conclusions**: These results directly inform the evaluation of pediatric TPS outcomes identified as important by youth, parents, health professionals, and decisionmakers, and the optimal design of pediatric TPS.

**Comparative benefits and harms of pharmacological interventions for fibromyalgia: a systematic review and network meta-analysis of randomized trials**

Behnam Sadeghirad^a^, Dena Zeraatkar^a^, Elena Kum^a^, Yvgeniy Oparin^a^, Malgorzata M. Bala^b^, Luis E. Colunga-Lozano^a^, Mi A. Han^c^, Montserrat Rabassa^d^, Robin Vernooij^e^, Li Wang^a^, Elisabetta Trinari^a^, Xiaoqin Wang^a^, Rachel Couban^a^, Matthew Cooper^a^, Shanil Ebrahim^a^, Gaelan Connell^f^, Eric A. Coomes^g^, Paul Bruno^g^, Keshena Malik^f^, David Torrance^f^, Trung Ngo^f^, Karin I. Kirmayr^i^, Daniel Avrahami^f^, John J. Riva^a^, Peter A Struijs^j^, David Brunarski^f^, Stephen J. Burnie^f^, Stefan Schandelmaier^k^, Milosz Jankowski^l^, Wiktoria Lesniak^l^, Gordon Guyatt^a^, Victor Montori^m^, Jason W Busse^a^

^a^McMaster University; ^b^Jagiellonian University; ^c^Chosun University; ^d^Biomedical Research Institute San Pau; ^e^University Medical Center Utrecht; ^f^Canadian Memorial Chiropractic College; ^g^University of Toronto; ^h^University of Regina; ^i^Sanatorio San Carlos Bariloche; ^j^Academic Medical Center; ^k^University Hospital Basel; ^l^Jagiellonian University Medical College; ^m^Mayo Clinic

**Introduction/Aim**: Fibromyalgia is a common chronic pain syndrome and it is associated with reduced quality of life. Several treatments are available for management of fibromyalgia, but their relative benefits and harms are unknown. We performed a systematic review and network meta-analysis to examine the comparative effectiveness of available pharmacotherapies for management of fibromyalgia.

**Methods**: We searched MEDLINE, EMBASE, Web of Science, CINAHL, and Cochrane CENTRAL up to February 2021 for trials that: (1) enrolled outpatient adults diagnosed with fibromyalgia, (2) randomized at least 10 patients to any pharmacotherapy vs. an alternate pharmacotherapy or placebo, and (3) measured a patient-important outcome (pain, physical and mental function, sleep quality, quality of life, drop-out due adverse effects, and all-cause drop-out) with at least 4-weeks follow-up. We performed a random-effects frequentist network meta-analysis and assessed the certainty of evidence using GRADE methodology.

**Results**: We included 113 randomized trials assessing more than 40 pharmacological interventions, several of which showed statistically significant benefit across outcomes. Of the 16 interventions that showed significant pain reduction, tricyclic antidepressants (TCA) and their combination with SSRI or vitamin D, selective serotonin reuptake inhibitors (SSRI) and their combination with melatonin or muscle relaxant, serotonin–norepinephrine reuptake inhibitors (SNRI) and their combination with pregabalin, and melatonin were associated with the largest, most precise effects. Only five treatments showed statistically significant benefit for physical function (SNRI, pregabalin, combination of SNRI and pregabalin, sodium oxybate, and combination of paracetamol with an opioid), but the associated precision for each included both important and unimportant effects. SNRI, pregabalin, combination of SNRI and pregabalin, and sodium oxybate were associated with significant drop-out due to adverse effects.

**Discussion/Conclusions**: Our findings suggest that several antidepressants, alone or in combination with other co-interventions, may provide important pain relief for fibromyalgia, but these interventions were also associated with drop-out and drop-out due to adverse effects suggesting they are not well-tolerated by all patients.

**Peripheral nerve blocks for treatment of migraine headaches: Systematic review and network meta-analysis of randomized trials**

Behnam Sadeghirad^a^, Emily Thorburn-Winsor^a^, Simranjit Kaur^a^, Malahat Khalili^a^, Mojtaba Dayyani^b^, Rami Z. Morsi^c^, Preksha Rathod^a^, Cieran Tran^a^, Feryal Momenilandi^d^, Rachel Couban^a^ and Jason W. Busse^a^

^a^McMaster University; ^b^City of Hope Hospital; ^c^University of Chicago; ^d^Shahid Beheshti University of Medical Sciences

**Introduction/Aim**: Current migraine treatment options are to an extent effective, but the time to pain relief is suboptimal. Peripheral nerve blocks (PNBs) have been suggested to have promising benefits, while their comparative effects are unknown. We performed a systematic review and network meta-analysis to examine the effectiveness of different PNBs for management of migraine headaches.

**Methods**: We searched MEDLINE, EMBASE, Web of Science, CINAHL, Scopus, and Cochrane CENTRAL up to September 2022 for trials that: (1) enrolled outpatient adults with primary chronic headache, tension-headache, migraine, or cluster headache, and (2) randomized them to any PNB methods directed at management of headache disorder or sham injection. We performed a random-effects frequentist network meta-analysis for all patient-important outcomes (pain, number of days with headache, treatment response/headache freedom, and number of headache episodes).

**Results**: We included 19 randomized trials comparing greater occipital nerve block (GONB), suboccipital nerve block, supraorbital nerve block, sphenopalatine ganglion blockade, their combination or PNBs together with corticosteroids or botulinum toxin. Compared to sham injection, GONB (mean difference [MD] on a 10cm VAS: -1.3, 95% CI: -0.6 to -2.0, moderate quality) or GONB+botulinum toxin (MD on a 10cm VAS: -3.9, 95% CI: -2.0 to -5.8, moderate quality) or GONB+corticosteroid (MD on a 10cm VAS: -1.5, 95% CI: -0.2 to -2.7, low quality) were among the most effective treatments for pain management. GONB and GONB+corticosteroid reduced the average number of headache days/months (MD=-8.3 days, 95% CI: -3.3 to -12.8 days and MD=-10.7, 95% CI: -4.0 to -17.3, respectively; both low quality). No treatment showed benefit for headache freedom.

**Discussion/Conclusions**: Our findings suggest that GONB alone or in combination with corticosteroids or botulinum toxin may be among the most effective PNBs to manage pain and headache associated with migraine. Networks for headache freedom and average headaches were sparse and mostly based on low quality evidence.

**Identification of pre-surgical risk factors for the development of chronic post-surgical pain in adults: A comprehensive umbrella review**

Beate Sydora^a^, Lindsay Whelan^b^, Sumera Idris^a^, Gurpreet Brar^a^, Benjamin Abelseth^a^, Rachel Zhao^a^, Ashley Jane Leonard^a^, Sanjay Beesoon^a^, Hance Clarke^c^, Joel Katz^d^ and Nivez Rasic^e^

^a^Alberta Health Services; ^b^Alberta Health Services and University of Calgary; ^c^Toronto General Hospital; ^d^York University; ^e^University of Calgary

**Introduction/Aim**: A variety of risk factors for chronic post-surgical pain (CPSP) have been reported in primary studies and an increasing numbers of reviews. The objective of our study is to conduct an umbrella review to collate the full range of published pre-surgical risk factors for the development of CPSP for various surgery types.

**Methods**: Six databases were searched from January 2000 to August 2022 to identify Meta-analysis, scoping and systematic reviews investigating pre-surgery CPSP predictors in adult patients. Articles were screened by title/abstract and subsequently by full text by two independent reviewers. The final selected papers were appraised for scientific quality and validity. Data was extracted and analyzed descriptively.

**Results**: From a total of 2049 retrieved articles, 235 were screened in full and 29 were selected for in-depth scrutiny. The number of primary studies, mainly observational (76%), ranged from 4 to 317. Surgery types were arthroplasty (*n*=9), spine (*n*=6), breast (*n*=4), shoulder (*n*=2), carpel tunnel syndrome (*n*=1), and various orthopedic surgeries (*n*=1). Six reviews included combinations of assorted surgeries. A total of 38 pre-surgical risk factors could be identified; further grouping reduced this number. The risk factors were themed into six categories: psychological, health-related, pain-related, demographic, genetic, and social/lifestyle-related. Strength of evidence was varied and conflicting for some predictors. Consistently high evidence was found for risk factors in the psychological grouping.

**Discussion/Conclusions**: Identification of pre-surgical risk factors is crucial for the development of screening tools to predict CPSP. Further research is needed to ascertain modifiable pre-surgical risk factors to mitigate the development of CPSP.

**Improving access to specialist care through electronic asynchronous consultation for patients living with chronic pain: A feasibility study in quebec**

Regina Visca^a^, Yoram Shir^a^, Christine Florakas^b^, Samuel Rodriguez-Qizilbash^c^, Robert Iny^d^, Jamie Rozen^d^, Mark Karanofsky^d^, Irina Kudrina^d^, Benoit Lapierre^e^ and Clare Liddy^f^

^a^McGill University, McGill University Health Centre; ^b^McGill University, CIUSSS Centre-Ouest-de-l’ïle de Montréal; ^c^McGill University, Jewish General Hospital; ^d^McGill University; ^e^CIUSSS Centre-Ouest-de-l’Ile de Montréal; ^f^University of Ottawa

**Introduction/Aim**: Scarcity of resources may lead to unacceptable delays for patients who require evaluation and treatment by a chronic pain specialist. Electronic consultation (EC) has emerged as one possible solution, allowing primary care providers (PCP) appropriate and timely access to specialists’ advice. This study aimed to 1) implement EC in a small community of early adopters; 2) identify barriers and facilitators affecting implementation; 3) assess EC’s impact on access to care; and 4) identify workflow considerations and features of high-quality EC.

**Methods**: We conducted a qualitative participatory action study to determine how best to use EC for chronic pain in Quebec. Data collection methods included a post-EC survey and focus group discussions with 7 PCPs and 1 specialist. Patterns of use were summarized using descriptive analysis and focus groups were analysed through the lens of the Quadruple Aim using content analysis.

**Results**: Results focused on the impact of EC on PCP course of action, referral behavior, educational value and satisfaction. Perceived advantages included faster access to specialist advice and definitive care, improved communication and care coordination, stronger partnerships, enhanced decision-making, increased confidence, and enriched learning. Key barriers to using EC included timing, technology, and work-flow integration, while facilitators focused on strategic alignment, technology, and quality of the exchange.

**Discussion/Conclusions**: Our study demonstrates that it is feasible to implement EC for chronic pain. The PCPs and specialist had a positive experience with EC, including easier access to specialist advice and improved clinical course of action, and recommended workflow improvements, notably integrating EC with electronic referral.

**Understanding the experiences of parents engaged as partners in co-created knowledge mobilization initiatives in children’s pain: A qualitative study**

Emily Wildeboer^a^, Christine Chambers^a^, Perri Tutelman^b^, Justine Dol^c^, Isabel Jordan^c^, Jennifer Parker^c^, Jennifer Stinson^d^ and Erica Ehm^e^

^a^Dalhousie University; ^b^University of Calgary; ^c^IWK Health; ^d^University of Toronto, Hospital for Sick Children; ^e^Yummy Mummy Club

**Introduction/Aim**: Engagement of patients as members of research teams has been increasingly recognized as an important part of patient-centered health research. A better understanding of patient partners’ experiences is needed, however, to guide best practices in engaging patients in knowledge mobilization (KM) activities. The aim of this qualitative study is to understand the experiences of patient partners who were engaged in two co-created KM initiatives in children’s pain research.

**Methods**: Data were collected as part of two collaborative KM initiatives about children’s pain: It Doesn’t Have to Hurt and Kids Cancer Pain. Both engaged patient partners to co-create digital content to disseminate evidence on children’s pain management. Following their participation, partners took part in a structured interview about their engagement experiences. Interviews from each initiative were recorded, transcribed, and analyzed separately using an inductive content analysis approach.

**Results**: A total of 18 patient partners (9 from each initiative) participated. Overall, partners described their engagement experiences very favourably. Four categories were generated across both sets of data: 1) positive outcomes for partners, including empowerment within the project and applicable knowledge outside the project; 2) differences between researcher and partner perspectives based on their unique experiences; 3) necessity of multiple patient partners on research team; and 4) key areas of strength, including researcher authenticity and passion.

**Discussion/Conclusions**: These results can inform the development of best practices in patient engagement. Specifically, they can inform recommendations on how to improve engagement within pediatric pain research and KM.

**Knowledge translation initiatives for older adults and other knowledge users: A systematic review**

Laney Yarycky^a^, Louise I. R. Castillo^a^, Michelle M. Gagnon^b^ and Thomas Hadjistavropoulos^a^

^a^University of Regina; ^b^University of Saskatchewan

**Introduction/Aim**: Pain is often undertreated in older adult populations. Initiatives to increase knowledge about pain assessment and subsequent management are crucial to improving quality of life. Knowledge translation (KT) studies focused on delivering pain management education to older adults and relevant knowledge users are available in the literature. However, KT program formats and outcomes vary greatly, presenting a need to assess and comprehensively report on the current literature on geriatric pain management. We aimed to systematically review the literature on KT programs targeted towards older adults, healthcare professionals, and informal caregivers.

**Methods**: This registered systematic review examines general characteristics of KT initiatives, alignment of program goals to established implementation outcomes, and provides recommendations for future research. CINAHL, MEDLINE, PsycInfo, and Web of Science were searched from inception to July 2022. Search results have been organized in Covidence (2022). A random 20% of articles have been examined by a second reviewer to establish consensus.

**Results**: Consensus from title and abstract review was 88%. Consensus from full text review was 90%. From an initial 18,162 search results, 174 studies were retained for inclusion. Studies retained for inclusion were examined for study strength and risk of bias through a narrative synthesis.

**Discussion/Conclusions**: Overall the number of geriatric KT pain management programs is limited and the literature is characterised by an inconsistent selection of measured implementation outcomes. Research gaps will be reported in order to better inform future KT research practices and program development.

**Imaging: Pain Imaging and Neuroimaging L’imagerie: l’imagerie de la douleur et la neuroimagerieGreater physical activity moderates brain connectivity to decrease pain interference**

Yi An Wang^a^, Allison McPeak^a^, Cara Nania^a^, Melanie Noel^a^, Lucy Stuckey^a^, Inge Timmers^b^, Laura Simons^c^ and Jillian Vinall Miller^a^

^a^University of Calgary; ^b^University of Tilberg; ^c^Stanford University

**Introduction/Aim**: An estimated one-in-five Canadian youth have pediatric chronic pain (pain > 3 months). Chronic headache is the most common pain experienced by youth. Chronic headache impairs daily functioning, including school, extracurricular activities, and social functioning, and is strongly associated with comorbid mood disorders. Evidence suggests that chronic headache significantly alters brain microstructure and connectivity. Physical activity is a protective factor against pain interference. However, little is known about how physical activity affects brain connectivity to decrease pain interference in youth with chronic headache.

**Methods**: Thirty youth (age=14.4, SD=2.6, 68% female) with chronic headache underwent an MRI and diffusion tensor images were obtained. Fractional anisotropy (FA, a quantitative measure of brain microstructure) was acquired across 10 bilateral white matter tracts. Prior to the MRI, participants wore an actigraph for one-week to measure their average activity per minute. Moreover, youth completed measures of pain interference, anxiety, and depression. Generalized linear models were used to evaluate the relationship between physical activity, brain connectivity, and pain interference.

**Results**: Participants with higher average activity per minute demonstrated less pain interference. Lower physical activity was associated with higher pain interference with increased connectivity of the left inferior fronto-occipital fasciculus, right superior longitudinal fasciculus, and right uncinate, as compared to youth with higher physical activity per minute.

**Discussion/Conclusions**: Greater physical activity ameliorated the relationship between higher brain white matter connectivity and greater pain interference in youth with chronic headache.

**Trauma-related increases in brain white matter connectivity decreases health-related quality of life**

Neta Bar Am^a^, Daniel Kopala-Sibley^a^, Nivez Rasic^b^, Catherine Lebel^a^, Melanie Noel^a^, Richelle Mychasiuk^c^ and Jillian Vinall Miller^a^

^a^University of Calgary; ^b^Alberta Children’s Hospital; ^c^Monash University

**Introduction/Aim**: By 16 years of age, 65% adolescents will have had at least one adverse childhood experience (ACE), and approximately 13% of these youth will develop post-traumatic stress symptoms (PTSS). PTSS can influence the structural connectivity of brain regions involved in emotional processing (e.g., cingulum and uncinate fasciculus), as well as health-related quality of life (HRQOL). However, the relationships between PTSS, brain white matter connectivity, and HRQOL are not known.

**Methods**: 50 youth, aged 14-18 years underwent 3T MRI, and answered questionnaires regarding ACEs, PTSS and HRQOL, two times, approximately 3 months apart. DTI was acquired, and quantitative measures of white matter connectivity (fractional anisotopy (FA) values, axial diffusivity (AD) and radial diffusivity (RD)) were obtained bilaterally from the cingulum and the uncinate fasciculus. Generalized estimating equations were used to examine the relationships between ACEs, PTSS, HRQOL, cingulum and uncinate FA, accounting for age.

**Results**: Higher ACEs, CPSS, and FA of the left cingulate and uncinate were related to lower HRQOL. These factors specifically affected Physical HRQOL, as opposed to Emotional, School or Social HRQOL. Higher ACEs and CPSS and lower Physical PedQL were associated with lower RD of the left cingulum and uncinate, and lower AD of the left uncinate.

**Discussion/Conclusions**: Early exposure to trauma may have increased the rate white matter maturation in both the uncinate and cingulum. Previously, we demonstrated that youth with chronic pain had greater cingulum connectivity as compared to health controls. Trauma-related maturation of the uncinate and cingulum may increase the risk for the chronification of pain.

**Diffusion MRI of the accumbofrontal tract: A longitudinal study in chronic low back pain**

Paul Bautin, Monica Sean and Pascal Tétreault

University of Sherbrooke

**Introduction/Aim**: Structural and functional MRI studies suggest implication of the corticolimbic circuitry in chronic pain. This study explores white matter microstructure and connectivity properties of the accumbofrontal tract (NAc-mPFC) in chronic low back pain patients (CLBP) and healthy controls (HC).

**Methods**: We evaluate and compare the accumbofrontal tract structure in both CLBPS (n=27) and healthy controls (n=25) at 3 time points (0, 2 and 4 months) with a fully automated pipeline. Notably, we segment the NAc-mPFC using anatomical priors from the brainnetome atlas and compare along tract profiles (tractometry) across free water corrected DTI, HARDI, NODDI metrics and cross-metric PCA on CLBP vs. healthy controls.

**Results**: Visual quality control and statistics on bundle streamlines (count, length) demonstrate that the NAC-mPFC tract can be segmented most successfully with endpoints in brainnetome regions 123 (NAc) and 27 (middle frontal gyrus). When nearing the cortex, the accumbofrontal tract shows segments with higher NuFO (number of fiber orientation), AFD (apparent fiber density) and first PCA component values in CLBPS than in healthy controls. In the middle of the bundle, segments show lower FA (fractional anisotropy), AD (axial diffusivity), second PCA component and higher ODI (Orientation dispersion index) values in CLBPS than in healthy controls.

**Discussion/Conclusions**: Further investigations are needed to assess segmentation reproducibility, connectivity metrics and relationship with clinical questionnaires evaluating catastrophization, mood and symptom severity. However, preliminary observations suggest: (i) loss of complexity towards the mPFC and (ii) loss of fiber coherence in the middle of the bundle.

**The role of metabolite variability in the anterior cingulate cortex in predicting pain sensitivity: A magnetic resonance spectroscopy study**

Cassandra Choles^a^, Jessica Archibald ^a^, Erin L MacMillan^b^ and John L. K. Kramer^c^

^a^University of British Columbia, International Collaboration on Repair Discoveries (ICORD); ^b^University of British Columbia, ImageTech Lab, Simon Fraser University, Philips Healthcare Canada; ^c^University of British Columbia, International Collaboration on Repair Discoveries (ICORD), Djavad Mowafaghian Center for Brain Health (DMCH), Hugill Anesthesia Research Centre

**Introduction/Aim**: Pain is a complex, multifaceted experience with substantial sources of variability within and between individuals. Investigation into the variability of neuronal communication and metabolism within the brain provides insight into how pain is processed and interpreted. Magnetic resonance spectroscopy (MRS) in the cingulate cortex, known for its role in pain perception, provides an avenue to quantify the excitatory neurotransmitter glutamate, its precursor glutamine, and other metabolites involved in the normal functioning of neurons (creatine, inositol, and N-acetyl aspartate).

**Methods**: Previous research by Archibald et al (2020) demonstrated that glutamate concentrations increased at the onset of acute pain exposure, compared to resting state, in the anterior cingulate cortex in ‘healthy’ participants. Through a secondary analysis of this raw data, we investigated whether the resting variability of metabolites was influenced by pain exposure, and whether sex effects were present.

**Results**: Preliminary results demonstrate glutamate had significantly less variability after pain exposure, t(14)=2.61, p=0.016. The metabolites glutamine, creatine, inositol, and N-acetyl aspartate followed this trend but did not reach significance (p > 0.05). Females were found to have increased metabolite variability before and after pain exposure, in comparison to males; however, this trend did not reach significance.

**Discussion/Conclusions**: This provides further insight into the importance of glutamatergic resting variability, and its contribution to pain perception. Areas of continued investigation include sex differences, expanding MRS to other cingulate regions, and resting metabolite variability of individuals with chronic pain. This research contributes to our understanding of pain perception, and pharmacological insight for more effective pain management.

**Brain functional connectivity changes following exposure to either sevoflurane- or propofol-based anesthesia in children undergoing MRI**

Karen L. Cobos, Jillian V. Miller, Allison McPeak, Filomeno Cortese, Adam O. Spencer, Naweed Syed, Andrew Walker, Nivez Rasic, Melanie Noel and Tiffany K. Rice

University of Calgary

**Introduction/Aim**: In children, MRIs are often performed using either a volatile-based anesthetic (i.e., sevoflurane), or a total intravenous anesthetic (i.e., propofol). Evidence from animal models suggests that these anesthetic agents could adversely affect cognition and behavior. We compared changes in functional connectivity in brain areas that could affect cognitive and behavioral function between children exposed to either sevoflurane- or propofol-based general anesthesia (GA) for MRI.

**Methods**: Participants included 50 healthy children between the ages 1.5 to 5 years who required GA for MRI. Children were randomized to receive either sevoflurane- or propofol-based GA. Resting-state functional MRI (rs-fMRI) data was acquired. Independent sample t-tests were used to compare rs-fMRI data between sevoflurane- and propofol-exposed groups.

**Results**: Children who received sevoflurane GA demonstrated alterations in functional connectivity compared to children who received propofol GA. Namely, the sevoflurane group appeared to have greater connectivity of: 1) the anterior cingulate to the insular cortex, precuneus cortex, and lingual gyrus; 2) the posterior cingulate to the anterior cingulate, frontal pole, middle frontal gyrus and insular cortex; and 3) the posterior lobe to the frontal pole, anterior cingulate gyrus, parahippocampal gyrus and paracingulate gyrus; brain regions critically involved in both cognitive and behavioral functioning.

**Discussion/Conclusions**: Administration of sevoflurane for MRI may lead to greater changes in brain functional connectivity than propofol. The next step will be to examine how differences in functional connectivity relate to the cognitive and behavioral outcomes of these two groups.

**Trauma alters brain white matter connectivity and increases pain sensitization in LGBTQ2S+ youth**

Adeline Eldred, Nils Forkert, Nivez Rasic, Catherine Lebel, Melanie Noel, Daniel Kopala-Sibley and Jillian Miller

University of Calgary

**Introduction/Aim**: Lesbian, gay, bisexual, transgender, queer, and two-spirit (LGBTQ2S+) individuals may experience higher rates of trauma than their non-LGBTQ2S+ peers. Greater posttraumatic stress symptoms (PTSS) have been shown to increase the risk of developing chronic pain. However, it is unknown whether LGBTQ2S+ youth have higher rates of chronic pain, and whether this is due to PTSS-related changes to the brain.

**Methods**: 50 youth aged 14-18 years, were assessed twice, at an interval of three months. At each visit, participants reported their gender, sexuality, pain symptomology, number of adverse childhood experiences (ACEs), and PTSS. Further, they underwent quantitative sensory testing (QST), to examine mechanical and thermal thresholds, and were scanned using a 3T GE MRI fractional anisotropy (FA), to quantitatively measure brain white matter connectivity from their diffusion tensor images, at each visit. A repeated measures ANOVA will be used to compare trauma and pain symptomology between groups. A moderated mediation analysis will be conducted using PROCESS to examine the relationships between trauma, FA, and pain symptomology between groups.

**Results**: 13/50 (26%) of youth identified as LGBTQ2S+. Youth identifying as LGBTQ2S+ reported greater ACEs and PTSS and had a higher cold threshold relative to their heterosexual, cisgender peers (p < 0.05). It is predicted that greater PTSS will be associated with decreased whole-brain FA, resulting in higher thresholds, particularly for LGBTQ2S+ youth.

**Discussion/Conclusions**: LGBTQ2S+ youth may experience higher levels of trauma and lower whole-brain connectivity, possibly resulting in a higher risk of developing chronic pain as compared to non-LGBTQ2S+ youth.

**Evaluating a-priori neuromarkers for chronic pain: Generalizability and sex differences**

Matt Fillingim^a^, Christophe Tanguay-Sabourin^b^, Gianluca Guglietti^a^, Azin Zare^a^, Jax Norman^a^ and Etienne Vachon-Presseau^a^

^a^Mcgill; ^b^University of Montreal

**Introduction/Aim**: This study evaluated a set of a-priori brain-based biomarkers (neuromarkers) of chronic pain in two datasets: UKBiobank (n=37,781) and Open Pain (n=353). We aimed to assess the robustness of these neuromarkers in predicting chronic pain-associated illnesses and examined sex-specific differences in neuromarker expression.

**Methods**: We evaluated 13 a-priori neuromarkers in 24 pain-associated illnesses. We used random-effects ANCOVA and Area Under the Curve (AUC) scores to compare the neuromarkers between the illness groups and illness-free controls. We examined neuromarker expression within each sex separately, after matching the sexes on number of self-reported pain sites.

**Results**: The Neuromarkers performed best in discriminating subjects reporting fibromyalgia and chronic fatigue syndrome (AUC Max: .61 and .60). In both datasets (UKBB and Open Pain), and across Neuromarkers, we found that women with chronic pain were more easily discriminated from their pain-free counterparts than men, [Open Pain Female/Male AUC avg: .58/.54; UK Biobank Female/Male AUC avg: .53/.51].

**Discussion/Conclusions**: We observed varying neuromarker expression across specific chronic pain conditions, suggesting that the neural mechanisms underlying different chronic pain subtypes may differ. Additionally, neuromarkers showed increased discriminability in females, indicating that sex-specific differences in brain function may play a role in the underlying neural mechanisms of chronic pain.

**Evaluating the brain regions implicated in descending pain modulation and their role in endometriosis associated pain: An fMRI study investigating offset analgesia**

Scott Holmes^a^, Jenny Kim^b^, Gabriela Comptdaer^b^, Edina Szabo^a^, Claire Lunde^c^, David Borsook^d^ and Christine Sieberg^e^

^a^Boston Children’s Hospital/Harvard Medical School; ^b^Boston Children’s Hospital; ^c^Boston Children’s Hospital/Oxford University; ^d^Massachusetts General Hospital and ^e^Boston Children’s Hospital/Biobehavioral Pain Innovations Lab

**Introduction/Aim**: Endometriosis is a chronic pain condition that impacts women of reproductive age with few available treatments. There has been little research attention on pain processing in this cohort, particularly as it relates to how pain is processed and modulated in the central nervous system. Our lab has developed an fMRI-based thermal pain sensitivity protocol to evaluate the brain regions implicated in descending pain modulation (offset analgesia). Objective: To understand the brain regions implicated in descending pain modulation in persons with surgically confirmed endometriosis.

**Methods**: A total of six participants - biologically female at birth - were recruited who had surgically confirmed endometriosis. Each participant completed an MRI study session that included rating their 7/10 pain thresholds in the MRI scanner, and then completing three blocks of a task-based fMRI thermal paradigm (and one rest condition) that included offset analgesia, a control condition, and conditioned pain modulation. Participants rated their level of pain using a Likert scale (from one to ten) presented in the MR scanner and manipulated their response using hand-held triggers.

**Results**: Findings from this preliminary analysis suggest that offset analgesia involves increased activity (relative to rest) in frontal regions including the medial frontal lobe, frontal pole, and the rostral anterior cingulate gyrus (ps < 0.1).

**Discussion/Conclusions**: Findings from this investigation highlight the ability to perform an offset analgesia paradigm in an fMRI scanner and the ability to resolve central nervous system processing of painful stimuli in persons with endometriosis. This preliminary cohort is being expanded to help support the role of potential neuromodulatory investigations as a possible treatment tool for persons of all ages with endometriosis associated pain.

**Resting state functional connectivity of the extrastriate body area in complex regional pain syndrome patients: A pilot study**

Matthew Mockford^a^, Jenny Lewis^b^ and Massieh Moayedi^a^

^a^University of Toronto; ^b^University of the West of England

**Introduction/Aim**: Complex regional pain syndrome (CRPS) occurs commonly following trauma to a limb, is accompanied by various sensorimotor problems, and is of unknown etiology. Patients with CRPS often report that their affected limb is distorted—i.e., body perception disturbances (BPD). We have previously shown that changes to body image are mediated by the extrastriate body area (EBA) and its connectivity to the posterior parietal cortex. Here, we investigated whether the EBA in CRPS patients shows abnormal connectivity compared to pain-free individuals using functional magnetic resonance imaging (fMRI), and whether this is related to the extent of BPD.

**Methods**: 44 participants (20 upper limb CRPS, 24 healthy; 8 males, 36 females) aged (mean ± SD) 53.1 ± 13.5 years consented to procedures approved by NIHR and UWE ethics. Participants underwent an EBA functional localizer task and resting state fMRI. We investigated EBA functional connectivity to the rest of the brain, and the relationship with BPD. Given the exploratory nature of this study, significance was set at p < 0.001, cluster extent k > 10.

**Results**: In CRPS, the left EBA had stronger connectivity with the hand region of the left somatosensory cortex (S1; p < 0.001 uncorrected, k=21). BPD scores correlated with connectivity between the right EBA and bilateral posterior insula, right S1, and other body image areas.

**Discussion/Conclusions**: EBA connectivity to S1 was stronger in patients with CRPS, indicating the contribution of body image and body perception disturbances to chronic pain. Future studies are required to validate these findings and determine the causal role of the EBA in CRPS.

**Cervical muscle as possible predictors of postoperative prognosis in patients with DCM**

Neda Naghdi^a^, James M. Elliott^b^, Michael H. Weber^c^, Michael G. Fehlings^d^ and Maryse Fortin^e^

^a^Concordia University; ^b^The University of Sydney; ^c^McGill University Health Centre; ^d^University of Toronto; ^e^Concordia University

**Introduction/Aim**:

**Objective**: To examine whether preoperative cervical muscle size, composition and asymmetry from magnetic resonance imaging (MRI) can predict post-operative outcomes in patients with degenerative cervical myelopathy (DCM).

**Methods**: A total of 171 patients with DCM were included. Relative total cross-sectional area (RCSA), functional CSA (fat free area, FCSA), ratio of FCSA/CSA (fatty infiltration) and asymmetry of the multifidus (MF) and semispinalis cervicis (SCer) together (MF+SCer), and cervical muscle as a group (MF, SCer, semispinalis capitis, splenius capitis) were obtained from T2-weighted axial MR images at mid-disc, at the level of maximum cord compression and the level below. Univariate and multivariate linear regression analyses adjusting for age, BMI and sex were used to assess the relationship between baseline cervical muscle measurements of interest with mJOA, Nurick and NDI scores at 6-month and 12-month post-surgery.

**Results**: Lower RCSA of MF+SCer, less CSA MF+SCer asymmetry and greater FCSA/CSA for the cervical muscle group (e.g., less fatty infiltration) and younger age (p-value=0.024) were significant predictors of higher mJOA scores (e.g., less disability) at 6-month and 12-months post-surgery (all p < 0.05). Greater CSA asymmetry in MF+SCer and lower FCSA/CSA (e.g., more fatty infiltration) for the cervical muscle group were significant predictors of higher Nurick scores (e.g., more disability) at 6-months and 12-months post-surgery. While greater RCSA MF+Scer was associated with higher NDI scores at 12-months.

**Discussion/Conclusions**: Our result suggested that cervical paraspinal muscle morphology, specifically greater asymmetry, and fatty infiltration may be important predictors of functional recovery and post-surgical outcomes in patients with DCM.

**Traumatic experiences reduce brain efficiency to increase pain intensity in youth**

Tarannum Rahnuma, Samantha Miller, Karen L. Cobos, Nivez Rasic, Xiangyu Long, Catherine Lebel, Neta Bar Am, Melanie Noel, Daniel Kopala-Sibley, Richelle Mychasiuk and Jillian V. Miller

University of Calgary

**Introduction/Aim**: Youth with chronic pain (pain > 3 months) report elevated posttraumatic stress symptoms (PTSS) and adverse childhood experiences (ACEs) compared to healthy peers. Early-life trauma is known to impede brain function, but the relationships between trauma, functional connectivity and pain symptomology remain underexplored.

**Methods**: Questionnaire (i.e., PTSS, ACEs, pain intensity) and resting-state fMRI data were collected from 50 youth aged 14-18 years at two times, three months apart. Functional connectivity was assessed using graph theory metrics, (i.e., clustering coefficient and path length). Differences in functional connectivity metrics between baseline and follow-up were assessed using paired-samples t-tests. Generalized estimating equations were used to investigate the relationship between PTSS, ACEs and functional connectivity measures, accounting for age, gender, and pubertal status. The extent that ACEs and PTSS moderated brain functional connectivity to increase pain intensity was also explored.

**Results**: There were no significant differences in graph metrics between baseline and follow-up. Higher ACEs were associated with increased path length (P < .05). Greater path length was associated with increased pain intensity in youth with higher PTSS (P < .05).

**Discussion/Conclusions**: Youth with greater exposure to early-life trauma appear to have reduced brain efficiency as compared to youth with lesser exposure to trauma. Greater PTSS and reduced brain efficiency may increase pain intensity, and thereby increase the risk for chronification of pain. Early identification of PTSS and treatment are necessary to reduce the burden of pain.

**The assessment of paraspinal muscle epimuscular fat in subjects with and without low back pain: A case-control study**

Brent Rosenstein^a^, Jessica Burdick^a^, Alexa Roussac^a^, Meaghan Rye^a^, Neda Naghdi^a^, Stephanie Valentin^b^, Theresia Licka^c^, Monica Sean^d^, Pascal Tétreault^d^, Jim Elliott^e^ and Maryse Fortin^a^

^a^Concordia University; ^b^University of the West of Scotland; ^c^University of Veterinary Medicine Vienna; ^d^Université de Sherbrooke; ^e^University of Sydney

**Introduction/Aim**: Measures obtained from conventional radiologic imaging of the lower back are poor predictors of LBP severity and future outcomes but paraspinal muscle characteristics are seldomly explored. This project aims to 1) compare epimuscular fat in subjects with and without chronic LBP, and 2) Determine whether epimuscular fat is associated with spinal levels, BMI, age, sex and LBP status, duration or severity.

**Methods**: Fat and water lumbosacral MRIs of 50 participants with chronic LBP and 37 controls were used. The presence and extent of epimuscular fat for the paraspinal muscle group (erector spinae and multifidus) from L1-L5 to L5-S1 was assessed using a qualitative score (0-5 scale; 0=no epimuscular fat and 5=epimuscular fat present along the entire muscle) and quantitative manual segmentation method. Chi-squared tests evaluated associations between qualitative epimuscular fat and LBP status at each lumbar level. Pearson and Spearman’s correlation assessed relationships between quantitative and qualitative epimuscular fat with subjects’ characteristics.

**Results**: Epimuscular fat was more frequent at the L4-L5 (X2=14.619, p=0.012) and L5-S1 level (X2=24.437, p < 0.001) in subjects with LBP as compared to controls. The total qualitative score (combined from all levels) showed a significant positive correlation with BMI, age, sex (female) and LBP status (r=0.23-0.55; p < 0.05). Similarly, the total area of epimuscular fat (quantitative measure) was significantly correlated with BMI, age and LBP status (r=0.26-0.55; p < 0.05). No correlations were found between epimuscular fat and LBP duration or severity.

**Discussion/Conclusions**: Paraspinal muscle epimuscular fat is more common is subjects with chronic LBP. The functional implications of epimuscular fat should be further explored.

**Stability of hippocampal subfields and clinical questionnaires in patients with chronic low back pain and healthy controls: A longitudinal 4-months study**

Monica Sean, William Nadeau, Kevin Whittingstall, Guillaume Léonard and Pascal Tétreault

Université de Sherbrooke

**Introduction/Aim**: Few studies have investigated the role of hippocampal subfields in chronic low back pain (CLBP) patients. The goals were to evaluate the relationship between hippocampal subfields and physical/psychological components, and the stability of these findings.

**Methods**: We recruited 25 healthy controls (HC) and 23 CLBP subjects to acquire brain structural magnetic resonance imaging and various questionnaires at baseline (V1), and at 2 months (V2) and 4 months (V3) after baseline. Volumetric values of 3 hippocampus subfields (Cornu Ammonis [CA]1-3, CA4-Dentate Gyrus [DG] and the subiculum) were obtained using the automated pipeline HIPS from VolBrain and corrected for intracranial volume. The questionnaires investigated were the PainDETECT (PD), Brief Pain Inventory (BPI), Pain Disability Index (PDI), and State-Trait-Anxiety Inventory (STAI-S/T).

**Results**: In CLBP patients, findings remained stable only at V1 and V2[GL1] [MS2]. Spearman’s correlation showed positive relationships between the right CA1-3 volume and higher pain intensity (r > 0.43; p < 0.04) and average pain intensity (r > 0.52; p < 0.01) reported in the PD. There was also a positive relationship between the bilateral CA1-3 volume and physical function BPI (r > 0.63, p < 0.001). Finally, there was a positive relationship between the left CA1-3 volume and PDI score (r > 0.49; p < 0.01). [GL3] [MS4]

In HC, findings remained stable over the three visits. Spearman’s correlation showed positive relationships between the STAI-T and right CA4-DG (r > 0.40; p < 0.04) and bilateral subiculum (r > 0.54; p < 0.006).

**Discussion/Conclusions**: Interestingly, CA1-3, but not CA4-DG or subiculum, was linked to CLBP symptoms compared to HC. Investigating hippocampal properties and their relationship to physical/psychological data in CLBP could reveal the interplay between hippocampus function and behavioral phenotype.

**Identifying chronic low back pain patients using machine learning on graph theory measures from functional brain MRI: Preliminary results**

Mahsa Vafaei, Monica Sean and Pascal Tetreault

Faculty of Medicine and Health Sciences Université de Sherbrooke

**Introduction/Aim**: Chronic low back pain (CLBP) is the most prominent type of musculoskeletal pain in the world. Pain is by nature subjective, which means people report their pain based on their life experiences, psychological conditions, past trauma, etc. We aim to identify an objective method to classify healthy controls (HC) from CLBP patients. Several machine learning-based approaches have been investigated to classify HC from CLBP but there is no clinical or real-world application for these approaches and one problem is the reproducibility of results.

**Methods**: To address this problem, we are investigating the brain properties of 52 subjects, 27 suffering from CLBP and 25 HC (age and sex-matched). All participants are followed for 4 months, with visits at 0, 2, and 4 months. We used resting-state functional MRI images of each visit to model brain gray matter (GM) as a graph using fMRI Blood Oxygen Level Dependent (BOLD) signal at 8 mm3 resolution. Then, we applied a support vector machine to classify CLBP patients from HC over three visits.

**Results**: Our preliminary results show that we were able to classify patients from controls with fMRI data up to ~75% at the first and second visits. However, this accuracy plummets to ~50% on the third visit. On an out-of-site dataset (CLBP and HC from OpenPain.org), we were able to reach ~70% accuracy.

**Discussion/Conclusions**: We are currently investigating the relationship between misclassified participants and their behavioral profiles as this could explain the lack of accuracy at visit 3.

**Gender/sex differences Les différences entre les genres et les sexesUnderstanding chronic pain through a gender lens**

Fiona Webster^a^, Laura Connoy^b^, Kathleen Rice^c^, Craig Dale^d^, Abhimanyu Sud^d^ and Joel Katz^e^

^a^University of Western Ontario; ^b^Western University; ^c^McGill University; ^d^University of Toronto; ^e^York University

**Introduction/Aim**: Gender is a broad category distinct from sex that offers a lens through which to understand how and in what ways social factors (such as caregiving roles) shape experiences of pain and pain management. While it is well known that women disproportionately experience chronic pain, their pain is taken less seriously, and is often discounted. Additional inequities faced by women include poverty and single parenting responsibilities which directly shape experiences with, and management of, chronic pain. In this regard, while it important to include gender in chronic pain research, it is not enough; we must also consider intersections of socio-economic class, racialized identity, sexual orientation, migration status, etc. Drawing on our study findings, we present chronic pain experiences among women in Canada who identify as being socially and economically marginalized. Our goal is to offer politicized accounts of chronic pain that begin with standpoint and illuminate the connections between personal experience and larger social systems and structures.

**Methods**: We conducted a secondary analysis of data collected for a study using the sociological approach of institutional ethnography (IE) on experiences of chronic pain and marginalization. During the original study, we had not anticipated that the majority of our participants would in fact be seemingly cisgender women. Given this, it seemed clear that a gender-based analysis was necessary for much of our data. IE allows our research team to investigate the work of people living with chronic pain, from the standpoint (social positioning or location) of those whose lives shaped by processes of marginalization. For the purposes of this secondary analysis, we begin in the standpoint of women living with chronic pain and marginalization. In our primary study, we purposively recruited people living in Canada, who are over the age of 18 years, speak fluent English, who identified as living with chronic pain, and consider themselves as struggling to make ends meet. We recruited participants through Twitter and Kijiji, and in public areas such as laundromats and libraries, and through the clinic of one of our team members using posters and postcards, and through the newsletters of leading pain organizations (Pain B.C. and Canadian Arthritis Patient Alliance). Due to the pandemic, this study relied on telephone interviews. Participants were asked to describe what they do as they go about performing their everyday lives. All interviews were audio recorded and professionally transcribed and then indexed into themes.

**Results**: We have organized our preliminary findings around five inter-related themes that constitute women’s experiences of encountering marginalization and gender bias in the context of chronic pain: 1) gendered poverty; 2) inter-generational poverty and its links with abuse; 3) stigmatized pain conditions; 4) relational aspects of pain management; and, 5) mothering.

**Discussion/Conclusions**: People’s standpoint – that is, where one is located within existing social systems - can directly and/or indirectly facilitate or impede the time and effort that goes into managing chronic pain. The social construct of, and associated norms attributed to, gender significantly impacts how chronic pain is experienced among women in Canada. As a gendered experience, chronic pain is informed by social inequities and gender stereotypes that must be contextualized. The preliminary findings from our research make an important contribution to understanding the largely ignored work that people who are dealing with poverty, chronic pain and gender biases perform. Researchers and primary care providers must think about how gender influences how people’s stories about chronic pain experiences and treatments are heard. One means to do this is through more critical and sociological research that gets beyond sex difference.

**Stereotypically masculine or feminine personality traits and cannabis use for chronic pain management**

Marimée Godbout-Parent^a^, Nancy Julien^a^, Hermine Lore Nguena Nguefack^a^, Claudie Audet^a^, M. Gabrielle Pagé ^b^, Line Guénette^c^, Lucie Blais ^b^ and Anaïs Lacasse^a^

^a^Université du Québec en Abitibi-Témiscamingue (UQAT); ^b^Université de Montréal; ^c^Université Laval

**Introduction/Aim**: There has been a significant increase in cannabis use for chronic pain (CP) management. The literature regarding gender differences in the use of cannabis to treat CP is contradictory. This study aimed to assess the association between gender roles, their interaction with gender identity and cannabis use for CP management.

**Methods**: This analysis was conducted using the COPE Cohort, a database resulting from a web-based survey among people living with CP across Quebec (Canada). Measurements included past year use of cannabis for CP management (yes/no), and the Bem Sex-Role Inventory (BSRI) which assess stereotypically masculine or feminine personality traits and categorize participants into four gender role subgroups (feminine, masculine, androgynous, undifferentiated). Gender identity was defined as self-identification as woman, man, or non-binary.

**Results**: Among the 1,120 women, 216 men and 4 non-binary participants, prevalence of cannabis use for CP management among gender role subgroups was estimated as follows, feminine: 33.0%, masculine: 32.0%, androgynous: 27.7%, undifferentiated: 29.4% (p > .05). Adjusting for potentially confounding variables in a multivariable model, neither gender roles nor gender identity or the statistical interaction between these two gender constructs were associated with cannabis use (p > .05). Analyzing the BSRI as continuous scores did not yield different results.

**Discussion/Conclusions**: Persons with stereotypically masculine or feminine personality traits or identifying as women or men did not appear to differ in their use of cannabis for CP management. Our multivariable analysis suggests that the differences between men and women found in the literature are potentially explained by other bio-psycho-social factors.

**Men show greater association between chronic pain and workplace stress**

Gianluca Guglietti^a^, Matthew Fillingim^a^, Azin Zare^b^, Jax Norman^a^, Christophe Tanguay-Sabourin^c^, Luda Diatchenko^a^ and Etienne Vachon-Presseau^a^

^a^McGill University; ^b^McGill; ^c^Université de Montréal

**Introduction/Aim**: Both sex and socioeconomic status (SES) are shown to be associated with chronic pain, but the interaction of these factors is poorly understood. Increased physical and emotional stress can both mediate SES’s relationship with pain. In this study we investigate the interaction between these factors and sex on chronic pain outcomes.

**Methods**: We utilized two cohorts, the United Kingdom Biobank (UKB) (n=533,489) and Northern Finland Birth Cohort (NFBC) (n=12,231). Participants provide self-report of chronic pain and effected body sites. Questionnaires on job type, job satisfaction, and work-related physical stress were provided. Chi-squared test was used to determine differences in pain across job types. Linear regression was utilized to measure the association between pain severity and work-related factors after stratifying by sex.

**Results**: Job coding in the UKB is hierarchically coded using SOC2000 classification, chi-squared tests were performed at each level of classification. Both men and women showed consistent association between chronic pain and job coding with larger effects in among men (male: Cramer’s V=0.09-0.11, p=9.7*10-270-4.6*10-300; female: Cramer’s V=0.05-0.08, p=7.08*10-91-1.19*10-111). Men also showed a stronger association between number of pain sites and physical work (male: r=0.11,p=0; female: r=0.06,p=4.62*10-134), walking/ standing at work (male: r=0.08,p=2.98*10-216; female: r=0.04,p=4.93*10-64) and work satisfaction (male: r=0.09,p=1.01*10-147; female: r=0.07,p=3.72*10-129). In the NFBC men show higher associations between pain and job type; and complaints regarding work satisfaction across multiple measures even for those participating in “intellectual” non-physical work.

**Discussion/Conclusions**: Both physical and psychological workplace stress show associations with chronic pain across cohorts, with stronger effects in men.

**The intersection of gender, the veteran experience, and chronic pain**

Avery Hart^a^, Eleni G. Hapidou^b^ and Jennifer Anthonypillai^c^

^a^McMaster University; ^b^Hamilton Health Sciences, McMaster University; ^c^Michael G. DeGroote Pain Clinic, Hamilton Health Sciences

**Introduction/Aim**:

**Background**: Sex and gender moderate pain (Fillingim, 2017). Veterans are overrepresented in chronic pain (Sweet et al., 2020). Women and veterans undergoing interdisciplinary pain management demonstrate greater improvements than men and civilians (Jomy & Hapidou, 2020; Racine et al., 2020).

**Purpose**: The aim of this study was to examine how the veteran-woman intersectionality moderates chronic pain and response to treatment.

**Methods**: Data were collected from the five-week intensive interdisciplinary pain management program (adapted for the pandemic) at the Michael G. DeGroote Pain Clinic, Hamilton, ON (n=106, 44% females, 56% Veterans). Participants completed psychometrics assessing pain intensity (PIS), pain disability (PDI), kinesiophobia (TSK), anxiety (CAS), depression (CES-D), catastrophizing (PCS), sensitivity to pain traumatization (SPTS), pain stages of change (PSOCQ), pain acceptance (CPAQ) at admission and discharge, and self-evaluations of program benefit and satisfaction at discharge. 2x2x2 mixed ANOVAs on outcome measures and 2x2 ANOVAs on satisfaction measures were conducted.

**Results**: Improvements in all variables (p < 0.001) at discharge, main effects of gender on SPTS, PCS, TSK, SHS, Pre-cont (p < 0.05), and on Veteran-Civilian on the CES-D, PCS, CAS, PDI, PQ, SPTS, Pre-cont, Cont, Action and CAPQ, and interactions between gender and veteran-civilian (p < 0.05) for the contemplation and maintenance stages of change were obtained.

**Discussion/Conclusions**: Results replicate previous findings on the benefits of interdisciplinary pain management for all patients and highlight differences between men and women, veterans and civilians. Overall, women and veterans scored lower on several outcome variables. That women scored lower than men on pain traumatization has not been reported. It is recommended to explore the effect of gender further as it relates to pain traumatization and interactions between gender and veteran-civilian with larger sample sizes.

*Acknowledgment: This project was supported in part by funding from the Chronic Pain Network through the Strategy for Patient-Oriented Research (SPOR) (reference #SCA-145102), and the Chronic Pain Center of Excellence for Canadian Veterans.

**Molecular determinants of dorsal horn excitability and pain processing in male and female rats and humans**

Marrium Khan^a^, Laurence S. David^a^, Annemarie Dedek^b^, Jessica Parnell^b^, Eve Tsai^c^ and Michael Hildebrand^a^

^a^Carleton University, 21. Carleton University; ^b^Ottawa Hospital Research Institute; ^c^The Ottawa Hospital, Ottawa Hospital Research Institute, University of Ottawa

**Introduction/Aim**: Chronic pain represents a debilitating healthcare challenge. Although females report more incidences of pain, most pain research has not been sex inclusive and thus sex-specific therapeutics remain unexplored. To address this, we characterized the expression of molecular determinants of a canonical dorsal horn hyperexcitability pathway across sex. In this mechanism of pathological pain, BDNF-TrkB signalling drives disinhibition that couples to increased excitatory NMDA receptor activity only at male dorsal horn synapses. Here, we investigated whether differences in baseline expression of key players in this hyperexcitability mechanism, including the BDNF receptor, TrkB; the potassium-chloride cotransporter, KCC2; protein tyrosine phosphatase, STEP61; and NMDAR subunits, account for this sexually dimorphic pain processing across species.

**Methods**: Using RT-qPCR and western blot approaches on micro-dissected regions of spinal cord, we investigated baseline gene and protein expression profiles in the superficial dorsal horn (pain-processing; SDH) versus deep dorsal horn (other somatosensory modalities; DDH) regions. Viable cord tissue was immediately flash-frozen following collection from adult (3-4 months) male and female Sprague Dawley rats (n=8/sex) and from adult (20-70 years) human organ donors.

**Results**: Our findings suggest that distinct determinants of dorsal horn excitability, such as TrkB, KCC2 and STEP61, have differential relative expression in the SDH and DDH, both by target and by region. Moreover, preliminary analysis suggests potential sex differences in the SDH/DDH expression ratio for select molecular players.

**Discussion/Conclusions**: This study furthers our fundamental understanding of spinal mechanisms of pain processing, which may help identify future pain therapeutic targets that are efficacious across sex and species.

**Allostatic load and chronic pain in racial and ethnic minority groups from the UK biobank**

Jax Norman, Christophe Tanguay-Sabourin, Azin Zare, Gianluca Guglietti, Matthew Fillingim, Ronrick Da-ano and Etienne Vachon-Presseau

McGill University

**Introduction/Aim**: Allostatic load, the cumulative burden of stress, is associated with negative health outcomes, including chronic pain. Allostatic load has been proposed as a physiological mechanism of “weathering” – a theory which suggests racial and ethnic minority groups are at greater risk of adverse health outcomes due to long-term exposure to socioeconomic disadvantages and systemic inequities. This study aims to use allostatic load to investigate how chronic pain and chronic stress are associated within racial and ethnic groups.

**Methods**: This study used baseline anthropometric, metabolic, cardiovascular, and immune system measures and self-reported sociodemographic data from the UK Biobank. Odds ratios were used to measure the association between racial and ethnic identity and allostatic load scores, and the prevalence of chronic pain and widespread pain were calculated for each demographic group.

**Results**: We found that identifying as White was negatively associated with increased allostatic load in female participants (OR=0.75) and male participants (OR=0.83). Conversely, identifying as Black or Asian was positively associated with increased allostatic load in female participants (Black: OR=1.64; Asian: OR=1.21) and male participants (Black: OR=1.4; Asian: OR=1.14).

**Discussion/Conclusions**: We observed that allostatic load scores were lower in White participants than in Black and Asian participants of the same age. As allostatic load represents stress, these findings suggest that Black and Asian participants either experienced more stressors than White participants and/or the stressors experienced by Black and Asian participants had a greater impact on their physiological function.

**The role of testosterone on pain perception and conditioned pain modulation in eugonadal and hypogonadal men: A cross-sectional study**

Martine Bordeleau^a^, Guillaume Léonard^a^, Alexia Coulombe-Lévêque^a^, Matthieu Vincenot^a^, Isabelle Gaumond^b^, Philippe Chalaye ^b^, Jean-Luc Ardilouze ^c^, Serge Marchand^b^ and Catherine Pagé^a^

^a^Research Centre on Aging, Centre intégré universitaire de santé et de services sociaux de l’Estrie - Centre hospitalier universitaire de Sherbrooke, Sherbrooke, Quebec, Canada; ^b^Department of surgery, Faculty of Medicine and Health Sciences, Université de Sherbrooke, Sherbrooke, Quebec, Canada; ^c^Department of medicine, Faculty of Medicine and Health Sciences, Université de Sherbrooke, Sherbrooke, Quebec, Canada

**Introduction/Aim**: The way pain is perceived is highly variable among individuals and sex differences have been proposed to play a substantial role in this variability. Our aim was to evaluate the influence of free testosterone levels on pain sensitivity and descending pain modulation in groups of eugonadal (normal testosterone levels with > 210 pmol/L free testosterone) and hypogonadal (low testosterone levels with < 190 pmol/L free testosterone) men.

**Methods**: Twelve hypogonadal and 16 eugonadal men participated in this study. Heat pain threshold (HPT) and heat pain tolerance thresholds (HPTT) were evaluated with a contact thermode placed on the left forearm. Conditioned pain modulation (CPM) responses were obtained by comparing thermode-induced heat pain scores (2 minutes at constant temperature) before and after activating CPM triggered using a standard counter–irritation paradigm (i.e., immersion in cold water).

**Results**: HPT (45.0°C vs 47.0°C; p=0.02) and HPTT (49.1°C vs 50.1°C; p=0.02) were lower in hypogonadal men compared to the eugonadal men. Free testosterone levels were positively correlated with HTTP (r=0.40, p=0.04). Hypogonadal and eugonadal men showed comparable CPM responses (0-100 pain score delta of 9.7 vs 9.1, p=1.00).

**Discussion/Conclusions**: Healthy men with higher testosterone levels show reduced pain sensitivity compared to hypogonadal men. However, no between-group differences were observed for CPM responses, suggesting that male hormone concentration does not affect the efficacy of descending inhibitory systems.

**Sex differences in post-traumatic stress symptoms following musculoskeletal injury**

Antonina Pavilanis^a^, Heather Adams^b^, Maria Milioto^c^, Nicolas Houlachi^d^ and Michael Sullivan^a^

^a^McGill University; ^b^Dalhousie University; ^c^Lifemark; ^d^Physiothérapie Universelle

**Introduction/Aim**: Increasingly, research has revealed that individuals can experience debilitating symptoms of post-traumatic stress even when the precipitating event does not meet criteria for a ‘trauma.’ Recent evidence suggests that post-traumatic stress symptoms (PTSS) are prevalent and elevated following musculoskeletal injury, particularly in women. The purpose of the present study was to examine the factors that underlie sex differences in PTSS in individuals who have sustained a work-related musculoskeletal injury.

**Methods**: A sample of 187 (95 women and 92 men) completed measures of pain, PTSS, catastrophic thinking and perceived injustice within 6 weeks of submitting a time-loss claim for a work-related musculoskeletal injury.

**Results**: Analyses revealed that women, compared to men, had more severe and prolonged symptoms of pain at the time of enrolment, and scored higher on a measure of PTSS. Regression analyses were conducted separately for women and men to identify factors that might underlie sex differences in PTSS. For women, pain severity, pain duration, catastrophic thinking and perceived injustice emerged as significant unique predictors of the severity of PTSS. For men, catastrophic thinking, perceived injustice, but not pain duration or pain severity contributed significantly to PTSS.

**Discussion/Conclusions**: The discussion explores possible explanations for why pain duration and severity contribute to PTSS in women and not in men. Clinical implications of the findings are also addressed.

**Predictors of the development of chronic pain following concussion in youth**

Atiqa Pirwani^a^, Melanie Noel^b^, Miriam Beauchamp^c^, William Craig^d^, Quynh Doan^e^, Roger Zemekf and Keith Yeates^b^

^a^University of Calgary; ^b^Psychology, University of Calgary, Calgary, Research Institute, Alberta Children’s Hospital, Hotchkiss Brain Institute, University of Calgary; ^c^Department of Psychology, Universite de Montreal and Ste Justine Hospital; ^d^Department of Pediatrics, University of Alberta and Stollery Children’s Hospital; ^e^Department of Pediatrics, University of British Columbia and BC Children’s Hospital; ^f^Department of Pediatrics and Emergency Medicine, Children’s Hospital of Eastern Ontario

**Introduction/Aim**: Concussions are one of the most common neurological injuries in youth. Although most youths recover from concussion in a few weeks, a subset of youth report persisting symptoms that can last months or years. Headache is the most common symptom that persists. Age, sex, and parental behavior can influence the transition to chronic pain. Therefore, this study explored the association that age, sex, and parental protectiveness have with post-injury pain in children and adolescents from shortly after concussion to 3 and 6 months later.

**Methods**: Participants included youth with concussion (N=633) or orthopedic injury (OI; N=334) between the ages of 8-16 years who were recruited from 5 pediatric hospitals in Canada. Injury information was collected during the visit and follow-up assessments were conducted 10 days, 3- and 6-months post-injury.

**Results**: Following concussion, pain ratings decreased over time. Age did not significantly predict pain ratings across time in either group. Sex was a significant predictor of pain across time, with girls reporting greater pain ratings compared to boys across time, in both concussion (p < 0.001) and OI (p < 0.001) groups. The concussion group reported higher pain ratings than OI group. The concussion group is expected to show larger differences in pain compared to the OI group at higher levels of parent protectiveness.

**Discussion/Conclusions**: This study will shed light on factors that predict pain in youth following concussion. Pain is underrecognized among youth with concussions; thus, these findings can inform future intervention and prevention efforts for this vulnerable population.

**Sex differences in astrocyte neuronal metabolic coupling during chronic inflammatory pain in the anterior cingulate cortex of mice**

Paige Reid, James Tang, Kaitlin Scherer, Danielle Halaz, Fariya Zaheer and Giannina Descalzi

University of Guelph

**Introduction/Aim**: 25% of Canadians over the age of 15 suffer from chronic pain, and women make up 67% of this group. Treatment remains inadequate, and new molecular targets to improve treatment efficacy are needed. The astrocyte-neuronal lactate shuttle (ANLS) is involved in the occurrence of long-lasting inflammatory pain in the anterior cingulate cortex (ACC) of male mice. Whether the ANLS contributes to inflammatory pain in female mice remains unknown. The present study aims to investigate the ANLS in the development of chronic inflammatory pain in the ACC of both male and female mice.

**Methods**: Adult Male and Female C57BL/6 mice were used for all experiments. Hind paw injections of Complete Freud Adjuvant (CFA) induced chronic inflammatory pain. Mechanical allodynia was confirmed using the Von Frey Test. Activation of the ANLS was examined through both measurement of lactate levels and lactate-related protein expression,3h, 24h, 3d and 7d following injection. Lactate levels were assessed using colorimetric assays and protein through western blot analyses.

**Results**: Female and male mice differed in the severity of mechanical allodynia displayed at early time points of pain chronification. Although both sexes showed a rapid, significant increase in lactate levels in the ACC, only male mice showed significant levels seven days following CFA Injection, compared to vehicle-injected controls. Expression of lactate-related proteins also differs between sexes across time.

**Discussion/Conclusions**: We identified sex differences within the ANLS in the ACC during chronic inflammatory pain development, reinforcing the need to examine both sexes when investigating molecular target treatment options in pain chronification.

**Sex differences in toddler pain behaviours, parental worry, and perceptions of acute pain**

Amy Stern^a^, Daniel Flanders^b^, Eitan Weinberg^b^, Hartley Garfield^c^, Deena Savlov^b^ and Rebecca Pillai Riddell^a^

^a^York University; ^b^University of Toronto; ^c^The Hospital for Sick Children

**Introduction/Aim**: Previous studies have shown a gender bias in parental judgements of infant pain, with fathers rating pain in males higher than females (Moon et al., 2008). Furthermore, the majority of studies investigating sex differences across childhood have found no significant differences in pain expression (Boerner et al., 2014). The current study aimed to examine sex differences (assigned sex at birth; male versus female) in toddlers’ pain expression, parental worry, and parental judgments of toddlers’ post-immunization pain.

**Methods**: Parent-toddler dyads (N=144) were videotaped during older toddler routine immunization appointments (18 and 24 months). Toddler pain was operationalized by facial activity and broader body movements using the Neonatal Facial Action System (NFCS; Grunau & Craig, 1987) and the Face, Legs, Activity, Cry, Consolability Scale (FLACC; Merkel, Voepel-Lewis, Shayevitz, & Malviya, 1997). Parent worry and pain judgements were rated using a visual analog scale (0-10).

**Results**: T-tests were conducted and assumptions were met with one exception (Welch correction used). Analyses suggested that there were no significant sex differences in toddlers’ behavioural expression of pain, in parent worry during the vaccination appointment nor in parents’ judgements of toddler pain post-vaccination.

**Discussion/Conclusions**: The results indicated no differences in the behavioural expression of toddler pain, parental worry, and judgements of toddler pain based on whether the child was reported as male or female. Future research should explore other factors that influence toddler pain expression and parents’ perceptions of infant pain.

**Education L’éducationTowards a new integrated service delivery model to tackle chronic pain after cancer: Investing in patient partner guided imagery training**

Caroline Arbour^a^, Karine Bilodeau^b^, David Ogez^c^, Danny Hjeij^d^ and Sandie Oberoi^e^

^a^Faculty of Nursing, Université de Montréal, Centre de recherche de l’Hôpital du Sacré-Cœur de Montréal, CIUSSS du Nord-de-l’Île-de-Montréal, Montreal, Quebec, Canada; ^b^Faculty of Nursing, Université de Montréal, Centre de Recherche de l’Hôpital de Maisonneuve-Rosemont (CRHMR), CIUSSS de l’Est-de-l’Île-de-Montréal, Montreal, Quebec, Canada; ^c^Faculty of Medicine, Department of Anesthesia, Université de Montréal, Centre de Recherche de l’Hôpital de Maisonneuve-Rosemont (CRHMR), CIUSSS de l’Est-de-l’Île-de-Montréal, Montreal, Quebec, Canada; ^d^Centre de recherche de l’Hôpital du Sacré-Cœur de Montréal, CIUSSS du Nord-de-l’Île-de-Montréal, Montreal, Quebec, Canada; ^e^Project Manager, Espace partenaire en cancérologie, CIUSSS de l’Est-de-l’Île-de-Montréal, Montreal, Quebec, Canada

**Introduction/Aim**: Chronic pain is highly prevalent after cancer. An integrated approach to pain management combining analgesic drugs and self-regulation practices such as guided imagery is strongly recommended in cancer survivors. However, access to professional coaching during patient uptake of guided imagery is limited, hence the need to involve patient partners in the process. This study explored the feasibility and acceptability of a training program on guided imagery in a cohort of patient partners.

**Methods**: An observational descriptive study with mixed data collection is ongoing. To date, eight patient partners have been recruited and have completed a 5-hour pre-validated training on guided imagery. Based on Kirkpatrick’s theoretical model, participants were videotaped and evaluated at different points during the training using a pre-validated grid. Descriptive statistics were performed to obtain quantitative measures of the feasibility of this training. Participants experience with the training was gathered through individual semi-structured interviews and analyzed using thematic analysis.

**Results**: Wilcoxon paired-sample tests (pre–post) showed improvement with large effect sizes in the total score of the guide imagery training grid (P=0.034, r=0.832) and improvement with large effect sizes on the relational item (P=0.018, r=0.930). Three themes emerged from the interviews: 1) A positive experience; 2) Feeling prepared to support others; and 3) Being hopeful about real-life implementation.

**Discussion/Conclusions**: Although preliminary, results from this project provide the first empirical evidence supporting patient partners training in guided imagery as part of a larger integrated cancer pain management initiative.

**Canadian health care professionals continuing professional development needs in pain management: A cross sectional survey**

Craig M. Dale^a^, Iacopo Cioffi^b^, Christine B. Novak^c^, Franklin Gorospe^b^, Laura Murphy^d^, Deepika Chugh^b^, Judy Watt-Watson^b^ and Bonnie Stevens^e^

^a^University of Toronto/Sunnybrook Health Sciences Centre; ^b^University of Toronto; ^c^The Hospital for Sick Children; ^d^University Health Network; ^e^The Hospital for Sick Children/ University of Toronto

**Introduction/Aim**: The Canadian Pain Task Force (CPTF) recommends health care professionals (HCPs) continually develop the competencies to provide effective pain care. However, little is known about how HCPs advance or maintain pain management competence. Our aim was to investigate pain Continuing Professional Development (CPD) needs, activities, and preferences among Canadian HCPs.

**Methods**: Using an existing HCP database, we conducted a self-administered, cross-sectional de-identified web survey of CPD needs, activities, and preferred learning modalities (December 2018-January 2019). We compared responses defined by work setting (academic versus non-academic) and professional attributes.

**Results**: Survey response rate was 57% (230/400). Respondents were primarily nurses (48%), university educated (95%), employed in academic hospital settings (62%), with ≥11-years post-license experience (70%). In an average week, most patients (> 50%) cared for presented with pain. Compared to those HCPs working in non-academic settings, HCPs in academic settings reported significantly higher acute pain assessment competence (mean 7.8/10 versus 6.9/10; P < 0.002) and greater access to pain specialist consultants (73% versus 29%; P < 0.0001). Chronic pain assessment competence was not significantly different between groups. Top learning needs included neuropathic, musculoskeletal, and chronic pain. Learning modalities (completed and preferred, respectively) were primarily informal and work-based: reading journal articles (56%, 54%), online independent learning (44%, 53%), and attending hospital rounds (43%, 42%). There were 17% of HCPs who had not completed any pain learning activities in the previous 12-months.

**Discussion/Conclusions**: Canadian HCPs require greater access to and participation in formal and informal CPD to build knowledge, skill, and confidence in pain management.

**The importance of patient-healthcare professional partnerships when developing a national online pain management and substance use disorder curriculum**

Lisa Graves^a^, Jeanne Mulder^b^, Annie-Danielle Grenier^c^, Catherine Lemyze^c^, Nancy Dalgarno^b^, Samsoor Akberzai^b^, Jennifer Turnnidge^b^, Robert Van Hoorn^d^, Amber Hastings-Truelove^b^ and Richard van Wylick^b^

^a^Western Michigan University; ^b^Queen’s University; ^c^Centre of Excellence on Partnership with Patients and the Public; ^d^Association of Faculties of Medicine of Canada

**Introduction/Aim**: Patient engagement in medical education is essential as patients are the population ultimately affected by the interventions. In collaboration with patient subject matter experts (SMEs), the Association of Faculties of Medicine of Canada is in the process of implementing postgraduate medical education and continuing professional development curricula on pain management and substance use disorder. Online modules were co-created by patient and healthcare SMEs. Fourteen SME teams developed 16 online modules. 

**Methods**: This phenomenological study explores the lived experience of the patient-healthcare SME relationship in co-creating curricular content. Thirteen patient and 16 healthcare SMEs were invited to participate in interviews. The transcripts were thematically analyzed.

**Results**: Eight patients and six healthcare SMEs participated in the interviews. Six themes represented the SME experiences: navigating collaborative processes and setting expectations, building collaborative partnerships grounded in mutual trust and respect, sharing experiences, empowering patient voices, confronting stigma, and producing educational resources. Participants identified the need for increasing communication, providing explicit expectations, and involving patients early in the process. They highlighted the importance of building the relationship, navigating power differentials, and sharing lived experiences. Participants who spoke about stigma discussed the biases within the healthcare system. Overall, participants believe that co-producing the curriculum through collaborative SME partnerships facilitated the development of high quality resources.

**Discussion/Conclusions**: Involving patient SMEs were critical to developing an effective curriculum. This project demonstrates how patient engagement in medical education could help address barriers in our healthcare system and how this project could serve as a roadmap for other patient-inclusive initiatives.

**Evaluation of the first Project Extension for Community Healthcare Outcomes (Project ECHO) indigenous chronic pain and substance use in Canada**

Andrew Koscielniak^a^, Paul Francis^a^, Teresa Trudeau-Magiskan^a^, Christopher Mushquash^b^, Donna Garstin^a^, Marinna Read^a^, Yaadwinder Shergill^c^, Andrea Furlan^d^, Natalie Zur Nedden^c^, Alex Falcigno^a^ and Patricia Poulin^c^

^a^St. Joseph’s Care Group; ^b^Lakehead University; ^c^The Ottawa Hospital Research Institute; ^d^University of Toronto

**Introduction/Aim**: Indigenous Peoples are disproportionally affected by chronic pain (CP). Improving health care providers’ (HCPs) ability to provide culturally safe care is a priority highlighted in Canada’s Truth and Reconciliation Calls to Action. We present the evaluation of our first pilot Project ECHO Indigenous CP and Substance Use (SU) continuing professional development series covering the following core curriculum: 1) Understanding Trauma; 2) History of Opioids in CP and Impact on Indigenous Peoples; 3) Clinical Monitoring and Opioid Tapering; 4) How Psychological Pain Manifests as Physical Pain; 5) Conducting an Assessment for CP &SU Through an Indigenous Lens; 6) Traditional Approaches in CP & SU Management.

**Methods**: Fifty-seven participants (21.1% nurses, 14% social workers, 12.3% physicians) completed an average of 4.96 sessions. We measured knowledge improvements through in-session polls using a 4-point Likert scale (not at all to greatly improved). We interviewed six participants and used the constant comparison method to identify themes for continuous quality improvement. We shared results with our Indigenous Advisory Board.

**Results**: Across the series, 77.8% to 94.1% of respondents indicated moderately or greatly improved knowledge relative to the 6 core themes of the curriculum. Over two-thirds (68.8%) of respondents agreed (41.2%) or strongly agreed (27.7%) with the satisfaction measures. Emerging themes from the interviews included an appreciation of the Medicine Wheel as a framework to assess and address pain.

**Discussion/Conclusions**: Our pilot resulted in improved self-perceived knowledge and a majority of respondents were satisfied with the program. Recommendations for program improvement will be presented, as well as future direction.

**Evaluation of an online educational program about opioids for chronic non-cancer pain**

Andrea Furlan^a^, Nagina Parmar^a^, Talia Varley^b^ and John Flannery^a^

^a^UHN; ^b^Cleveland Clinic

**Introduction/Aim**: Indigenous Peoples are disproportionally affected by chronic pain (CP). Improving health care providers’ (HCPs) ability to provide culturally safe care is a priority highlighted in Canada’s Truth and Reconciliation Calls to Action. We present the evaluation of our first pilot Project ECHO Indigenous CP and Substance Use (SU) continuing professional development series covering the following core curriculum: 1) Understanding Trauma; 2) History of Opioids in CP and Impact on Indigenous Peoples; 3) Clinical Monitoring and Opioid Tapering; 4) How Psychological Pain Manifests as Physical Pain; 5) Conducting an Assessment for CP &SU Through an Indigenous Lens; 6) Traditional Approaches in CP & SU Management.

**Methods**: Fifty-seven participants (21.1% nurses, 14% social workers, 12.3% physicians) completed an average of 4.96 sessions. We measured knowledge improvements through in-session polls using a 4-point Likert scale (not at all to greatly improved). We interviewed six participants and used the constant comparison method to identify themes for continuous quality improvement. We shared results with our Indigenous Advisory Board.

**Results**: Across the series, 77.8% to 94.1% of respondents indicated moderately or greatly improved knowledge relative to the 6 core themes of the curriculum. Over two-thirds (68.8%) of respondents agreed (41.2%) or strongly agreed (27.7%) with the satisfaction measures. Emerging themes from the interviews included an appreciation of the Medicine Wheel as a framework to assess and address pain.

**Discussion/Conclusions**: Our pilot resulted in improved self-perceived knowledge and a majority of respondents were satisfied with the program. Recommendations for program improvement will be presented, as well as future direction.

**Evaluation of interprofessional pain core competencies among pre-licensure healthcare professional students attending the interfaculty pain curriculum program**

Samah Hassan^a^, Andrea Furlan^b^, Sylvia Langlois^c^, Craig Dale Dale^d^, Iacopo Cioffi^e^ and Laura Murphy^b^

^a^The Institute of Education Research (TIER) -University Health Network (UHN); ^b^Toronto Rehabilitation Institute; ^c^University of Toronto; ^d^Lawrence S. Bloomberg Faculty of Nursing; ^e^Faculty of Dentistry, University of Toronto

**Introduction/Aim**: The Interfaculty Pain Curriculum (IPC) is a 20-hour pain program based on the International Association for the Study of Pain (IASP) interprofessional pain curriculum. More than 1000 University of Toronto prelicensure students from different faculties and departments attend the program annually. This study reports on the evaluation of student chronic pain competencies following completion of the curriculum.

**Methods**: Upon concluding the IPC, students were asked to complete the Pain Competence Assessment Tool (PCAT), a validated online tool assessing the IASP competency domains, either individually or in interprofessional groups. The PCAT scores range from 0 to 100% where scores lower than 65% indicate novice, between 65% and 85% competent, and above 85% expert.

**Results**: Individual completion: 95% completed the PCAT with a mean score of 67%. More than half scored at the competent level, and 7% scored at the expert level. No significant differences were detected among different faculties.

Group completion: 9 out of 11 groups fully completed the PCAT with a mean of 82%. Four (44%) groups scored at the expert level, while the rest scored at the competent level. The PCAT scores were significantly higher among those who completed the PCAT as a team compared to those who completed it individually.

**Discussion/Conclusions**: These results suggest that the interprofessional IPC program achieves its goals of imparting pain competences to prelicensure students. The results also confirmed the validity of the use of the PCAT for different professions. Future inquiry is required to better understand the mechanisms behind student learning in interprofessional pain education.

**Facilitators to successful online continuing professional education in interprofessional chronic pain management**

Lisa Jasper^a^, Kim Dao^b^ and Bernadette Martin^a^

^a^University of Alberta; ^b^Tufts University

**Introduction/Aim**: One in five Canadians live with chronic pain, and our current healthcare system is ill-equipped to meet their needs. Increasing access to quality continuing professional education (CPE) in interprofessional chronic pain management is essential to provide health providers with the necessary skills and knowledge. The aim of this study was to explore factors leading to enrolment and completion of an online Certificate in Pain Management.

**Methods**: Surveys were sent via electronic mail to registrants of the Certificate in Pain Management (Faculty of Rehabilitation Medicine, University of Alberta) between 2010 and 2021 to gather information on demographics and facilitators to enrolment and completion of the certificate.

**Results**: Twenty-nine respondents (12 male/15 female/1 other) were from 11 different health professions, five provinces/countries, both public and private practice settings, and both rural and urban residencies.

Respondents stated the five most important facilitators to enrolment were topics/content, content provided at a graduate level, certificate offered by recognized post-secondary institutions, online format, and asynchronous/self-paced learning format. When asked about strategies that facilitated successful completion of coursework, the following factors were identified (% respondents): decreasing personal time (82.7%), decreasing family time (58.6%), rearranging work schedule (24.1%), financial support from employer (17.2%), taking time-off from work (10.3%), and financial support from others (6.9%).

**Discussion/Conclusions**: Providing online, asynchronous, graduate level CPE in interprofessional chronic pain management encourages continued learning for healthcare professionals to help address the significant challenges in management of people with chronic pain.

**Impact and reach of online continuing professional education in interprofessional chronic pain management**

Kim Dao^a^, Lisa Jasper^b^ and Bernadette Martin^b^

^a^Tufts University; ^b^University of Alberta

**Introduction/Aim**: The chronic pain epidemic is growing within Canada, and the healthcare system is unable to effectively meet needs. Health professionals need access to quality learning opportunities to improve skills and interprofessional practice. Online continuing professional education (CPE) has the potential to meet this need. This study aims to investigate the impact and reach of an online CPE certificate on knowledge and care.

**Methods**: A survey was sent to students in the Certificate in Pain Management offered by the University of Alberta to gather information on demographics, opportunities for interprofessional development, and impact of learning.

**Results**: Twenty-nine individuals completed the survey, representing four Canadian provinces and the United States, and eleven professional designations. By population census group, 65.52% lived in metropolitan, 20.69% in urban core, and 13.79% in urban fringe or rural centers.

Over 75% respondents indicated that they strongly or somewhat agreed with statements that the CPE improved clinical expertise, confidence, knowledge to improve care, ability to share knowledge, and integration of interprofessional care. A range of 73.08% - 89.66% of respondents indicated that they strongly or somewhat agreed that the CPE experience increased interprofessional knowledge of practice roles, team functioning, communication, conflict resolution, patient-centered care, and collaborative leadership.

**Discussion/Conclusions**: Online CPE allowed access to effective higher-learning for individuals across varied geographical locations and population centers, indicating access to the opportunity regardless of residence. Demographic data reflect its interdisciplinary reach, improving interprofessional practice and addressing workforce shortages in chronic pain management.

**Knowledge and beliefs of the medical use of cannabis among nurses in the acute care setting: Understanding current needs and exploring knowledge enhancement strategies**

Salima Ladak^a^, Arlene Buzon-Tan^b^, Diana Tamir^c^ and Hance Clarke^d^

^a^University Health Network, Toronto General Hospital, University of Toronto Lawrence S. Bloomberg Faculty of Nursing; ^b^University Health Network, Toronto Western Hospital; ^c^University of Toronto, University Health Network, Toronto General Hospital and; ^d^University Health Network, University of Toronto

**Introduction/Aim**: The primary aim was to understand nurses’ knowledge of medical cannabis use in the post-operative setting, and their beliefs regarding medical cannabis. The secondary aim was to understand factors influencing these knowledge levels and beliefs. This poster will illustrate knowledge gaps and describe RNs’ recommendations for future education.

**Methods**: A prospective observational study of surgical nurses (n=230; aged 20-70 years) in a multisite academic health science centre was conducted. Main outcome measures were composite knowledge and belief scores, and their predictors.

**Results**: Desired knowledge across all topics pertaining to medical cannabis use, such as therapeutic indications, potential risks, mechanism of action, routes of administration, and dosing strategies were higher than current knowledge; all paired t-test p-values < 0.001. Nurses’ current levels of knowledge were rated lowest in the areas pertaining to Health Canada’s Marijuana for Medical Access Regulations, dosing and creating effective treatment plans.

Generally, nurses provided neutral responses to whether (1) RNs would be comfortable in discussing cannabis for medical use with their patients, (2) protection from legal liability would improve this level of comfort, (3) they believed patients could die from cannabis overuse, (4) cannabis can be used to treat acute pain, and (5) additional education would improve their confidence to have discussions with patients or treat patients already using medical cannabis.

**Discussion/Conclusions**: Gaps identified RNs current versus desired knowledge levels related to the use of medical cannabis, and beliefs about the effects of cannabis. Future educational interventions should address these gaps to support RNs practice and their education of patients.

**Evolving project ECHO: A virtual education model for interprofessional core competencies in paediatric pain**

Chitra Lalloo^a^, Vina Mohabir^a^, Fiona Campbell^a^, Naiyi Sun^a^, Sara Klein^b^, Jennifer Tyrrell^a^, Giulia Mesaroli^a^ and Jennifer Stinson^a^

^a^The Hospital for Sick Children; ^b^Department of Rehabilitation

**Introduction/Aim**: Paediatric Project ECHO for Pain is a virtual education program designed to empower interprofessional healthcare providers (HCPs) to locally manage children with pain. The program has delivered ~6,000 education hours since 2017. Unique from other Canadian ECHO programs, Paediatric Project ECHO offers foundational Core Competency pain education (https://sickkids.echoontario.ca/courses/pain). This study sought to evaluate model implementation from the perspective of HCP learners.

**Methods**: The Core Competency model (eLearning course and live debrief) underwent a pilot implementation between October and December 2021. A convenience HCP sample was recruited from the program registrant database. Each learner was given authenticated eLearning access, including interactive cases, quizzes, and resources, and was invited to a moderated debrief. REDCap surveys assessed usability, clinical relevance, changes in knowledge and/or self-efficacy, and anticipated applications. The study received local ethics approval (#1000057321).

**Results**: N=18 interprofessional HCPs from across Ontario were enrolled into the study. Baseline levels of self-reported pain knowledge were: beginner (39%), moderate (56%), or advanced (5%). Most learners (79%) completed > 75% of the eLearning course and 53% attended the debrief. Usability was high (94% characterized the system as easy to use) and all learners described the patient cases as clinically realistic. The model was associated with knowledge and self-efficacy improvements (100% of topics; 98% of skills). Anticipated Core Competency applications included: clinical refresher, support of patient assessment, and team orientation.

**Discussion/Conclusions**: Paediatric Project ECHO For Pain has innovated the ECHO model by integrating Core Competency learning. This advancement has demonstrated value for interprofessional HCPs who manage children with pain in the community.

**Building a better curriculum: Comprehensive education for healthcare professionals on opioids for pediatric pain management**

Chad Larabie^a^, Cynthia Nguyen^a^, Raad Fadaak^b^, Louise Tunnah^b^, Kathryn Birnie^c^, Jennifer Stinson^a^ and Fiona Campbell^a^

^a^SickKids; ^b^Solutions for Kids in Pain; ^c^University of Calgary, Solutions for Kids in Pain

**Introduction/Aim**: The opioid crisis is affecting communities worldwide, making it crucial for healthcare professionals to have the necessary knowledge and tools to make informed decisions regarding the safe and effective use of opioids for pain management in children. The Online Pediatric Pain Curriculum (OPPC) was developed to educate pre-licensure students and entry level healthcare professionals about pediatric pain. However, critical and evidence-based information about opioids is lacking from the module’s content. Thus, two new educational modules are being developed to support the safe, equitable, and effective use of opioids in children for both acute and chronic pain using a co-design approach.

**Methods**: These modules are being developed by the SickKids Pain Centre with Solutions for Kids in Pain (SKIP), a national mobilization network. The goal is to have modules that can be completed in 25 minutes or less and provide comprehensive knowledge to help address gaps for effective, safe, and equitable prescribing and use of opioids for pain in children and youth.

**Results**: We will describe a patient-centered approach that emphasizes the importance of involving patient partners and caregivers in co-creation of these modules with healthcare professionals. Module authors include youth/caregivers living with pain, and interprofessional experts, who will use an equity-based lens, ensuring that the information provided is culturally responsive and inclusive. The opioid modules will cover the benefits, risks, and harms of using opioids for pediatric pain, as well as important principles of pain assessment and treatment. They will use different clinical examples and showcase various treatment options. Additional experts will review both modules to ensure accuracy and alignment with best practices.

**Discussion/Conclusions**: These modules can improve pain management for children and are an important role in addressing pain as an upstream factor in the opioid crisis. The comprehensive, culturally responsive, and patient-centered approach emphasized in development of these modules will support healthcare professionals in providing equitable care to all children experiencing pain.

**From insights to action: The importance of evaluating an online interprofessional pediatric pain curriculum**

Chad Larabie^a^, Cynthia Nguyen^a^, Raad Fadaak^b^, Louise Tunnah^b^, Kathryn Birnie^c^, Chitra Lalloo^a^, Vina Mohabir^a^, Fiona Campbell^a^ and Jennifer Stinson^d^

^a^SickKids; ^b^Solutions for Kids in Pain; ^c^University of Calgary; ^d^Sickkids

**Introduction/Aim**: The Online Pediatric Pain Curriculum (OPPC) is a well-established, evidence-based, interprofessional educational program designed to enhance the knowledge of healthcare professionals in the assessment and management of pediatric pain. Developed based on the core educational curriculum of the International Association for the Study of Pain (IASP), the OPPC aims to equip pre-licensure students and entry-level healthcare professionals with the necessary competencies to manage pediatric pain more effectively. Here, we provide an overview of the usage and evaluation of the OPPC, with a focus on identifying the challenges faced by the curriculum and outlining the measures that will be taken to address them.

**Methods**: Website analytics, course metrics, surveys and post-course evaluations are used to analyze the OPPC modules.

**Results**: Since January 1, 2022, the OPPC has received 1,882 course completions with most participants being Nurses accessing the modules via desktop (95.3%) from over 10 countries, including low-to-middle income countries. Notably, Twitter was the largest external source of traffic. While the modules had an impressive 77% completion rate with an average completion time of 24 minutes, 6% of users from 359 post-course evaluations indicated a need for more interactivity and animation to enhance engagement.

To further improve completion rates, engagement, and global reach, we are undertaking several measures. These include updating the website and reducing the number of clicks required to access the modules, launching large-scale marketing campaigns to reach more countries, and partnering with people with lived experience to create interactive case studies.

**Discussion/Conclusions**: The OPPC is a valuable educational resource for healthcare professionals looking to improve their skills and knowledge in pediatric pain assessment and management. To address some of the challenges identified, the OPPC will be implementing measures to improve completion rates, engagement, and global reach. Maintaining a comprehensive evaluation framework and analysis will be critical in assessing the effectiveness and impact of the OPPC in years to come, so that the program continues to meet the needs of healthcare professionals.

**Readability and quality of online self-management educational resources for persons with knee osteoarthritis: An environmental scan**

Tarin Moni, Tara Packham, Lisa Carlesso and Luciana Macedo

McMaster University

**Introduction/Aim**: Increasing global disease burden of knee osteoarthritis (KOA) warrants effective treatments and management strategies. Self-management is often recommended for persons with KOA through online educational resources (ERs), however accessibility of these online resources may be variable. We aimed to identify and assess the readability and quality of online KOA self-management ERs.

**Methods**: Google searches using terms related to “knee osteoarthritis,” “educational resources,” and “self-management” were performed on private browsers. Flesch-Kincaid Reading Ease (FKRE) and Grade Level (FKGL) measured readability. Patient Educational Materials Assessment Tool (PEMAT) measured quality through understandability and actionability scores. ANOVA was performed to compare mean readability and quality between website origins. Pearson correlations tested the relationship between FKGL and understandability.

**Results**: Of 36 included ERs, content covered exercise (31, 86%), assistive devices (22, 61%), and weight loss (21, 58%). Website origins included government (20, 56%), academic (8, 22%), private (7, 19%), and commercial (1, 3%). Mean FKRE and FRGL were 53 ± 11.8 and 8.8 ± 1.8, respectively. Overall understandability and actionability were also low, (mean 62 ± 15.8 and 34 ± 28.3, respectively). No significant differences in mean readability and quality were seen between website origins. A moderate negative correlation was seen with FKGL and understandability (r=-0.35, p=0.03).

**Discussion/Conclusions**: Most online self-management ERs for persons with KOA exceed the recommended 6-8th grade level for patient education materials. ERs also show variable understandability and low actionability, making it difficult for persons with KOA to access and benefit from the information. Literacy levels must be a priority when developing client-focused ERs.

**Choosing wisely: Opioid wisely quality improvement initiative at sickkids**

Petra Hroch Tiessen^a^, Conor McDonnell^a^, Maha Al Mandhari^b^, Monica Caldeira-Kulbakas^c^, Suja Sri Satgunarajah^d^, Carmina Santos^e^, Anya Nair^c^, Jacqueline Hanley^c^, Ashley Harvey^c^, Kathryn A. Birnie^f^, Jennifer Stinson^a^ and Fiona Campbell^a^

^a^The Hospital for Sick Children, University of Toronto; ^b^Interdepartmental Division of Critical Care Medicine, University of Toronto; ^c^The Hospital for Sick Children; ^d^University of Ottawa; ^e^Toronto Metropolitan University; ^f^Alberta Children’s Hospital, University of Calgary

**Introduction/Aim**: This Quality Improvement (QI) initiative aims to: (1) mobilize evidence-based guidelines and standards for opioid prescribing for acute pain through a Choosing Wisely: Opioid Wisely recommendation and (2) develop, implement, and evaluate an education protocol aimed at prescribers and patients/caregivers to reduce harm and improve pain management. Our SickKids recommendation: Don’t routinely discharge children with acute pain on opioid analgesia for more than 3 days.

**Methods**: An audit of surgical populations at SickKids showed over-prescribing of opioids at discharge in children undergoing cleft palate repair. Focusing on this population, we undertook (1) an audit identifying baseline opioid prescribing practices, (2) an audit of existing educational materials, (3) qualitative interviews with prescribers and patients/caregivers to identify knowledge gaps, and (4) development of prescriber and patient/caregiver education packages to address these gaps.

**Results**: We found that knowledge gaps existed about (1) pain expectations, the importance of treating pain, and pain assessment after surgery (2) non-opioid and opioid pharmacotherapy (3) non-pharmacological pain management strategies, and (4) safe use, storage, and disposal of opioids. We also found how prescribers and patients/caregivers would like to receive education regarding opioids and pain management. Implementation and evaluation of the education protocol is underway, with ongoing monthly outcome, process, and balancing measures, feedback to/from stakeholders, continuous data analysis and iterative improvements.

**Discussion/Conclusions**: Based on our work at SickKids, we are working with Solutions for Kids in Pain (SKIP) supported by Health Canada’s Substance Use and Addictions Program (SUAP) Grant to develop and scale opioid-prescribing packages nationwide.

**Solutions-focused story telling: Challenging pain- and drug-related stigma and marginalization with graphic medicine**

Susan Tupper^a^, Erin Beckwell^b^, Alexandria Pavelich^c^, Kayla Arisman^c^, Dayna Fesciuc^d^, Jean Coe^a^, Michael Macfadden^a^, Cheyanne Desnomie^e^, Barbara Fornssler^c^ and Pamela Downe^c^

^a^Saskatchewan Health Authority; ^b^University of Regina; ^c^University of Saskatchewan; ^d^Person with lived experience; ^e^First Nations University of Canada

**Introduction/Aim**: Graphic medicine (i.e. educational comics) can improve patient and provider knowledge, change health behaviors, and improve provider empathy and care delivery. The goal of this research is to develop graphic medicine stories about chronic pain management for use in healthcare provider or patient education to challenge pain- and drug-related stigma and marginalization in healthcare settings and improve person-centered care.

**Methods**: Narrative interviews were conducted with n=13 people living with chronic pain residing in Saskatoon’s downtown core neighborhoods and n=5 healthcare providers. Thematic narrative analysis was conducted on interview transcripts. Five story-board meetings were held with the research team, two Indigenous Knowledge Keepers, and the graphic design team to co-design a series of brief graphic medicine stories.

**Results**: Participants living with pain primarily identified as women (n=10; average age 44 years), First Nations or Metis (n=11), and repored severe pain (n=7) for an average of 12.8 years. HCP participants were nurses and physicians from 5 different healthcare settings. Story narratives for people living with pain centered on themes of feeling invisible, pain dismissal, racism, and behavior escalation to be seen. Healthcare provider narratives focused on feeling helpless to change the system and recognition of the complexity of care. Both participant groups identified simple strategies for person-centered care including connecting as a person and open communication.

**Discussion/Conclusions**: Graphic medicine provides a novel educational opportunity to raise awareness about ways to deliver compassionate care in with patients living with pain in everyday healthcare encounters. Further research is needed to collect patient and provider perspectives on the graphic medicine stories and to examine the impact of these stories on provider empathy and patient care.

**Let’s talk about pain! Shared picture-book reading as an opportunity for children to learn about pain and injury**

Sarah Wallwork^a^, Melanie Noel^b^, Sue Nichols^a^, Abbie Jordan^c^ and Lorimer Moseley^a^

^a^University of South Australia; ^b^University of Calgary; ^c^University of Bath

**Introduction/Aim**: Children’s fundamental beliefs about pain and injury are shaped from early childhood and can be guided by social, cultural, and environmental contexts. In this study, we explored whether shared picture-book reading between caregivers/parents and their children could be a potential opportunity for children to learn about pain and injury.

**Methods**: Twenty parent/caregiver-child groups (target child age: 3-6 years) were recruited through local libraries in Adelaide, Australia. Participation involved undertaking shared picture-book reading of books that had pain and/or injury depicted in the narrative. Participants were told that the aims of the study were to better understand the conversations and interactions that occur during shared picture-book reading and were not privy to the pain/injury specific aims of the study. Parents were asked to read the books with their child[ren] as they typically would. Participants were debriefed about the undisclosed aims of the study (pain/injury content) on completion of the reading. Video recordings of the readings were transcribed, and picture books underwent a structural narrative analysis. Transcriptions and the book analysis were combined for an overall reflexive thematic analysis with an inductive analysis approach.

**Results**: Eight male and twelve female caregivers and their children (10 male, 17 female; aged 2-7 years) participated in the study. Preliminary analyses indicate that parents/caregivers and children both initiate conversations and interactions that relate to pain and injury during shared picture-book reading.

**Discussion/Conclusions**: Shared picture-book reading between parents/caregivers and young children is an opportunity for children to learn about pain and injury.

**ECHO ontario chronic pain and opioid stewardship: Clinicians’ goals, experiences, and referral patterns**

Q. Jane Zhao^a^, Leslie Carlin^b^, John Flannery^b^, Paul Taenzer^c^ and Andrea D. Furlan^b^

^a^ECHO at UHN, Toronto Rehabilitation Institute, University Health Network; ^b^University of Toronto; ^c^Queen’s University

**Introduction/Aim**: Extension for Community Healthcare Outcomes Ontario Chronic Pain and Opioid Stewardship (‘ECHO’) is a telehealth education intervention that bridges specialists in academic centres to health care clinicians across Ontario and nationally. In doing so, ECHO disseminates knowledge regarding chronic pain and opioid management, increases clinicians’ self-efficacy, and fosters a community of practice. This study evaluated clinicians’ goals when joining ECHO and ECHO impact on clinicians’ experiences managing chronic pain patients and practice characteristics.

**Methods**: An online questionnaire was administered pre-post ECHO attendance. The questionnaire assessed: 1. goals (open-text response); 2. experiences managing chronic pain patients (8 items); 3. practice characteristics. Goals were analyzed using the qualitative descriptive approach. Experiences were analyzed using the Wilcoxon signed-rank test after testing for normality. Descriptive statistics were summarized for practice characteristics.

**Results**: From 2018 to 2023, 389 clinicians participated in this study. 111 (28.6%) were physicians and 84 (21.6%) were nurse practitioners. Clinicians varied in goals in attending ECHO: increase knowledge and confidence, develop a team approach to managing chronic pain, and develop a support network. Of the eight items measuring clinicians’ experiences managing chrosssnic pain patients, two items (“I feel invigorated” and “I feel a sense of accomplishment”) increased significantly (p=0.10 and p=0.035 respectively). After attending ECHO, 34.4% of clinicians referred the same, 22.2% referred less, and 10% referred more than before; 33.3% answered not applicable.

**Discussion/Conclusions**: ECHO demonstrated positive impact in building clinicians’ capacity and their experiences managing chronic pain patients. There were no significant changes in clinicians’ referral patterns.

**Treatment/management/pain programs Le traitement, la gestion ou les programmes de prise en charge de la douleurThe flavour of pain-relief: Tongue stimulation to reduce pain in poststroke patients**

Maureen Ahiatsi^a^, Marie-Hélène Milot^b^, Eléonor Riesco^c^, Marie-Claude Girard^d^ and Guillaume Léonard^e^

^a^Université de Sherbrooke, Faculté de médecine et des sciences de la santé; ^b^Université de Sherbrooke, Centre de recherche sur le vieillissement du CIUSSS de l’Estrie-CHUS; ^c^Université de Sherbrooke, Faculté des sciences de l’activité physique et Centre de recherche sur le vieillissement du CIUSSS de l’Estrie-CHUS; ^d^Centre de recherche sur le vieillissement du CIUSSS de l’Estrie-CHUS; ^e^Université de Sherbrooke, Centre de recherche sur le vieillissement du CIUSSS de l’Estrie-CHUS

**Introduction/Aim**: Stroke survivors often experience pain in the affected upper limb (UL), impacting their function and quality of life. To minimise the impact of these deleterious effects, non-invasive neurostimulation techniques, such as cranial nerve non-invasive neuromodulation (CN-NINM), is increasingly studied and used in rehabilitation. By stimulating the tongue, CN-NINM depolarizes cranial nerves, creating direct neural impulses to the brainstem in areas known to play a key role in pain modulation. The objective of this project was to investigate the feasibility and the effectiveness of CN-NINM on pain in the affected UL in individuals with chronic poststroke (> 6months).

**Methods**: In this feasibility pilot pre/post intervention study, 12 adults in the chronic phase of a stroke are recruited. A tonic experimental thermal pain stimulus is used to measure the impact of CN-NINM on experimental pain (tonic heat pain test applied over the participant’s forearm) before and after a single 20-minute application of CN-NINM. Feasibility measures include recruitment rates, adherence to the intervention and adverse effects. Inferential statistics will be used to explore the immediate effect of the CN-NINM intervention on pain perception.

**Results**: All 10 participants recruited to date completed the intervention. No adverse effects were reported. The recruitment rate was 100%. Although some participants experienced a reduction in pain following the intervention, the hypoalgesic effects of CN-NINM remain somewhat marginal and do not reach the threshold of clinical significance.

**Discussion/Conclusions**: Unfortunately, the initial findings looking into the effect of CN-NINM on pain suggest that the intervention has no direct immediate impact on pain perception.

**Do all patients benefit from the soothing properties of comfort talk to reduce symptom burden during outpatient chemotherapy? A multimethod secondary analysis**

Caroline Arbour^a^, Danny Hjeij^b^, Alexandra Lapierre^a^, Karine Bilodeau^c^, Pierre Rainville^d^ and David Ogez^e^

^a^Faculty of Nursing, Université de Montréal, Centre de recherche de l’Hôpital du Sacré-Cœur de Montréal, CIUSSS du Nord-de-l’Île-de-Montréal, Montreal, Quebec, Canada; ^b^Centre de recherche de l’Hôpital du Sacré-Cœur de Montréal, CIUSSS du Nord-de-l’Île-de-Montréal, Montreal, Quebec, Canada; ^c^Faculty of Nursing, Université de Montréal, Centre de Recherche de l’Hôpital de Maisonneuve-Rosemont (CRHMR), CIUSSS de l’Est-de-l’Île-de-Montréal, Montreal, Quebec, Canada; ^d^Faculty of Dental Medicine, Department of stomatology, Centre de recherche de l’Institut universitaire de gériatrie de Montréal, CIUSSS Centre-sud-de-l’Île de Montréal, Montreal, Quebec, Canada; ^e^Faculty of Medicine, Department of Anesthesia, Université de Montréal, Centre de Recherche de l’Hôpital de Maisonneuve-Rosemont (CRHMR), CIUSSS de l’Est-de-l’Île-de-Montréal, Montreal, Quebec, Canada

**Introduction/Aim**: Patients undergoing outpatient chemotherapy often report breakthrough pain resisting standard pharmacotherapy, which may lead to treatment discontinuation. Our work on comfort talk (CT), a brief intervention inspired by the language principles of clinical hypnosis, support its feasibility in helping prevent pain outbreaks during chemotherapy perfusions. Still, the profile of patients most likely to benefit from CT is poorly understood. The study aimed to identify preliminary CT assignment criteria to reduce pain and other disturbing symptoms during chemotherapy perfusions.

**Methods**: A secondary analysis of data from two previous CT studies (one quantitative, one qualitative) was conducted. A total of 24 patients were exposed to a nurse-led and partially scripted CT intervention during a planned outpatient appointment and completed the Edmonton Symptom Assessment Scale before and after chemotherapy perfusion. Qualitative content analysis was used on existing data from six interviews with oncology nurses who participated in CT administration during outpatient chemotherapy.

**Results**: Older patients (> 60 years) with metastatic cancer, shorter treatment duration (< 1 hour), and less social support were more likely to report symptom reduction following CT during chemotherapy perfusion. Conversely, nurses’ interviews suggest that CT could prevent pain outbursts mainly in patients: 1) with higher anxiety or fear of needles, 2) undergoing long chemotherapy treatment (> 1 hour), and 3) experiencing late side effects at home.

**Discussion/Conclusions**: A gap exists between oncology patients’ and nurses’ perceptions of CT utility for preventing pain and other disruptive symptoms during outpatient chemotherapy, with several implications for education and future research.

**Outcomes of spinal cord stimulation: Real-world data from participants with peripheral neuropathic pain syndromes**

Sireedhorn Assavanop, Siu Min Lim, Jamal Kara, Pranab Kumar, Suneil Kalia, Mojgan Hodaie, Victoria Bains and Anuj Bhatia

Neuromodulation for Pain program, Toronto Western Hospital, University Health Network, University of Toronto

**Introduction/Aim**: Peripheral neuropathic pain (PNP) is challenging to treat. Spinal cord stimulation (SCS) has been used to relieve pain in participants with PNP with variable success rates. We present data from our centre to evaluate the outcomes of SCS in participants with PNP. We aimed to identify potential predictors for successful SCS trial and implant.

**Methods**: Fifty-six participants who had a diagnosis of PNP received SCS trials. 28 participants who had successful SCS trials underwent SCS implants between July 1, 2017 to February 28, 2022. Successful SCS trial defined as 50% or more, and successful SCS implant defined as 30% or more reduction in NRS scores compared to baseline.

**Results**: The Pain NRS score (2.8 vs 6.0, p < 0.001), GAD-7 score (4.1 vs 9.7, p=0.002), PDI score (23.4 vs 41.0, p=0.008), PSQ-3 score (95.0 vs 183.3, p=0.039), NPSI score (23.3 vs 61.3, p < 0.001), and PCS score (9.5 vs 26, p=0.002) were significantly lower in the successful SCS implant compared to unsuccessful group. Participants’ Global Impression of Change scale showed remarkable satisfactory outcomes after SCS implant (same or worse/improved: 1/18 in successful vs. 4/5 in unsuccessful implant cohort, p=0.026). Adverse outcomes included reimplantation following infection (3.6%), explantation due to lack of efficacy (1.8%), and lead migration requiring revision (1.8%).

**Discussion/Conclusions**: Success rate of SCS implant in participants with PNP at our centre was 67.9% with significant improvement in mental and physical health at 6-12 months. Potential factors associated with unsuccessful trial or implant in our study were history of anxiety and depression, smoking, higher PSQ-3 score.

**Clinician knowledge, attitude, beliefs and behavior with regards to managing the psychosocial aspects of pain and disability in hand therapy practice: A qualitative study**

Folarin Babatunde^a^, Joy MacDermid^a^, Luciana Macedo^b^, Ruby Grewal^a^ and Mike Szekeres^a^

^a^Western University; ^b^McMaster University

**Introduction/Aim**: Hand and upper limb injuries are very common in orthopaedic settings with substantial burden to the individual and society. Psychosocial factors account for prolonged recovery and long-term disability in these injuries. Psychologically informed hand therapy practice can enhance treatment outcomes through evidence-based risk identification, clinical decision making, delivery of supplemental interventions and mental health service referral. The objective of this study was to gain an understanding of the knowledge, attitudes, beliefs and practice behavior of physical therapists and occupational therapists in hand therapy with regards to screening, assessing, and managing psychosocial issues in patients with hand injuries.

**Methods**: Qualitative descriptive study using individual audio-taped semi-structured interviews. Purposive sampling of clinicians practicing hand therapy in private and public healthcare settings based in Ontario, Canada. Discussions were guided by an interview guide based on the Theoretical Domains Framework. Transcriptions were thematically analyzed.

**Results**: A total of eighteen physical therapists and occupational therapists attended the interviews. Four major themes with twelve subthemes were identified, namely: (a) Taking responsibility (Awareness, Opportunity, Confidence, Apprehension) (b) Knowledge base (Foundational, Clinical), (c) Skill and Resiliency (Communication, Stigmatization, Experience) and, (d) Leveraging resources (Patient, Health system, Socio-environmental).

**Discussion/Conclusions**: Occupational therapists demonstared more confidence with managing psychosocial issues. However, varied foundational and clinical knowledge, organizational climate and leadership, available support and therapist skill and experience account for ongoing challenges with psychosocial adjustment in hand therapy. Physical therapists and Occupational therapists in hand therapy would benefit from additional training, tools and guidelines to support patients with psychosocial issues.

**Self-treatment of chronic low back pain based on a rapid and objective sacroiliac asymmetry test: A pilot study**

Hélène Bertrand^a^, K. Dean Reeves^b^, Rajneet Mattu^a^, Remerlita Garcia^c^, Mahir Mohammed^d^, Ellen Wiebe^a^ and An-Lin Cheng^e^

^a^University of British Columbia; ^b^Independent Researcher; ^c^No affiliation; ^d^No institution; ^e^UMKC

**Introduction/Aim**: Low back pain (LBP) is common, costly, and disabling. This study assesses a novel and simple LBP evaluation method and its merit in guiding the direction of a self-treatment exercise.

**Methods**: Randomized open-label intention to treat study. Consecutive patients with LBP ≥ three months and pain ≥ 5/10 were evaluated in a Vancouver clinic with the sacroiliac forward flexion test (SIFFT) by comparing the height of posterior superior iliac spines using a level. Those with asymmetry ≥ 5 mm were offered participation. The assistant, who generated and encrypted the randomization, assigned participants: group 1 learned a two-minute, SIFFT-derived, sacroiliac-leveling exercise (SIFFT-E) as needed for LBP relief; group 2 used a pelvic stabilization belt as needed to prevent LBP, and group 3 continued the usual care. After one month, all participants used SIFFT-E and belt as needed for one month. Our primary outcome measure was the Oswestry disability index (ODI) (decrease) from baseline to one and two months.

**Results**: Of 72 LBP patients, 62(86%) had ≥5mm asymmetry. From zero to one month, the 21 (one dropout) SIFFT-E participants outperformed the 20 usual care participants for ODI improvement (12.5 ± 14.8 vs. -3.4 ± 14.9 points; mean difference 15.9 [CI 6.7-25.0]; P=0.002. Number needed to treat 3.0 for ODI improvement ≥11). Belt use results were intermediate. After all participants used exercise and belt as needed, ODI improvements were clinically significant (12.0 ± 18.4 points). Five (8%) exercise and 12(19%) belt wearers experienced mild side effects.

**Discussion/Conclusions**: Sacroiliac asymmetry appears to be frequent. SIFFT may be clinically useful as an evaluation tool for prescribing a simple self-directed corrective exercise (SIFFT-E) as seen by clinically significant improvements in function.

**The experience of moment-to-moment opioid withdrawal symptoms and opioid craving in chronic non-cancer pain patients**

Alice Bruneau^a^, Sarah Petkau^a^, Alexandra Gavrilescu^a^, Yami-Louise Djoudi^a^, Juliet Ware^b^, Amanda Sirois^a^, Jordi Perez^c^ and Marc O. Martel^a^

^a^McGill University; ^b^University of British Columbia; ^c^Alan Edwards Pain Management Unit, McGill University Health Centre

**Introduction/Aim**: Chronic non-cancer pain (CNCP) patients who are using opioids may experience symptoms of opioid withdrawal and opioid craving. However, little is known on the factors that contribute to momentary fluctuations of opioid withdrawal symptoms and opioid craving in these patients. The first objective of this study was to examine the association between opioid withdrawal symptoms and opioid craving among CNCP patients on opioid therapy. We also examined the psychological factors that may contribute to opioid withdrawal symptoms and craving.

**Methods**: In this ecological momentary assessment (EMA) study, patients (n=44) provided momentary reports of opioid withdrawal symptoms and opioid craving throughout the day, when randomly prompted, for 10 consecutive days. At the same moments, patients also provided reports of pain intensity, negative affect, and pain catastrophizing.

**Results**: A multilevel regression analysis revealed that higher momentary levels of opioid withdrawal symptoms were associated with higher craving (p < .001). Higher momentary levels of pain, negative affect, and catastrophizing were also associated with heightened opioid withdrawal symptoms and craving (all p’s < .05). Results from a multivariable regression analysis indicated that opioid withdrawal symptoms did not significantly contribute to opioid craving after accounting for patients’ moment-to-moment levels of pain intensity, negative affect, and catastrophizing.

**Discussion/Conclusions**: Results from our study advance our understanding of factors contributing to daily opioid withdrawal symptoms and opioid craving among chronic pain patients prescribed opioid therapy.

**Predictors of fatal and non-fatal overdose following prescription of opioids for chronic pain: A systematic review and meta-analysis of observational studies**

Li Wang^a^, Patrick Jiho Hong^b^, Wenjun Jiang^a^, Yasir Rehman^a^, Brian Younho Hong^b^, Rachel Couban^a^, Chunming Wang^c^, David Juurlink^b^ and Jason Busse^a^

^a^McMaster University; ^b^University of Toronto; ^c^Guangdong Academy of Sciences

**Introduction/Aim**: Long-term opioid use is associated with serious harms, including nonfatal and fatal overdose; however, the factors associated with opioid overdose when prescribed for chronic pain are unclear. We conducted a systematic review and meta-analysis to explore predictors of opioid overdose after prescription for chronic pain.

**Methods**: We searched MEDLINE, EMBASE, CINAHL, PsycINFO and Web of Science from inception to October 2022, for observational studies that explored predictors of opioid overdose following opioid prescription for chronic pain. Paired reviewers independently extracted data, assessed risk of bias, and overall certainty of evidence. We performed random-effects meta-analyses for all factors reported by ≥2 studies.

**Results**: Twenty-seven studies (23,948,324 patients) reported the association of over 100 factors with opioid overdose after prescription for chronic pain. Moderate to high certainty evidence supported large associations with opioid overdose and a history of overdose (OR 4.83 [95% CI 3.34 to 6.99]), higher opioid dose (OR 2.77 [95% CI 2.09 to 3.68] for every 90-mg increment in morphine equivalent dose), transdermal fentanyl (OR 2.80 [95% CI 2.30 to 3.41]), long-acting formulations (OR 1.95 [95% CI 1.51 to 2.51]), current substance use disorder (OR 2.42 [95% CI 1.75 to 3.37]), any mental health diagnosis (OR 2.12 [95% CI 1.83 to 2.45]), depression (OR 2.17 [95% CI 1.50 to 3.12]), or pancreatitis (OR 2.00 [95% CI 1.52 to 2.64]). These factors were associated with absolute risks ranging from 2‰ (2 in 1000) to 5‰ for fatal and 4‰ to 10‰ for non-fatal overdose.

**Discussion/Conclusions**: Minimizing high-dose opioid prescribing, fentanyl, long-acting formulations, and opioid prescribing to chronic pain patients that have previously overdosed or present with a current substance use disorder, depression, other mental illness or pancreatitis may reduce risk of overdose.

**Inhaled medical cannabis for chronic pain: A systematic review and meta-analysis of randomized clinical trials**

Li Wang^a^, Vahid Ashoorion^a^, Fares Hayek^a^, Kevin Shao^a^, Cody Tran^a^, Wenjun Jiang^a^, Yiwei Chen^b^, Henry Kwon^c^, Rachel Couban^a^ and Jason Busse^a^

^a^McMaster University; ^b^Cornell University; ^c^Wayne State University

**Introduction/Aim**: Medical cannabis is consumed in inhaled forms (i.e., smoked or vaped) by most patients with chronic pain; however, its benefits and harms are uncertain. We aimed to conduct a systematic review and meta-analysis of randomized clinical trials (RCTs) to assess the effectiveness of inhaled forms of medical cannabis for chronic pain.

**Methods**: We searched MEDLINE, EMBASE, AMED, PsycInfo, CENTRAL, CINAHL PubMed, Web of Science, Cannabis-Med, Epistemonikos, and trial registries up to July 2022 to identify RCTs of inhaled medical cannabis for chronic pain. Paired reviewers independently assessed risk of bias and extracted data from eligible studies. We used random-effect models for all meta-analyses and the grading of recommendations assessment, development and evaluation (GRADE) system to assess the certainty of evidence.

**Results**: A total of 12 eligible trials with 395 adult patients living with chronic non-cancer pain compared inhaled medical cannabis, either as smoked cigarettes (7 RCTs) a smoke device (1 RCT) or vaporized (n=5 RCTs), vs. placebo. The median sample size among included trials was 33 patients (interquartile range [IQR] 26-39) and the median length of follow-up was 1.5 days (IQR 5 hours to 5 days).

Compared with placebo, inhaled medical cannabis probably improves short-term pain relief between 2.5 hours to 5 days with larger proportion of patients achieving at least 30% pain reduction: risk difference [RD] 20% (95%CI 11% to 30%) based on a relative risk [RR] 1.60 (95%CI 1.30 to 1.95, high certainty), and a weighted mean difference [WMD] of -0.70 cm on a 10 cm visual analogue scale [VAS] (95%CI -1.04 to -0.36 cm, moderate certainty). Inhaled medical cannabis did not improve physical, emotional, role functioning, or sleep quality (moderate certainty), but the follow-up time was likely too short to detect effects on these outcomes. Moderate certainty evidence shows that inhaled medical cannabis probably results in small increased risk of dizziness (RD 10%, 95%CI 3% to 25%), cough (RD 16%, 95%CI 3% to 44%), and euphoria (RD 12%, 95%CI 4% to 23%). Low certainty evidence shows that the short-term inhaled medical cannabis might not increase the risk of cognitive impairment, anxiety, drowsiness, shortness of breath, headache, paranoia, vomiting, nausea, tachycardia, hypotension, weakness, fatigue, bad taste, or dry mouth.

**Discussion/Conclusions**: Moderate to high certainty evidence shows that short-term use of non-inhaled medical cannabis results in a modest improvement in pain relief among patients who live with chronic noncancer pain, along with several transient adverse side effects, compared to placebo.

**Acupuncture for diabetic peripheral neuropathy: A systematic review and meta-analysis of randomized clinical trials**

Lei Lan, Li Wang, Rachel Couban, Behnam Sadeghirad and Jason Busse

McMaster University

**Introduction/Aim**: Diabetic peripheral neuropathy (DPN) affects up to half of all patients with diabetes mellitus. Acupuncture is a popular treatment for chronic pain, but its’ effectiveness for DPN is uncertain. We completed a systematic review and meta-analysis of randomized clinical trials (RCTs) to assess the effect of acupuncture for DPN.

**Methods**: We searched CENTRAL, CINAHL, EMBASE, MEDLINE, AMED, CNKI, VIP, and Wang Fang from inception to September 2022 for RCTs of acupuncture for DNP. Paired reviewers independently extracted data and assessed risk of bias. We used random-effect models for all meta-analyses and GRADE approach to assess the certainty of evidence.

**Results**: Eighteen RCTs including 1525 participants (32% female) were included. Moderate certainty evidence suggests that, compared with sham acupuncture or usual care, acupuncture is likely to reduce both pain (weighted mean difference [WMD] −1.85 cm [95%CI −2.96 to −0.74 cm] on a 10-cm scale; modeled risk difference for achieving the MID of 1cm, 49% [95% CI 22 to 62]), and severity of neurologic symptoms (WMD -0.79 [-1.46 to -0.12] on the 19-point Toronto clinical scoring system [TCSS]). Low certainty evidence suggested acupuncture may reduce pain compared with amitriptyline (WMD -1.01 cm [-1.26, -0.76 cm]) or pregabalin (WMD -1.96 cm [-2.68, -1.24cm]), and severity of neurologic symptoms compared with mecobalamin (WMD −1.72 [−2.55, -0.89]), lipoic acid and alprostadil (WMD −7.65 [−8.53, -6.78]), but may make no difference with α-LA and BPS (WMD −0.32 [−1.08, 0.44]).

**Discussion/Conclusions**: Acupuncture is likely to improve pain and neurologic symptoms severity associated with DPN compared with sham acupuncture or usual care; however, the evidnece for the comparative effectiveness of acupuncture vs. pharmacothertapy is only low certainty.

**Prevalence of return to work following acceptance for disability or sick absence benefits: A systematic review and meta-analysis of observational studies**

Li Wang^a^, Vahid Ashoorion^a^, Yaping Chang^a^, Ke Guo^b^, Dan Liu^c^, Sha Diao^c^, Henry Kwon^d^, Sara Ghazizadeh^e^, Zijun Li^b^, Alireza Malektojari^e^, Yanfei Li^b^, Cody Tran^a^, Sean Kennedy^f^, Katie Kennedy^f^, Mina Ma^b^, Minyan Yang^b^, Haitong Zhao^b^, Rachel Couban^a^ and Jason Busse^a^

^a^McMaster University; ^b^Lanzhou University; ^c^Sichuan University; ^d^Wayne State University; ^e^Hormozgan University of Medical Sciences; ^f^University of Toronto

**Introduction/Aim**: Work-related injuries and illnesses are important public health problems, which account for 4% of annual worldwide GDP (about $1.25 trillion), and costs are disproportionally incurred by claimants with delayed recovery. The prevalence of return to work (RTW) after receiving disability or other benefits is uncertain. We therefore conducted a systematic review and meta-analysis to establish the overall prevalence of return to work after receipt of disability benefits.

**Methods**: We searched MEDLINE, EMBASE, CINAHL, and PsycINFO from inception to March 2022, for observational studies reporting the rate of RTW or claim closure following receipt of disability or sick absence benefits. We used random-effects meta-analysis with Freeman-Tukey Double Arcsine transformation to estimate the overall prevelance of RTW, and logit transformation as a sensitivity analysis

**Results**: We included 181 cohort studies including 2,894,905 patients awarded disability or sick absence benefits. Among them, 42 studies (23%) were conducted in USA, 28 (17%) in Canada, 81 (45%) in Europe, 19 (10%) in Australia and New Zealand, and 11 (6%) in Asia; no studies enrolled participants from South America or Africa.

The prevalence of RTW or claim closure ranged from 19%-98%, median 71% (IQR 57-83 %); and the pooled prevalence was 70% (95%CI 67-73%). The sensitivity analysis using logit transformation showed similar results [72% (95%CI 67-75%)]. Meta-regression showed significant association between rate of RTW and length of follow-up (p < 0.001) with 3% increase in RTW for every year of follow-up (95%CI 1 to 4%). The rates of RTW or claim closure were 63% (95%CI 55-70%) within 6 months, 71% (95%CI 64-77%) between 6 months to 1 year, 69% (95%CI 65-73%) between 1 to 2 years, 71% (95%CI 60-81%) between 2 to 3 years, and 80% (95%CI 73-85%) at more than 3 years. No additional subgroup effects were found.

**Discussion/Conclusions**: According to our findings, for every10 patients receiving disability or sick absence benefits, 7 return to work on average. Individuals that have not resumed employment at 6-months after receiving disability benefits are likely to remain disabled at 2 years. Future studies should explore the predictors that were associated with prolonged recovery after receiving disability or sick absence benefits to identify high-risk population and modifiable factors for interventions.

**The association between initiating chiropractic care and continued prescription opioid use for chronic non-cancer pain: A sequential explanatory mixed-methods study**

Peter C. Emary^a^, Amy L. Brown^b^, Mark Oremus^c^, Lawrence Mbuagbaw^a^, Douglas F. Cameron^b^, Jenna DiDonato^d^ and Jason Busse^a^

^a^McMaster University; ^b^Private practice; ^c^University of Waterloo; ^d^D’Youville University

**Introduction/Aim**: We explored the association between receipt of chiropractic care and continued opioid use among adult patients with chronic pain attending an Ontario community health centre.

**Methods**: In this sequential explanatory mixed-methods study, we conducted a retrospective analysis of 210 patient records (January 1, 2014 to December 31, 2020) and completed interviews with 14 patients and nine general practitioners. We used generalized estimating equations, adjusted for patient demographics, co-morbidities, visit frequency, and calendar year to evaluate the association between receipt versus non-receipt of chiropractic services and continued opioid use over 12-month follow-up.

**Results**: There were lower rates of opioid fills (IRR=0.66; 95% CI, 0.52-0.83) and refills (IRR=0.27; 95% CI, 0.17-0.42) among patients who initiated chiropractic care (n=49) versus non-recipients (n=161). Although there were no differences in average daily opioid dose between patients who did and did not receive chiropractic care at inception of our cohort study, those patients who subsequently received chiropractic care were less likely to be prescribed higher-dose opioids (> 50 mg MED) compared to non-recipients at 3-months (OR=0.14; 95% CI, 0.04-0.47), 6-months (OR=0.14; 95% CI, 0.05-0.40), 9-months (OR=0.19; 95% CI, 0.07-0.57), and 12-months (OR=0.22; 95% CI, 0.08-0.62). Interviews suggested that patient self-efficacy, limited effectiveness of opioids for chronic pain, stigma regarding opioid use, and access to chiropractic treatment were influencing factors.

**Discussion/Conclusions**: We found a negative association between continued prescription opioid use and receipt of chiropractic care among patients with chronic pain.

**Intercostobrachial nerve preservation during breast cancer surgery to prevent chronic postoperative pain: A systematic review and meta-analysis of randomized clinical trials**

Zhaoxia Li^a^, Fan Wang^b^, Jason Busse^c^, Rachel Couban^c^ and Li Wang^c^

^a^Second Hospital of Gansu Province; ^b^Northwest Minzu University; ^c^McMaster University

**Introduction/Aim**: Damage to the intercostobrachial nerve (ICBN) during breast cancer surgery is associated with chronic postoperative pain, sensory disturbance, and impaired function. We aimed to assess the effects of the ICBN preservation vs. sacrifice in breast cancer surgery patients.

**Methods**: We searched MEDLINE, EMBASE, CENTRAL,China National Knowledge Infrastructure (CNKI) and Wanfang Datadase up to October 2022 for randomized clinical trials (RCTs) that assessed the effects of ICBN preservation vs. sacrifice in breast cancer surgery patients on chronic postoperative pain and sensory disturbance. We used random-effects meta-analysis to pool effects across trials, and the Grading of Recommendations, Assessment, Development and Evaluations (GRADE) approach to rate certainty of evidence.

**Results**: Thirty-six RCTs including 3768 breast cancer surgery patients were included. Moderate certainty evidence shows that, compared to ICBN sacrifice, preservation probably reduces the development of chronic postoperative pain at 90 days (relative risk [RR] 0.34, 95% CI 0.22 to 0.53; absolute risk reduction [ARR]14.8%, 95%CI 10.5 % to 17.4%), at 180 days (RR 0.25, 95% CI 0.18 to 0.34; ARR 29.1%, 95%CI 25.6 % to 31.8%) and at 360 days and greater (RR 0.26, 95% CI 0.17 to 0.38; ARR 13.2%, 95%CI 11.0% to 14.8%).

The impact of ICBN preservation vs. sacrifice on sensory disturbance will be analyzed and reported at the 2023 Canadian Pain Society conference.

**Discussion/Conclusions**: Preservation of the ICBN during breast cancer surgery is likely to reduce the risk of developing chronic postoperative pain.

**Effectiveness of usual medical care plus chiropractic care vs. usual medical care alone for low back pain: A systematic review of randomized clinical trials**

Andrea Darzi^a^, Behnam Sadeghirad^a^, Rana Charide^a^, Samer G. Karam^a^, Sophia Mangala^a^, Dale Alameddine^b^, Sophia Mangala^a^, Gordon H. Guyatt^a^ and Jason Busse^a^

^a^McMaster University; ^b^Yale University

**Introduction/Aim**: Low back pain (LBP) is a prevalent complaint for which many patients seek chiropractic care. We conducted a systematic review to explore the effectiveness of chiropractic care, when added to usual medical care, vs. medical care alone.

**Methods**: We searched Medline, AMED, EMBASE, CINAHL, the Cochrane Database of Systematic Reviews, Index to Chiropractic Literature, and Cochrane Library from inception to February 28, 2022. Studies eligible for review included pragmatic randomized controlled trials of chiropractic care plus usual medical care for low back pain compared to usual medical care alone. We used the GRADE approach to assess the certainty of evidence.

**Results**: We included 6 randomised trials which enrolled 2329 patients with variable durations of low back pain. Moderate certainty evidence showed that receiving chiropractic care vs usual medical care alone probably slightly decreases bothersomeness (weighted mean difference [WMD] −0.40cm [95% CI, −0.55 to −0.25] on a 5-point scale). Low certainty evidence suggests that augmenting usual medical care with chiropractic care, compared with usual care alone, may reduce pain (WMD −0.69cm [95% CI, −1.00 to −0.39] on a 10cm NRS scale for pain), improve physical function (WMD −1.01 cm [95% CI, −1.69 to −0.32] on the 24-point Roland Morris disability score for function), reduce of fear avoidance behaviour related to physical activity (WMD −2.5 cm [95% CI, −4.5 to -0.5] on the 0–24-point fear avoidance belief questionnaire physical activity subscale), and reduce medication use (odds ratio 0.76 [95% CI, 0.58 to 1.00]). Low- certainty evidence suggests little to no difference in fear avoidance behaviour related to work activities in those receiving chiropractic care compared to usual care alone (WMD −1.7 cm [95% CI, −4.5 to 1.1] on the 0–42-point fear avoidance belief questionnaire work subscale). No serious adverse events related to treatment were reported for either intervention.

**Discussion/Conclusions**: Compared with usual medical care, augmentation with chiropractic care probably decreases bothersomeness of low back pain, and may improve pain, physical functioning and fear avoidance behaviour related to physical activities and reduce medication use. Adding chiropractic care to usual medical care may make little to no difference in fear avoidance behaviour related to work activities. Although overall results are promising, establishing the role of chiropractic care in the management of low back pain requires large, rigorously conducted trials, that consider duration of pain (acute vs. chronic) as an effect modifier.

**Medical cannabis versus opioids for chronic noncancer pain: A systematic review and network meta-analysis of randomized clinical trials**

Haron M. Jeddi, Jason Busse, Behnam Sadeghirad, Mitch Levine, Micheal J. Zoratti, Li Wang, Atefeh Noori, Rachel Couban and Jean-Eric Tarride

McMaster University

**Introduction/Aim**: Increased awareness of harms associated with opioids for chronic noncancer pain has generated interest in medical cannabis as a therapeutic alternative. We explored their comparative effectiveness.

**Methods**: We searched EMBASE, MEDLINE, CINAHL, AMED, PsycINFO, PubMed, Web of Science, Cannabis-Med, Epistemonikos and the Cochrane Library (CENTRAL) to March 2021 for trials that randomized adults with chronic noncancer pain to medical cannabis vs. opioids, or to medical cannabis or opioids vs. placebo, and followed patients for ≥4 weeks. We performed Bayesian random-effects network meta-analyses to summarize the evidence and applied the GRADE approach to evaluate the certainty of evidence.

**Results**: Ninety trials involving 22 028 patients were eligible for review, among which the length of follow-up ranged from 28 to 180 days. Moderate certainty evidence showed that opioids provide small improvements in pain, physical functioning, and sleep quality vs. placebo; low to moderate certainty evidence supported similar effects for medical cannabis vs. placebo. Neither were more effective than placebo for role, social or emotional functioning (all high to moderate certainty evidence). Moderate certainty evidence showed there is probably little to no difference between medical cannabis and opioids for physical functioning (weighted mean difference [WMD] 0·47 on the 100-point SF-36 physical component summary score, 95% CrI -1·97 to 2·99), and cannabis results in fewer discontinuations due to adverse events vs. opioids (odds ratio 0·55, 95% CrI 0·36 to 0·83). Low certainty evidence suggested little to no difference between medical cannabis and opioids for pain relief (WMD 0·23cm on a 10cm visual analogue scale [VAS], 95% CrI -0.06 to 0·53) or sleep quality (WMD 0·49mm on a 100mm VAS, 95% CrI -4·72 to 5·59).

**Discussion/Conclusions**: Medical cannabis may be similarly effective and less harmful than opioids for chronic noncancer pain.

**Medical cannabis for chronic pain: A systematic assessment of equity considerations**

Omar Dewidar^a^, Jordi Pardo Pardo^b^, Vivian Welch^a^, Glen S. Hazlewood^c^, Andrea Darzi^d^, Cheryl Barnabe^c^, Kevin Pottie^a^, Jennifer Petkovic^a^, Shawn Kuria^b^, Zhiming Sha^b^, Sarah Allam^b^, Jason Busse^d^, Gordon H Guyatt^d^, Peter Tugwell^b^

^a^Bruyère Research Institute; ^b^University of Ottawa; ^c^University of Calgary; ^d^McMaster University

**Introduction/Aim**: **Background**: Chronic pain is the leading cause of disability worldwide. Medical cannabis may be effective for a minority of patients; however, access and impact may be affected by geographical as well as socioeconomic factors. As part of the process of developing a guideline on the use of medical cannabis for chronic pain, the guideline panel sought evidence to incorporate equity considerations into their recommendations using a novel approach.

**Objective**: To systematically identify and evaluate evidence from eligible primary studies to inform equity judgements; for example, regarding generalizability and subgroup effects.

**Methods**: We reviewed all studies identified in four systematic reviews summarizing the evidence for benefits and harms, opioid-sparing effects, long-term effects and values and preferences regarding cannabis for chronic pain. We extracted data on the following dimensions: place of residence, race, occupation, gender, religion, education, socioeconomic status, social capital, age, breastfeeding, and pregnancy.

**Results**: Of the identified 96 studies, 7% were conducted in low-to-middle income countries, 8% were focused on vulnerable populations, approximately half (47%) excluded patients with comorbidities, and 22% excluded pregnant or lactating women. Thirty-six percent of studies analyzed outcomes across at least one dimension of inequity. Differences in effects may exist across at least one dimension of inequities in 19% studies on benefits and harms, 43% studies on values and preferences and 11% studies on long-term effects. Little to no differences in effects across dimensions of inequities were identified for opioid-sparing effects.

**Discussion/Conclusions**: We developed and applied a new approach to systematically identify and assess equity considerations to inform the development of a guideline for cannabis and chronic pain. We anticipate that consideration of these issues will lead to more equitable guideline recommendations.

**Comparative effectiveness of corticosteroids for prevention of postoperative sore throat in adults undergoing tracheal intubation: A systematic review and network meta-analysis of randomized trials**

Vahid Ashoorion^a^, Alireza Malektojari^b^, Sara Ghazizadeh^b^, Fatemeh Mehrabi, Bita Mesgarpour, Leila Janani, Sara Moradi^a^, Patrick Jiho Hong^c^, Yvgeniy Oparin^a^, William Yao^a^, Rachel Couban^a^, Harsha Shanthanna^a^ and Jason Busse^a^

^a^McMaster University; ^b^Hormozgan University of Medical Sciences; ^c^University of Toronto

**Introduction/Aim**: Approximately half of patients that undergo endotracheal intubation experience postoperative sore throat (POST) after extubation. We aimed to determine the comparative effectiveness of competing corticosteroids for the prevention of POST, cough and hoarseness after extubation by performing a systematic review and network meta-analysis.

**Methods**: We searched MEDLINE, EMBASE, Web of Science, CINAHL, Scopus, and Cochrane Central Register of Controlled Trials from inception to February 2022 for trials that compared any formulation of corticosteroids to lubricant gel, placebo, saline, or no-treatment to prevent POST. We performed a random-effects network meta-analysis using a frequentist approach at three-time intervals (0-2h, 6-12h and 12.5-24h), and assessed the certainty of evidence using GRADE methodology.

**Results**: We included 44 trials involving 4,177 patients. Moderate certainty evidence showed that budesonide inhaler was among the most effective treatment to prevent POST: absolute risk reduction (ARR) 46% (95%CI: 31%- 48%) at 0-2h; ARR: 47% (95% CI: 42%-47%) at 6-12h and ARR: 40% (95% CI: 36%-40%) at 12.5-24h after extubation. High to moderate certainty evidence showed betamethasone applied on tube also reduced risk of POST, hoarseness and cough. Moderate certainty evidence showed that filling cuff with dexamethasone, IV dexamethasone, beclomethasone inhaler, gargling dexamethasone and applying dexamethasone on tube probably reduce the risk of POST 24h after surgery.

**Discussion/Conclusions**: Budesonide inhaler and betamethasone applied on tube were the most effective treatments to prevent POST after endotracheal intubation. Betamethasone applied on tube was the most effective approach to prevent hoarseness and cough after endotracheal intubation.

**Factors associated with initiating cannabis use after legalization in Canada: A cross-sectional study**

Vahid Ashoorion^a^, Behnam Sadeghirad^a^, Harman Sandhu^b^ and Jason Busse^a^

^a^McMaster University; ^b^University of Toronto

**Introduction/Aim**: Cannabis use has increased since the Government of Canada legalized nonmedical use in October 2018. We investigated demographic factors associated with initiating cannabis use following legalization.

**Methods**: We used data from the 2018 and 2019 National Cannabis Survey and constructed multivariable regression models. Respondents’ data were weighted and bootstrapped. We report relative measures of association as adjusted odds ratios (ORs) and absolute measures of association as adjusted risk increases (RIs).

**Results**: Among the 58,195 households surveyed, 28,566 provided complete data (49%) and our weighted analysis represented 27,904,258 Canadians aged ≥ 15 years. Approximately 1 in 5 Canadians endorsed use of cannabis (19.8%), predominantly for non-medical (9.5%) or combined medical and non-medical (5.8%) reasons. Those who initiated cannabis use in the past 3-months (1.9%) were more likely to be younger (25-34 yr vs. ≥65; adjusted OR 1.7, 95%CI 1.1-2.8; adjusted RI 1.1%, 95%CI 0.1%-2.0%) and endorse poor to fair vs. good to excellent physical health (adjusted OR 2.0, 95%CI 1.3-3.1; adjusted RI 1.7%, 95%CI 0.3%-3.1%). The 1% of Canadians who endorsed initiating use of cannabis due to legalization were more likely to reside outside of Quebec (adjusted OR 1.9, 95%CI 1.1-3.2; adjusted RI 0.5%, 95%CI 0.2%-0.9%).

**Discussion/Conclusions**: Canadians initiating cannabis use after nonmedical legalization were likely to be younger and endorse worse physical health, and half of those using cannabis reported therapeutic use. Stricter policies, lower social acceptance, and less availability of cannabis in Quebec appear to have curtailed initiation of use after legalization.

**Chronic disease group-based treatment: Applications for chronic pain**

Kelsey Haczkewicz, Natasha Gallant, Zona Iftikhar and Courtney Cameron

University of Regina

**Introduction/Aim**: In Canada, 1 in 3 individuals live with at least one major chronic disease.(i.e., cancer, chronic obstructive pulmonary disease, diabetes, ischemic heart disease, or heart failure). A common symptom of chronic disease individuals experience is pain which can, in turn, cause significant psychological distress. The current study was therefore aimed at understanding the types of psychological strategies and supports that are of interest to individuals living with chronic disease and associated symptoms such as pain.

**Methods**: A sample of 208 adults (26.9% male, 72.6% female, 0.2% non-binary) living with chronic disease completed a set of online self-report measures regarding their preferences for a new group focused on providing strategies and support for managing mental health for individuals living with chronic disease.

**Results**: More than 50 chronic diseases were self-reported and many of these participants suggested that pain management should be a focus of the group. Results also showed that 47.5% preferred the group be in a hybrid format, whereas 32.8% preferred the group be in a virtual format. Bi-weekly meetings of an hour or less in length were most preferred.

**Discussion/Conclusions**: Overall, our results provide insights into the format of group-based strategies and support for this population, and these findings also support the inclusion of a pain management module within the group.

**Opioid prescription and consumption following major joint replacement surgery**

Rachel Chin^a^, Sireedhorn Assavanop^b^, Sarah Tierney^c^, Sanjho Srikandharajah^d^, Yasmine Hoydonckx^b^, Jamal Kara^b^, Kawalpreet Singh^b^, Vincent Chan^b^ and Anuj Bhatia ^b^

^a^Queen’s University; ^b^Toronto Western Hospital and University of Toronto; ^c^University of Ottawa; ^d^North York General Hospital

**Introduction/Aim**: This study examines trends in post-discharge opioid use following hip and knee arthroplasty (THA, TKA) to determine appropriateness of opioid prescription and consumption.

**Methods**: Oral morphine equivalents (OMEQ) consumed in the first 48 hours after surgery and discharge opioid pill counts were recorded for 443 patients undergoing elective THA or TKA at an academic and a community hospital. Surveys were conducted 1-2, 6, and 12 weeks after discharge to collect data on consumption patterns and satisfaction. Data on the preoperative and 12-week psychological profiles of patients from TWH was also collected.

**Results**: Median opioid pill count at discharge was 60 [50,80]. Most patients reported that pain relief provided by their opioid prescriptions was “just right” (73.5%, 75.6%, and 78.2% at 1-2 weeks, 6 weeks, and 12 weeks, respectively). However, a significant proportion of patients had 20 or more pills leftover at each follow-up period (53.5%, 36.7%, and 33.9% of the cohort at 1-2 weeks, 6 weeks, and 12 weeks respectively). Significant associations were found between leftover pill count at 6 weeks and female gender, preoperative opioid intake, and surgery type (TKA vs. THA) (p=0.044, p=0.007, p=0.003 respectively); and between pain relief at 6 weeks and opioid consumption in first 48 hours after surgery, (p=0.025).

**Discussion/Conclusions**: While patients are generally satisfied with their opioid prescriptions, one third of them had over 20 pills remaining at the end of the 12-week period. This suggests that patients may be over-prescribed opioids after major joint surgery.

**Self-compassion moderates the relationship between pain interference and depression in a chronic pain sample**

Alanna Coady^a^, Kimberley Kaseweter^a^, Nina Gregoire^a^ and Susan Holtzman^b^

^a^University of British Columbia Okanagan; ^b^University of British Columbia

**Introduction/Aim**: Approximately one in four Canadians live with chronic pain. Of those, an estimated 18-85% will experience comorbid depression, resulting in worse health outcomes compared to chronic pain alone. Longitudinal studies demonstrate that greater pain interference (i.e., the inability to fully engage in activities due to pain) predicts an increased risk of developing depression. Promisingly, recent meta-analyses have demonstrated that self-compassion can be a protective factor against depression for those with chronic conditions. Evidence also suggests self-compassion is associated with lower depressive symptoms among people with chronic pain specifically. In order to determine if self-compassion can buffer the harmful sequelae of pain interference, the current study examined whether self-compassion moderated the relationship between pain interference and depressive symptoms.

**Methods**: Participants (N=303) were recruited from a specialized pain treatment clinic in British Columbia and completed a cross-sectional survey online.

**Results**: Moderation analysis revealed that, when controlling for pain severity, pain interference was significantly directly associated with depressive symptoms (b=2.21 SE=0.24, p < .001, 95% CI=1.63, 2.57), as was self-compassion (b=-8.91 SE=0.58, p < .001, 95% CI=-10.05, -7.78). Further, there was a significant interaction between self-compassion and pain interference, F(1, 298)=13.28, p < .001, such that the link between pain interference and depressive symptoms was attenuated among patients who reported higher self-compassion.

**Discussion/Conclusions**: Findings highlight the protective potential of cultivating self-compassion among those with chronic pain, and particularly those who are experiencing high levels of pain interference.

**Reconsolidation blockade with propranolol as a novel treatment for chronic pain - a double-blind feasibility study**

Alexia Coulombe-Leveque^a^, Sylvie Lafrenaye^a^, Alain Brunet^b^, Serge Marchand^a^ and Guillaume Léonard^a^

^a^Université de Sherbrooke; ^b^McGill University, Douglas Hospital

**Introduction/Aim**: Chronic pain is characterized by increased connectivity in the nervous system, similar to that observed in patients with post-traumatic stress disorder (PTSD). The aim of this study was to investigate whether reconsolidation therapy, a new treatment for PTSD that consists in reactivating the hyperactive synapses encoding the threat response (by remembering/describing the trauma) and blocking their reconsolidation using propranolol, is feasible with a chronic pain population.

**Methods**: We conducted a double-blind, placebo-controlled study with 24 adults suffering from chronic lower-back pain (< 3 months) and no contra-indication to propranolol. All participants received pain education (10 short videos) and attended 6 weekly sessions where they received 40-80 mg of propranolol (n=12) or placebo one hour before a short reactivation procedure (description of painful events/movements). Feasibility outcome measures included recruitment rates and side effects frequency/severity; efficacy was measured 4 weeks post-intervention (Brief Pain Inventory).

**Results**: Feasibility: 67 patients were screened over 6 months; 24 participants were enrolled in the study and 22 participants completed the follow-up. Four participants showed an asymptomatic decrease in heart rate during 1 or 2 sessions, and a fifth experienced headache and nausea following her second session.

**Efficacy:** no clinically meaningful difference was observed between the BPI scores of the two groups.

**Discussion/Conclusions**: Reconsolidation therapy is a feasible intervention for chronic pain. Preliminary results suggest no improvement in function of pain; however, we noted prevalent catastrophic/kinesiophobic discourse during the sessions. We hypothesize that correcting these negative pain beliefs might be a prerequisite (unmet in this study) for the success of the intervention.

**Motivations, perceptions, and effects of cannabis use in individuals with mood and anxiety disorders**

Ankita Das^a^, Stefan Kloiber^b^

^a^Institute of Medical Science, University of Toronto; ^b^University of Toronto

**Introduction/Aim**: Cannabis use is common in individuals with mood and anxiety disorders, the most prevalent mental health conditions in Canada and worldwide. In the era of cannabis legalization, medical cannabis program and a variety of available cannabis products in Canada, there is controversy and uncertainty about the potential risks and benefits of cannabis in such individuals.

**Objective**: To conduct a systematic review of the current scientific literature on perceptions, motivations, knowledge, and effects of cannabis use in individuals with mood and anxiety disorders and then comparing the results from this systematic review with data from a clinical study using a mixed methods approach to assess perceptions and patterns of cannabis in this patient population.

**Methods:** (1) Semi-structured anonymous survey to comprehensively assess and understand patterns of cannabis use, potential risks and areas of potential benefits, subjective knowledge and perceptions around CU in individuals with mood and anxiety disorders including OCD and PTSD. (2) Focus groups and in-depth interviews as a qualitative approach to specifically assess the motivations for cannabis use, expectations of the effects of cannabis on cognition, mood, anxiety, and behavior. (3) Systematic review and synthesis of the current literature of human studies on patterns, perceptions, motivations, attitudes, knowledge, or effects of CU in individuals with mood, anxiety, trauma-related, or obsessive or compulsive disorders/symptoms.

**Results**: Cannabis use appeared to provide symptom relief for mood disorders but seemed to exaggerate depressive symptoms over time. Most frequent users identified cannabis use for mental health conditions, sleep problems and pain management for somatic pain as the most common reasons. In contrast, those with social anxiety expected greater cognitive and behavioural impairment from cannabis use. Some veterans with PTSD reported reasons related to relief of side effects of psychotherapy medication, to facilitate social competency and for direct confrontation of the source of trauma. Medical users reported failing to meet responsibilities because of use and problems with concentration/memory after use. Participants reported differential experiences and effects of products predominantly containing THC or CBD. Cannabis products with high CBD was reported to be particularly helpful for sleep problems and pain including migraines. On the other hand, reported effects of cannabis with high THC content included enhanced creativity, focus, and libido, helpful to overcome inhibitions and be more extroverted.

**Discussion/Conclusions**: Combined results from interviews, survey and systematic review indicate that about half of individuals reported young age (< 18) when initiating CU, with curiosity, peer pressure and acceptance but also treatment of mental health symptoms among most common motives for initiating CU. The majority (> 60%) of individuals with specific mental health conditions using cannabis report and perceive their CU for medical reasons. Nearly 50% of cannabis users reported CU for medical and at the same time recreational purposes creating a problematic overlap of using a substance for treatment as well as recreationally. Despite > 60% reporting CU for medical reasons, only 20% reported this being recommended and/or prescribed, and only a small number (14.6%) obtained information about CU from a medical practitioner. Also, the majority (> 50%) reported using inhalation as method for CU which is associated with medical problems related to smoke inhalation. An additional risk identified was that the majority (53.1%) of individuals obtained products from non-regulated sources, e.g. dealer. While mental health symptoms were among the most frequent reasons for starting and continuing CU and while participants reported positive effects on some symptoms, concerns and negative effects were clearly reported as well, most frequently worsening of cognition (memory, concentration) energy, motivation, productivity. Although CCU reported experiencing various symptoms and aspects of CUD very few reported motivation for change and reported higher peer approval including concerning aspects such as passing out or driving after CU or frequent/daily CU. Reported motivations and explanations for CU were side effects associated with pharmacological treatment, or CU being perceived as safer than alcohol use though CCU reported higher alcohol consumption compared to PCU and NCU. In addition, while insomnia was frequently reported a reason for CU, participants reported no clear effect on sleep except for improved sleep onset. The systematic review additionally revealed potential risk constellations such as anxiety sensitivity, social anxiety, depression being more frequently associated with CU as well as increased risk for problems related to CU.

Data from this research to inform development of engagement, treatment and prevention strategies addressing concerning aspects of CU in this population as well as additional research on potential beneficial effects and constellations.

**Update, adaptation to the Canadian clinical practice context and validation of a new version of the medication quantification scale: The MQS-4.0**

Gwenaelle De Clifford-Faugère^a^, Hermine Lore Nguena Nguefack^a^, Marimée Godbout-Parent^a^, Mamadou Aliou Diallo^a^, Line Guénette^b^, Gabrielle Pagé^c^, Manon Choinière^c^, Sylvie Beaudoin^d^, Aline Boulanger^e^, Anne Marie Pinard^f^, David Lussier^g^, Philippe De Grandpré^h^, Simon Deslauriers^i^ and Anaïs Lacasse^j^

^a^Université du Québec en Abitibi-Témiscamingue (UQAT); ^b^Centre de recherche du CHU de Québec - Université Laval; Faculté de pharmacie, Université Laval; ^c^Centre de recherche, Centre hospitalier de l’Université de Montréal (CHUM); Département d’anesthésiologie et de médecine de la douleur, Faculté de médecine, Université de Montréal; ^d^Laboratoire de recherche en épidémiologie de la douleur chronique, Université du Québec en Abitibi-Témiscamingue (UQAT); ^e^Centre d’expertise en gestion de la douleur chronique, Centre hospitalier de l’Université de Montréal (CHUM); Département d’anesthésiologie et de médecine de la douleur, Faculté de médecine, Université de Montréal; ^f^Centre d’expertise en gestion de la douleur chronique, CHU de Québec-Université Laval; Département d’anesthésiologie et de soins intensifs, Université Laval; Centre intégré de recherche en réadaptation et intégration sociale, Centre intégré de santé et de services sociaux (CIUSSS) de la Capitale-Nationale; ^g^Institut universitaire de gériatrie de Montréal; Département de médecine, Faculté de médecine, Université de Montréal; ^h^Familiprix Chantale Gaboury & Marie-Ève Gélinas; GMF Clinique familiale des Prairies; ^i^VITAM - Centre de recherche en santé durable, Centre intégré universitaire de santé et de services sociaux (CIUSSS) de la Capitale-Nationale; ^j^Département des sciences de la santé, Université du Québec en Abitibi-Témiscamingue (UQAT)

**Introduction/Aim**: Quantifying real-world risks associated with pain medications poses several methodological challenges, especially considering that polypharmacy is very common among persons living with chronic pain (CP). To meet these challenges, an index for quantifying the risk associated with analgesics, called the Medication Quantification Scale (MQS), was developed in the US and last updated in 2003. This study aimed to provide an updated version of the MQS based on the current Canadian clinical practice context.

**Methods**: In Step1, an expert committee (clinicians/researchers: n=10) adapted the MQS to the current Canadian clinical practice context (face and content validity). Step 2 was an update of risk weights given to medication subclasses using a prescriber (physicians/pharmacists/nurse practitioners: n=207) and patient (n=141) perception survey (risk weights were derived from median 0-10 scores given to each medication subclass). Step 3 assessed the construct validity of the MQS-4.0 after applying risk weights to the medication use profile of 9,122 ≥12-year-old persons living with CP covered by the Quebec public prescription drug insurance.

**Results**: The expert committee identified 36 medication subclasses for which people living with CP and prescribers have expressed their perception of risk. When applied to prescription claims, the MQS-4.0 score was positively correlated (p < .05) with the original MQS score and variables known to be associated with polypharmacy (e.g., Charlson Comorbidity Index, number of prescribers, healthcare visits).

**Discussion/Conclusions**: This study provides clinicians and the scientific community with an updated index for the quantification of the risk associated with polypharmacy in persons living with CP.

**Pharmacological treatment of fibromyalgia in Québec and comparison with clinical guidelines**

Gwenaelle De Clifford-Faugère^a^, Hermine Lore Nguena Nguefack^a^, Marimée Godbout-Parent^a^, Mamadou Aliou Diallo^a^, Line Guénette^b^, Gabrielle Pagé^c^, Manon Choinière^c^, Sylvie Beaudoin^d^, Aline Boulanger^e^, Anne Marie Pinard^f^, David Lussier^g^, Philippe De Grandpré^h^, Simon Deslauriers^i^ and Anaïs Lacasse^j^

^a^Université du Québec en Abitibi-Témiscamingue (UQAT); ^b^Centre de recherche du CHU de Québec - Université Laval; Faculté de pharmacie, Université Laval; ^c^Centre de recherche, Centre hospitalier de l’Université de Montréal (CHUM); Département d’anesthésiologie et de médecine de la douleur, Faculté de médecine, Université de Montréal; ^d^Laboratoire de recherche en épidémiologie de la douleur chronique, Université du Québec en Abitibi-Témiscamingue (UQAT); ^e^Centre d’expertise en gestion de la douleur chronique, Centre hospitalier de l’Université de Montréal (CHUM), Département d’anesthésiologie et de médecine de la douleur, Faculté de médecine, Université de Montréal; ^f^Centre d’expertise en gestion de la douleur chronique, CHU de Québec-Université Laval, Département d’anesthésiologie et de soins intensifs, Université Laval; Centre intégré de recherche en réadaptation et intégration sociale, Centre intégré de santé et de services sociaux (CIUSSS) de la Capitale-Nationale; ^g^Institut universitaire de gériatrie de Montréal; Département de médecine, Faculté de médecine, Université de Montréal; ^h^Familiprix Chantale Gaboury & Marie-Ève Gélinas; GMF Clinique familiale des Prairies; ^i^VITAM - Centre de recherche en santé durable, Centre intégré universitaire de santé et de services sociaux (CIUSSS) de la Capitale-Nationale; ^j^Département des sciences de la santé, Université du Québec en Abitibi-Témiscamingue (UQAT)

**Introduction/Aim**: Fibromyalgia is defined as chronic widespread pain. Lack of evidence on certain medications such as opioids and benzodiazepines limit their use, whereas antidepressants and anticonvulsants are effective and safe according to evidence. This study aimed to describe real-world pharmacological treatments used by persons living with fibromyalgia and compare them to treatment guidelines.

**Methods**: Directive interviews were conducted by telephone with 63 individuals self-reporting a diagnosis of fibromyalgia (Quebec, Canada). The questionnaire addressed specific questions about their pain and pharmacological treatments currently used for pain management (prescribed and over-the-counter). Descriptive analyses were performed. Quebec and Canadian fibromyalgia treatment guidelines were used as well as published evidence reports of Health technology assessment organizations.

**Results**: 56% of participants reported another diagnosis in addition to fibromyalgia. Despite a lack of scientific evidence supporting opioid use or benzodiazepines, they were respectively used by 33.3% and 27.0% of our sample. Non-steroidal anti-inflammatory drugs were used by 54.0% of participants, although this medication is not recommended due to its lack of efficacy. Muscle relaxants and tramadol, which are recommended, were used respectively by 25.4% and 23.8% of participants. Among the medications strongly recommended for their efficacy and safety, anticonvulsants were used by 36.5%, serotonin-norepinephrine reuptake inhibitor antidepressants by 55.6%, and tricyclic antidepressants by 22.2%. Cannabinoids (17.5%) and medical cannabis use (34.9%) was also reported.

**Discussion/Conclusions**: Results reveal obvious discordance between evidence-based recommendations and real-word medication use, which highlights the complexity of the pharmacological treatment of fibromyalgia and the need for further real-world evidence.

**Getting in the flow: Investigating the relationship between intrinsically pleasant tasks and pain reduction**

Zoha Deldar ^a^, Jerome Genzling^b^, Roman Sarrazin-Gendron^b^, Sophie Desjardins^c^, Mathieu Roy^b^, Najmeh Khalili-Mahani^d^, Lea Fhima^b^, Zoe Arvanitis^b^ and Stephany Dumas^b^

^a^McGill University; ^b^McGill University; ^c^McGill University; ^d^Concordia University

**Introduction/Aim**: This study aimed to investigate how flow experience affects pain modulation. While pain is a natural response that captures our attention to protect us from harm, it can be reduced by distraction techniques, which redirect our focus away from the source of pain. As pain and cognitive tasks compete for shared resources, it is essential to understand how to modulate attention to painful stimuli. Demanding cognitive tasks have been shown to reduce pain; however, they are often perceived as unpleasant and sub-optimal due to the aversive effort required. Tasks that are intrinsically pleasant, tied to passion, and create a flow experience (i.e., a state of complete absorption in the task linked to passion and intrinsic rewards) are more likely to produce analgesia. When experiencing flow, the task occupies shared mental resources, leading to increased performance and pain reduction.

**Methods**: The study recruited 66 healthy young volunteers, divided into novice and advanced chess player groups, who completed 5 blocks of easy, flow, and difficult chess puzzles, a 2-back task, and a pain-alone task while receiving thermal pain stimulations. They reported pain intensity and unpleasantness and completed game experience and flow questionnaires after each block.

**Results**: Preliminary analyses showed that solving chess puzzles significantly reduced pain intensity and unpleasantness compared to the 2-back and pain-alone tasks between groups. Additionally, flow experience was significantly different between blocks.

**Discussion/Conclusions**: Flow experience can be crucial in cognitive pain reduction, and future research should consider this effect when examining the efficacy of distraction on pain inhibition.

**Improving access by using a collaborative stepped triaging approach: a pilot project**

Eliane Domingue^a^, Rima Noormohamed^b^, Evan Ward^c^, Corinne Bryant^d^ and Magali Robert^e^

^a^Alberta Health Services; ^b^Alberta Health Services, College of Registered Nurses of Alberta; ^c^Mosaic Primary Care Network; ^d^Calgary Foothills Primary Care Network; ^e^University of Calgary

**Introduction/Aim**: The Canadian Pain Task Force has identified one of its goals as improving timely access to patient-centered care.1 At the Calgary Chronic Pain Centre (CPC), a tertiary pain centre, approximately 1500 referrals are on the wait list resulting in a 14 month wait. Four of seven Primary Care Networks (PCN) in the Calgary area offer a pain program with less than a one month wait. The objective of this study was to improve patient access to pain programs by implementing a stepped care approach.

1. Canadian Pain Task Force Report: March 2021- Canada.ca

**Methods**: Through a collaborative process between the four PCNs and CPC, referrals were redirected from CPC to PCN pain programs for individuals who had not attended the PCN pain program and met the eligibility criteria. This information was relayed to the patient and the referring physician.

**Results**: Collaborative networking was done over six months. Between Jan 2022 to July 2022, 217 referrals from CPC were reviewed to identify eligible patients in the geographic location of the participating PCNs. This allowed 74/217 s (34%) to be redirected. This resulted in those individuals entering a program within a month rather than 14. If 34% of referrals could be redirected then this would result in the CPC wait time decreasing from 14 months to 9.25 months.

**Discussion/Conclusions**: The initial objective of using a stepped care triage approach to improve access to care was successful. This study identified the need for further collaboration with Primary Care Networks to optimize the triage process including defining eligibility criteria to ensure timely access.

**Outcomes associated with brain stimulation combined with intensive rehabilitation for youth with chronic pain**

Spencer Epp^a^, Adam Kirton^b^, Nivez Rasic^a^, Catherine Lebel^a^, Frank MacMaster^a^, Melanie Noel^a^, Laura Rayner^b^, Joanne Vallely^b^ and Jillian Vinall Miller^a^

^a^University of Calgary; ^b^Alberta Children’s Hospital

**Introduction/Aim**: Intensive Interdisciplinary Pain Treatment (IIPT) programs provide day-treatment therapy to help youth with chronic pain (pain for > 3 months) and functional disability. IIPT is associated with decreases in pain interference, related to decreases in middle frontal gyral (MFG) activity. Repetitive transcranial magnetic stimulation (rTMS) of the MFG has been shown to be a safe and effective treatment for adults with chronic pain. rTMS was added to our IIPT intervention for youth.

**Methods**: Currently, 11 youth (of a proposed 25) have completed IIPT with rTMS. rTMS is applied to the MFG at 10Hz for 40 pulses over 4s with an inter-train interval of 26s. Sessions last 37.5min every weekday for three weeks, alongside IIPT. At baseline and discharge from IIPT participants report on their pain interference. On days 1, 6 and 13, participants report on their symptoms following rTMS. Paired T-testing is used to evaluate changes in pain interference. Descriptive statistics were used to examine reports of adverse symptoms following rTMS.

**Results**: Preliminary analysis revealed a decrease in pain interference from baseline (mean: 64.8) to discharge (58.3, p < 0.05). The most frequently reported adverse event was neck pain (n=7), however, fewer youth report neck pain by week three (n=5), and all but one youth reported experiencing less intense neck pain by week three.

**Discussion/Conclusions**: To date, the addition of rTMS to IIPT has maintained previously reported decreases in pain interference post-IIPT. Despite the majority reporting neck pain in the first week, all youth completed the combined three-week rTMS and IIPT intervention.

**Case report: The feasibility of rTMS in a patient with intrathecal baclofen pump for the treatment of unresolved neuropathic pain following spinal cord injury**

Stevie Foglia^a^, Ravjot Rehsi^a^, Claudia Turco^b^, Harsha Shanthanna^a^ and Aimee Nelson^a^

^a^McMaster University; ^b^University of Alberta

**Introduction/Aim**: The purpose of this study was to assess the efficacy of repetitive transcranial magnetic stimulation (rTMS) to treat neuropathic pain (NP) refractory to pharmacological intervention in a patient with complete spinal cord lesion with ongoing treatment with intrathecal baclofen pump. We also aimed to investigate the feasibility of rTMS with an intrathecal baclofen pump.

**Methods**: This study involved 6-weeks of rTMS performed 5-days per week (treatment), a 6-week follow up period with no stimulation, and an 8-week top-up session period. 10 Hz rTMS was delivered to the left primary motor cortex for 2004 pulses. Pain was classified as pressure pain in left foot, burning pain in buttocks, burning pain in sternum, and electrical attacks in the trunk. Assessments included the numerical rating scale (NRS), neuropathic pain scale (NPS), Hamilton Depression and Anxiety rating scales.

**Results**: A 30, 13, and 29% reduction in sternum, buttocks, and left foot pain respectively was observed following the treatment period. This was associated with a 38% decrease in NPS score, and a 65 and 25% reduction in anxiety and depressions scores respectively. Following the third week of treatment, the number of electrical attacks was zero. The change in pain persisted for 1 week following treatment. Top-up sessions did not reduce pain to the level achieved during the treatment period. rTMS stimulation did not cause any interference with the functioning of the intrathecal baclofen pump.

**Discussion/Conclusions**: rTMS may therefore be beneficial for patients with NP who are refractory to pharmacological intervention with a greater effect on certain pain phenotypes.

**Community linkages for equity-oriented chronic pain care**

Nicole George and Sara Ahmed

McGill University

**Introduction/Aim**: Chronic pain impacts nearly 1 in 5 Canadian adults, with demonstrated inequities in the prevalence, severity, and barriers to care. Linkages between health systems and community resources can add to the continuum of care by addressing unmet needs, such as the social determinants of health. The aim of this research is to co-design tailored community linkages within a chronic pain self-management program at a multidisciplinary rehabilitation center.

**Methods**: Through integrated knowledge translation, stakeholders (individuals living with pain, clinicians, researchers, and program decision-makers, n=8) engaged in a series of 2-hour online meetings. Audio recordings were transcribed, analyzed iteratively, and mapped to the PRECEDE-PROCEED model. Areas of integration were further operationalized by mapping to the biopsychosocial model of pain and social determinants of health.

**Results**: The defined goal of the community-linked program is for individuals living with pain to stay active physically, mentally, and socially. Factors influencing this goal were categorized as predisposing (e.g., knowledge of resources, level of pain), reinforcing (e.g., the influence of peers, caregivers, health professionals), and enabling (e.g., accessible delivery, cost, language, age). Identified community areas included resources for breaking isolation and engaging in meaningful activities (e.g., cultural events). Final prioritized areas and mapping will be presented.

**Discussion/Conclusions**: Health systems require strategies to better connect individuals with chronic pain to resources in the community to help them stay active physically, mentally, and socially. Leveraging the strengths of the community through tailored linkages can support equity-oriented care that reflects the multidimensional experience of living with chronic pain.

**Combination Analgesic Development for Enhanced Clinical Efficacy (the CADENCE trial): A double-blind, randomized, controlled crossover trial of an alpha-lipoic acid - pregabalin combination for the treatment of fibromyalgia pain**

Ian Gilron, Sylvia Robb, Dongsheng Tu, Ronald Holden, Roumen Milev and Tanveer Towheed

Queen’s University

**Introduction/Aim**: Drug therapy for fibromyalgia is limited by incomplete efficacy and dose-limiting adverse effects (AEs). Combining agents with complementary analgesic mechanisms – and differing AE profiles – could provide added benefit.

**Methods**: We assessed an alpha-lipoic acid (ALA)-pregabalin combination with a randomized, double-blind, 3-period crossover design. Participants received maximally tolerated doses of ALA, pregabalin, and ALA-pregabalin combination—for 6 weeks. Primary outcome was daily pain (0-10); secondary outcomes included Fibromyalgia Impact Questionnaire, SF-36 survey, Medical Outcomes Study Sleep Scale, Beck Depression Inventory (BDI-II), adverse events, and other measures.

**Results**: The primary outcome of daily pain (0-10) during ALA (4.9), pregabalin (4.6), and combination (4.5) was not significantly different (p=0.54). There were no significant differences between combination and each monotherapy for any secondary outcomes although combination and pregabalin were both superior to ALA for measures of mood and sleep. ALA and pregabalin maximal tolerated doses were similar during combination and monotherapy and AEs were not more frequent with combination therapy.

**Discussion/Conclusions**: These results do not support any additive benefit of combining ALA with pregabalin for fibromyalgia. The observation of similarly reached maximal tolerated drug doses of these two agents (which have differing side effect profiles) during combination and monotherapy – without increased side effects – provides support for future development of potentially more beneficial combinations with complementary mechanisms and non-overlapping side effects.

**Cold and vibration for children undergoing needle-related procedures: A non-inferiority randomized clinical trial**

Arianne Ballard^a^, Christelle Khadra^a^, Samara Adler^a^, Emilie Parent^b^, Olivier Fortin^a^, Estelle Guingo^c^, Evelyne D. Trottier^d^, Benoit Bailey^d^, Naveen Poonai^e^ and Sylvie Le May^a^

^a^Université de Montréal; ^b^Université de Sherbrooke; ^c^Université du Québec en Abitibi-Témiscamingue; ^d^Centre Hospitalier Universitaire de Ste Justine; ^e^Schulich School of Medicine and Dentistry, Western University

**Introduction/Aim**: The aim of this study was to determine if a cold vibrating device was non-inferior to a topical anesthetic cream for pain management in children undergoing needle-related procedures in the Emergency Department (ED). 

**Methods**: In this randomized controlled non-inferiority trial, we enrolled children aged between 4-17 years presenting to the ED and requiring a needle-related procedure. Participants were randomly assigned to either the cold vibrating device or topical anesthetic groups. The primary outcome was the mean difference in procedural pain intensity on the 0-10 Color Analogue Scale (CAS), using a non-inferiority margin of 0.70.

**Results**: A total of 352 participants were randomized (cold vibration device n=176, topical anesthetic cream n=176). Mean (SD) age of children recruited was 9.8 (3.9) years. Procedural pain scores’ mean difference between groups was 0.56 (95% CI: -0.08–1.20) on the CAS, showing that the cold vibrating device was not non-inferior to topical anesthetic.

**Discussion/Conclusions**: The non-inferiority of the cold vibrating device over a topical anesthetic cream was not demonstrated for pain management in children during a needle-related procedure in the ED. Despite this, and because topical anesthetic creams require an application time, had a cost per unit and are underused in the ED setting, the cold vibrating device remains a promising alternative as it is a rapid, easy-to-use and reusable device.

**AVATAR - Effects of a customized avatar development within an immersive multi-platform virtual environment on pain and anxiety of hospitalized children in hemato-oncology**

Estelle Guingo^a^, David Paquin^a^, Casey Côtes-Turpin^a^, Christine Genest^b^, Léandra Desjardins^c^, Pascal Bernier^c^, Michel Duval^d^, Cathy Vézina^e^, Marie-France Langlet^d^, Félix Côtes-Charlebois^a^ and Sylvie Le May^b^

^a^Université du Québec en Abitibi-Témiscamingue; ^b^Université de Montréal; ^c^Centre Hospitalier Universitaire de Sainte-Justine; ^d^Centre Hospitalier Universitaire de Ste Justine; ^e^Université du Québec en Abitibi-Témicamingue

**Introduction/Aim**: Our objective was to study the feasibility, acceptability and effects of an immersive multi-platform distraction (virtual reality and mobile) based on a customized avatar, on pain and anxiety of hospitalized children in hemato-oncology.

**Methods**: This qualitative research-action design study aimed to recruit five children (6-17Y.), currently hospitalized in hemato-oncology in pediatric hospital. Feasibility and acceptability were measured by surveys. Data was collected during semi-structured interviews with both child and one of his parents. Analyses has been done through thematic content analysisusing NVivo. Qualitative data on parents’ perceptions of the intervention was collected through a logbook. Clinicians’ perception was also collected using open-questions surveys. Our study followed a co-design approach in art-based research where children are involved in the game conception by drawing their virtual friend (avatar).

**Results**: Recruitment process is undergoing. We already recruited and collected data on three child-parents dyads. Preliminary results show the following themes: “Anxiety effect,” “mood effect” and “improvement possibilities.” So far, surveys have shown that children and their parents are satisfied with the intervention and consider that the intervention has a positive effect on both the anxiety and pain of children.

**Discussion/Conclusions**: Preliminary results show potential for the use of customized avatar for anxiety and pain management of this group of children. Moreover, our results underline the effects of virtual reality and avatar on children’s mood, by providing distraction for medical procedures and hospital environment.

**An evaluation of pain BC’s peer-delivered online support groups: Participant satisfaction and valued outcomes**

Sage Wiebe^a^, Melanie McDonald^b^, Carmelle Jaeggle^b^ and Susan Holtzman^a^

^a^The University of British Columbia; ^b^Pain BC

**Introduction/Aim**: Social support is a critical determinant of mental and physical health among people living with chronic pain (PLCP). Yet, PLCP frequently report high levels of social isolation and a perceived lack of understanding from social networks. Pain BC is a non-profit organization offering a wide range of psychosocial supports for PLCP, including online, peer-delivered support groups. The objective of this study was to evaluate participant satisfaction with Pain BC’s support groups, and to identify which aspects were perceived as most valuable.

**Methods**: Pain BC’s peer-delivered support groups are held twice-monthly in nine geographic regions across BC. Between April and November 2021, anonymous online surveys were sent out after each group session to obtain participant feedback.

**Results**: 743 responses were obtained. Satisfaction was high, with 97% of responses describing the group session as “excellent,” or “very good.” Responses indicated agreement that participants felt respected (99%), welcome (99%), and that the content was useful and easy to understand (96%). Results were similar between geographic-specific groups (e.g., Vancouver, Kootenays) and the group with participants from across BC. A content analysis of 237 responses to an open-ended question about the most valuable take-away from each session will be presented.

**Discussion/Conclusions**: PLCP face systemic barriers to healthcare and often lack support in their daily lives. Findings highlight the perceived value of peer-delivered, online support groups and key factors that may drive satisfaction. In an era of greater openness to, and necessity of, virtual care, findings also support the potential for expanding peer-delivered online groups across provincial borders.

**Healthcare utilization of youth with chronic pain before and 12 months after participating in an intensive interdisciplinary pain treatment**

Karen Hurtubise^a^, Melanie Noel^b^, Astrid Brousselle^c^, Nivez Rasic^d^ and Chantal Camden^e^

^a^McMaster Universty; ^b^Univeristy of Calgary; ^c^Univeristy of Victoria; ^d^University of Calgary; ^e^Université de Sherbrooke

**Introduction/Aim**: Repeated use of healthcare resources renders chronic pain among the highest-costing health conditions in childhood. Identifying treatments that reduce the use of these resources is imperative. This study aimed to analyze the changes in healthcare utilization in a sample of youth with chronic pain, who participated in intensive interdisciplinary pain treatment (IIPT).

**Methods**: Healthcare utilization from a healthcare system perspective was evaluated. From a database of IIPT participants, 30 youth were randomly selected. Anonymized healthcare utilization data (for the year pre- and 12 months post-treatment) were extracted from the provincial health organization data systems for 28 of these youth for which data was available. Changes in healthcare utilization were calculated using Wilcoxon signed-ranked tests, using a significance of 0.05.

**Results**: Of the 28 participants, a decrease in specialist visits was noted in 10 IIPT participants (63%), and significant changes were noted in appointments with allied health professionals for many participants 12 months post-IIPT. Occupational therapy appointments decreased for 9 participants (56%), physiotherapy sessions diminished for 17 participants (74%), and psychology visits were reduced for 12 participants (55%). No statistical changes were observed in hospitalization or emergency visits.

**Discussion/Conclusions**: Unlike previously published findings, our analysis highlighted a significant reduction in healthcare utilization for many youth with chronic pain 12 months following participation in an IIPT. Future research is required with a larger sample and a control group comparator accessing another treatment, exploring associated costs and cost-savings to establish the cost-effectiveness of these programs in Canada.

**Have we had it backwards: Does analgesic response in spinal cord stimulation drive pain catastrophizing?**

Neha Kanojia, Anuj Bhatia, Victoria Bains, Jamal Kara and Kawalpreet Singh

Toronto Western Hospital

**Introduction/Aim**: Spinal cord stimulation (SCS) can treat chronic neuropathic pain. Numerical Rating Scale (NRS) scores assess analgesic response. Patient Global Impression of Change (PGIC) scale gives composite report of the experience. The psychological evaluation by Pain Catastrophizing Scale (PCS) and improvements in PCS scores correlate with analgesic benefit. The objective of this study was to explore the changes in PCS scores in response to the changes in pain intensity and PGIC during trials and following implants of SCS.

**Methods**: 135 patients with neuropathic pain conditions were enrolled. The patients underwent a SCS trial and a ≥ 50% reduction in pain NRS was considered as the criterion for success. NRS, PGIC, and PCS scores were collected at the end of the SCS trial. In SCS implant patients, reduction of pain NRS by ≥ 30% at 1 year after the implant was considered as a responder. The responders and non-responders were also classified on the basis of PGIC. PCS scores were compared between responders and non-responders after SCS trial and a year after SCS implant.

**Results**: In SCS trial and implant group, NRS scores were significantly low in responders as compared to non-responders. There was no significant difference between the change in PCS scores in SCS trial group. Similarly, the PCS scores were significantly lower in the SCS implant responders as compared to the non-responders when PGIC was used to categorize patients.

**Discussion/Conclusions**: Patients with successful SCS implants reported a decrease in pain catastrophizing as compared to those experiencing successful SCS trials.

**Trajectories of psychological distress among individuals with chronic pain during the COVID-19 pandemic**

Kimberley Kaseweter^a^, Nina Gregoire^a^, Mark Nazemi^b^, John-Paul Etheridge^c^, W. Francois Louw^c^, Vishal Varshney^c^ and Susan Holtzman^a^

^a^University of British Columbia Okanagan; ^b^Thrive Health; ^c^University of British Columbia

**Introduction/Aim**: Chronic pain (CP) care is complicated by its multidimensional nature, which manifests with complex physical and psychological comorbidities. Although there is a well-established link between psychological stress and pain-related functioning, little is known about the trajectory of psychological functioning among people with CP over the course of the COVID-19 pandemic.

**Methods**: Our study aimed to determine whether the degree of psychological comorbidities changed in conjunction with the trajectory of the pandemic in a large sample of people with CP. Intake questionnaires from 4772 patients at CP specialty clinics in British Columbia (completed between March 2020 and June 2022) were examined. Bivariate analyses and line graphs were used to examine and visualize the relationships between pain catastrophizing (PCS), anxiety (GAD-7), and depression (PHQ-9) with date of intake completion.

**Results**: Results revealed small negative correlations between the helplessness (r=-.09, p < .01) and rumination (r=-.08, p < .01) subscales of the PCS and questionnaire date. However, line graphs revealed a peak in catastrophizing scores in August 2020. Meanwhile, GAD-7 and PHQ-9 scores remained fairly stable, with average levels in the minimal-to-mild and mild-to-moderate range, respectively.

**Discussion/Conclusions**: The small spike in catastrophizing in August 2020 maps onto the onset of Canada’s second COVID-19 wave, and is in line with recent data from the United States that similarly found increased pain catastrophizing during a peak in COVID cases in July 2020. Mild to moderate depressive symptoms across the duration of the pandemic also highlight the ever-present need for addressing psychiatric symptoms in pain care.

**Real-world pain relief trajectories among persons using cannabis as a treatment for chronic pain**

Anaïs Lacasse^a^, Hermine Lore Nguena Nguefack^a^, Reza Sharif^b^, Mélanie Bérubé^c^, Claudie Audet^a^ and Nancy Julien^a^

^a^Université du Québec en Abitibi-Témiscamingue (UQAT); ^b^McGill University; ^c^Université Laval

**Introduction/Aim**: Evidence regarding cannabis as a treatment for chronic pain (CP) is needed. This study aimed to describe pain relief trajectories among persons living with CP using cannabis.

**Methods**: Strainprint® data was used (a mobile app that tracks symptoms for which medical cannabis is used). All ≥18-year-old users reporting pain at first use of the app and who used the app for ≥3 months to track their pain symptoms were selected (n=2,360; age:18-76yr; women:61%). When a person uses cannabis, he/she enters pre- and post-use pain severity measures in the app (0-10 scale). As users can consume cannabis for multiple symptoms at once, they can have multiple entries per session and multiple sessions per day. Pain relief was operationalized in an aggregated measure using weeks as the time unit: % of pain-related entries for which a user achieved a clinically important decrease in pain severity (≥2-point). Group-based trajectory modelling was applied to these weekly repeated measures over a 3-month period to identify subgroups of users with similar patterns over time (pain relief trajectories).

**Results**: Three trajectories were found: (1) Users reporting clinically important pain relief almost all of the time (95-98% of the time) (n=1,120); (2) Pain relief most of the time (≥71% of the time) with a slight 3-month increase in effectiveness (n=847); (3) Pain relief occasionally (29-41% of the time) with a decrease in effectiveness over time (n=393).

**Discussion/Conclusions**: Individual-centered statistical approaches are an interesting angle of analysis to understand who is most likely to respond to cannabis.

**Certified child life specialist in a pediatric emergency department: Impact of a quality improvement project to improve procedural pain and distress management for needle procedure**

Céline Thémelin, Jocelyn Gravel, Kaitlen Gattuso, Marie-Joëlle Doré-Bergeron, Patricia Laforce, Julie Paquette, Nathalie Gaucher, Charlotte Grandjean-Blanchet, Marie-France Langlet, Sarah Loemba, Valérie Leclair, Annie Lacroix, Corinne Thériault and Evelyne D. Trottier

CHU Sainte-Justine

**Introduction/Aim**:

**Background**: Combining coping strategies can help reduce children’s pain and distress during needle procedures in the emergency department (ED).

**Objective**: To evaluate the use of strategies to reduce procedural pain/distress for children in the pediatric ED, with the implementation of an institutional quality improvement (QI) initiative working with a Certified Child Life Specialist (CCLS).

**Methods**: The QI initiative Tout doux aims to alleviate procedural pain/distress. To evaluate the impact of this project on the use of coping strategies during needle procedures in the ED, CCLS interventions were recorded over a one-year period. The number of strategies used during needle procedures was reported to track progress monthly throughout the integration of the QI initiative Tout doux.

**Results**: Between Sept 2021-Aug 2022, the CCLS was present for 397 needle procedures (Mean age: 6yo [0months-17yo]). Procedures included 56% IV-line insertions, 30% venous blood draws and 14% capillary blood tests. Parents were present at 99% of procedures. Preparation strategies were used in 65%, distraction in 98% and deep breathing in 46%. Topical anesthetic was offered for 37% of procedures. The proportion of children receiving at least 3 strategies alleviating procedural pain/distress increased to more than 90% during the last 7 months (except one very busy month) in comparison to 22%-66% for the first 5 months with the implementation of the QI initiative Tout doux.

**Discussion/Conclusions**: The QI initiative Tout doux increased the use of combined coping strategies with the help of a CCLS. Further studies will need to evaluate impact on patients and the sustainability of their use.

**Key to success to improve best practices: A nurse champion to increase institutional use of procedural pain and distress management strategies**

Patricia Laforce, Julie Paquette, Marie-Joëlle Doré-Bergeron, Kaitlen Gattuso, Sarah Loemba, Annie Lacroix, Bénédicte Grou and Evelyne D. Trottier

CHU Sainte-Justine

**Introduction/Aim**: Since studies have shown that nurse champions are facilitators in implementing best practices, a procedural pain nurse champion (PPNC) became a key part of the Tout doux Quality Improvement (QI) institutional initiative to improve procedural pain/distress management.

**Aim**: To assist the healthcare providers (HCP) in the reduction of children’s procedural pain/distress by increasing the use of coping strategies with the help of a PPNC.

**Methods**: The PPNC coordinates and adapts local deployments with the nursing/medical heads of each sector, including the creation of a local multidisciplinary Tout doux committee. Audits are conducted to assess the baseline use of coping strategies, including prevention, psychological, physical and pharmacological strategies (4Ps). HCP receive mandatory training to enhance their knowledge on the 4Ps. The PPNC offers field support through procedural coaching to translate knowledge into practice. Post-deployment audits are then completed to evaluate the use of the combined strategies.

**Results**: Between June 2021-October 2022, Tout doux has been deployed in 13 sectors. More than 400 audits were conducted pre-deployment. More than 150 post-deployment audits have been completed in 6 sectors. Results show improvement in the use of two or more strategies from 160/234 (68%) procedures pre-deployment to 132/150 (89%) procedures post.

**Discussion/Conclusions**: Deploying the Tout doux project on procedural pain and distress management improved the combined use of coping strategies with the support of a dedicated PPNC. The next step will be to ensure its dissemination throughout the institution and further assess the long-term outcome on patients.

**Educational strategies to improve procedural pain and distress management in a tertiary pediatric hospital**

Julie Paquette, Patricia Laforce, Marie-Joëlle Doré-Bergeron, Annie Lacroix, Kaitlen Gattuso, Yann Poirier, Marie-France Langlet, Sarah Loemba and Evelyne D. Trottier

CHU Sainte-Justine

**Introduction/Aim**: In order to improve the use of procedural pain/distress coping strategies and create long-lasting changes in practice, educational strategies for healthcare providers (HCP) need to be diverse, dynamic and interdisciplinary.

**Aim**: To increase and diversify the educational opportunities for HCP, in order to enhance training and the use of procedural pain/distress management strategies in a pediatric hospital.

**Methods**: The QI initiative Tout Doux aims to improve procedural pain/distress management in a tertiary pediatric hospital. With Tout Doux, various educational strategies were developed by HCP and patient-family advisors, including a mandatory training on best practices for pain/distress management. Currently employed HCP were offered the choice between an e-learning or a formal presentation. Educational strategies also included short videos, one-pagers and simulations. Data on the number of HCP trained by sector were collected and, when given the choice, their preferred method of training.

**Results**: Between June 2021-July 2022, a total of 1539 HCP were trained on coping strategies for procedural pain/distress management with the integration of Tout doux. Each newly hired HCP (702/1539, 46%) were trained on best practices as part of their training. Currently employed HCP, who were given the choice, preferred the e-learning module on coping strategies (643/837, 77%). In addition, 238 HCP completed an additional e-learning training on psychological strategies.

**Discussion/Conclusions**: Through diverse educational strategies, a QI project aiming to improve procedural pain/distress management in a pediatric institution trained more than 1500 HCP. When given the choice, more than 75% of current HCP preferred the e-learning training compared to the formal lecture.

**Evidence of effective knowledge mobilization and transmission among Canadian Francophone healthcare network on procedural and acute pain management in children**

Sarah Loemba, Marie-Joëlle Doré-Bergeron, Patricia Laforce, Julie Paquette, Kaitlen Gattuso, Annie Lacroix, Evelyne D. Trottier and Emilie Trempe

CHU Sainte-Justine

**Introduction/Aim**: Effective knowledge mobilization and transmission (KMT) is crucial within an institution and its network to facilitate meaningful clinical practice changes for procedural pain management.

**Aim**: To illustrate how the availability of KMT of evidence-based strategies on pediatric procedural pain/distress prevention/management has facilitated meaningful collaborations and dialogue throughout a national network of Francophone health institutions.

**Methods**: Since 2021, CHU Sainte-Justine has partnered with the organization Solutions for Kids in Pain(SKIP) and deployed a hospital-wide quality improvement project (QI) called Tout doux, aimed to improve procedural pain/distress management for patients/families locally. Since then, interest emerged from the francophone health institution network and Tout doux provided support and shared locally-developed French resources to health care providers (HCP) requiring specific or general knowledge on procedural pain management in children. Addressing these needs, Tout doux conducted virtual or on-site presentation for this network on evidence-based strategies to improve pain management.

**Results**: Between 2021/06-2022/12, 80 HCP throughout 46 healthcare organizations have contacted Tout doux to receive support on best practices in procedural pain prevention/management. 205 HCP throughout 13 hospitals have attended virtual or on-site training on the subject. Through a recently developed post-evaluation survey, 16/16 (100%) HCP said they have learned concepts/messages that has encouraged practice changes and 16/16 (100%) HCP are very/extremely satisfied with the adequacy of the resources and tools provided for their practice.

**Discussion/Conclusions**: With the growing interest of the network, the KMT between Tout doux and its network permitted transmission of evidence-based knowledge to improve procedural pain management practices for children and their families.

**Who responds to treatment? Factors associated with pain catastrophizing outcomes in pediatric intensive interdisciplinary pain treatment**

Rob D. Long^a^, Andrew Walker^a^, Jillian Vinall Miller^a^, Si Chen Pan^a^, Laura Rayner^b^, Melanie Noel^a^, Joanne Vallely^b^ and Nivez Rasic^a^

^a^University of Calgary; ^b^Alberta Children’s Hospital

**Introduction/Aim**: Intensive Interdisciplinary Pain Treatment (IIPT) is the gold standard for youth with chronic pain and functional impairment. Though studies have examined the relationship between baseline patient factors and post-treatment pain intensity, emerging research shows that pain catastrophizing outcomes may be more important to improving functional disability and wellbeing. This study explores patient factors associated with clinically significant post-treatment pain catastrophizing. 

**Methods**: Pain catastrophizing (PCS) scores were acquired in 45 IIPT patients aged 12 to 18 years at intake (baseline), discharge, and 3-month follow-up. Patients were dichotomized at discharge/follow-up based on being above or below clinically defined thresholds of high pain catastrophizing (≥26). Univariate logistic regression was used to assess unadjusted associations between patients who remained above these thresholds and baseline age, PROMIS® depression and anxiety, PedsQLTM quality of life social subscale, PCS, and parent pain catastrophizing. 

**Results**: Higher baseline PCS, depression, and anxiety scores were associated with significantly increased odds of high post-treatment PCS scores. Age, social functioning, and parent catastrophizing were not significantly associated with high post-treatment scores. 

**Discussion/Conclusions**: Differences in PCS outcomes may be explained by its association with emotional dysregulation. Patients with high PCS scores were unaffected by conventional IIPT treatment. Implementation of interventions that target emotional dysregulation (e.g., Acceptance Commitment Therapy or Dialectical Behavioral Therapy) may be beneficial for individuals who have high baseline PCS. This research provides support for using baseline questionnaires to help inform individualized IIPT programming for patients who may require additional, or modified, interventions.

**Comparing intensive pain rehabilitation outcomes for youth with headache, neuropathic, or musculoskeletal pain**

Charles Mabutas, Nivez Rasic, Laura Rayner, Si Chen Pan, Melanie Noel and Jillian Vinall Miller

University of Calgary

**Introduction/Aim**: A 3-to-6-week multidisciplinary, day-treatment, Intensive Pain Rehabilitation Program (IPRP) was developed at the Alberta Children’s Hospital (ACH) to help youth with chronic pain (pain > 3 months). Uniquely, the IPRP at ACH provides intervention for all types of chronic pain. It is not known at present which pain subgroups experience the greatest benefit from IPRP.

**Methods**: Between April 2016 to November 2022, 23 youth (age=16.2, 78% female) participated in IPRP and filled out baseline, discharge, and 3-month follow-up questionnaires. Eight youth had headache pain, ten youth had neuropathic pain, and five had musculoskeletal pain. At each time point, youth filled out the PROMIS pain interference questionnaire. T-scores were used for analysis. A higher t-score represented greater pain interference. One-way ANOVAs and linear mixed models were used to compare change in pain interference over time, both between and within groups.

**Results**: At baseline, youth with neuropathic pain (M=69.3) had significantly higher pain interference than youth with either headache (M=62.9) or musculoskeletal pain (M=64.3). Across the three-month period, pain interference of the neuropathic pain group remained significantly higher as compared to youth with headache pain (p=0.02). Across the three groups, only youth with headache showed a significant reduction in pain interference between baseline and three-month follow-up (p=0.02).

**Discussion/Conclusions**: Youth with neuropathic pain had the greatest pain-related disability over time; however, youth with chronic headache appear to benefit the most from IPRP intervention. Further investigation will be required to determine which components of IPRP may be most effective for managing youth with higher pain-related disability.

**Pain informed movement program for people with knee osteoarthritis: A feasibility trial**

Shirin Modarresi^a^, Neil Pearson^b^, Kim Madden^c^, Margaret Fahnestock^c^, Dawn Bowdish^c^ and Lisa C. Carlesso^c^

^a^Michael DeGroote Institute for Pain Research and Care (IPRC), McMaster University; ^b^University of British Columbia; ^c^McMaster University

**Introduction/Aim**: Pain in knee osteoarthritis (KOA) is complex and not well-managed. We aimed to establish the feasibility of a program which we call ‘Pain Informed Movement’ consisting of neuromuscular exercise, mind-body techniques, and pain neuroscience education (PNE) in people with KOA. This program has the potential to further our understanding of how to harness intrinsic pain modulation to improve pain management for KOA.

**Methods**: Single-arm feasibility trial with a nested qualitative component. Primary outcome: complete follow-up. Inclusion criteria: age ≥40years, KOA diagnosis or meeting KOA NICE criteria, and pain intensity ≥3/10. Intervention: 8 weeks of twice weekly in-person group exercise sessions, with a third at-home session, and PNE and mind-body techniques provided as videos and integrated into the exercise sessions. Assessment: clinical questionnaires, physical tests including blood draws at baseline and program completion. Secondary outcomes: program acceptability, burden, rates of recruitment, compliance and adherence, and adverse events. A priori success criteria were used. Participants were invited to an online focus group.

**Results**: 19 participants were enrolled, with a complete follow-up rate of 74% (mean age 63.3 years (SD 10.5), 73% female), indicating modifications were necessary to proceed. All other success criteria were met. The focus groups revealed that demonstrations of the mind-body techniques in the videos would be beneficial.

**Discussion/Conclusions**: The Pain Informed Movement program is feasible, but minor modifications are needed to proceed. A pilot two-arm randomized controlled trial will be conducted to explore potential effects of Pain Informed Movement compared to conventional neuromuscular exercise and standard OA education.

**The effects of combined motor control and isolated extensor strengthening versus general exercise on paraspinal muscle morphology and function in patients with chronic low back pain: A randomized controlled trial**

Maryse Fortin^a^, Meaghan Rye^a^, Alexa Roussac^a^, Chanelle Montpetit^a^, Jessica Burdick^a^, Neda Naghdi^a^, Brent Rosenstein^a^, Cleo Bertrand^a^, Luciana G. Macedo^b^, Geoffrey Dover^a^, James Elliott^c^, Richard DeMont^a^, Michael H. Weber^d^ and Véronique Pepin^a^

^a^Concordia University; ^b^McMaster University; ^c^University of Sydney School of Health Sciences; ^d^McGill University

**Introduction/Aim**: 1) To compare the effects of combined motor control and isolated lumbar strengthening exercise (MC + ILEX) versus a general exercise (GE) program on paraspinal muscle morphology and function, and 2) investigate if changes in paraspinal muscle are associated with improvement in pain, functional status (ODI) and quality of life (SF-12).

**Methods**: A total of 50 participants with chronic LBP were randomly allocated to each group (MC+ILEX, n=25; GE, n=25). Both groups completed a 12-week supervised intervention program (2 sessions/week). IDEAL fat-water magnetic resonance imaging (MRI), ultrasound assessments and self-reported questionnaires were acquired at baseline, 6- and 12-week to assess the effect of each intervention on multifidus (MF) and erector spinae (ES) muscle morphology (e.g., cross-sectional area (CSA); and fatty infiltration) and function (e.g., MF thickness change from a rested to contracted state) at L4-L5 and L5-S1, respectively.

**Results**: A mixed model ANOVA with repeated measures revealed significant time*group interactions for MF and ES CSA at L4-L5 (p < 0.01) and L5-S1 level (p < 0.001). A significant decrease in ES fatty infiltration was also observed in the MC+ILEX group at L5-S1, however the time*group interaction was not significant. There was no change in MF function. While both groups experienced significant improvements in pain, function and quality of life, no correlation between the changes in functional scores and MF or ES morphology was noted.

**Discussion/Conclusions**: Participants with chronic LBP included in the MC+ILEX group achieved significantly greater improvement in MF and ES morphology as compared to the GE group.

**Transition to virtual care services during COVID-19 at Canadian pain clinics: Survey and future recommendations**

Victoria Borg Debono^a^, Samuel Neumark^b^, Norman Buckley^a^, Ramesh Zacharias^c^, Eleni Hapidou^d^, Jennifer Anthonypillai^d^, Susy Faria^d^, Carrie-Lynn Meyer^d^, Thomas Carter^e^, Nadia Parker^e^, Brenda Lau^f^, Emmanuel Abreu^g^, Scott Duggan^h^, Etienne J Bisson^h^, Josie Pierre^i^, Regina Visca^j^ and Patricia Poulin^k^

^a^McMaster University; ^b^University of Toronto; ^c^Chronic Pain Centre of Excellence for Canadian Veterans; ^d^Hamilton Health Sciences; ^e^CBI Health Clinics; ^f^CHANGEpain clinic; ^g^CHANGEpain Clinic; ^h^Kingston Health Sciences Centre; ^i^Ste-Anne’s Hospital; ^j^McGill University Health Centre; ^k^University of Ottawa

**Introduction/Aim**: The COVID-19 pandemic led healthcare centers to shift to virtual formats quickly. This study aimed to describe and compare the transition from in-person to virtual pain care services at Canadian pain clinics during the onset of the COVID-19 pandemic and provide post-pandemic recommendations for pain care services to optimize patient care.

**Methods**: A qualitative participatory action study design was used, including a cross-sectional survey for data collection. The survey was administered to leadership teams of 11 adult pain clinics affiliated with the Chronic Pain Centre of Excellence for Canadian Veterans. The data were qualitatively analyzed and categorized into themes and subthemes. Results were shared independently with participants to verify the accuracy and relevancy of the data prior to knowledge dissemination.

**Results**: We achieved a 100% response rate (n=11). All clinics collectively transitioned 72 services from in-person to virtual formats. The results focus on describing the transition process to virtual care, current treatments and services, quality of patient care, program sustainability, barriers to maintaining virtual services, and future considerations. Most clinics (n=9) reported that the overall quality of patient care was unchanged before and after the transition.

**Discussion/Conclusions**: The pandemic demonstrated the feasibility and sustainability of hybrid virtual and in-person care for treating those with pain. It is recommended there should be a hybrid of both virtual and in-person care for pain clinics moving forward, and Ministries of Health should continue to support innovations aimed at holistic healthcare, interdisciplinary teams, and the expansion of clinics’ geographical reach for patient access.

**Repetitive peripheral magnetic stimulation for phasic heat pain modulation**

Evgeny Osokin^a^, Rossi Tomin^a^, James Khan^b^, Ali Mazaheri^c^, David Seminowicz^d^ and Massieh Moayedi^a^

^a^University of Toronto; ^b^Mount Sinai Hospital; ^c^University of Birmingham; ^d^Western University

**Introduction/Aim**: Existing pain management tools, such as transcutaneous electrical stimulation (TENS) and pharmacology have limited efficacy and can be associated with significant side effects. Repetitive peripheral magnetic stimulation (rPMS) is a novel, non-pharmacological technique that can induce non-invasive analgesia. To date, there are no high-quality controlled studies that demonstrate its efficacy. Thus, the aim of the study was to determine whether rPMS can reduce experimental phasic heat pain.

**Methods**: 54 healthy participants (27M, 27F), aged 18-40 years, consented to procedures approved by University of Toronto’s Human Research Ethics Board. Participants attended two sessions: a control session and a stimulation session, counterbalanced across subjects. In the stimulation session, participants received one of three stimulations: TENS or rPMS. RPMS parameters were either intermittent theta burst stimulation (iTBS), or continuous TBS (cTBS). Phasic heat pain stimuli were applied before and after stimulation. Outcome measures collected included pain intensity, pain unpleasantness, and the area of secondary hyperalgesia. A repeated-measures ANCOVA was run in each sex to determine the effect of stimulation type on outcome measures.

**Results**: There was a significant effect of the stimulation arm on pain intensity and secondary hyperalgesia in females (F=3.603, p=0.044 and F=6.697, p=0.005, respectively), but not in males (p > 0.05). There was no significant effect of the treatment arm on pain unpleasantness both in females and males (p > 0.1).

**Discussion/Conclusions**: These results show that rPMS can potentially impact central sensitization in women.

**Sustained changes in wellbeing in patients with chronic pain attending an intensive chronic pain management program during the COVID - 19 pandemic**

Jiyeon Park^a^, Jennifer Anthonypillai^b^ and Eleni G. Hapidou^b^

^a^McMaster University; ^b^McMaster University and Hamilton Health Sciences

**Introduction/Aim**: Chronic pain may result in a decline of one’s wellbeing and is strongly linked to depression, anxiety and catastrophizing. The aims of the study were to examine sustained changes in wellbeing in patients with chronic pain 6 months after attending an intensive interdisciplinary pain management program.

**Methods**: Data were collected from 37 (48%) participants of the intensive interdisciplinary chronic pain management program at the Michael G. DeGroote Pain Clinic, Hamilton Health Sciences, who responded to the follow-up survey 6 months after discharge (40% females, 62% Veterans, 76% Virtual program). Participants completed psychometric instruments including emotional distress, pain interference, stages of change, acceptance of pain, and subjective happiness.

**Results**: ANOVAs demonstrated maintenance of significantly improved outcomes for depression, catastrophizing, kinesiophobia, sensitivity to pain traumatization, recent bothersome symptoms, pain interference, self-management strategies, pain acceptance, and happiness at follow-up (p < 0.001). Results also showed many significant interactions among the factors examined with most pointing towards better outcomes in females and veterans overall. Male civilians fared worse than all others on pain interference, bothersome symptoms, depression, anxiety (trend), catastrophizing, kinesiophobia, sensitivity to pain traumatization, and stages of change (p < 0.05).

**Discussion/Conclusions**: Results highlight the effectiveness of the program in decreasing emotional distress/improving wellbeing in patients with chronic pain long after discharge, during the pandemic. They also point to differences in outcomes between males and females, veterans and civilians. Findings support and strengthen those of previous studies in our program and the literature and point to the need to improve outcomes of male civilians.

**The lived experience of people with chronic pain undergoing a combined opioid deprescribing and self-management intervention**

Katherine Poser, Jordan Miler, Kevin Varette, Chad McClintock, Nicole Bobbette, Abey Abebe and Kyle Vader

Queen’s University

**Introduction/Aim**: To reduce the burden and suffering of those living with chronic pain, primary care providers have prescribed opioids at increasing rates. Opioid prescription has led to an increase in opioid misuse and opioid-related deaths in epidemic proportions. In response, opioid deprescribing and self-management programs have shown promising results. This study explores the experiences of patients who participated in an intervention that combines opioid deprescribing and self-management.

**Methods**: An interpretive descriptive design was used. Participants were eligible for this study if they 1) had non-cancer related chronic pain 2) were on a daily opioid dose of > / 50mg morphine equivalents and 3) participated in a combined opioid deprescribing and 6-week chronic pain self-management intervention in primary care. 15 participants completed semi-structured interviews after completing the intervention. All interviews were audio recorded and transcribed. Thematic analysis and a constant comparative approach were used as the main analytic strategies.

**Results**: Key themes constructed from the data were empowerment, impact of a therapeutic relationship, application of knowledge, physical comorbidities, inclusion of mental health support, and accessibility.

**Discussion/Conclusions**: While patient participants expressed a reduction in pain, improved function and quality of life, and feeling supported with continued implementation of the intervention, their feedback suggests potential adjustments to the intervention are required. Mandatory detailing to ensure supportive communication among participants, alteration of the delivery schedule to provide a more combined intervention, and the need for both virtual and in-person offerings are key areas for improvements to the intervention.

**Improvements in parent mental health and responses to pain moderate child brain connectivity and decreases child pain interference following intensive pain rehabilitation**

Sankait Rattu^a^, Si Chen Pan^b^, Nivez Rasic ^a^, Melanie Noel^a^, Laura Rayner^c^, Catherine Lebel^a^ and Jillian Miller^a^

^a^University of Calgary; ^b^Sir Winston Churchill High School; ^c^Alberta Children’s Hospital

**Introduction/Aim**: With Intensive Pediatric Pain Rehabilitation (IPRP), parents are taught how to best support their child with chronic pain. Given the critical role of the parent in their child’s daily care, it is imperative to identify whether IPRP is associated with changes in parents’ mental health and behaviour, and whether these changes contribute to improvements in their child’s pain-related disability.

**Methods**: Data was collected on 22 parents and their respective children, aged 10-18 years. Pre- and post-IPRP, parents and children completed questionnaires regarding their own pain catastrophizing and anxiety symptoms. Additionally, parents completed questionnaires related to their responses to their child’s symptoms. Patients underwent a 3T MRI scan pre- and post-IPRP. Diffusion tensor imaging (DTI) and fractional anisotropy (FA), a quantitative measure of white matter microstructure, were acquired. Paired t-tests were used to compare parent and patient questionnaire data pre- and post-IPRP. Linear mixed models will be used to examine whether parents’ mental health and behaviour moderates FA values to reduce child pain interference pre- to post-IRPP.

**Results**: Parent anxiety, catastrophizing, and distraction/monitoring behaviours significantly decreased pre- to post-IPRP (p < 0.05). Child pain interference also significantly decreased (p < 0.001). The next steps will be to determine whether decreases in parent anxiety, catastrophizing, distraction/monitoring behaviours, and changes in FA are associated with decreased child pain interference pre- to post-IPRP.

**Discussion/Conclusions**: Parental mental health and behaviour improve pre- to post-IPRP. It will be important to examine whether improvements in parental functioning help to facilitate improvements in child outcomes.

**Adjuvant pharmacotherapy with spinal COR stimulation – A help or a hindrance? A scoping review**

Amarnath Reddy Basavanapalli^a^, Sachin Sahni^a^, Anuj Bhatia^a^, Pranab Kumar^a^ and Bengt Linderoth^b^

^a^Toronto Western Hospital; ^b^Karolinska Institutet

**Introduction/Aim**: Spinal cord stimulation (SCS) can ameliorate neuropathic pain. The analgesic benefit from SCS may be altered by the use of adjuvant analgesic medications by patients who recieve SCS implants. We undertook a scoping review to scan and summarize the evidence for modulation of SCS therapy by adjuvant pharmacotherapy.

**Methods**: A literature review of SCS studies on humans and animals was performed by using medical databases including MEDLINE, EMBASE, CINAHL, Cochrane CENTRAL and Google scholar from inception until October 31, 2022. Data extraction and analysis was done on the effect of pharmacotherapy on pain and related domains and adverse effects.

**Results**: 22 studies, 13 on humans and 9 on animals, were identified. In human studies, SCS non-responders with neuropathic pain reported substantial improvement with administration of intrathecal baclofen, clonidine and ketamine. The suppressive effect of SCS on mechanical hypersensitivity was enhanced with antidepressents., However, gabapentenoids did not augment the analgesic benefit from SCS. Patients who eliminated opioid use, or who were opoid naive, had superior clinical outcomes with SCS compared to those who continued opoids. In animal studies, SCS augmentation was seen with intrathecal GABA, baclofen and clonidine. In one study, intrathecal ketamine enhanced pain relieving effect of SCS on tactile hypersensitivity and play a role in converting SCS non-responders to responders.

**Discussion/Conclusions**: This review suggests adjunctive pharmacotherapy may have a role in enhancing analgesic benefits from SCS therapy. Further prospective comparitive studies are required to establish the impact of adjuvant pharmacotherapy on SCS for pain.

**Nerve blocks in patients with chronic headaches, neck, and low back pain: Preliminary results of an interventional study at pain care clinics in ontario**

Rifat Rehmani^a^ and Hany Demian^b^

^a^Pain Care Clinics, Mississauga; ^b^McMaster University; Pain Care Clinics

**Introduction/Aim**: To evaluate the effectiveness of nerve blocks in improving pain and function among adults with chronic headaches, neck pain, and low back pain.

**Methods**: The study was conducted prospectively at Pain Care Clinics in Ontario. A one-group pretest and post-test design to determine the analgesic and functional effects of 8- treatments of bupivacaine injection in patients with chronic low back pain, headaches, and chronic neck pain. A sample size of 597 subjects was calculated by using PASS software.

Primary outcomes were assessed utilizing the numeric pain rating scale and disease-specific functional status questionnaires. Secondary outcome measures were studied by Brief Pain Inventory, Pain Disability Index, Anxiety and Depression by Hospital Anxiety and Depression Scale, and Short Form 36 (SF-36) physical and mental health component scores. Descriptive statistics was performed. The difference amongst the pretest and post-test groups was analyzed by two-sided paired t-test.

**Results**: To date, 343 patients completed the study. Of those, 271 had back pain, 61 has neck pain and 11has chronic headaches. 80% of the patients were females. The numeric pain rating scale was 7.2 ± 0.6 in the pretest while it was 2.1 ± 0.5 in the post-test group (P=< .001). There was also significant improvement in the disease-specific functional status scores. The analyses of secondary outcome also revealed functional status improvement (P=< 0.001).

**Discussion/Conclusions**: In this preliminary analyses, 8-treatments of bupivacaine injection in patients with chronic low back pain, headaches and chronic neck pain provide a greater reduction in pain and improve function.

**Demographic and baseline measures of patients entering a tertiary pain center**

Magali Robert^a^, Eliane Domingue^b^, Andrea Bezuidenhout^b^ and Andrew Walker^b^

^a^University of Calgary; ^b^Alberta Health Services

**Introduction/Aim**: Understanding the characteristics of the referral base of patients entering a tertiary care chronic pain center is paramount in addressing both external and internal unmet needs.

**Methods**: All patients entering the Calgary Chronic Pain Program (a tertiary level care program with three streams: Neuro-musculoskeletal (NMSK) pain, pelvic pain or post-concussion headache) are sent a battery of questionnaires to fill online through RedCap. Data was extracted to provide an overview of demographics and intake measures at time of participant program initiation. Summary statistics were compiled from patients entering the program from February 2020 to February 2022.

**Results**: Summary intake statistics were collected for 1125 patients, including intake demographics. Length of chronic pain experienced ranged from 8.4 ± 10.8 years to 11.7 ± 10.9 years in post-concussion headache and NMSK patients, respectively. Across all streams, most patients were female, Canadian and in a relationship. Superior scores for intake measures were noted for pelvic patients.

**Discussion/Conclusions**: Demographic information is not reflective of the Calgary population and weighted to a Canadian common law/married population. The means of the intake measures were similar amongst the streams with a trend showing pelvic pain patients as having less dysfunction. However, intimacy and sexual function were not measured. The wide confidence intervals surrounding the intake measures reveals a diversity of presentations. This study informs changes to target the referral base to vulnerable populations and consider intake criteria for participation that characterizes a tertiary level pain program.

**Exploratory evaluation of patient trajectory through a one-year tertiary care chronic pain program**

Magali Robert^a^, Andrea Bezuidenhout^b^ and Andrew Walker^b^

^a^University of Calgary; ^b^Alberta Health Services

**Introduction/Aim**: The Calgary Chronic Pain Clinic (CPC) is a one-year interdisciplinary pain clinic. Following patient trajectories could identify patients who are not thriving earlier and allow mitigating strategies.

**Methods**: Patients entering (CPC) enter the same measures on intake, at six months and on exit. At completion they are asked about their perceived success. This outcome was dichotomized to improved or no change/worse. Measures include: Short Musculoskeletal Function Assessment, Chalder Fatigue Questionnaire, Pain Catastrophizing Scale, Brief Pain Inventory (BPI), Pain Self-Efficacy Questionnaire (PSEQ), Satisfaction with Life, Patient Health Questionnaire 4, EQ-5D. Scatter plots were generated for each intake measure and trajectories explored using a polynomial curve fit. A sensitivity analysis was completed whereupon missing data was imputed using predictive mean matching.

**Results**: Ninety-six patients completed the one-year program and provided outcome measures of their perceived success. 75 patients (78.1%) reported improvement. Specifically, at the 6-month time point, no discernable/clinically important difference in measures can be observed between patients who improved and those who reported no change/worse outcomes. This was seen for all measures. Although imputation served to reduce the width of the 95% confidence interval, no appreciable separation of trajectories was noted at 6 months.

**Discussion/Conclusions**: This exploratory study recognizes the importance of using measures to inform care. Using available and imputed data, no discernable difference was seen for all measures at 6 months with respect to patients who will improve or report no change/worse outcomes upon discharge. The paucity of data at 6 months lends itself to such a finding. On-going analysis will continue as patients progress through the program.

**Comparison of outcome measures and perceived improvement following completion of a one-year interdisciplinary chronic pain program**

Magali Robert^a^, Eliane Domingue^b^, Andrea Bezuidenhout^b^ and Andrew Walker^b^

^a^University of Calgary; ^b^Alberta Health Services

**Introduction/Aim**: All outcome questionnaires measure different domains and aspects of health. Changes from baseline may not represent an impression of improvement. This study looks at changes in domain questionnaires with perceived success, which was dichotomized into improved and no change or worse.

**Methods**: Upon discharge patients complete a questionnaire to assess their status satisfaction (scored from 1 to 7) compared to intake. Responses were dichotomized as improvement or no change/worse status. This was compared to the outcome measures at discharge. Missing data was imputed using predictive mean matching for sensitivity analysis with complete cases. Independent samples t-tests were used to compare outcome measures between groups.

**Results**: 96 patients complete the outcome measures and were included in analysis. Missing data was up to a maximum of 20% for outcome measures. Appreciable differences were noted for SMFA daily activities and bothersome scores, Satisfaction with Life scores, PSEQ scores and PCS scores. Imputed results (not shown) were similar to those presented for complete case analysis.

**Discussion/Conclusions**: This exploratory study begins to establish norms for individuals who report improvement and individuals who report no change or worsening following completion of a one year interdisciplinary program. Even those who considered themselves improved presented with outcome values below population normals in the EQ5D-5L, Short Musculoskeletal Function Assessment, Chalder Fatigue Scale. However, those who improved showed Satisfaction with Life scores that were comparable to normal population values.

**One-on-one care distribution amongst providers in an interdisciplinary pain clinic**

Magali Robert^a^, Kimberly Musselwhite^b^ and Eliane Domingue^b^

^a^University of Calgary; ^b^Alberta Health Services

**Introduction/Aim**: Distribution of care in an interdisciplinary pain clinic is critical for strategic planning and improving delivery of care.

**Methods**: Measurement of time and number of patients seen by providers at the Calgary Chronic Pain Center was undertaken between Jan 1, 2022 and Sept 30, 2022. The time does not include leading group activities or indirect patient care (such as planning or meetings). The nurses take on a coordinator role and their direct patient time is thus not reflective of their other responsibilities.

**Results**: The direct time for one-on-one care is similar for physicians, physiotherapists and psychologists. Less social worker time was likely a result of a gap in filling the position rather than less need during the study period. Most providers have a 1:4 new to follow up appointment ratio.

Nursing time was also on a 1:1 ratio. Total rehab time compared to physician time was in a 1:2.

**Discussion/Conclusions**: The Calgary Chronic Pain Clinic functions at a 1:1:1 ratio between physician:physiotherapist:psychologist one-on-one time. A 2:1 time ratio for rehabilitation and physician is seen. This does not include time spent in delivering group sessions. Similarly nursing time is similar to physician time. This data can guide strategic planning and recruitment.

**Bias in pain care: What factors do providers report as influencing their treatment decisions?**

Margaret A. Rose-McCandlish^a^, Tracy M. Anastas^b^, Megan M. Miller^c^ and Adam T. Hirsh^a^

^a^Indiana University-Purdue University Indianapolis; ^b^University of Washington School of Medicine; ^c^Cincinnati Children’s Hospital Medical Center

**Introduction/Aim**: Providers often treat pain differently based on patient race and socioeconomic status (SES). Whether providers perceive these factors as influencing their decisions remains to be known. We examined a) which patient factors providers report as influencing their treatment decisions, and b) whether providers who exhibit racial or SES biases in their decisions (“biased providers”) report different factors as influencing these decisions compared to providers who do not exhibit biases (“non-biased providers”).

**Methods**: Physician residents/fellows (N=434; “providers”) in the US made treatment decisions for 12 computer-simulated patients with chronic pain who differed by race and SES. Providers then rated the extent to which 15 different patient factors influenced their treatment decision-making.

**Results**: Providers rated patient demographic factors (e.g., race, sex/gender, age) as significantly less influential in their treatment decision-making than other, pain-specific factors (e.g., etiology, duration; p < 0.05). Biased and non-biased providers significantly differed in their self-reported ratings of treatment influences (F(1,430)=4.44, p=0.04). Although none of the pairwise comparisons were significant, the largest differences were observed for biased providers giving higher ratings for demographic (sex/gender, race, age) and social (occupation, interference with social relationships) factors, as well as for provider intuition.

**Discussion/Conclusions**: Providers reportedly placed less weight on patient demographic factors than on pain-specific factors when deciding which pain treatments to recommend. Providers who exhibited racial or SES biases in their treatment decisions reported being more influenced by a range of factors than did non-biased providers; however, the extent to which this reflects self-aware intentional behavior requires future study.

**Pediatric pain and mental health trajectories over the course of interdisciplinary pain treatment**

Brittany N. Rosenbloom^a^, Gabrielle M.G. Pagé^b^, Vina Mohabir^a^, Fiona Campbell^c^ and Jennifer Stinson^c^

^a^The Hospital for Sick Children; ^b^Université de Montréal; ^c^The Hospital for Sick Children; University of Toronto

**Introduction/Aim**: Interdisciplinary pediatric chronic pain clinics (pedCPC) have shown good outcomes, but little is known about individual trajectories through treatment. The aim of this study was to follow youth attending a pedCPC in a major tertiary care hospital to identify and describe pain and mental health trajectories from the time of initial clinic appointment to 12 months after commencing treatment.

**Methods**: Youth (n=386; Female n=302 (78.3%); Mage=14.42, SDage=2.52, 8-19 years) were followed in a pedCPC over the course of their treatment (2018-2022). Youth who had two or more appointments were included [initial consultation and follow-up appointment(s)]. Youth completed questionnaires on pain intensity, pain interference, pain catastrophizing, anxiety, and depression. Growth mixture modeling was used to characterize trajectories on each variable over three timepoints (7 linear trajectory models, 7 linear + quadratic trajectory models). Model selection was based on Bayesian Information Criterion (BIC) and a minimum of 5% of participants classified in each trajectory.

**Results**: Each of the five variables had different associated trajectory models: Average pain intensity had two linear trajectory groups (BIC=3629.72); Pain interference had five linear trajectory groups (BIC=6029.1); Pain catastrophizing had two quadratic trajectory groups (BIC=6485.04); Anxiety had three linear trajectory groups (BIC=6435.2); and Depression had three linear trajectory groups (BIC=6389.88).

**Discussion/Conclusions**: Youth attending a pedCPC have differing trajectories over the course of a year of treatment on measures of pain intensity, pain interference, pain catastrophizing, anxiety and depression. While most youth improved on these metrics, there is a subset who have high scores throughout their treatment.

**Outcomes of triphasic trials of spinal cord stimulation**

Tamiris Soares^a^, Pranab Kumar^b^, Jamal Kara^a^, Victoria Bains^a^ and Anuj Bhatia^a^

^a^TWH, University of Toronto, UHN; ^b^TWH, University of Toronto, UHN

**Introduction/Aim**: We conducted a prospective observational study on patients undergoing SCS trials to evaluate long terms outcomes based on decisions made using the triphasic SCS trial protocol. The three trial SCS phases were low-frequency, paresthesia-based stimulation (PB), high-frequency paresthesia-free stimulation (PF), and placebo (PL) stimulation. The study phases were randomized, and the participants were blinded to the allocation of high-frequency or placebo modes.

**Methods**: IRB approval was obtained (UHN REB # 18-5864). 196 patients diagnosed with refractory back and lower limb neuropathic pain received SCS percutaneous trials between July 2017, to February 2022. The percutaneous trial lasts 12 days and is divided into 3 phases. During the trial, patients were assessed at the end of each phase, which included pain intensity (NRS), DN4, NPSI, PSQ3, GAD-7, PHQ9, and PCS. We also do QST, functional MRI, and magnetoencephalography before, at the end of the trial, 6 months, and 1 year after the implantation.

A positive response was defined as at least a 50% reduction in pain intensity from their baseline.

**Results**: 196 patients proceeded to the trial. Demographic data are detailed. There were 134 responders to the triphasic SCS trials and 62 non-responders. Eighty (80) patients responded to PB and or PF but not with the placebo mode, and only four of 196 responded solely to placebo.

**Discussion/Conclusions**: The findings of our observational prospective study show that active SCS modes (PB and PF) are associated with a decrease in pain intensity when compared with a placebo intervention.

**Implementing CARD (Comfort Ask Relax Distract) for university-based influenza vaccination pop-up clinics: Client and staff feedback**

Anna Taddio^a^, Victoria Gudzak^a^, Charlotte Logeman^b^, Natalie Crown^a^, Joshua LeBlanc^a^, Lisa Dolovich^a^, C. Meghan McMurtry^c^ and Lucie Marisa Bucci^d^

^a^University of Toronto; ^b^Hospital for Sick Children; ^c^University of Guelph; ^d^Bucci-Hepworth Health Services Inc.

**Introduction/Aim**: CARD (Comfort Ask Relax Distract) is an evidence-based vaccination delivery framework that promotes person-centred care. No studies have evaluated its implementation in mass influenza vaccination clinics. The objective was to integrate CARD in university-based influenza vaccination pop-up clinics and evaluate client and staff experiences.

**Methods**: Mixed methods before-and-after quality improvement study. A baseline (control – usual care) phase preceded CARD implementation in one popup clinic. Then CARD was implemented incrementally in two subsequent clinics. Changes to the environment (delineating waiting and aftercare areas, providing privacy, obscuring needles from view), education (clients given CARD coping checklist at check-in), and interactions (defining discrete clinic roles and processes, using coping-promoting behaviours, removing alcohol skin antisepsis prior to vaccine injection) were made. Clients provided feedback using standardized surveys. Staff, including pharmacy student vaccinators, participated in clinic debriefs. Feedback informed real-time process changes during and between clinics.

**Results**: Clinics were held Nov 17 (control), Nov 22 and Nov 24 (both CARD). Feedback from 298 adult vaccine clients, including university students and staff (representing > 98% of all vaccinated individuals) demonstrated an incremental increase in the percent of clients with an improved experience compared to the last vaccination (34% baseline, vs. 49% and 57% in CARD clinics, respectively; p=0.003). Ten percent reported CARD influenced their decision to attend by a moderate amount to a lot. Staff liked the changes. Sample quotes after the final clinic included: “very organized” and “best clinic ever.”

**Discussion/Conclusions**: This study demonstrated improvement in vaccination delivery after CARD implementation as reported by vaccine clients and staff.

**Self-selected favourite music induces analgesia in healthy participants**

Rossi Tomin, Stephanie Bourke, Nilina Mohabir, Majid Saberi, Liat Honigman and Massieh Moayedi

University of Toronto Faculty of Dentistry

**Introduction/Aim**: There is a growing body of literature investigating music-induced analgesia (MIA) as a non-invasive and low-cost option for self-management of pain. Previous methodological approaches exploring MIA have used standardized music to assess their modulatory effects on experimental acute pain and chronic pain. The aim of this study was to investigate the effect of self-selected favourite music on experimental pain intensity compared to pink noise in healthy individuals.

**Methods**: Nineteen healthy participants (9 female, 10 male; 27 ± 4.3 years) were recruited and consented to approved procedures by the University of Toronto research ethics board. Participants received a fluctuating tonic heat stimulus, with a baseline of 32°C and peaks ranging between 43-47°C, on the volar forearm for the duration of the sound stimuli. The order in which participants received the music or pink noise was counterbalanced and randomly assigned. Heat pain intensities were continuously recorded using an electronic visual analog scale (eVAS). The overall pain intensities for each condition were calculated as area under the curve. A two-way repeated measures ANOVA was performed using two factors: music type (two-levels: ‘pink noise’ vs. ‘favourite’) and sex (two-levels: ‘male’ vs ‘female’).

**Results**: Favourite music resulted in significantly lower pain intensity scores compared to pink noise (favourite music: 2129.48 ± 1904.11, Pink Noise: 3026.21 ± 2580.46, p=0.03). Two-way repeated measures ANOVA resulted in a main effect of sex (p=0.866; sex-by-music type interaction: p=0.968).

**Discussion/Conclusions**: This exploratory study supports the hypothesis that self-selected favourite music reduces subjective pain intensity ratings, compared to pink noise.

**Development of the chronic pain pathway, regina area pilot: Resource documents for healthcare providers and patients to support chronic pain management in primary care**

Susan M. Tupper^a^, Jason Vanstone^a^, Crystal Larson^a^, Warren Berry^a^ and Amir Azizian^b^

^a^Saskatchewan Health Authority; ^b^Saskatchewan Health Authority, University of Saskatchewan

**Introduction/Aim**: Primary care providers play a key role in chronic pain management due to the high prevalence of pain and limited access to specialized pain services. Healthcare providers and people living with chronic pain report lack of awareness of treatment resources, high reliance on medication management approaches, and difficulty navigating care. To address these barriers, the Saskatchewan Health Authority (SHA) Department of Clinical Excellence designed resource documents for healthcare providers and patients to support chronic pain management in primary care settings. The iterative patient- and provider-engaged development process is described.

**Methods**: Pathway development began in November, 2019 with a key stakeholder facilitated dialogue with multidisciplinary healthcare providers, patient partners, health services decision makers, and representatives from the Saskatchewan Ministry of Health. Targeted literature reviews and five cycles of feedback informed organization and refinement of the pathway documents. Feedback was integrated from physician specialists, multidisciplinary health care providers with expertise in pain management, family physicians, nurse practitioners, and patient partners.

**Results**: Pathway documents are a Regina area pilot, with plans to scale to other locations across Saskatchewan. Documents contain hyperlinks to assessment tools, health services, pain education resources, and practice support tools. On December 1, 2022, pathway documents were published on the SHA website with a 22-question evaluation survey, education support materials, and electronic medical record upload instructions. Implementation efforts are focused on raising awareness. Evaluation is focused on informing content, clarity, and usability improvements.

**Discussion/Conclusions**: The Chronic Pain Pathway documents support primary care management of chronic pain. Implementation and evaluation are ongoing.

**Virtual or in-person mode of delivery of chronic pain management: What’s your preference?**

Anthony Tutunjian^a^, Jennifer Anthonypillai^b^ and Eleni G. Hapidou^c,d^

^a^McMaster University; ^b^Michael G. DeGroote Pain Clinic, Hamilton Health Sciences; ^c^Michael G. DeGroote Pain Clinic, Hamilton Health Sciences, Department of Psychiatry and Behavioural Neurosciences, McMaster University; ^d^Department of Psychology Neuroscience and Behaviour, McMaster University

**Introduction/Aim**: The COVID-19 pandemic necessitated alternate modes of delivery of chronic pain management programs. The aim of this study was to examine virtual and in-person programs during the pandemic.

**Methods**: Data were collected from the five-week intensive interdisciplinary pain management program (adapted for the pandemic) at the Michael G. DeGroote Pain Clinic, Hamilton Health Sciences (n=100, 69% Virtual, 62% Veterans, 44% females). Participants completed psychometrics on pain intensity (PIS), pain disability (PDI), kinesiophobia (TSK), anxiety (CAS), depression (CES-D), catastrophizing (PCS), sensitivity to pain traumatization (SPTS), pain stages of change (PSOCQ), pain acceptance (CPAQ), and likelihood estimates of return to work (RTW) at admission and discharge, and self-evaluations of program benefit and satisfaction at discharge. 2x2x2X2 mixed ANOVAs on outcomes and 2x2X2 ANOVAs on satisfaction measures were conducted.

**Results**: Both programs produced highly significant outcomes in all (but CAS) measures.

**Discussion/Conclusions**: Results show that virtual delivery of chronic pain management is just as effective as in-person delivery. Previous findings on the benefits of interdisciplinary chronic pain management for all patients are replicated. Results point to the need to have both programs available for patients so as to maximize benefit in all patients.

**How people living with chronic pain and the health system care providers, managers and decision-makers perceive access to integrated care**

Regina Visca^a^, Jeannie Haggerty^b^, Krista Brecht^c^, Yoram Shir^a^ and Alayne Adams^b^

^a^McGill University, McGill University Health Centre; ^b^McGill University; ^c^McGill University Health Centre

**Introduction/Aim**: Although chronic pain centres in Quebec offer multidisciplinary services, access is a challenge due to unacceptably long wait times from excessive patient demand, limited resources, and system inefficiencies. With the broader goal of redesigning care, this study describes challenges in accessing integrated care from the perspectives of patients, providers, managers and policymakers.

**Methods**: We employed an exploratory qualitative study design with diverse stakeholders (N=12) from Quebec pain clinics. Nominal group technique (NGT) was used to analyze patient trajectories with the aim of prioritizing and discussing challenges to accessing integrated care. A structured priority ranking process was applied, and framework analysis was used to synthesize discussions of challenges impacting patient and provider experience, outcomes, and costs.

**Results**: Five overarching challenges were prioritized: 1) inadequate referral structure; 2) failures in ensuring the right service at the right time by the right person; 3) lack of continuity of clinical information; 4) weak service integration; and 5) insufficient patient navigation. Specific challenges that emerged during the discussions acknowledged cross-cutting relationships between the clinical interactions, the design of the delivery system (professional and organizational context), and the larger health system.

**Discussion/Conclusions**: Study findings point to multi-level challenges impeding access to integrated care. Referral processes to needed services must be aligned with integrated care. Actionable information must be available to patients, providers, and decision-makers to ensure resources, structures, and policies support access to integrated care. Using multistakeholder perspectives to understand care gaps, contributes to ensuring the responsiveness of health systems.

**Clinical hypnosis protects against low frequency heart rate variability-associated increases in postsurgical opioid use: Secondary outcome analysis of a randomized controlled trial**

Anna Waisman^a^, Maxwell Slepian^b^, Muhammad Abid Azam^b^, Aliza Weinrib^b^, Brittany Rosenbloom^c^, Hance Clarke^b^ and Joel Katz^a^

^a^York University; ^b^Toronto General Hospital; ^c^The Hospital for Sick Children

**Introduction/Aim**: Heart-rate variability (HRV) is an index of parasympathetic nervous system (PNS) activity inversely related to pain. Postsurgical opioid consumption (POC) is considered a proxy for severe pain. A recent RCT identified that clinical hypnosis (CH) reduces POC and preserves HRV after major oncological surgery. The current study conducts a secondary analysis to examine whether presurgical HRV predicts POC in the context of the CH trial.

**Methods**: Following Toronto General Hospital REB approval, 92 oncological surgery patients randomly received CH before and 1-3 days after surgery or treatment-as-usual (TAU). LF-HRV, a measure of low PNS activity, was assessed before surgery. Opioid dosage in milligram morphine equivalents (MME) was assessed 1-5 days post-surgery.

**Results**: A linear mixed-effect model was conducted with MME as the outcome; group (CH, TAU), LF-HRV, postsurgical timepoint (days 1-5), and a group x LF-HRV interaction for fixed effects; and a random intercept for participants. The main effect of time, F(4,250.07)=7.08 p < 0.001, and the group x LF-HRV interaction were significant, F(1,82.87)=6.90, p=0.01. Post-hoc analyses revealed a significant positive trend of MME for LF-HRV only in the TAU group (simple slope estimate for LF-HRV=0.01; 95%CI=0.003, 0.02; p=0.004). Following a median split of LF-HRV, pairwise contrasts showed that MME in the high, but not low, LF-HRV classification was significantly greater for the TAU vs CH group (p < 0.01).

**Discussion/Conclusions**: The finding in the TAU but not CH group of greater MME in participants with lower PNS tone suggests perioperative CH may have protected against the negative effects of lower PNS tone.

**Diverse forms of social presence in VR for chronic pain**

Yuemei Wu^a^, Timothy Kagiri^a^, Kit-Ying Angela Chong^a^, Chris Shaw^b^, Diane Gromala^c^, Ruoyu Li^a^ and Owen Williamson^d^

^a^Simon Fraser University; ^b^Professor, Simon Fraser University; ^c^Distinguished Professor, Simon Fraser University; ^d^Adjunct Professor, Simon Fraser University

**Introduction/Aim**: Virtual Reality has been demonstrated to be useful for acute pain distraction, managing chronic pain, changing somatosensation, enhancing graded motor imagery, and helping motivate physical activity. However, until recently, the social aspects of chronic pain have been explored far less often. Moreover, most research has been primarily limited to the short-term use of VR. However, given the technology industry’s focus on social interaction in VR, the primacy of biopsychosocial methods in pain research, and the importance of social isolation among chronic pain patients, explorations of the social aspects of VR are highly salient.

**Methods**: We conducted a retrospective analysis of the qualitative findings from our existing studies, analyzing the impact of the social aspects of our VR systems. One component was a mixed methods study of 16 participants (ages 56-89, 8 females; during COVID) diagnosed with chronic pain to measure to what extent a sense of social presence in a virtual environment affected participants’ feelings of welcomeness, safety, and loneliness.

**Results**: In one of our VR systems, we found meaningful increases in welcomeness and safety; Impersonality and Immersion remain unchanged, with a decrease in loneliness. From these study components, we developed a taxonomy of diverse aspects of social presence in VR for the purpose of inferring its utility in addressing social isolation among chronic pain patients.

**Discussion/Conclusions**: While most’ social presence’ in VR implies synchronous interaction with another person, our taxonomy identifies numerous other ways to use asynchronous social presence in VR that may also help alleviate social isolation in Chronic Pain patients.

**Transcranial alternating current stimulation is associated with lower ratings of both tourniquet ischemic pain and low back pain**

Dominic Ysidron and Christopher France

Ohio University

**Introduction/Aim**: Transcranial alternating current stimulation is a promising non-invasive and non-pharmacological approach to pain management, but its effects have yet to be examined in the same sample for both experimental and clinical pain.

**Methods**: In a within-subject design, young adults completed a 5 minute tourniquet ischemia pain task on two counterbalanced testing sessions held one week apart. During one session forearm ischemia was accompanied by active treatment (transcranial alternating current stimulation at 10Hz) while the other was accompanied by sham stimulation (transcranial random noise stimulation at 10Hz). For the full sample (n=24), continuous forearm pain ratings were averaged for minutes 1-5 of ischemia. In addition, for a subsample of participants with chronic low back pain (CLBP, n=15), Brief Pain Inventory ratings of low back pain were assessed before and after stimulation.

**Results**: A repeated measures analysis of forearm pain ratings revealed a significant effect of stimulation, F(1,22)=8.91, p < 0.01, Wilks’ Λ=0.712, with follow-up tests revealing lower pain ratings for active vs. sham stimulation during minute 1, t(23)=-3.02, p < 0.01, and minute 2, t(23)=-2.97, p < 0.01 of ischemia, but not during minutes 3-5. Among those with CLBP, back pain ratings did not differ before stimulation, t(14)=-0.70, p=0.50, but were significantly lower immediately after active versus sham stimulation, t(14)=-2.83, p < 0.05.

**Discussion/Conclusions**: The present findings indicate that transcranial alternating current stimulation may induce temporary reductions in both experimental and clinical pain, suggesting that further testing is needed to better understand now this intervention may be used as an adjunctive approach to pain management.

**Pain relief achieved by pharmacological, physical and psychosocial treatments: A real-world portrait of people living with chronic pain**

Meriem Zerriouh^a^, Sylvie Beaudoin^b^, Christian Bertrand ^b^, M. Gabrielle Pagé^c^, Line Guénette^d^, Lucie Blais^e^ and Anaïs Lacasse^a^

^a^Département des sciences de la santé, Université du Québec en Abitibi-Témiscamingue (UQAT), Rouyn-Noranda, Québec, Canada, Laboratoire de recherche en épidémiologie de la douleur chronique, Université du Québec en Abitibi-Témiscamingue (UQAT), Québec, Canada; ^b^Laboratoire de recherche en épidémiologie de la douleur chronique, Université du Québec en Abitibi-Témiscamingue (UQAT), Québec, Canada; ^c^Centre de recherche du Centre hospitalier de l’Université de Montréal (CRCHUM), Montréal, Québec, Canada, Département d’anesthésiologie et de médecine de la douleur, Faculté de médecine, Université de Montréal, Montréal, Québec, Canada; ^d^Faculté de pharmacie, Université Laval, Québec, Québec, Canada,, Centre de recherche du CHU de Québec - Université Laval, Québec, Québec; ^e^Faculté de pharmacie, Université de Montréal, Montréal, Québec, Canada

**Introduction/Aim**: The efficacy of individual pain treatment approaches is often evaluated in randomized clinical trials and few observational studies have portrayed the overall relief experienced by persons living with chronic pain (CP) in the community. We aimed to describe relief brought by pain treatments used in real-world clinical settings and identify clinical and sociodemographic factors associated with greater pain relief.

**Methods**: We have used the COPE Cohort, a database of adults living with CP across Quebec (Canada) which included a self-reported measure of the overall pain relief brought by the different treatments currently used by participants (0-100% numeric rating scale). A multivariable logistic regression model was used to identify factors associated with ≥70% pain relief.

**Results**: Mean age of participants was 50 years and 83.7% self-identified as women (n=1419); 21.8% reported ≥70% pain relief. Adjusting for potential confounders, the following variables were associated with greater chances of reporting ≥70% pain relief: less severe pain characteristics, reporting a stressful event as the circumstance surrounding the onset of pain, living with CP for ≥10 years, access to a trusted healthcare professional for pain management, receiving disability benefits, and greater self-perceived general health. Variables associated with lower chances of ≥70% pain relief included repetitive work as the cause of pain, using non-pharmacological approaches or over-the-counter pain medications, being born in Canada, feminine personality traits, and greater psychological distress.

**Discussion/Conclusions**: Despite our cross-sectional design, our results still allow us to emphasize and focus on modifiable factors as priorities for improving patient well-being.

**Peripheral magnetic stimulation in the treatment of postoperative pain: A systematic review and meta-analysis**

Stephanie Park M.Sc^a^, Rex Park M.D^b^, Duncan Westwood B.A^b,c^, Massieh Moayedi Ph.D^d,e,f^ and James S. Khan M.D M.Sc^b,c^

^a^Temerty Faculty of Medicine, University of Toronto, Toronto, ON, Canada; ^b^Department of Anesthesiology and Pain Medicine, University of Toronto, Toronto, ON, Canada; ^c^Mount Sinai Hospital, Toronto ON, Canada; ^d^Centre for Multimodal Sensorimotor and Pain Research, Faculty of Dentistry, University of Toronto, ON, Canada; ^e^University of Toronto Centre for the Study of Pain, Toronto, ON, Canada; ^f^Department of Dentistry, Mount Sinai Hospital, Toronto, ON, Canada

**Introduction/Aim**: Management of postoperative pain continues to be a major challenge. There is a pressing need for novel therapies that are both safe and effective. One promising modality is peripheral magnetic stimulation (PMS). Here, we aimed to systematically review the effects of PMS on postoperative pain management.

**Methods**: MEDLINE, CENTRAL, EMBASE, ProQuest Dissertations, and clinicaltrials.gov were searched up to May 2021. We included studies of any design with (1) patients ≥18 years, (2) undergoing any surgery, (3) had PMS within the perioperative period and (4) assessed for postoperative pain outcomes. We conducted a meta-analysis of RCTs if ≥2 trials reported pain outcomes at the same follow-up.

**Results**: Eighteen studies (17 RCTs, 1 nonrandomized) were included and we found that PMS was more effective than controls within the first 7 postoperative days (mean difference [MD] -1.64 on a 10-point numerical rating scale, 95% confidence interval [CI] -2.08 to -1.20, I2=77%, 6 studies [231 patients]), 1-month post-surgery (MD -1.82, 95% CI -2.48 to -1.17, I2=0%, 3 studies [104 patients]), and 2-months post-surgery (MD -1.96, 95% CI -3.67 to -0.26, I2=84%, 3 studies [104 patients]). There was no effect on pain at 6 and 12-months post-surgery. Ten studies reported no major differences in adverse events between PMS and control groups.

**Discussion/Conclusions**: Our findings suggests that PMS is effective at reducing postoperative pain up to 2 months post-surgery. Results are limited by heterogeneity and small sample sizes. Larger trials are needed to identify the definitive effects of PMS in the perioperative period.

**Peripheral magnetic stimulation in the treatment of postoperative pain: A systematic review and meta-analysis**

Stephanie Park MSc^a^, Rex Park MD^b^, Duncan Westwood BA^b,c^, Massieh Moayedi PhD^d,e,f^ and James S. Khan M.D MSc^b,c^

^a^Temerty Faculty of Medicine, University of Toronto, Toronto, ON, Canada; ^b^Department of Anesthesiology and Pain Medicine, University of Toronto, Toronto, ON, Canada; ^c^Mount Sinai Hospital, Toronto ON, Canada; ^d^Centre for Multimodal Sensorimotor and Pain Research, Faculty of Dentistry, University of Toronto, ON, Canada; ^e^University of Toronto Centre for the Study of Pain, Toronto, ON, Canada; ^f^Department of Dentistry, Mount Sinai Hospital, Toronto, ON, Canada

**Introduction/Aim**: Management of postoperative pain continues to be a major challenge. There is a pressing need for novel therapies that are both safe and effective. One promising modality is peripheral magnetic stimulation (PMS). Here, we aimed to systematically review the effects of PMS on postoperative pain management.

**Methods**: MEDLINE, CENTRAL, EMBASE, ProQuest Dissertations, and HYPERLINK “http://clinicaltrials.gov”clinicaltrials.gov were searched up to May 2021. We included studies of any design with (1) patients ≥18 years, (2) undergoing any surgery, (3) had PMS within the perioperative period and (4) assessed for postoperative pain outcomes. We conducted a meta-analysis of RCTs if ≥2 trials reported pain outcomes at the same follow-up.

**Results**: Eighteen studies (17 RCTs, 1 nonrandomized) were included and we found that PMS was more effective than controls within the first 7 postoperative days (mean difference [MD] -1.64 on a 10-point numerical rating scale, 95% confidence interval [CI] -2.08 to -1.20, I2=77%, 6 studies [231 patients]), 1-month post-surgery (MD -1.82, 95% CI -2.48 to -1.17, I2=0%, 3 studies [104 patients]), and 2-months post-surgery (MD -1.96, 95% CI -3.67 to -0.26, I2=84%, 3 studies [104 patients]). There was no effect on pain at 6 and 12-months post-surgery. Ten studies reported no major differences in adverse events between PMS and control groups.

**Discussion/Conclusions**: Our findings suggests that PMS is effective at reducing postoperative pain up to 2 months post-surgery. Results are limited by heterogeneity and small sample sizes. Larger trials are needed to identify the definitive effects of PMS in the perioperative period.

